# Synthesis of (−)‐Dihydroraputindole D by Enantioselective Benzoylation of a 1,3‐Diol Intermediate

**DOI:** 10.1002/chem.202002579

**Published:** 2020-09-16

**Authors:** Marvin Fresia, Mario Kock, Thomas Lindel

**Affiliations:** ^1^ Institute of Organic Chemistry Technical University Braunschweig Hagenring 30 38106 Braunschweig Germany

**Keywords:** asymmetric synthesis, desymmetrization, gold catalysis, indole alkaloids, natural products

## Abstract

The enantioselective synthesis of (−)‐dihydroraputindole D is reported. The key step is the desymmetrizing benzoylation of a prochiral 1,3‐diol employing Trost′s ProPhenol catalyst system, which has been applied for the first time to a cyclic molecule carrying geminal hydroxymethyl groups. The cyclopenta[*f*]indoline system was assembled by Au(I)‐catalyzed cyclization of an alkynylated indoline precursor. (−)‐Dihydroraputindole D was obtained in 17 steps and 8% overall yield starting from dihydroxyacetone. In combination with quantum chemical calculations of the ECD spectra, our synthesis allowed us to determine the absolute configuration (5*S*,7*R*) of the natural product (+)‐raputindole D from the Rutaceous plant *Raputia simulans*.

## Graphical abstract

**Enantioselective desymmetrization paves the way**: The cyclopenta[*f*]indoline system of (−)‐dihydroraputindole D was assembled by Au^I^‐catalyzed cyclization. Desymmetrizing benzoylation of an achiral 1,3‐diol intermediate was possible with a Trost's ProPhenol catalyst system, which was applied for the first time to a cyclic molecule carrying geminal hydroxymethyl groups.

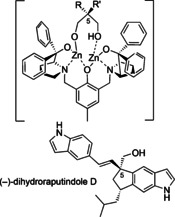

The unique indole alkaloids raputindole A–D were isolated from the neotropical tree *Raputia simulans* Kallunki (Rutaceae) from Peru.^1^ Biosynthetically, the characteristic cyclopenta[*f*]indole moiety of the raputindoles is probably formed by oxidative coupling of 5‐, 6‐, or 7‐prenylindole, as suggested by the occurrence of 5‐ and 7‐prenylindole in the same species.^2^ The enamine section of the indole moiety of the raputindoles is not substituted, a feature that can be found in only a few other natural products such as the raputimonoindoles,^2^ the caulindoles,^3^ the trikentrines,^4^ and the herbindoles.^5^ The cyclopenta[*f*]indole moiety can also be found in the nodulisporic acids,^6^ shearinines,^7^ and janthitrems.^8^ Biological activity was reported only for desoxyraputindole C from *Raputia praetermissa*; it induces cell‐cycle arrest, likely by binding to cathepsin L (IC_50_ 1.7 μm).^9^ However, not all raputindoles were tested.

In the course of our total synthesis of raputindole A (**1**), we developed the regioselective Au^I^‐catalyzed cyclization of 6‐alkynylindole precursors as the key step in constructing the cyclopenta[*f*]indole system.^10^ Having the quaternary center installed, diastereoselectivity of the synthesis was achieved by tethered Ir‐catalyzed hydrogenation of an intermediate cyclopentene‐containing tricycle.^11^ However, our attempts to assemble the quaternary center in an enantioselective manner by using chiral Au^I^ complexes remained unsuccessful.

For raputindole D (**2**), optical activity was reported without determination of the absolute configuration. Raputindole D differs from raputindole A by the presence of a hydroxymethyl instead of a methyl group at the quaternary center (C5, Scheme 1). This led us to the idea of exploiting the 1,3‐diol moiety of cyclopenta[*f*]indoline **5** for an enantioselective desymmetrization strategy. Among the few existing methods, the use of Trost's ProPhenol catalyst **4** appeared to be most promising, as the benzoylation of 2‐monosubstituted 1,3‐diols had provided yields and enantioselectivities superior to those accessible by enzymatic methods.^12^

**Scheme 1:**
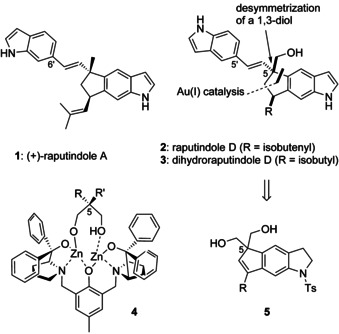
Structures of raputindoles A (**1**) and D (**2**) from the Peruvian rutaceous plant *R. simulans*, model of a possible ProPhenol catalyst–substrate complex, and synthetic strategy.

In the case of indole **5**, it was unclear how catalyst **4** would behave both in terms of reactivity and enantioselectivity. In the mechanism proposed by Trost et al., the enantiodifferentiation of the two hydroxymethyl groups is possible because the carbonyl oxygen of the benzoyl source—vinyl benzoate—coordinates to the zinc center from the sterically clearly preferred side, where the hydrogen substituent is located (R′=H, Scheme 1).12b However, differing from all earlier examples, our envisaged substrate **5** contains a quaternary carbon in the 2‐position of the 1,3‐diol moiety.

The synthesis of tricyclic 1,3‐diol **11** starts from dihydroxyacetone (**6**) that, after double TIPS protection, underwent alkynylation to the tertiary propargylic alcohol **7** (Scheme 2). Sonogashira coupling with *N*‐TIPS‐6‐iodoindoline (**8**, 5 steps from *p*‐toluidine)^13^ and subsequent DMAP‐catalyzed acetylation (86 h) of the sterically hindered tertiary alcohol provided propargylic acetate **9**. Excess alkyne coupling partner was also acetylated and removed after the subsequent step. Gold‐catalyzed^14^ cyclization (1 mol % of Au(PPh_3_)NTf_2_) afforded the cyclopenta[*f*]indoline as the alkenyl acetate, which was saponified to ketone **10** (NaOMe), exhibiting blue fluorescence (*λ*_em max_=429 nm in THF). Based on our earlier experience with the total synthesis of raputindole A, the *N*‐TIPS protecting group was replaced by tosyl, which was required for the envisaged introduction of the isobutenyl side chain by Suzuki–Miyaura coupling. Ketone **10** was triflated (LHMDS, PhNTf_2_), N‐desilylated (2 m HCl), and the resulting indoline was N‐tosylated. Finally, double O‐desilylation afforded cyclopenta[*f*]indoline **11** with two hydroxymethyl groups.

**Scheme 2:**
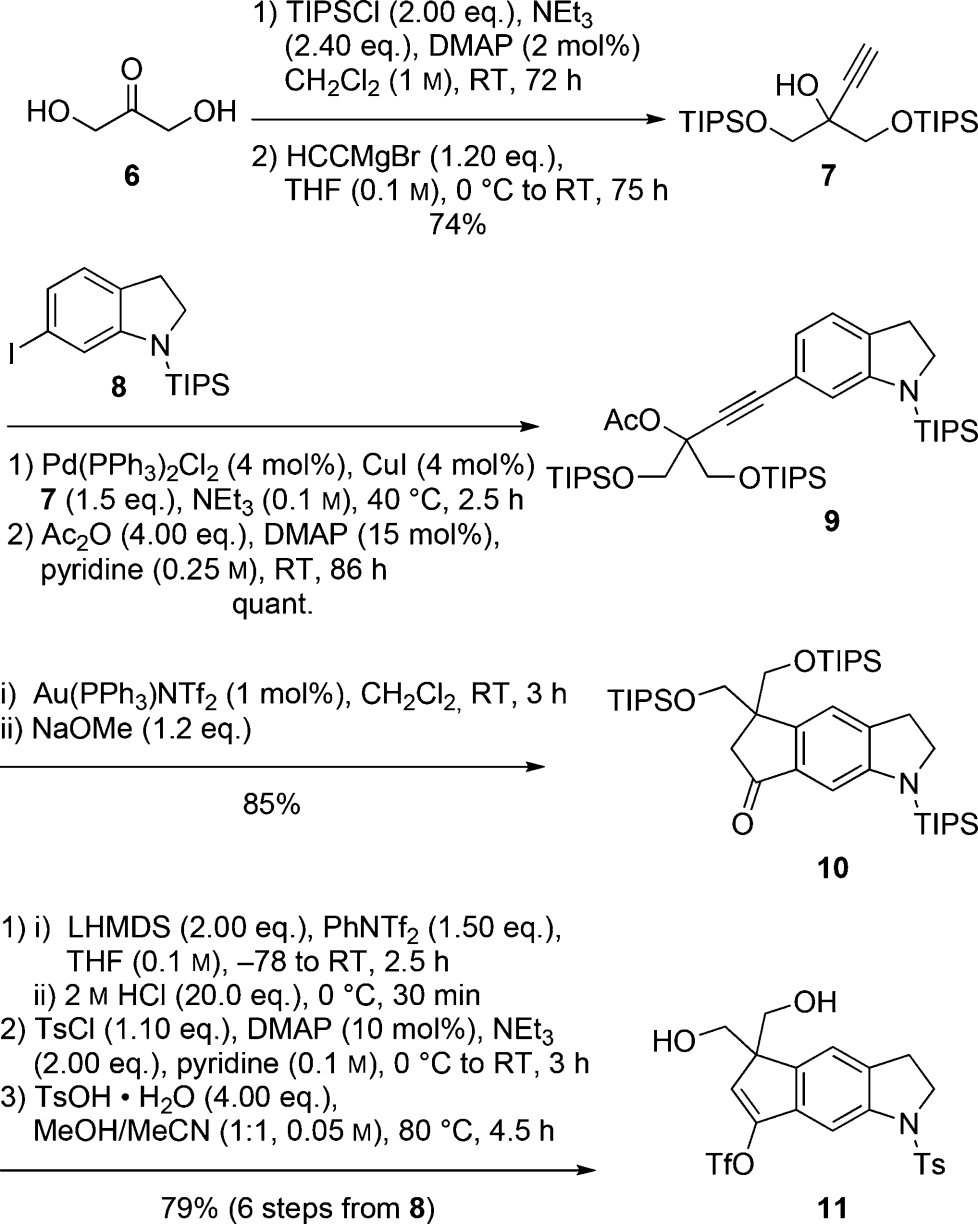
Synthesis of tricyclic 1,3**‐**diol **11** by Au^I^ **‐**catalyzed cyclopentannulation of propargylic acetate **9**.

For the desymmetrizing, enantioselective O‐monobenzoylation of **11**, we employed a dinuclear zinc asymmetric catalyst developed by the Trost group (Scheme 3).^12^ Diol **11** was treated with vinyl benzoate in the presence of Et_2_Zn and (*S*,*S*)‐ProPhenol ((*S*,*S*)‐**12**, 2:1, 5 mol %) to afford monoester **13** (91 %). The enantiomeric ratio of triflate **13** (84:16) was determined by HPLC on a chiral column (Lux amylose‐2, *n*‐hexane/EtOH (75:25)). Suzuki–Miyaura coupling of **13** with isobutenylboronic acid afforded diene **15** in high yield (90 %). Changing the order of benzoylation and cross coupling gave a lower yield in the coupling step (70 %) and a lower *er* of **15** (79:21, determined by ^19^F NMR spectroscopy of the (*R*)‐Mosher ester).

**Scheme 3:**
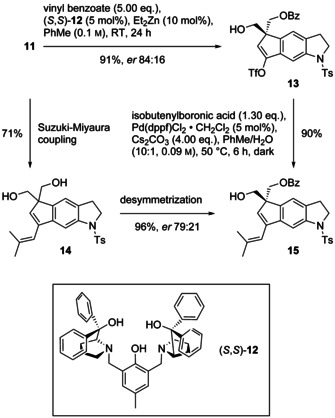
Desymmetrization of 1,3‐diol **11** by enantioselective benzoylation employing Trost's catalyst (*S*,*S*)‐**12**/Et_2_Zn (1:2), followed by Suzuki–Miyaura cross coupling.

To our surprise, the assignment of the absolute configuration of tricyclic benzoate **15** by quantum chemical calculation of the ECD (TDDFT, ωB97XD/TApr‐cc‐pVDZ) proved to be difficult. Fortunately, the situation was clear for the tricyclic alkenyl triflate **13**, for which the configuration shown in Scheme 3 was unambiguously assigned by ECD calculation (see the Supporting Information). The prediction of the stereochemical outcome of the benzoylation was not possible based on analogy with existing examples. Compounds **13** and **14** constitute the first examples with a quaternary center in the 2‐position.

The successful monobenzoylation of **11** gave us the opportunity to exploit the remaining primary hydroxy group as a tether for the diastereoselective Crabtree hydrogenation of tricyclic diene **15**. To our surprise it proved to be necessary to use the high amount of 28 mol % of Crabtree catalyst [Ir(COD)py(PCy_3_)]BARF^15^ to achieve a satisfactory degree of conversion (Table 1). It was impossible to achieve the selective monohydrogenation of **15**. Even at −20 °C, the major product was the isobutyl‐substituted indane derivative **17** (79 %), which was accompanied with isobutenyl indane **16**. At −40 °C, we observed only minimal conversion, whereas at 0 °C, dihydrogenation of had taken place exclusively.

**Table 1:** Hydrogenation of **15**.^[a]^

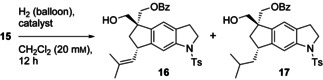						
	Catalyst	[mol %]	*T* [°C]	**15**	**16**	**17**
1	[Ir(COD)py(PCy_3_)]PF_6_	7	0	100	0	0
2	[Ir(COD)py(PCy_3_)]PF_6_	7	RT	100	0	0
3	[Rh(NBD)(dppb)]BF_4_	15	RT	no reaction^[b]^		
4	Pd/C	15	RT	full conversion^[b,c]^		
5	[Rh(COD)py(PCy_3_)]BARF	7	RT	no reaction^[b]^		
6	[Ir(COD)py(PCy_3_)]BARF	7	RT	no reaction^[b]^		
7	[Ir(COD)py(PCy_3_)]BARF	28	RT	0	0	100
8	[Ir(COD)py(PCy_3_)]BARF	28	0	0	0	100
9	[Ir(COD)py(PCy_3_)]BARF	28	−20	0	21	79
10	[Ir(COD)py(PCy_3_)]BARF	28	−40	low conversion^[b]^		
11	[Ir(COD)((*S*)‐*t*Bu‐PHOX)]BARF	7	RT	no reaction^[b]^		

[a] Product ratios determined by ^1^H NMR spectroscopy are given. All reactions were performed in dry CH_2_Cl_2_ (1 mL) under H_2_ overnight (12–14 h) on a 10 mg/0.02 mmol scale. [b] Determined by TLC. [c] Products not identified.

When starting from diol **14**, the hydrogenation was much faster, but it was still impossible to avoid reduction of the isobutenyl side chain. The best result was obtained when using [Ir(COD)py(PCy_3_)]PF_6_, providing a 1:2 mixture of mono‐ and dihydrogenated products, accompanied by traces of starting material. Chiral hydrogenation catalysts (entries 6–8, Table 2) were not successful, either.

**Table 2:** Hydrogenation of **14**.^[a]^

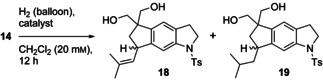						
	Catalyst	[mol %]	*T* [°C]	**14**	**18**	**19**
**1**	[Ir(COD)py(PCy_3_)]PF_6_	5	RT	0	0	100
**2**	[Ir(COD)py(PCy_3_)]PF_6_	5	0	0	0	100
**3**	[Ir(COD)py(PCy_3_)]PF_6_	5	−20	3	31	66
**4**	[Ir(COD)py(PCy_3_)]PF_6_	5	−30	3	34	63
**5**	[Ir(COD)py(PCy_3_)]PF_6_	5	−40	61	17	22
**6**	[Ru((*R*)‐BINAP)](OAc)_2_	5	RT	no reaction^[b]^		
**7**	Ir(COD)((*S*)‐*t*Bu‐PHOX)]BARF	5	RT	see text		
**8**	[Ir(COD)((*S*)‐*t*Bu‐PHOX)]BARF	5	0	no reaction^[b]^		

[a] Product ratios determined by ^1^H NMR spectroscopy are given. All reactions were performed in dry CH_2_Cl_2_ (1 mL) under H_2_ overnight (12–14 h) on a 10 mg/0.02 mmol scale. [b] Determined by TLC.

To our surprise, hydrogenation of **14** in the presence of [Ir(COD)(*S*)‐*t*Bu‐PHOX)]BARF (5 mol %) on the 10 mg scale afforded the diastereomeric Diels–Alder dimers **20 a** (59 %) and **20 b** (7 %) as racemic major products (Scheme 4), the structures of which were elucidated by extensive 2D NMR spectroscopy. In both cases, we observed a singlet for the aliphatic methine‐H of the cyclohexene moiety (*δ*=3.15, 3.14 ppm), which excludes the alternative regiochemistry. The decisive NOESY correlation allowing the assignment of the major diastereomer was observed between 6‐H and 8′‐H. By DFT calculation (B3LYP/6‐31G(d)), diastereomer **20 a** is more stable than **20 b**. For the formation of **20 a** and **20 b**, half of the starting material must have undergone isomerization of the isobutenylcyclopentene to a 2‐methylallylidene moiety. One of the rare examples, where this behavior occurred when employing a Crabtree catalyst under hydrogenation conditions, was described by Guillou et al.^16^ who reported the isomerization of an exocyclic methenyl double bond to the endocyclic position. The hydrogen served only as activator of the Crabtree catalyst, but was not incorporated.

**Scheme 4:**
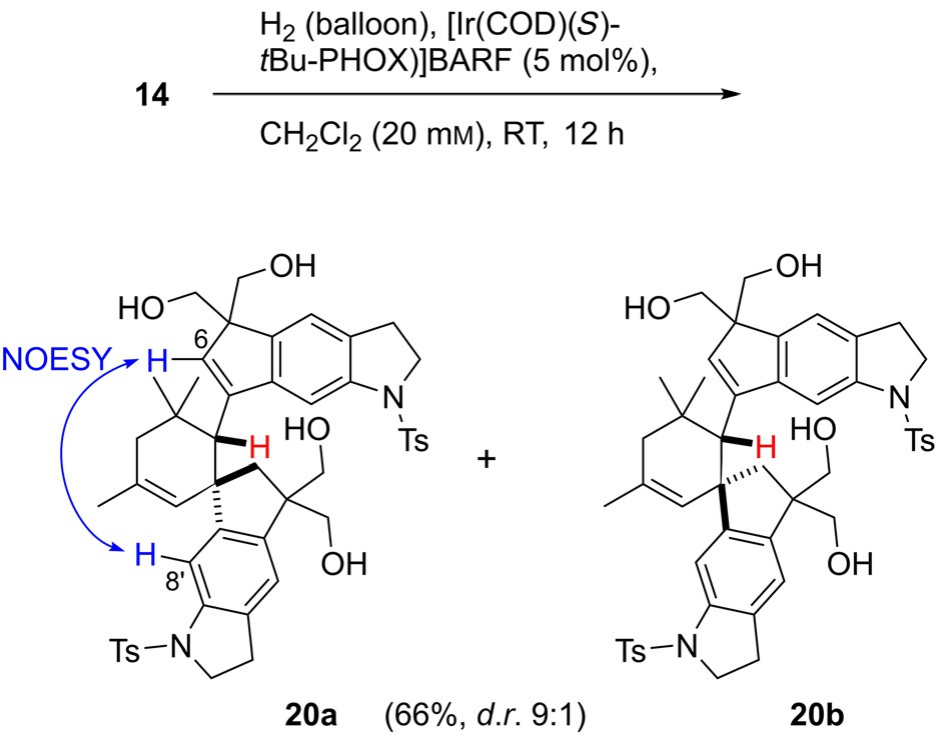
Formation of diastereomeric Diels–Alder dimers **20 a** and **20 b** upon attempted enantioselective hydrogenation of 1,3‐diol **14**.

Given the difficulties experienced with the monohydrogenation of **15**, we decided to pursue the enantioselective synthesis of dihydroraputindole D (**3**), which differs from the natural product by the presence of an isobutyl instead of an isobutenyl side chain.

It was unclear, whether the benzoyloxy group of **17** would be compatible with the Takai/Suzuki–Miyaura route that was envisaged for the installation of the second indole moiety. Moreover, it seemed to be interesting to compare the optical properties of a hitherto unknown dihydroraputindole D with those of **2**, for which the absolute configuration has remained undetermined. Dihydrogenated product **17** was obtained from **15** in 93 % yield and oxidized to the aldehyde (IBX), followed by Takai olefination to (*E*)‐iodoalkene **21** (Scheme 5). Suzuki–Miyaura coupling with indol‐5‐ylboronic acid proceeded smoothly (91 %) and provided the complete skeleton of raputindole D. Saponification (LiOH), reductive detosylation (Na/naphthalene), and dehydrogenation (Pd/C) afforded (−)‐dihydroraputindole D in 8 % overall yield over 17 steps starting from dihydroxyacetone (**6**). The enantiomeric ratio of (−)‐dihydroraputindole D (**3**, 90:10) was determined by HPLC on a chiral column (Lux Cellulose‐1, *n*‐hexane/EtOH (85:15)).

**Scheme 5:**
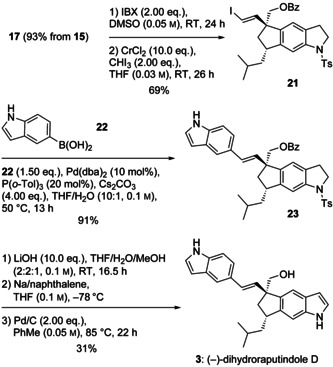
Endgame from alcohol **17** to (−)‐dihydroraputindole D (**3**).

Quantum chemical calculation (TDDFT, ωB97XD/TApr‐cc‐pVDZ) of the ECD spectrum allowed the assignment of the absolute configuration of (−)‐dihydroraputindole D (**3**). The calculated spectra agreed well with the experimental data of the synthesized product (Figure 1, see also the Supporting Information). For the natural product (+)‐raputindole D (**2**), which differs from **3** by the presence of an isobutenyl instead of an isobutyl side chain, there were no ECD spectra reported. Because we could show that the ECD spectrum of (−)‐**3** can be calculated quantum chemically, we calculated the ECD spectrum of (+)‐**2** (Figure 1), which proved to be almost the mirror image of that of (−)‐**3**. For the enantiomers of all three compounds **2**, **3**, and **13**, we also calculated to optical rotatory power the signs of which agreed with experimental values (DFT, ωB97XD/TApr‐cc‐pVDZ). Thus, we conclude that the natural product (+)‐raputindole D ((+)‐**2**) has the configuration (5*S*,7*R*). The absolute configuration of (+)‐**2** corresponds to that of the desoxy form, (+)‐**1**, which we had determined after separation of racemic synthetic material by HPLC on a chiral column.

**Figure 1:**
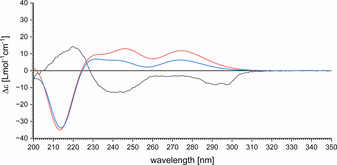
Comparison of the experimental ECD spectrum of **3** (black) with the calculated ECD spectra of (5*S*,7*S*)‐**3** (red) and of the natural product (5*S*,7*R*)‐**2** (blue, TDDFT, ωB97XD/TApr‐cc‐pVDZ).

In summary, we report the first enantioselective synthesis of a raputindole derivative. Key steps are the Au^I^‐catalyzed cyclization forming the cyclopenta[*f*]indoline system and the enantioselective benzoylation of the achiral tricyclic 1,3‐diol **11** employing Trost's ProPhenol catalyst system. Thus, in addition the synthesis dihydroraputindole D, our approach also addresses a hitherto unexplored type of substrate for ProPhenol‐type catalysts. Desymmetrization of the 1,3‐diol proved to be superior to hydrogenation employing a chiral Crabtree catalyst that led to isomerization and surprising dimerization of the 1,3‐diol precursor. (−)‐Dihydroraputindole D (**3**) was obtained in 17 steps and 8 % overall yield starting from dihydroxyacetone. Our synthesis also allowed us to determine the absolute configuration of the natural product (+)‐raputindole D (**2**) from the Rutaceous plant *R. simulans*.

## Note added in proof

After revision of this manuscript, a new total synthesis of (+)‐raputindole A was reported.^17^

## Conflict of interest

The authors declare no conflict of interest.

## Supporting information

As a service to our authors and readers, this journal provides supporting information supplied by the authors. Such materials are peer reviewed and may be re‐organized for online delivery, but are not copy‐edited or typeset. Technical support issues arising from supporting information (other than missing files) should be addressed to the authors.

Supplementary
