# Supplementary material for: Synthesis of (−)‐Dihydroraputindole D by Enantioselective Benzoylation of a 1,3‐Diol Intermediate
Source: Chemistry. 2020 Sep 16;26(56):12733–7. doi: 10.1002/chem.202002579 (PMC7590097; doi:10.1002/chem.202002579)
Supplement: Supplementary file 1 — Supplementary [file CHEM-26-12733-s001.pdf]

Chemistry–A European Journal

Supporting Information

Synthesis of (−)-Dihydroraputindole D by Enantioselective Benzoylation of a 1,3-Diol Intermediate

Marvin Fresia, Mario Kock, and Thomas Lindel*[a]

Contents

1 General information **S4**
1.1 General Methods S4
1.2 Materials S4
1.3 Instrumentation S4
1.4 Computional Methods S5

2 Experimental procedures and analytical data **S6**
2.1 6-Ethynyl-3,3,9,9-tetraisopropyl-2,10-dimethyl-4,8-dioxa-3,9-disilaundecan-6-ol (**7**) S6
2.2 3,3,9,9-Tetraisopropyl-2,10-dimethyl-6-((1-(triisopropylsilyl)indolin-6-yl)ethynyl)-4,8-dioxa-3,9-disilaundecan-6-yl acetate (**9**) S7
2.3 1-(Triisopropylsilyl)-5,5-bis(((triisopropylsilyl)oxy)methyl)-2,3,5,6-tetrahydrocyclopenta[*f*]indol-7(1*H*)-one (**10**) S8
2.4 5,5-Bis(hydroxymethyl)-1-tosyl-1,2,3,5-tetrahydrocyclopenta[*f*]indol-7-yl trifluormethansulfonate (**11**) S9
2.5 (7-(2-Methylprop-1-en-1-yl)-1-tosyl-1,2,3,5-tetrahydrocyclopenta[*f*]indole-5,5-diyl)-dimethanol (**14**) S10
2.6 (*S*)-(5-(Hydroxymethyl)-1-tosyl-7-(((trifluoromethyl)sulfonyl)oxy)-1,2,3,5-tetrahydro-cyclopenta[*f*]indol-5-yl)methyl benzoate (**13**) S11
2.7 (*rac*)-(5-Hydroxymethyl)-1-tosyl-(((trifluoromethyl)sulfonyl)oxy)-1,2,3,5-tetrahydro-cyclopenta[*f*]indol-5-yl)methyl benzoate ((*rac*)-13) S12
2.8 Dimer **20a** and dimer **20b** S12
2.9 (*S*)-(5-(Hydroxymethyl)-7-(2-methylprop-1-en-1-yl)-1-tosyl-1,2,3,5-tetrahydrocyclopenta[*f*]indol-5-yl)methyl benzoate (**15**) S14
2.10 ((5*S*,7*R*)-5-(Hydroxymethyl)-7-isobutyl-1-tosyl-1,2,3,5,6,7-hexahydrocyclopenta[*f*]-indol-5-yl)methyl benzoate (**17**) S15
2.11 ((5*R*,7*R*)-5-Formyl-7-isobutyl-1-tosyl-1,2,3,5,6,7-hexahydrocyclopenta[*f*]indol-5-yl)-methyl benzoate (**S1**) S16
2.12 ((5*R*,7*R*)-5-((*E*)-2-Iodovinyl)-7-isobutyl-1-tosyl-1,2,3,5,6,7-hexahydrocyclopenta[*f*]-indol-5-yl)methyl benzoate (**21**) S17
2.13 ((5*R*,7*R*)-5-((*E*)-2-(1*H*-Indol-5-yl)vinyl)-7-isobutyl-1-tosyl-1,2,3,5,6,7-hexahydrocy-clopenta[*f*]indol-5-yl)methyl benzoate (**23**) S18
2.14 ((5*R*,7*R*)-5-((*E*)-2-(1*H*-Indol-5-yl)vinyl)-7-isobutyl-1-tosyl-1,2,3,5,6,7-hexahydrocy-clopenta[*f*]indol-5-yl)methanol (**S2**) S19
2.15 ((5*R*,7*R*)-5-((*E*)-2-(1*H*-indol-5-yl)vinyl)-7-isobutyl-1,2,3,5,6,7-hexahydrocyclopen-ta[*f*]indol-5-yl)methanol (**S3**) S20
2.16 ((5*R*,7*R*)-5-((*E*)-2-(1*H*-indol-5-yl)vinyl)-7-isobutyl-1,5,6,7-tetrahydrocyclopenta[*f*]in-dol-5-yl)methanol (**3**) S21

3 Chiral phase HPLC **S23**
3.1 (*rac*)-Benzoate (*rac*)-13 . . . . S23
3.2 Benzoate 13 . . . . S24
3.3 Dihydroraputindole D (3) S25

4 ECD spectra and optical rotations **S26**

5 NMR spectra of new compounds **S29**
5.1 6-Ethynyl-3,3,9,9-tetraisopropyl-2,10-dimethyl-4,8-dioxa-3,9-disilaundecan-6-ol (**7**) S29
5.2 3,3,9,9-Tetraisopropyl-2,10-dimethyl-6-((1-(triisopropylsilyl)indolin-6-yl)ethynyl)-4,8-dioxa-3,9-disilaundecan-6-yl acetate (**9**) S30
5.3 1-(Triisopropylsilyl)-5,5-bis(((triisopropylsilyl)oxy)methyl)-2,3,5,6-tetrahydrocyclopenta[*f*]indol-7(1*H*)-one (**10**) S31
5.4 5,5-Bis(hydroxymethyl)-1-tosyl-1,2,3,5-tetrahydrocyclopenta[*f*]indol-7-yl trifluormethansulfonate (**11**) S32
5.5 (7-(2-Methylprop-1-en-1-yl)-1-tosyl-1,2,3,5-tetrahydrocyclopenta[*f*]indole-5,5-diyl)dimethanol (**14**) S34
5.6 Dimer **20a** S35
5.7 Dimer **20b** S36
5.8 (*S*)-(5-(Hydroxymethyl)-1-tosyl-7-(((trifluoromethyl)sulfonyl)oxy)-1,2,3,5-tetrahydrocyclopenta[*f*]indol-5-yl)methyl benzoate (**13**) S37
5.9 (*S*)-(5-(Hydroxymethyl)-7-(2-methylprop-1-en-1-yl)-1-tosyl-1,2,3,5-tetrahydrocyclopenta[*f*]indol-5-yl)methyl benzoate (**15**) S39
5.10 ((5*S*,7*R*)-5-(Hydroxymethyl)-7-isobutyl-1-tosyl-1,2,3,5,6,7-hexahydrocyclopenta[*f*]indol-5-yl)methyl benzoate (**17**) S40
5.11 ((5*R*,7*R*)-5-Formyl-7-isobutyl-1-tosyl-1,2,3,5,6,7-hexahydrocyclopenta[*f*]indol-5-yl)methyl benzoate (**S1**) S41
5.12 ((5*R*,7*R*)-5-((*E*)-2-Iodovinyl)-7-isobutyl-1-tosyl-1,2,3,5,6,7-hexahydrocyclopenta[*f*]indol-5-yl)methyl benzoate (**21**) S42
5.13 ((5*R*,7*R*)-5-((*E*)-2-(1*H*-Indol-5-yl)vinyl)-7-isobutyl-1-tosyl-1,2,3,5,6,7-hexahydrocyclopenta[*f*]indol-5-yl)methyl benzoate (**23**) S43
5.14 ((5*R*,7*R*)-5-((*E*)-2-(1*H*-Indol-5-yl)vinyl)-7-isobutyl-1-tosyl-1,2,3,5,6,7-hexahydrocyclopenta[*f*]indol-5-yl)methanol (**S2**) S44
5.15 ((5*R*,7*R*)-5-((*E*)-2-(1*H*-indol-5-yl)vinyl)-7-isobutyl-1,2,3,5,6,7-hexahydrocyclopenta[*f*]indol-5-yl)methanol (**S3**) S45
5.16 ((5*R*,7*R*)-5-((*E*)-2-(1*H*-indol-5-yl)vinyl)-7-isobutyl-1,5,6,7-tetrahydrocyclopenta[*f*]indol-5-yl)methanol (**3**) S46

6 Computational results **S47**
6.1 Dimer **20a**: rel. energy 0.00 kcal mol^{-1} S47
6.2 Dimer **20b**: rel. energy 14.16 kcal mol^{-1} S49
6.3 Triflate **13** S51
6.3.1 Conformation 1 (internal code: M0022): rel. energy 1.52 kcal mol^{-1} S51
6.3.2 Conformation 2 (internal code: M0129): rel. energy 0.34 kcal mol^{-1} S52
6.3.3 Conformation 3 (internal code: M0151): rel. energy 1.48 kcal mol^{-1} S53
6.3.4 Conformation 4 (internal code: M0153): rel. energy 1.48 kcal mol^{-1} S54
6.3.5 Conformation 5 (internal code: M0171): rel. energy 0.05 kcal mol^{-1} S55
6.3.6 Conformation 6 (internal code: M0194): rel. energy 0.04 kcal mol^{-1} S56
6.3.7 Conformation 7 (internal code: M0226): rel. energy 1.30 kcal mol^{-1} S57
6.3.8 Conformation 8 (internal code: M0231): rel. energy 0.00 kcal mol^{-1} S58
6.3.9 Conformation 9 (internal code: M0257): rel. energy 0.50 kcal mol^{-1} S59
6.3.10 Conformation 10 (internal code: M0264): rel. energy 1.30 kcal mol^{-1} . . . S60

6.3.11 Conformation 11 (internal code: M0271): rel. energy 1.34 kcal mol^{-1} . . . S61
6.3.12 Conformation 12 (internal code: M0311): rel. energy 0.42 kcal mol^{-1} . . . S62
6.4 Dihydroraputindole D (**3**) S63
6.4.1 Conformation 1 (internal code: M0005): rel. energy 2.00 kcal mol^{-1} S63
6.4.2 Conformation 2 (internal code: M0008): rel. energy 1.52 kcal mol^{-1} S64
6.4.3 Conformation 3 (internal code: M0012): rel. energy 1.16 kcal mol^{-1} S65
6.4.4 Conformation 4 (internal code: M0069): rel. energy 0.33 kcal mol^{-1} S66
6.4.5 Conformation 5 (internal code: M0078): rel. energy 0.00 kcal mol^{-1} S67
6.4.6 Conformation 6 (internal code: M0154): rel. energy 1.97 kcal mol^{-1} S68
6.4.7 Conformation 7 (internal code: M0200): rel. energy 2.01 kcal mol^{-1} S69
6.5 Raputindole D (**2**) S70
6.5.1 Conformation 1 (internal code: M0001): rel. energy 0.33 kcal mol^{-1} S70
6.5.2 Conformation 2 (internal code: M0002): rel. energy 0.00 kcal mol^{-1} S71
6.5.3 Conformation 3 (internal code: M0006): rel. energy 1.67 kcal mol^{-1} S72
6.5.4 Conformation 4 (internal code: M0007): rel. energy 0.51 kcal mol^{-1} S73
6.5.5 Conformation 5 (internal code: M0008): rel. energy 0.15 kcal mol^{-1} S74
6.5.6 Conformation 6 (internal code: M0010): rel. energy 1.75 kcal mol^{-1} S75
6.5.7 Conformation 7 (internal code: M0014): rel. energy 1.29 kcal mol^{-1} S76
6.5.8 Conformation 8 (internal code: M0015): rel. energy 1.11 kcal mol^{-1} S77
6.5.9 Conformation 9 (internal code: M0022): rel. energy 1.38 kcal mol^{-1} S78
6.5.10 Conformation 10 (internal code: M0023): rel. energy 1.44 kcal mol^{-1} . . . S79
6.5.11 Conformation 11 (internal code: M0037): rel. energy 1.71 kcal mol^{-1} . . . S80
6.5.12 Conformation 12 (internal code: M0039): rel. energy 1.59 kcal mol^{-1} . . . S81
6.5.13 Conformation 13 (internal code: M0042): rel. energy 1.52 kcal mol^{-1} . . . S82
6.5.14 Conformation 14 (internal code: M0045): rel. energy 1.19 kcal mol^{-1} . . . S83
6.5.15 Conformation 15 (internal code: M0048): rel. energy 0.90 kcal mol^{-1} . . . S84

1.1 General Methods

All reactions utilizing dry solvents were performed in Schlenk flasks fitted with rubber septa under a positive pressure of argon, unless otherwise noted. Air- and moisture-sensitive liquids were transferred by syringe. All reactions in dry solvents were carried out under argon. Analytical thin-layer chromatography (TLC) was performed using aluminum plates pre-coated with silica gel (silica gel 60 F_{254}, Merck). TLC plates were visualized by exposure to ultraviolet light (λ = 254 nm) and then were stained by submersion in a vanillin solution (6.8 g vanillin dissolved in 200 mL EtOH and 2.5 mL H_2SO_4) followed by a brief heating. Concentration under reduced pressure was performed by rotary evaporation at 50 °C, unless otherwise noted. Flash column chromatography was performed on Merck silica gel 60 (40 – 63 µm).

1.2 Materials

Chemicals were purchased from commercial suppliers and used without further purification. Solvents were dried prior to use by using standard methods, unless otherwise noted.

1.3 Instrumentation

NMR spectra were recorded with a Bruker AVIIIHD300N (300 MHz for ^{1}H, 75 MHz for ^{13}C), a Bruker AV III 400 (400 MHz for ^{1}H, 100 MHz for ^{13}C), a Bruker AVIIIHD500 (500 MHz for ^{1}H, 125 MHz for ^{13}C) or a Bruker AV-II 600 (600 MHz for ^{1}H, 150 MHz for ^{13}C) spectrometer at 299 K. Chemical shifts are given in ppm (δ scale) and referenced to tetramethylsilane (for ^{1}H = 0.00 ppm) or the residual solvent peak (for ^{13}C, $CDCl_3$ = 77.16 ppm). The signals were assigned by ^{1}H,^{13}C-HSQC-, ^{1}H,^{13}C-HMBC-, ^{1}H,^{1}HCOSY-, and ^{1}H,^{1}H-NOESY-experiments. The multiplicities are given as s (singlet), d (doublet), t (triplet), q (quartet), quin (quintet), sext (sextet), sept (septet), m (multiplet) or a combination thereof. Mass spectra were obtained with a ThermoFinnigan MAT95XL or a ThermoFisher Scientific (LTQ-Orbitrap Velos) spectrometer. IR spectra were recorded with a Bruker Tensor 27 or Bruker Alpha-P spectrometer using diamond ATR technique. UV/Vis spectra were measured with a Varian Cary 100 Bio UV/Vis spectrometer. Optical rotations were recorded on Anton Paar MCP150 polarimeter at 589 nm. Chiral phase HPLC analysis was conducted with a Merck Hitachi L-6200 Intelligent Pump and L-4200 UV-Vis detector (detection at 250 nm or 270 nm) on a phenomenex Lux Cellulose 1 column (150x4.6 mm, 5 µm, 1 mL min^{-1} flow) with HPLC-grade *n*-hexane/EtOH (85:15) or on a phenomenex Lux Amylose 2 column (150x4.6 mm, 5 µm, 1 mL min^{-1} flow) with HPLC-grade *n*-hexane/EtOH (75:25).

1.4 Computional Methods

A conformational analysis was performed by Spartan18[1] employing MMFF with Monte-Carlo searching. All possible conformers were searched, keeping only the structures in an energy range of 5 kcal mol^{-1} or 10 kcal mol^{-1} with respect to the most stable one. The minimum energy conformers found by MMFF were then optimized by Gaussian09 program package[2] using DFT calculations at the B3LYP/6-31G(d) level. The conformers within an energy range of 5 kcal mol^{-1} with respect to the most stable one were optimized again using DFT calculations at the ωB97XD/TApr-cc-pVDZ level with acetonitrile or methanol as the solvent (PCM). All conformers are real minima, no imaginary vibrational frequencies were found, and the zero point energy values were used to calculate the Boltzmann population of all unique conformers at 298.15 K. The conformers which are at least 1% populated were submitted to TDDFT calculations at the ωB97XD/TApr-cc-pVDZ level with acetonitrile or methanol as the solvent (PCM) to obtain their UV and ECD spectra. The ECD spectra were obtained from calculated excitation energies and rotational strength (velocity), as a sum of Gaussian functions centered at the wavelength of each transition, with a parameter σ (width of the band at half height) of 0.25 eV using SpecDis v.1.71 program[3]. Average UV and ECD spectra were obtained weighing each conformer according to its Boltzmann population. The obtained ECD sepctra were then UV-corrected to give the final ECD spectra.

To calculate the optical rotation, the same conformers were submitted to DFT calculations at the ωB97XD/TApr-cc-pVDZ level with acetonitrile or methanol as the solvent (PCM). The average optical rotation were obtained weighting each conformer according to its Boltzmann population.

[1] Spartan18, Y. Shao, L.F. Molnar, Y. Jung, J. Kussmann, C. Ochsenfeld, S.T. Brown, A.T.B. Gilbert, L.V. Slipchenko, S.V. Levchenko, D.P. O'Neill, R.A. DiStasio Jr., R.C. Lochan, T. Wang, G.J.O. Beran, N.A. Besley, J.M. Herbert, C.Y. Lin, T. Van Voorhis, S.H. Chien, A. Sodt, R.P. Steele, V.A. Rassolov, P.E. Maslen, P.P. Korambath, R.D. Adamson, B. Austin, J. Baker, E.F.C. Byrd, H. Dachsel, R.J. Doerksen, A. Dreuw, B.D. Dunietz, A.D. Dutoi, T.R. Furlani, S.R. Gwaltney, A. Heyden, S. Hirata, C-P. Hsu, G. Kedziora, R.Z. Khalliulin, P. Klunzinger, A.M. Lee, M.S. Lee, W.Z. Liang, I. Lotan, N. Nair, B. Peters, E.I. Proynov, P.A. Pieniazek, Y.M. Rhee, J. Ritchie, E. Rosta, C.D. Sherrill, A.C. Simmonett, J.E. Subotnik, H.L. Woodcock III, W. Zhang, A.T. Bell, A.K. Chakraborty, D.M. Chipman, F.J. Keil, A.Warshel, W.J. Hehre, H.F. Schaefer, J. Kong, A.I. Krylov, P.M.W. Gill and M. Head-Gordon, *Phys. Chem. Chem. Phys.* **2006**, *8*, 3172–3191.

[2] Gaussian 09, Revision D.01, M. J. Frisch, G. W. Trucks, H. B. Schlegel, G. E. Scuseria, M. A. Robb, J. R. Cheeseman, G. Scalmani, V. Barone, B. Mennucci, G. A. Petersson, H. Nakatsuji, M. Caricato, X. Li, H. P. Hratchian, A. F. Izmaylov, J. Bloino, G. Zheng, J. L. Sonnenberg, M. Hada, M. Ehara, K. Toyota, R. Fukuda, J. Hasegawa, M. Ishida, T. Nakajima, Y. Honda, O. Kitao, H. Nakai, T. Vreven, J. A. Montgomery, Jr., J. E. Peralta, F. Ogliaro, M. Bearpark, J. J. Heyd, E. Brothers, K. N. Kudin, V. N. Staroverov, T. Keith, R. Kobayashi, J. Normand, K. Raghavachari, A. Rendell, J. C. Burant, S. S. Iyengar, J. Tomasi, M. Cossi, N. Rega, J. M. Millam, M. Klene, J. E. Knox, J. B. Cross, V. Bakken, C. Adamo, J. Jaramillo, R. Gomperts, R. E. Stratmann, O. Yazyev, A. J. Austin, R. Cammi, C. Pomelli, J. W. Ochterski, R. L. Martin, K. Morokuma, V. G. Zakrzewski, G. A. Voth, P. Salvador, J. J. Dannenberg, S. Dapprich, A. D. Daniels, O. Farkas, J. B. Foresman, J. V. Ortiz, J. Cioslowski, D. J. Fox, Gaussian, Inc., Wallingford CT, 2013.

[3] a) T. Bruhn, A. Schaumlöffel, Y. Hemberger, G. Pescitelli, *SpecDis version 1.71*, Berlin, Germany, **2017**, `http://specdis-software.jimdo.com`. b) T. Bruhn, A. Schaumlöffel, Y. Hemberger, G. Bringmann, *Chirality* **2013**, *25*, 243–249.

2.1 6-Ethynyl-3,3,9,9-tetraisopropyl-2,10-dimethyl-4,8-dioxa-3,9-disilaundecan-6-ol (7)

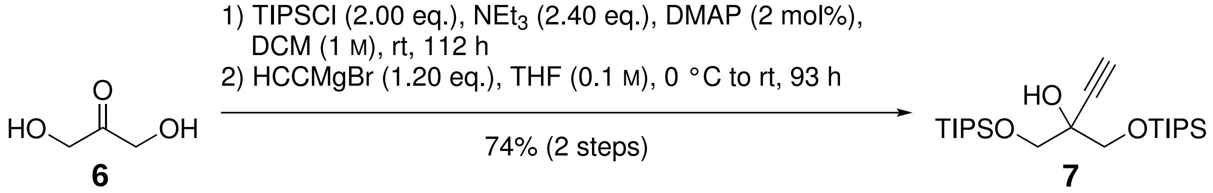

To a solution of dihydroxyacetone (**6**, 3.75 g, 41.7 mmol, 1.00 eq.), DMAP (102 mg, 0.833 mmol, 0.02 eq.) and dry NEt_3 (13.9 mL, 100 mmol, 2.40 eq.) in dry DCM (42 mL) was added TIPSCl (18.2 mL, 83.3 mmol, 2.00 eq.). The suspension was stirred at rt for 112 h and turned turbid over time. The reaction was quenched with sat. NH_4Cl (50 mL) and the aqueous phase was extracted with TBME (3x50 mL). The combined organic phases were washed with sat. NaCl (50 mL), dried over $MgSO_4$, filtered, and concentrated.
To a solution of the crude product in dry THF (110 mL) was added dropwise HCCMgBr (0.5 M in THF, 100 mL, 50.0 mmol, 1.20 eq.) at 0 °C. The solution was stirred at rt for 93 h. The reaction was quenched with sat. NH_4Cl (75 mL) and the aqueous phase was extracted with TBME (3x75 mL). The combined organic phases were washed with sat. NaCl (75 mL), dried over $MgSO_4$, filtered, and concentrated. Flash column chromatography on silica gel [petroleum ether/EtOAc (50:1) to (30:1)] afforded the product **7** as a yellow oil (13.3 g, 30.9 mmol, 74% over 2 steps, from **6**).
TLC [petroleum ether/DCM (1:1)]: R_f = 0.51 [vanillin: blue, not visible under UV-light].
^{1}H NMR (300 MHz, $CDCl_3$): δ [ppm] = 3.88 (d, J = 9.3 Hz, 2 H, TIPSO-CH_2), 3.79 (d, J = 9.3 Hz, 2 H, TIPSO-CH_2), 3.07 (s, 1 H, C-O*H*), 2.36 (s, 1 H, C≡C*H*), 1.17 – 0.97 (m, 42 H, C*H*(CH_3)$_2$, CH(CH_3)$_2$).
^{13}C NMR (76 MHz, $CDCl_3$): δ [ppm] = 84.4 (1 C, *C*-OH), 72.9 (1 C, C≡*C*H), 71.4 (1 C, *C*≡CH), 66.4 (2 C, TIPSO-*C*H_2), 18.1 (12 C, CH(*C*H_3)$_2$), 12.1 (6 C, *C*H(CH_3)$_2$).
IR (diamond ATR): $\tilde{\nu}$ [cm^{-1}] = 3313 (w), 2941 (m), 2894 (m), 2866 (m), 1463 (m), 1385 (w), 1329 (w), 1292 (w), 1251 (w), 1096 (m), 1064 (m), 995 (m), 953 (w), 917 (w), 881 (m), 800 (m), 755 (w), 681 (s), 652 (s), 548 (w).
UV/Vis (THF): λ_{max} (lg ε) = 278 (4.08).
HRESIMS: calculated [$C_{23}H_{48}O_3Si_2+Na^+$]: 451.30342
found: 451.30391 (1.09 ppm)

2.2 3,3,9,9-Tetraisopropyl-2,10-dimethyl-6-((1-(triisopropylsilyl)indolin-6-yl)-ethynyl)-4,8-dioxa-3,9-disilaundecan-6-yl acetate (9)

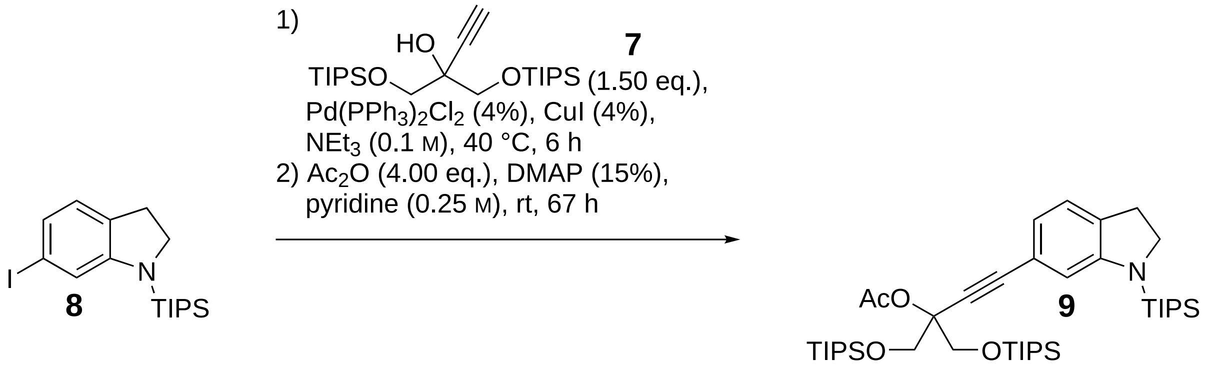

6-Iodo-1-(triisopropylsilyl)indoline (**8**, 4.01 g, 10.0 mmol, 1.00 eq.) and [Pd(PPh_3)$_2$$Cl_2$] (281 mg, 0.400 mmol, 0.04 eq.) were dissolved in dry, degassed (3x10 min sonication under reduced pressure/backfilling with argon) NEt_3 (90 mL). Separately, CuI (76 mg, 0.400 mmol, 0.04 eq.) and alkyne **7** (4.50 g, 10.5 mmol, 1.05 eq.) were dissolved in dry, degassed (3x10 min sonication) NEt_3 (10 mL) under argon. Both mixtures were degassed (3x10 min sonication) and combined. At 40 °C the second portion of alkyne **7** (1.93 g, 4.50 mmol, 0.45 eq.) was added and the mixture stirred for 6 h at this temperature. The color of the mixture changed from yellow to brown. The suspension was filtered through Celite 545, rinsed with TBME (150 mL), and concentrated.
To a solution of the crude product and DMAP (183 mg, 1.50 mmol, 0.15 eq.) in pyridine (40 mL) was added Ac_2O (3.8 mL, 40.0 mmol, 4.00 eq.). The solution was stirred for 67 h at rt. The reaction was cooled to 0 °C, quenched with sat. $NaHCO_3$ (200 mL), diluted with H_2O, and extracted with TBME (3x100 mL). The combined organic phases were washed with sat. NaCl (2x100 mL), dried over $MgSO_4$, filtered, and concentrated. Flash column chromatography on silica gel [petroleum ether/DCM (3:1) + 1% NEt_3] afforded crude product **9** that was used in the subsequent step without further purification.
An analytical pure sample was prepared by switching the equivalents of 6-iodo-1-(triisopropyl-silyl)indoline (**8**) (4.50 mmol) and alkyne **7** (3.00 mmol). The product **9** was obtained as yellow oil (900 mg, 1.21 mmol, 40% over 2 steps, from **8**).
TLC [petroleum ether/DCM (1:1)]: R_f = 0.66 [vanillin: green].
^{1}H NMR (400 MHz, $CDCl_3$): δ [ppm] = 6.94 (d, J = 7.5 Hz, 1 H, 4-*H*), 6.72 (dd, J = 7.4 Hz, J = 1.2 Hz, 1 H, 5-*H*), 6.59 (d, J = 0.9 Hz, 1 H, 7-*H*), 4.23 (d, J = 9.3 Hz, 2 H, TIPSO-CH_2), 4.08 (d, J = 9.5 Hz, 2 H, TIPSO-CH_2), 3.69 (t, J = 8.7 Hz, 1 H, 2-*H*), 2.94 (t, J = 8.5 Hz, 1 H, 3-*H*), 2.03 (s, 3 H, O-C(=O)-CH_3), 1.42 (sept, J = 7.5 Hz, 3 H, N-Si-(C*H*-(CH_2)$_2$)$_3$), 1.12 (d, J = 7.3 Hz, 18 H, N-Si-(CH-(CH_2)$_2$)$_3$), 1.10 – 1.05 (m, 42 H, O-Si-(C*H*-(CH_3)$_2$)$_3$, O-Si-(CH-(CH_3)$_2$)$_3$).
^{13}C NMR (101 MHz, $CDCl_3$): δ [ppm] = 169.1 (1 C, O-*C*(=O)-CH_3), 152.7 (1 C, *C*-7a), 133.0 (1 C, *C*-3a), 123.9 (1 C, *C*-4), 122.1 (1 C, *C*-5), 121.2 (1 C, *C*-6), 112.6 (1 C, *C*-7), 88.5 (1 C, C-6-*C*≡C), 84.2 (1 C, C-6-C≡*C*), 78.9 (1 C, *C*-OAc), 63.5 (2 C, TIPSO-*C*H_2), 50.8 (1 C, *C*-2), 30.5 (1 C, *C*-3), 21.9 (1 C, O-C(=O)-*C*H_3), 18.6 (6 C, N-Si-(CH-(*C*H_3)$_2$)$_3$), 18.1 (12 C, O-Si-(CH-(*C*H_3)$_2$)$_3$), 13.0 (3 C, N-Si-(*C*H-(CH_3)$_2$)$_3$), 12.2 (6 C, O-Si-(*C*H-(CH_3)$_2$)$_3$).
IR (diamond ATR): $\tilde{\nu}$ [cm^{-1}] = 2941 (m), 2865 (m), 1745 (m), 1598 (w), 1488 (m), 1463 (m), 1420 (w), 1365 (w), 1328 (w), 1257 (m), 1234 (m), 1212 (m), 1176 (w), 1112 (m), 1069 (m), 992 (m), 964 (w), 922 (w), 882 (m), 800 (m), 681 (s), 639 (m), 600 (m), 568 (w).
UV/Vis (THF): λ_{max} (lg ε) = 334 (3.58), 276 (4.12), 244 (4.52).

HRESIMS: calculated [$C_{42}H_{77}NO_4Si_3+Na^+$]: 766.50526
found: 766.50474 (0.68 ppm)

2.3 1-(Triisopropylsilyl)-5,5-bis(((triisopropylsilyl)oxy)methyl)-2,3,5,6-tetrahydro-cyclopenta[*f*]indol-7(1*H*)-one (10)

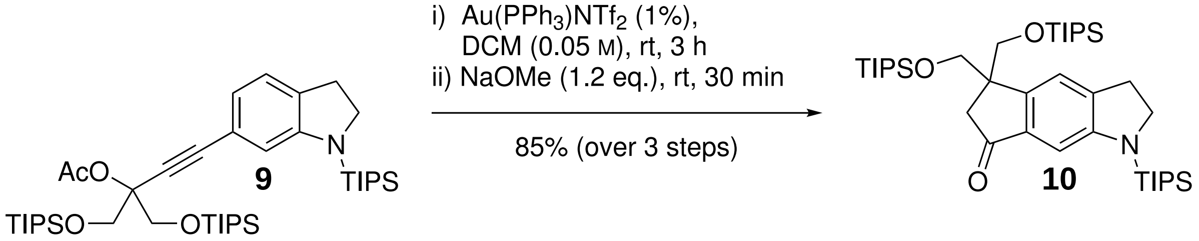

To a solution of propargylic acetate **9** (mixture obtained from the previous reaction, 8.79 g) in dry DCM (200 mL) was added $Au(PPh_3)NTf_2$ (74 mg, 0.100 mmol, 0.01 eq.). The solution was stirred at rt for 3 h. NaOMe (30 wt–% in MeOH, 2.2 mL, 12.0 mmol, 1.20 eq.) was added and the mixture stirred at rt for 30 min. The reaction was quenched with sat. NH_4Cl (100 mL) and extracted with DCM (3x100 mL). The combined organic phases were dried over $MgSO_4$, filtered, and concentrated. Flash column chromatography on silica gel [petroleum ether/DCM (3:1) + 1% NEt_3] afforded the crude product **10** that was used in the subsequent step without further purification.

An analytical pure sample was obtained after repeated flash column chromatography as a yellowish solid (5.99 g, 8.53 mmol, 85% over 3 steps, from **8**).

TLC [petroleum ether/DCM (1:1)]: R_f = 0.38 [vanillin: yellow, fluorescent].

^{1}H NMR (400 MHz, $CDCl_3$): δ [ppm] = 7.25 (s, 1 H, 4-*H*), 6.80 (s, 1 H, 8-*H*), 3.91 (d, J = 9.2 Hz, 2 H, TIPSO-CH_2), 3.87 (d, J = 9.2 Hz, 2 H, TIPSO-CH_2), 3.74 (t, J = 8.5 Hz, 2 H, 2-*H*), 2.98 (t, J = 8.4 Hz, 2 H, 3-*H*), 2.56 (s, 2 H, 6-*H*), 1.47 (sept, J = 7.5 Hz, 3 H, N-Si-(C*H*-($CH_3)_2)_3$), 1.11 (d, J = 7.5 Hz, 18 H, N-Si-(CH-(C$H_3)_2)_3$), 1.07 – 0.95 (m, 42 H, O-Si-(C*H*-($CH_3)_2)_3$, O-Si-(CH-(C$H_3)_2)_3$).

^{13}C NMR (101 MHz, $CDCl_3$): δ [ppm] = 205.6 (1 C, *C*-7), 153.5 (1 C, *C*-8a), 148.6 (1 C, *C*-4a), 141.2 (1 C, *C*-3a), 137.7 (1 C, *C*-7a), 121.3 (1 C, *C*-4), 102.1 (1 C, *C*-8), 67.3 (2 C, TIPSO-*C*H_2), 51.1 (1 C, *C*-2), 49.6 (1 C, *C*-5), 45.1 (1 C, *C*-6), 30.8 (1 C, *C*-3), 18.6 (6 C, N-Si-(CH-(*C*$H_3)_2)_3$), 18.1 (12 C, O-Si-(CH-(*C*$H_3)_2)_3$), 12.9 (3 C, N-Si-(*C*H-($CH_3)_2)_3$), 12.1 (6 C, O-Si-(*C*H-($CH_3)_2)_3$).

IR (diamond ATR): $\tilde{\nu}$ [cm^{-1}] = 2942 (m), 2893 (m), 2863 (m), 1701 (m), 1614 (w), 1464 (m), 1370 (m), 1301 (m), 1253 (m), 1173 (w), 1097 (s), 1059 (s), 992 (m), 946 (w), 920 (w), 880 (s), 847 (m), 810 (s), 743 (w), 681 (s), 643 (s), 573 (m).

UV/Vis (THF): λ_{max} (lg ε) = 368 (3.47), 277 (4.52), 247 (4.56).

Fluorescence (THF): λ_{ex} = 365 nm, λ_{em} = 429 nm.

HRESIMS: calculated [$C_{40}H_{75}NO_3Si_3+Na^+$]: 724.49470
found: 724.49450 (0.28 ppm)

2.4 5,5-Bis(hydroxymethyl)-1-tosyl-1,2,3,5-tetrahydrocyclopenta[*f*]indol-7-yl trifluormethansulfonate (11)

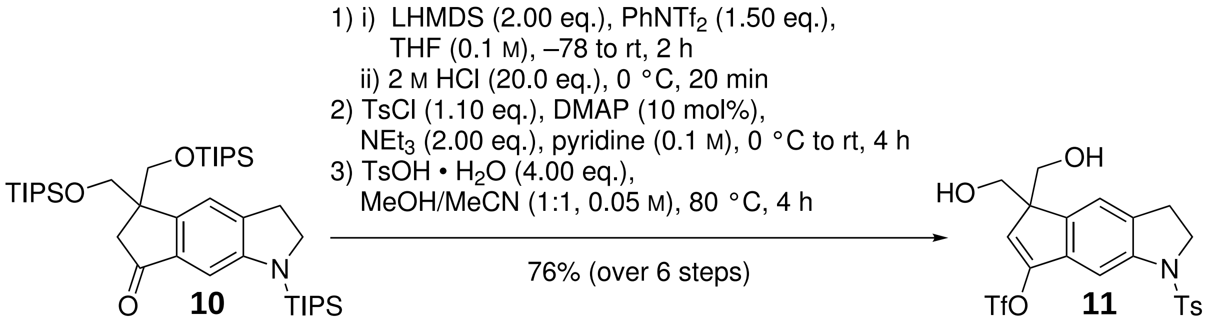

LHMDS (1 M in THF/ethylbenzene, 20 mL, 20.0 mmol, 2.00 eq.) was added dropwise to a solution of ketone **10** (mixture obtained from the previous reaction, 7.96 g) and $PhNTf_2$ (5.41 g, 15.0 mmol, 1.50 eq.) in dry THF (100 mL) at –78 °C. The solution was stirred at –78 °C for 20 min and at rt for 90 min. To the solution was added 2 M HCl (100 mL, 200 mmol, 20.0 eq.) at 0 °C. After stirring for 20 min at rt, 2 M NaOH (200 mL, 400 mmol, 40.0 eq.) was added at 0 °C and the solution was extracted with TBME (3x200 mL). The combined organic phases were washed with sat. $NaHCO_3$ (200 mL) and sat. NaCl (200 mL), dried over $MgSO_4$, filtered, and concentrated.
TsCl (2.14 g, 11.0 mmol, 1.10 eq.) was added to a solution of the obtained brown oil, DMAP (122 mg, 1 mmol, 0.1 eq.) and NEt_3 (2.8 mL, 20 mmol, 2.00 eq.) in pyridine (100 mL) at 0 °C. The solution was stirred at rt for 4 h The reaction was quenched with 2 M HCl (100 mL) and extracted with DCM (3x200 mL). The combined organic phases were dried over $MgSO_4$, filtered, and concentrated. The deep red residue was filtered trough silica gel (approx. 5 cm), rinsed with DCM (200 mL) and concentrated.
The obtained brown oil and $TsOH \cdot H_2O$ (7.61 g, 40.0 mmol, 4.00 eq.) were dissolved in MeOH (100 mL) and MeCN (100 mL). The solution was stirred at 80 °C for 4 h, while it turned to a darker shade of brown. The reaction was quenched with sat. $NaHCO_3$ (200 mL) and extracted with EtOAc (3x200 mL). The combined organic phases were washed with sat. $NaHCO_3$ (200 mL) and sat. NaCl (200 mL), dried over $MgSO_4$, filtered, and concentrated. Flash column chromatography on silica gel [petroleum ether/EtOAc (1:2) to (1:4)] afforded the product **11** as a colorless foam (3.96 g, 7.62 mmol, 76% over 6 steps, from **8**).
TLC [petroleum ether/EtOAc (1:2)]: R_f = 0.13 [vanillin: brown].
^{1}H NMR (400 MHz, $CDCl_3$): δ [ppm] = 7.73 – 7.69 (m, 2 H, *o*-H_{Ts}), 7.60 (s, 1 H, 8-*H*), 7.28 – 7.23 (m, 3 H, 4-*H*, *m*-H_{Ts}), 6.40 (s, 1 H, 6-*H*), 3.95 (t, *J* = 8.5 Hz, 2 H, 2-*H*), 3.88 (d, *J* = 10.7 Hz, 2 H, HO-CH_2), 3.79 (d, *J* = 10.7 Hz, 2 H, HO-CH_2), 2.97 (t, *J* = 8.5 Hz, 2 H, 3-*H*), 2.38 (s, 3 H, *p*-C_{Ts}-CH_3).
^{13}C NMR (101 MHz, $CDCl_3$): δ [ppm] = 148.5 (1 C, *C*-7), 144.5 (1 C, *p*-C_{Ts}), 142.6 (1 C, *C*-8a), 139.5 (1 C, *C*-4a), 136.1 (1 C, *C*-7a), 133.8 (1 C, *ipso*-C_{Ts}), 131.7 (1 C, *C*-3a), 129.9 (2 C, *m*-C_{Ts}), 127.5 (2 C, *o*-C_{Ts}), 122.9 (1 C, *C*-6), 121.1 (1 C, *C*-4), 120.5/117.3 (1 C, *C*F_3), 105.4 (1 C, *C*-8), 65.4 (2 C, HO-*C*H_2), 56.9 (1 C, *C*-5), 50.5 (1 C, *C*-2), 27.9 (1 C, *C*-3), 21.7 (2 C, *p*-C_{Ts}-*C*H_3).
^{19}F NMR (377 MHz, $CDCl_3$): δ [ppm] = –73.16 (CF_3).
IR (diamond ATR): $\tilde{\nu}$ [cm^{-1}] = 3310 (w), 3021 (w), 2928 (w), 2878 (w), 1601 (w), 1573 (w), 1477 (w), 1450 (m), 1426 (m), 1340 (m), 1244 (m), 1214 (s), 1159 (s), 1134 (s), 1069 (s), 1025 (s), 935 (m), 893 (m), 840 (m), 810 (m), 752 (s), 707 (m), 661 (s), 587 (s), 543 (m).

UV/Vis (THF): λ_{max} (lg ε) = 312 (3.56), 277 (4.20), 234 (4.45).
HRESIMS: calculated [$C_{21}H_{20}F_3NO_7S_2$+Na^+]: 542.05255
found: 542.05273 (0.33 ppm)

2.5 (7-(2-Methylprop-1-en-1-yl)-1-tosyl-1,2,3,5-tetrahydrocyclopenta[*f*]indole-5,5-diyl)dimethanol (14)

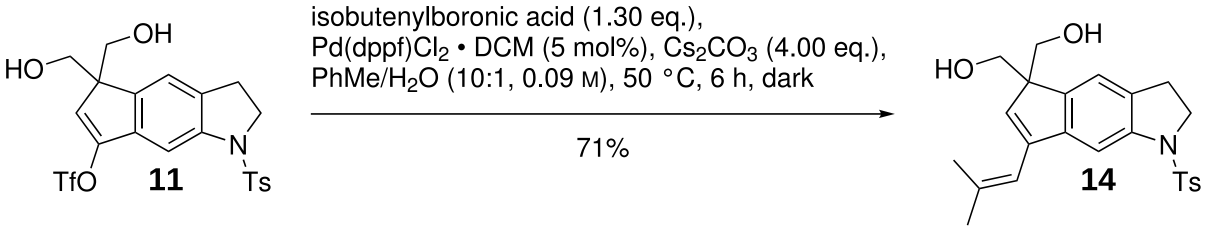

Alkenyl triflate **11** (2.60 g, 5.00 mmol, 1.00 eq.), isobutenylboronic acid (649 mg, 6.50 mmol, 1.30 eq.), Pd(dppf)Cl_2 · DCM (208 mg, 0.250 mmol, 0.05 eq.) und Cs_2CO_3 (6.52 g, 20.0 mmol, 4.00 eq.) were dissolved in PhMe (50 mL) and H_2O (5 mL). The red mixture was degassed by sonication and subsequently heated to 50 °C for 6 h in the dark, while it turned brown. After cooling to rt, the mixture was filtered through Celite 545, rinsed with EtOAc (150 mL) and concentrated. Purification of the residue by flash chromatography on silica gel [petroleum ether/EtOAc (1:2)] afforded the product **14** as a colorless foam (1.51 g, 3.54 mmol, 71%).
TLC [petroleum ether/EtOAc (1:2)]: R_f = 0.28 [vanillin: blue].
^{1}H NMR (400 MHz, $CDCl_3$): δ [ppm] = 7.70 – 7.64 (m, 2 H, *o*-H_{Ts}), 7.62 (s, 1 H, 8-*H*), 7.24 – 7.20 (m, 2 H, *m*-H_{Ts}), 7.19 (s, 1 H, 4-*H*), 6.27 (s, 1 H, 6-*H*), 6.21 – 6.14 (m, 1 H, C-7-C*H*=C(CH_3)$_2$), 3.92 (t, *J* = 8.4 Hz, 2 H, 2-*H*), 3.83 (s, 4 H, HO-CH_2), 2.87 (t, *J* = 8.3 Hz, 2 H, 3-*H*), 2.36 (s, 3 H, *p*-C_{Ts}-CH_3), 2.02 – 1.98 (m, 3 H, C-7-CH=C(CH_3)$_2$ (*cis* to *H*)), 1.94 – 1.90 (m, 3 H, C-7-CH=C(CH_3)$_2$ (*trans* to *H*)).
^{13}C NMR (101 MHz, $CDCl_3$): δ [ppm] = 145.6 (1 C, *C*-7a), 144.2 (1 C, *p*-C_{Ts}), 142.1 (1 C, *C*-8a), 142.0 (1 C, *C*-4a), 141.5 (1 C, *C*-7), 140.4 (1 C, C-7-CH=*C*(CH_3)$_2$), 134.3 (1 C, *ipso*-C_{Ts}), 133.6 (1 C, *C*-6), 129.8 (2 C, *m*-C_{Ts}), 129.5 (1 C, *C*-3a), 127.5 (2 C, *o*-C_{Ts}), 120.2 (1 C, *C*-4), 116.6 (1 C, C-7-*C*H=C(CH_3)$_2$), 107.5 (1 C, *C*-8), 66.6 (2 C, HO-*C*H_2), 59.0 (1 C, *C*-5), 50.6 (1 C, *C*-2), 28.0 (1 C, *C*-3), 27.2 (1 C, C-7-CH=C(*C*H_3)$_2$ (*cis* to *H*)), 21.7 (1 C, *p*-C_{Ts}-*C*H_3), 20.9 (1 C, C-7-CH=C(*C*H_3)$_2$ (*trans* to *H*)).
IR (diamond ATR): $\tilde{\nu}$ [cm^{-1}] = 3382 (w), 2922 (w), 2873 (w), 1650 (w), 1602 (w), 1565 (w), 1469 (w), 1443 (w), 1347 (m), 1258 (w), 1160 (m), 1089 (m), 1021 (m), 940 (w), 880 (w), 854 (w), 810 (m), 744 (w), 702 (w), 661 (m), 590 (m), 544 (m).
UV/Vis (MeOH): λ_{max} (lg ε) = 311 (3.69), 264 (4.06), 229 (4.51).
HRESIMS: calculated [$C_{24}H_{27}NO_4S$+Na^+]: 448.15530
found: 448.15572 (0.94 ppm)

2.6 (*S*)-(5-(Hydroxymethyl)-1-tosyl-7-(((trifluoromethyl)sulfonyl)oxy)-1,2,3,5-tetrahydrocyclopenta[*f*]indol-5-yl)methyl benzoate (13)

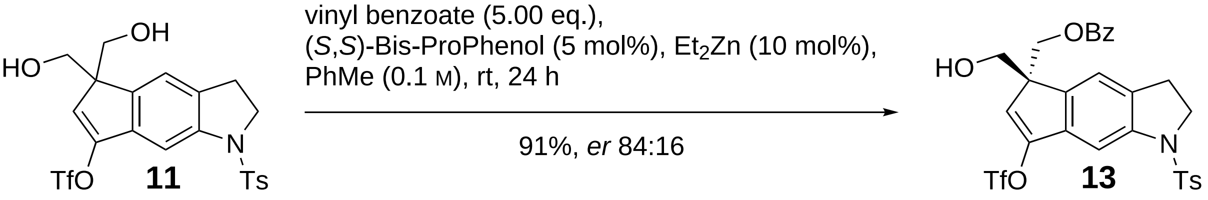

To a solution of ((2*S*,2'*S*)-((2-hydroxy-5-methyl-1,3-phenylene)bis(methylene))bis(pyrrolidine-1,2-diyl))bis(diphenylmethanol) (**12**, 54 mg, 0.080 mmol, 0.05 eq.) in dry PhMe (3.2 mL) was added dropwise Et_2Zn (1 M in hexane, 0.16 mL, 0.160 mmol, 0.10 eq.). After stirring for 30 min at rt, the mixture was added to a solution of diol **11** (835 mg, 1.70 mmol, 1.00 eq.) and vinyl benzoate (1.1 mL, 8.04 mmol, 5.00 eq.) in dry PhMe (13 mL). The solution was stirred at rt for 22 h. The reaction was quenched with an aqueous NaH_2PO_4 solution (5 wt %, 20 mL) and extract with TBME (3x25 mL). The combined organic phases were washed with H_2O (25 mL) and sat. NaCl (25 mL), dried over $MgSO_4$, filtered, and concentrated. Purification of the residue by flash chromatography on silica gel [petroleum ether/EtOAc (2:1) to (1:2)] gave the product **13** as a colorless foam (910 mg, 1.45 mmol, 91% 68% *ee*).

TLC [petroleum ether/EtOAc (1:2)]: R_f = 0.71 [vanillin: brown].

$[\alpha]_D^{20}$ = +56.1° (*c* = 0.54, MeOH).

^{1}H NMR (500 MHz, $CDCl_3$): δ [ppm] = 8.05 – 8.01 (m, 2 H, *o*-H_{Ph}), 7.72 – 7.68 (m, 2 H, *o*-H_{Ts}), 7.65 (s, 1 H, 8-*H*), 7.64 – 7.60 (m, 1 H, *p*-H_{Ph}), 7.50 – 7.45 (m, 2 H, *m*-H_{Ph}), 7.31 (s, 1 H, 4-*H*), 7.25 – 7.20 (m, 2 H, *m*-H_{Ts}), 6.37 (s, 1 H, 6-*H*), 4.73 (d, *J* = 11.1 Hz, 1 H, BzO-CH_2), 4.34 (d, *J* = 11.5 Hz, 1 H, BzO-CH_2), 3.96 (t, *J* = 8.5 Hz, 2 H, 2-*H*), 3.76 (d, *J* = 11.5 Hz, 1 H, HO-CH_2), 3.57 (d, *J* = 11.5 Hz, 1 H, HO-CH_2), 2.96 (t, *J* = 8.5 Hz, 2 H, 3-*H*), 2.37 (s, 3 H, *p*-C_{Ts}-CH_3).

^{13}C NMR (126 MHz, $CDCl_3$): δ [ppm] = 167.3 (1 C, Ph-*C*(=O)O), 148.9 (1 C, *C*-7), 144.5 (1 C, *p*-C_{Ts}), 142.9 (1 C, *C*-8a), 139.1 (1 C, *C*-4a), 136.0 (1 C, *C*-7a), 133.8 (1 C, *p*-C_{Ph}), 133.7 (1 C, *ipso*-C_{Ts}), 132.1 (1 C, *C*-3a), 129.9 (2 C, *m*-C_{Ts}), 129.9 (2 C, *o*-C_{Ph}), 129.3 (1 C, *ipso*-C_{Ph}), 128.8 (2 C, *m*-C_{Ph}), 127.5 (2 C, *o*-C_{Ts}), 122.1 (1 C, 6-*C*), 121.5 (1 C, 4-*C*), 118.9 (q, *J* = 321.2 Hz, 1 C, *C*F_3), 105.5 (1 C, *C*-8), 64.7 (1 C, BzO-*C*H_2), 63.2 (1 C, HO-*C*H_2), 55.8 (1 C, *C*-5), 50.5 (1 C, *C*-2), 27.9 (1 C, *C*-3), 21.7 (1 C, *p*-C-Ts-CH_3).

^{19}F NMR (377 MHz, $CDCl_3$): δ [ppm] = –73.11 (CF_3).

IR (diamond ATR): $\tilde{\nu}$ [cm^{-1}] = 3524 (w), 2951 (w), 1720 (m), 1602 (w), 1577 (w), 1473 (w), 1425 (m), 1351 (m), 1271 (m), 1247 (m), 1213 (s), 1164 (s), 1138 (s), 1093 (m), 1068 (m), 1045 (m), 998 (m), 901 (w), 838 (m), 813 (m), 766 (w), 745 (m), 711 (m), 662 (m), 591 (s), 546 (m), 422 (w).

UV/Vis (MeOH): λ_{max} (lg ε) = 310 (3.59), 229 (4.59).

**HRESIMS**:	calculated [$C_{28}H_{24}F_3NO_8S_2$+Na^+]:	646.07876
	found:	646.07856 (0.31 ppm)

2.7 (*rac*)-(5-Hydroxymethyl)-1-tosyl-(((trifluoromethyl)sulfonyl)oxy)-1,2,3,5-tetrahydrocyclopenta[*f*]indol-5-yl)methyl benzoate ((*rac*)-13)

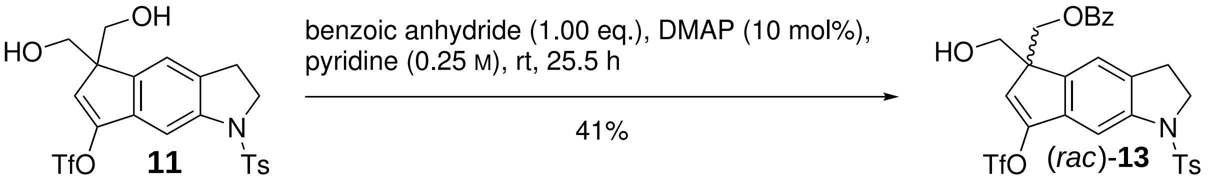

To a solution of diol **11** (547 mg, 1.05 mmol, 1.00 eq.) and DMAP (13 mg, 0.105 mmol, 0.10 eq.) in pyridine (4.2 mL) was added benzoic anhydride (251 mg, 1.05 mmol, 1.00 eq.). The solution was stirred at rt for 25.5 h. The reaction was quenched with sat. $NaHCO_3$ (25 mL), diluted with H_2O (25 mL), and extracted with EtOAc (3x50 mL). The combined organic phases were washed with sat. NaCl (2x50 mL), dried over $MgSO_4$, filtered, and concentrated. Purification of the residue by flash chromatography on silica gel [petroleum ether/EtOAc (4:1) to (2:1) to (0:1)] gave the product (*rac*)-**13** as a colorless foam (256 mg, 0.411 mmol, 41%).

2.8 Dimer 20a and dimer 20b

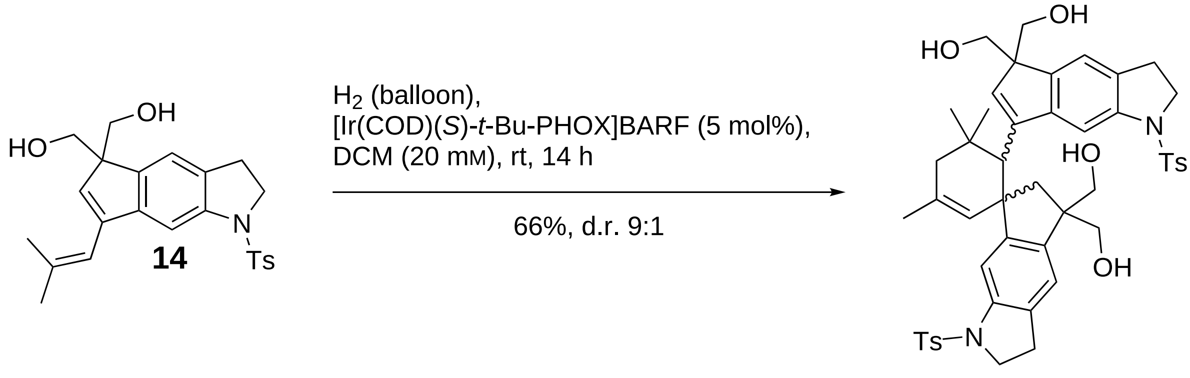

[Ir(COD)(*S*)-*t*-Bu-PHOX]BARF (2 mg, 0.001 mmol, 0.05 eq.) and diol **14** (10 mg, 0.023 mmol, 1.00 eq.) were dissolved in dry, degassed DCM (1 mL). The orange solution was purged with H_2, turned yellow and was stirred under H_2 (balloon) at rt for 14 h. This procedure was repeated seven times and all eight solutions were combined. The solvent was removed under reduced pressure and purification by flash chromatography on silica gel [petroleum ether/EtOAc (1:2) to (1:4) to (0:1)] afforded dimer **20a** (47 mg, 0.055 mmol, 59%) and dimer **20b** (6 mg, 0.007 mmol, 7%) as yellowish foams.

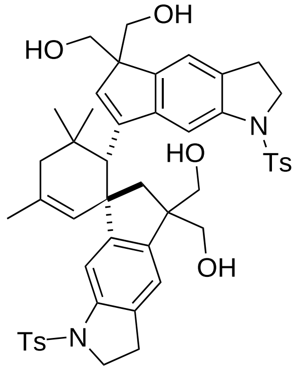

TLC [petroleum ether/EtOAc (1:4)]: R_f = 0.21 [vanillin: brown].
^{1}H NMR (400 MHz, $CDCl_3$, spiro moiety marked with *): δ [ppm] = 7.66 – 7.61 (m, 2 H, *o-H*$_{Ts*}$), 7.44 – 7.39 (m, 3 H, *o-H*$_{Ts}$, 8-*H*), 7.28 (s, 1 H, 8*-*H*), 7.27 – 7.23 (m, 2 H, *m-H*$_{Ts*}$), 7.11 – 7.07 (m, 2 H, *m-H*$_{Ts}$), 6.96 (s, 1 H, 4-*H*), 6.86 (s, 1 H, 4*-*H*), 6.42 (s, 1 H, 6-*H*), 5.25 (s, 1 H, C-7*-C*H*=C-CH_3), 4.16 – 3.53 (m, 10 H, 2-*H*, 2*-*H*, C-5-(CH_2OH)$_2$, C-5*-(CH_2OH)$_2$), 3.43 – 3.27 (m, 2 H, C-5-(CH_2OH)$_2$), 3.15 (s, 1 H, C-7-C*H*-C(CH_3)$_2$-CH_2), 2.95 (d, J = 14.0 Hz, 1 H, 6*-*H*), 2.89 – 2.60 (m, 4 H, 3-*H*, 3*-*H*), 2.47 (d, J = 17.5 Hz, 1 H, C-7-CH-C(CH_3)$_2$-CH_2), 2.39 (s, 3 H, *p*-C$_{Ts*}$-CH_3), 2.36 (s, 3 H, *p*-C$_{Ts}$-CH_3), 2.00 (d, J = 13.9 Hz, 1 H, 6*-*H*), 1.95 (s, 3 H, C-7*-C-CH=C-CH_3), 1.70 (d, J = 17.8 Hz, 1 H, C-7-CH-C(CH_3)$_2$-CH_2), 1.37 (s, 3 H, C_7-CH-C(CH_3)$_2$-CH_2), 0.80 (s, 3 H, C_7-CH-C(CH_3)$_2$-CH_2).
^{13}C NMR (101 MHz, $CDCl_3$, spiro moiety marked with *): δ [ppm] = 150.2 (1 C, *C*-7a*), 147.4 (1 C, *C*-7a), 144.8 (1 C, *p-C*$_{Ts*}$), 144.3 (1 C, *p-C*$_{Ts}$), 143.7 (1 C, *C*-7), 141.9 (1 C, *C*-4a*), 141.8 (1 C, *C*-4a), 141.4 (1 C, *C*-8a), 139.8 (1 C, *C*-8a*), 135.6 (1 C, *C*-6), 133.9 (1 C, *ipso-C*$_{Ts}$), 132.6 (1 C, C-7*-C-CH=*C*-(CH_3)), 132.5 (1 C, *ipso-C*$_{Ts*}$), 130.4 (1 C, C-7*-C-*C*H=C-(CH_3)), 130.2 (1 C, *C*-3a*), 129.8 (2 C, *m-C*$_{Ts*}$), 129.7 (2 C, *m-C*$_{Ts}$), 129.3 (1 C, *C*-3a), 127.8 (2 C, *o-C*$_{Ts*}$), 127.3 (2 C, *o-C*$_{Ts}$), 120.2 (1 C, *C*-4*), 119.6 (1 C, *C*-4), 116.0 (1 C, *C*-8*), 107.3 (1 C, *C*-8), 70.5 (1 C, C-5*-(*C*H_2OH)$_2$), 69.2 (1 C, C-5*-(*C*H_2OH)$_2$), 67.5 (1 C, C-5-(*C*H_2OH)$_2$), 66.7 (1 C, C-5-(*C*H_2OH)$_2$), 59.3 (1 C, *C*-5), 52.0 (1 C, *C*-5*), 51.6 (1 C, *C*-7*), 50.8 (2 C, *C*-2, *C*-2*), 49.5 (1 C, C-7-*C*H-C(CH_3)$_2$-CH_2), 47.1 (1 C, *C*-6*), 39.9 (1 C, C-7-CH-C(CH_3)$_2$-*C*H_2), 35.2 (1 C, C-7-CH-*C*(CH_3)$_2$-CH_2), 31.9 (1 C, C-7-CH-C(*C*H_3)$_2$-CH_2), 29.5 (1 C, C-7-CH-C(*C*H_3)$_2$-CH_2), 28.0 (1 C, *C*-3), 27.7 (1 C, *C*-3*), 24.6 (1 C, C-7*-C-CH=C-(*C*H_3)), 21.7 (2 C, *p*-C$_{Ts}$-*C*H_3, *p*-C$_{Ts*}$-*C*H_3).
IR (diamond ATR): $\tilde{\nu}$ [cm^{-1}] = 3483 (w), 2924 (m), 2924 (m), 2868 (w), 1602 (w), 1470 (m), 1439 (m), 1346 (m), 1277 (m), 1158 (s), 1127 (m), 1090 (m), 1020 (m), 938 (w), 883 (w), 810 (m), 751 (m), 706 (w), 661 (s), 589 (s), 544 (m).
UV/Vis (MeOH): λ_{max} (lg ε) = 301 (3.92), 272 (4.11), 211 (4.63).
HRESIMS: calculated [$C_{48}H_{54}N_2O_8S_2$+Na^+]: 873.32138
found: 873.32211 (0.84 ppm)

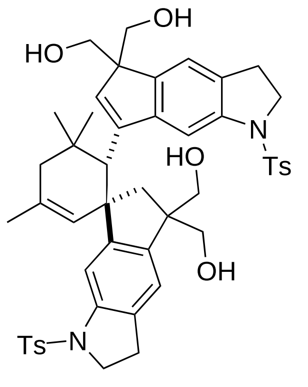

TLC [petroleum ether/EtOAc (1:4)]: R_f = 0.05 [vanillin: brown].
^{1}H NMR (500 MHz, $CDCl_3$, spiro moiety marked with *): δ [ppm] = 7.65 – 7.61 (m, 2 H, *o-H*$_{Ts*}$), 7.44 – 7.39 (m, 3 H, *o-H*$_{Ts}$, 8-*H*), 7.27 (s, 1 H, 8*-*H*), 7.26 – 7.23 (m, 2 H, *m-H*$_{Ts*}$), 7.11 – 7.07 (m, 2 H, *m-H*$_{Ts}$), 6.94 (s, 1 H, 4-*H*), 6.85 (s, 1 H, 4*-*H*), 6.44 (s, 1 H, 6-*H*), 5.24 (s, 1 H, C-7*-C*H*=C-CH_3), 4.16 – 3.56 (m, 10 H, 2-*H*, 2*-*H*, C-5-(CH_2OH)$_2$, C-5*-(CH_2OH)$_2$), 3.38 (d, J = 11.0 Hz, 2 H, C-5-(CH_2OH)$_2$), 3.14 (s, 1 H, C-7-C*H*-C(CH_3)$_2$-CH_2), 2.96 (d, J = 13.7 Hz, 1 H, 6*-*H*), 2.84 – 2.61 (m, 4 H, 3-*H*, 3*-*H*), 2.48 (d, J = 19.0 Hz, 1 H, C-7-CH-C(CH_3)$_2$-CH_2),

2.39 (s, 3 H, *p*-C_{Ts*}-C*H*$_3$), 2.35 (s, 3 H, *p*-C_{Ts}-C*H*$_3$), 1.99 (d, *J* = 14.0 Hz, 1 H, 6*-*H*), 1.95 (s, 3 H, C-7*-C-CH=C-C*H*$_3$), 1.69 (d, *J* = 17.9 Hz, 1 H, C-7-CH-C(CH_3)$_2$-C*H*$_2$), 1.37 (s, 3 H, C_7-CH-C(C*H*$_3$)$_2$-CH_2), 0.79 (s, 3 H, C_7-CH-C(C*H*$_3$)$_2$-CH_2).
^{13}C NMR (126 MHz, $CDCl_3$, spiro moiety marked with *): δ [ppm] = 150.2 (1 C, *C*-7a*), 147.4 (1 C, *C*-7a), 144.9 (1 C, *p*-*C*$_{Ts*}$), 144.4 (1 C, *p*-*C*$_{Ts}$), 143.7 (1 C, *C*-7), 141.8 (1 C, *C*-4a*), 141.7 (1 C, *C*-4a), 141.4 (1 C, *C*-8a), 139.8 (1 C, *C*-8a*), 135.6 (1 C, *C*-6), 133.8 (1 C, *ipso*-*C*$_{Ts}$), 132.7 (1 C, C-7*-C-CH=*C*-(CH_3)), 132.3 (1 C, *ipso*-*C*$_{Ts*}$), 130.3 (1 C, C-7*-C-*C*H=C-(CH_3)), 130.2 (1 C, *C*-3a*), 129.8 (2 C, *m*-*C*$_{Ts*}$), 129.7 (2 C, *m*-*C*$_{Ts}$), 129.3 (1 C, *C*-3a), 127.9 (2 C, *o*-*C*$_{Ts*}$), 127.3 (2 C, *o*-*C*$_{Ts}$), 120.2 (1 C, *C*-4*), 119.6 (1 C, *C*-4), 116.0 (1 C, *C*-8*), 107.2 (1 C, *C*-8), 70.6 (1 C, C-5*-(*C*H_2OH)$_2$), 69.4 (1 C, C-5*-(*C*H_2OH)$_2$), 67.7 (1 C, C-5-(*C*H_2OH)$_2$), 66.8 (1 C, C-5-(*C*H_2OH)$_2$), 59.3 (1 C, *C*-5), 52.0 (1 C, *C*-5*), 51.6 (1 C, *C*-7*), 50.8 (2 C, *C*-2, *C*-2*), 49.4 (1 C, C-7-*C*H-C(CH_3)$_2$-CH_2), 47.1 (1 C, *C*-6*), 39.8 (1 C, C-7-CH-C(CH_3)$_2$-*C*H_2), 35.2 (1 C, C-7-CH-*C*(CH_3)$_2$-CH_2), 31.9 (1 C, C-7-CH-C(*C*H_3)$_2$-CH_2), 29.6 (1 C, C-7-CH-C(*C*H_3)$_2$-CH_2), 27.9 (1 C, *C*-3), 27.6 (1 C, *C*-3*), 24.6 (1 C, C-7*-C-CH=C-(*C*H_3)), 21.7 (2 C, *p*-C_{Ts}-*C*H_3, *p*-C_{Ts*}-*C*H_3).

2.9 (*S*)-(5-(Hydroxymethyl)-7-(2-methylprop-1-en-1-yl)-1-tosyl-1,2,3,5-tetrahydrocyclopenta[*f*]indol-5-yl)methyl benzoate (15)

Method A:

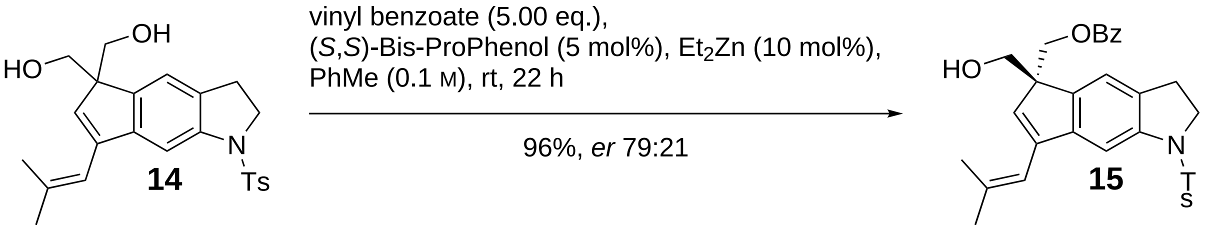

To a solution of ((2*S*,2'*S*)-((2-hydroxy-5-methyl-1,3-phenylene)bis(methylene))bis(pyrrolidine-1,2-diyl))bis(diphenylmethanol) (56 mg, 0.083 mmol, 0.05 eq.) in dry PhMe (3.3 mL) was added dropwise Et_2Zn (0.9 M in hexane, 0.20 mL, 0.166 mmol, 0.10 eq.). After stirring for 30 min at rt, the mixture was added to solution of diol **14** (705 mg, 1.66 mmol, 1.00 eq.) and vinyl benzoate (1.1 mL, 8.28 mmol, 5.00 eq.) in dry PhMe (13.2 mL). The solution was stirred at rt for 22 h. The reaction was quenched with an aqueous NaH_2PO_4 solution (5 wt%, 20 mL) and extract with TBME (3x25 mL). The combined organic phases were washed with H_2O (25 mL) and sat. NaCl (25 mL), dried over $MgSO_4$, filtered, and concentrated. Purification of the residue by flash chromatography on silica gel [petroleum ether/EtOAc (2:1) to (1:2)] gave the product **15** as colorless foam (842 mg, 1.59 mmol, 96% 56% *ee*).
Method B:

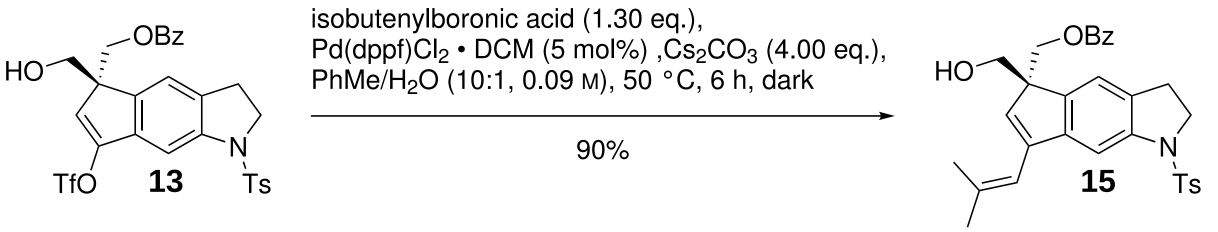

Alkenyl triflate **13** (1.56 g, 2.50 mmol, 1.00 eq.), isobutenylboronic acid (325 mg, 3.25 mmol, 1.30 eq.), Pd(dppf)Cl_2 · DCM (104 mg, 0.125 mmol, 0.05 eq.) and Cs_2CO_3 (3.26 g, 10.0 mmol, 4.00 eq.) were dissolved in PhMe (25 mL) and H_2O (2.5 mL). The red mixture was degassed with argon (3x10 min USB) and subsequently heated to 50 °C for 6 h in the dark, while it turned brown. After cooling to rt, the mixture was filtered through Celite 545, rinsed with TBME (75 mL), and concentrated. Purification of the residue by flash chromatography on silica gel [petroleum ether/EtOAc (1:2)] afforded the product **15** as a colorless foam (1.19 g, 2.24 mmol, 90%).
TLC [petroleum ether/EtOAc (1:2)]: R_f = 0.69 [vanillin: blue].
$[\alpha]_D^{20}$ = +13.6° (*c* = 0.41, MeOH).
^{1}H NMR (400 MHz, $CDCl_3$): δ [ppm] = 8.08 – 8.03 (m, 2 H, *o*-H_{Ph}), 7.68 – 7.64 (m, 3 H, *o*-H_{Ts}, 8-*H*), 7.63 – 7.57 (m, 1 H, *p*-H_{Ph}), 7.50 – 7.44 (m, 2 H, *m*-H_{Ph}), 7.24 (s, 1 H, 4-*H*), 7.21 – 7.17 (m, 2 H, *m*-H_{Ts}), 6.27 (s, 1 H, 6-*H*), 6.21 – 6.17 (m, 1 H, C-7-C*H*=C(CH_3)$_2$), 4.63 (d, *J* = 10.9 Hz, 1 H, BzO-CH_2), 4.37 (d, *J* = 10.9 Hz, 1 H, BzO-CH_2), 3.93 (t, *J* = 8.4 Hz, 2 H, 2-*H*), 3.75 (d, *J* = 11.3 Hz, 1 H, HO-CH_2), 3.61 (d, *J* = 11.3 Hz, 1 H, HO-CH_2), 2.85 (t, *J* = 8.4 Hz, 2 H, 3-*H*), 2.35 (s, 3 H, *p*-C_{Ts}-CH_3), 2.02 – 1.99 (m, 3 H, C-7-CH=C(CH_3)$_2$ (*cis* to *H*)), 1.94 – 1.91 (m, 3 H, C-7-CH=C(CH_3)$_2$ (*trans* to *H*)).
^{13}C NMR (101 MHz, $CDCl_3$): δ [ppm] = 167.4 (1 C, Ph-*C*(=O)O), 145.4 (1 C, *C*-7a), 144.1 (1 C, *p*-C_{Ts}), 142.3 (1 C, *C*-8a), 141.8 (1 C, *C*-4a), 141.6 (1 C, *C*-7), 140.7 (1 C, C-7-CH=*C*(CH_3)$_2$), 134.3 (1 C, *ipso*-C_{Ts}), 133.5 (1 C, *p*-C_{Ph}), 133.1 (1 C, *C*-6), 129.9 (1 C, *ipso*-C_{Ph}), 129.8 (2 C, *o*-C_{Ph}), 129.8 (2 C, *m*-C_{Ts}), 129.7 (1 C, *C*-3a), 128.7 (2 C, *m*-C_{Ph}), 127.4 (2 C, *o*-C_{Ts}), 120.5 (1 C, *C*-4), 116.5 (1 C, C-7-*C*H=C(CH_3)$_2$), 107.7 (1 C, *C*-8), 66.1 (1 C, BzO-*C*H_2), 64.1 (1 C, HO-*C*H_2), 57.7 (1 C, *C*-5), 50.6 (1 C, *C*-2), 28.0 (1 C, *C*-3), 27.1 (1 C, C-7-CH=C(*C*H_3)$_2$ (*cis* to *H*)), 21.6 (1 C, *p*-C_{Ts}-*C*H_3), 20.9 (1 C, C-7-CH=C(*C*H_3)$_2$ (*trans* to *H*)).
IR (diamond ATR): $\tilde{\nu}$ [cm^{-1}] = 3502 (w), 2921 (w), 2875 (w), 1715 (m), 1654 (w), 1602 (w), 1447 (m), 1447 (m), 1350 (m), 1317 (m), 1269 (s), 1160 (s), 1093 (m), 1029 (m), 1029 (m), 973 (m), 853 (m), 853 (m), 809 (m), 751 (m), 709 (s), 660 (s), 589 (s), 543 (m).
UV/Vis (MeOH): λ_{max} (lg ε) = 313 (3.61), 269 (4.01), 229 (4.61), 201 (4.59).
HRESIMS: calculated [$C_{31}H_{31}NO_5S+Na^+$]: 552.18151
found: 552.18167 (0.29 ppm)

2.10 ((5*S*,7*R*)-5-(Hydroxymethyl)-7-isobutyl-1-tosyl-1,2,3,5,6,7-hexahydrocyclopenta[*f*]indol-5-yl)methyl benzoate (17)

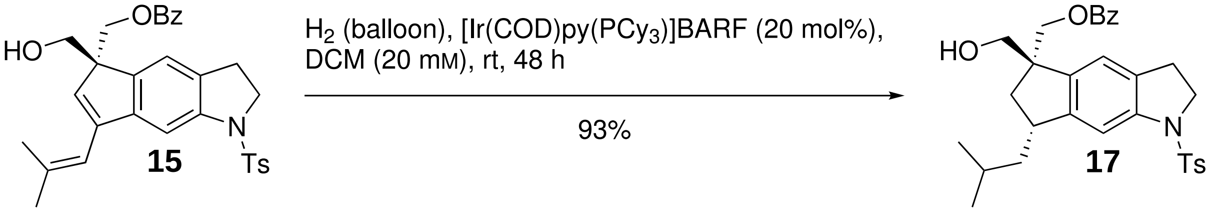

Diene **15** (265 mg, 0.500 mmol, 1.00 eq.) and [Ir(COD)py(PCy$_3$)]BARF (152 mg, 0.100 mmol, 0.20 eq.) were dissolved in dry, degassed DCM (25 mL). The orange solution was purged with H_2, turned yellow and was stirred under H_2 (balloon) at rt for 48 h. The solvent was removed under reduced pressure and purification by flash chromatography on silica gel [petroleum ether/EtOAc (3:2)] afforded the product **17** as a colorless foam (247 mg, 0.463 mmol, 93%).
TLC [petroleum ether/EtOAc (1:1)]: R_f = 0.53 [vanillin: brown].

$[\alpha]_D^{20}$ = +1.4° (*c* = 0.28, MeOH).
^{1}H NMR (300 MHz, $CDCl_3$): *δ* [ppm] = 8.06 – 8.00 (m, 2 H, *o*-H_{Ph}), 7.70 – 7.63 (m, 2 H, *o*-H_{Ts}), 7.63 – 7.55 (m, 1 H, *p*-H_{Ph}), 7.50 – 7.42 (m, 3 H, 8-*H*, *m*-H_{Ph}), 7.25 – 7.19 (m, 2 H, *m*-H_{Ts}), 7.02 (s, 1 H, 4-*H*), 4.59 (d, *J* = 11.0 Hz, 1 H, BzO-CH_2), 4.42 (d, *J* = 11.0 Hz, 1 H, BzO-CH_2), 3.98 – 3.83 (m, 2 H, 2-*H*), 3.63 (d, *J* = 11.2 Hz, 1 H, HO-CH_2), 3.56 (d, *J* = 11.3 Hz, 1 H, HO-CH_2), 3.32 – 3.17 (m, 1 H, 7-*H*), 2.87 – 2.78 (m, 2 H, 3-*H*), 2.37 (s, 3 H, *p*-C_{Ts}-CH_3), 2.37 (dd, *J* = 13.3 Hz, *J* = 7.9 Hz, 1 H, 6-*H* (*anti* zu 7-*H*)), 1.89 – 1.73 (m, 2 H, C-7-CH_2-CH-$(CH_3)_2$, C-7-CH_2-C*H*-$(CH_3)_2$), 1.68 (dd, *J* = 13.3 Hz, *J* = 8.4 Hz, 1 H, 6-*H* (*syn* zu 7-*H*)), 1.41 – 1.20 (m, 1 H, C-7-CH_2-CH-$(CH_3)_2$), 1.01 (d, *J* = 6.1 Hz, 6 H, C-7-CH_2-CH-(C$H_3)_2$).
^{13}C NMR (76 MHz, $CDCl_3$): *δ* [ppm] = 167.2 (1 C, Ph-*C*(=O)O), 148.7 (1 C, *C*-7a), 144.2 (1 C, *p*-C_{Ts}), 142.3 (1 C, *C*-8a), 139.4 (1 C, *C*-4a), 134.2 (1 C, *ipso*-C_{Ts}), 133.4 (1 C, *p*-C_{Ph}), 130.7 (1 C, *C*-3a), 130.0 (1 C, *ipso*-C_{Ph}), 129.8 (2 C, *o*-C_{Ph}), 129.8 (2 C, *m*-C_{Ts}), 128.7 (2 C, *m*-C_{Ph}), 127.5 (1 C, *o*-C_{Ts}), 120.9 (1 C, *C*-4), 111.0 (1 C, *C*-8), 68.6 (1 C, BzO-*C*H_2), 65.8 (1 C, HO-*C*H_2), 52.2 (1 C, *C*-5), 50.6 (1 C, *C*-2), 45.9 (1 C, C-7-*C*H_2-CH-$(CH_3)_2$), 40.1 (1 C, *C*-7), 39.5 (1 C, *C*-6), 27.9 (1 C, *C*-3), 26.3 (1 C, C-7-CH_2-*C*H-$(CH_3)_2$), 24.2 (1 C, C-7-CH_2-CH-(*C*$H_3)_2$), 21.7 (1 C, C-7-CH_2-CH-(*C*$H_3)_2$), 21.7 (1 C, *p*-C_{Ts}-*C*H_3).
IR (diamond ATR): $\tilde{\nu}$ [cm^{-1}] = 3528 (w), 2952 (w), 2869 (w), 1715 (m), 1602 (w), 1475 (w), 1450 (w), 1350 (m), 1314 (w), 1270 (m), 1161 (m), 1095 (m), 1031 (m), 964 (w), 879 (w), 810 (w), 750 (w), 710 (m), 660 (m), 589 (m), 542 (m).
UV/Vis (MeOH): λ_{max} (lg *ε*) = 295 (3.70), 224 (4.32), 210 (4.33).
HRESIMS: calculated [$C_{31}H_{35}NO_5S+Na^+$]: 556.21282
found: 556.21267 (0.27 ppm)

2.11 ((5*R*,7*R*)-5-Formyl-7-isobutyl-1-tosyl-1,2,3,5,6,7-hexahydrocyclopenta[*f*]indol-5-yl)methyl benzoate (S1)

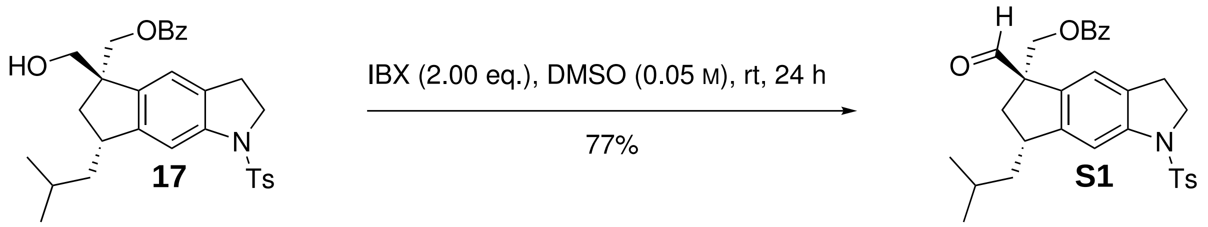

A solution of alcohol **17** (945 mg, 1.77 mmol, 1.00 eq.) and IBX (992 mg, 3.54 mmol, 2.00 eq.) in DMSO (35 mL) was stirred at rt for 24 h. The reaction was quenched with H_2O (50 mL) and extract with TBME (3x100 mL). The combined organic phases were washed with sat. $NaHCO_3$ (2x100 mL) and H_2O (2x100 mL), dried over $MgSO_4$, filtered, and concentrated. Purification of the residue by flash chromatography on silica gel [petroleum ether/EtOAc (5:2)] afforded a orange foam 498 mg. Addition of TBME to the foam afforded a orange solution and a colorless precipitate. After removal of the solvent by decanting and washing the residue with TBME the product **S1** was obtained as a colorless, amorphous solid (424 mg, 0.797 mmol, 45%).
Repeating the experiment with a fresh batch of IBX (259 mg, 0.926 mmol, 2.00 eq.) and alcohol **17** (247 mg, 0.463 mmol, 1.00 eq.), afforded the product **S1** as a colorless foam (190 mg, 0.357 mmol, 77%).
TLC [petroleum ether/EtOAc (2:1)]: R_f = 0.23 [vanillin: brown].
$[\alpha]_D^{20}$ = +1.5° (*c* = 0.54, DCM).

^{1}H NMR (500 MHz, $CDCl_3$): δ [ppm] = 9.60 (s, 1 H, C-5-C(=O)*H*), 7.97 – 7.94 (m, 2 H, *o*-H_{Ph}), 7.68 – 7.65 (m, 2 H, *o*-H_{Ts}), 7.59 – 7.55 (m, 1 H, *p*-H_{Ph}), 7.53 (s, 1 H, 8-*H*), 7.45 – 7.41 (m, 2 H, *m*-H_{Ph}), 7.25 – 7.22 (m, 2 H, *m*-H_{Ts}), 6.96 (s, 1 H, 4-*H*), 4.85 (d, *J* = 11.2 Hz, 1 H, BzO-CH_2), 4.48 (d, *J* = 11.2 Hz, 1 H, BzO-CH_2), 3.93 (t, *J* = 8.4 Hz, 2 H, 2-*H*), 3.37 – 3.27 (m, 1 H, 7-*H*), 2.92 (dd, *J* = 13.5 Hz, *J* = 8.1 Hz, 1 H, 6-*H* (*syn* to 7-*H*)), 2.89 – 2.78 (m, 2 H, 2-*H*), 2.38 (s, 3 H, *p*-C_{Ts}-CH_3), 1.87 – 1.75 (m, 3 H, 6-*H* (*anti* to 7-*H*), C-7-CH_2-CH-$(CH_3)_2$, C-7-CH_2-C*H*-$(CH_3)_2$), 1.39 (dd, *J* = 10.6 Hz, *J* = 8.8 Hz, 1 H, C-7-CH_2-CH-$(CH_3)_2$), 1.03 (d, *J* = 6.1 Hz, 3 H, C-7-CH_2-CH-(CH_3)$_2$), 1.01 (d, *J* = 6.2 Hz, 3 H, C-7-CH_2-CH-(CH_3)$_2$).
^{13}C NMR (126 MHz, $CDCl_3$): δ [ppm] = 198.5 (1 C, C-5-*C*(=O)H), 166.4 (1 C, Ph-*C*(=O)O), 149.7 (1 C, *C*-7a), 144.4 (1 C, *p*-C_{Ts}), 143.3 (1 C, *C*-8a), 134.1 (1 C, *ipso*-C_{Ts}), 133.5 (1 C, *C*-4a), 133.4 (1 C, *p*-C_{Ph}), 131.5 (1 C, *C*-3a), 129.9 (2 C, *m*-C_{Ts}), 129.8 (2 C, *o*-C_{Ph}), 129.7 (1 C, *ipso*-C_{Ph}), 128.6 (2 C, *m*-C_{Ph}), 127.4 (2 C, *o*-C_{Ts}), 121.1 (1 C, *C*-4), 111.3 (1 C, *C*-8), 67.2 (1 C, BzO-*C*H_2), 62.1 (1 C, *C*-5), 50.6 (1 C, *C*-2), 45.8 (1 C, C-7-*C*H_2-CH-$(CH_3)_2$), 40.9 (1 C, *C*-7), 37.2 (1 C, *C*-6), 27.7 (1 C, *C*-3), 26.3 (1 C, C-7-CH_2-*C*H-$(CH_3)_2$), 24.0 (1 C, C-7-CH_2-CH-(*C*H_3)$_2$), 21.7 (1 C, C-7-CH_2-CH-(*C*H_3)$_2$), 21.7 (1 C, *p*-C_{Ts}-*C*H_3).
IR (diamond ATR): $\tilde{\nu}$ [cm^{-1}] = 2956 (m), 2923 (w), 2868 (w), 1719 (s), 1602 (w), 1474 (m), 1474 (m), 1353 (m), 1312 (w), 1265 (s), 1161 (s), 1094 (s), 1063 (m), 1026 (m), 967 (m), 878 (m), 810 (m), 752 (m), 711 (s), 660 (s), 588 (s), 544 (m).
UV/Vis (MeOH): λ_{max} (lg ε) = 304 (3.77), 227 (4.37), 225 (5.00).
HRESIMS: calculated [$C_{31}H_{33}NO_5S+Na^+$]: 554.19716
found: 554.19747 (0.56 ppm)

2.12 ((5*R*,7*R*)-5-((*E*)-2-Iodovinyl)-7-isobutyl-1-tosyl-1,2,3,5,6,7-hexahydrocyclopenta[*f*]indol-5-yl)methyl benzoate (21)

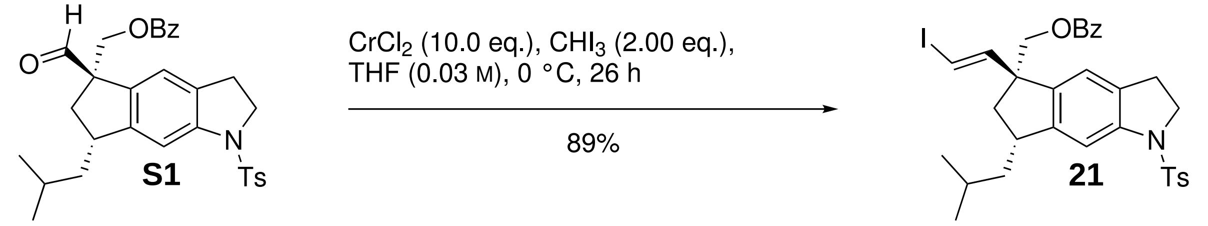

A solution of $CrCl_2$ (730 mg, 6.32 mmol, 10.0 eq.) in dry THF (15 mL) was cooled to 0 °C. Separately, a solution of aldehyde **S1** (316 mg, 0.632 mmol, 1.00 eq.) and CHI_3 (468 mg, 1.19 mmol, 2.00 eq.) in dry THF (6 mL) was cooled to 0 °C. At 0 °C the CHI_3 solution was added to the $CrCl_2$ solution and stirred at 0 °C for 26 h, while turning brown. The reaction was quenched with sat. $Na_2S_2O_3$ (20 mL), diluted with H_2O, and extracted with TBME (3x50 mL). The combined organic phases were washed with sat. NaCl, dried over $MgSO_4$, filtered, and concentrated. Purification of the residue by flash chromatography on silica gel [petroleum ether/EtOAc (4:1)] afforded the product **21** as a colorless foam (347 mg, 0.529 mmol, 89%).
TLC [petroleum ether/EtOAc (4:1)]: R_f = 0.34 [vanillin: brown].
$[\alpha]_D^{20}$ = −19.5° (*c* = 0.87, DCM).
^{1}H NMR (400 MHz, $CDCl_3$): δ [ppm] = 8.01 – 7.96 (m, 2 H, *o*-H_{Ph}), 7.66 – 7.62 (m, 2 H, *o*-H_{Ts}), 7.61 – 7.56 (m, 1 H, *p*-H_{Ph}), 7.50 (s, 1 H, 8-*H*), 7.48 – 7.43 (m, 2 H, *m*-H_{Ph}), 7.24 – 7.19 (m, 2 H, *m*-H_{Ts}), 6.91 (s, 1 H, 4-*H*), 6.77 (d, *J* = 14.8 Hz, 1 H, C-5-CH=C*H*I), 5.72 (d, *J* = 14.6 Hz, 1 H, C-5-C*H*=CHI), 4.45 (d, *J* = 10.9 Hz, 1 H, BzO-CH_2), 4.41 (d, *J* = 10.9 Hz, 1 H, BzO-CH_2),

4.01 – 3.85 (m, 2 H, 2-*H*), 3.23 – 3.10 (m, 1 H, 7-*H*), 2.89 – 2.71 (m, 2 H, 3-*H*), 2.41 (dd, *J* = 12.8 Hz, *J* = 7.2 Hz, 1 H, 6-*H* (*syn* to 7-*H*)), 2.38 (s, 3 H, *p*-C_{Ts}-CH_3), 1.86 (ddd, *J* = 13.3 Hz, *J* = 9.6 Hz, *J* = 3.9 Hz, 1 H, C-7-CH_2-CH-(CH_3)$_2$), 1.82 – 1.69 (m, 1 H, C-7-CH_2-C*H*-(CH_3)$_2$), 1.76 (dd, *J* = 12.8 Hz, *J* = 9.5 Hz, 1 H, 6-*H* (*anti* to 7-*H*)), 1.33 (ddd, *J* = 13.1 Hz, *J* = 10.8 Hz, *J* = 4.0 Hz, 1 H, C-7-CH_2-CH-(CH_3)$_2$), 1.01 (d, *J* = 6.2 Hz, 3 H, C-7-CH_2-CH-(CH_3)$_2$), 1.00 (d, *J* = 6.3 Hz, 3 H, C-7-CH_2-CH-(CH_3)$_2$).

^{13}C NMR (101 MHz, $CDCl_3$): δ [ppm] = 166.5 (1 C, Ph-*C*(=O)O), 148.9 (1 C, C-5-C*H*=CHI), 148.4 (1 C, *C*-7a), 144.2 (1 C, *p*-C_{Ts}), 142.4 (1 C, *C*-8a), 138.4 (1 C, *C*-4a), 134.2 (1 C, *ipso*-C_{Ts}), 133.3 (1 C, *p*-C_{Ph}), 131.3 (1 C, *C*-3a), 130.1 (1 C, *ipso*-C_{Ph}), 129.8 (2 C, *m*-C_{Ts}), 129.7 (2 C, *o*-C_{Ph}), 128.6 (2 C, *m*-C_{Ph}), 127.4 (2 C, *o*-C_{Ts}), 120.7 (1 C, *C*-4), 111.2 (1 C, *C*-8), 76.8 (1 C, C-5-CH=*C*HI), 69.8 (1 C, BzO-*C*H_2), 56.3 (1 C, *C*-5), 50.7 (1 C, *C*-2), 44.5 (1 C, C-7-*C*H_2-CH-(CH_3)$_2$), 43.0 (1 C, *C*-6), 39.5 (1 C, *C*-7), 27.9 (1 C, *C*-3), 26.3 (1 C, C-7-CH_2-*C*H-(CH_3)$_2$), 24.2 (1 C, C-7-CH_2-CH-(*C*H_3)$_2$), 21.7 (1 C, C-7-CH_2-CH-(*C*H_3)$_2$), 21.7 (1 C, *p*-C_{Ts}-*C*H_3).

IR (diamond ATR): $\tilde{\nu}$ [cm^{-1}] = 2954 (m), 2923 (m), 2864 (w), 1717 (m), 1599 (w), 1473 (m), 1473 (m), 1354 (m), 1313 (w), 1265 (s), 1161 (s), 1093 (s), 1063 (m), 1025 (m), 951 (m), 881 (w), 809 (m), 751 (m), 709 (s), 660 (s), 590 (s), 544 (m).

UV/Vis (THF): λ_{max} (lg ε) = 300 (3.76), 277 (4.48), 223 (5.14).

HRESIMS: calculated [$C_{32}H_{34}INO_4S+Na^+$]: 678.11454
found: 678.11483 (0.43 ppm)

2.13 ((5*R*,7*R*)-5-((*E*)-2-(1*H*-Indol-5-yl)vinyl)-7-isobutyl-1-tosyl-1,2,3,5,6,7-hexahydrocyclopenta[*f*]indol-5-yl)methyl benzoate (23)

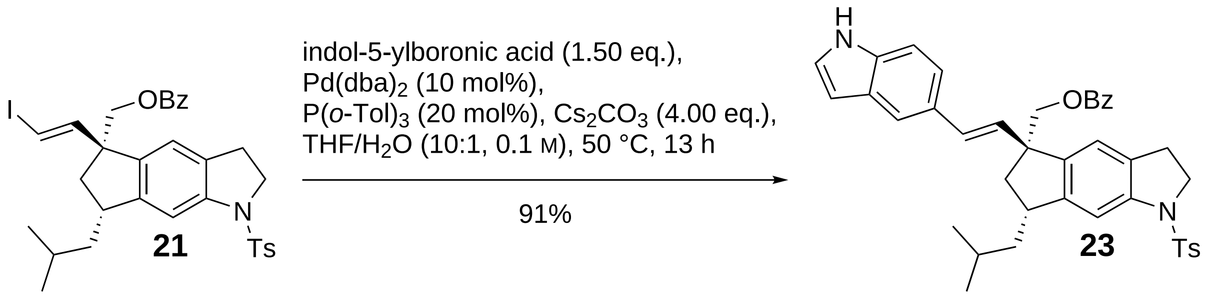

A solution of indol-5-ylboronic acid (**22**, 67 mg, 0.403 mmol, 1.50 eq.), iodoalkene **21** (176 mg, 0.268 mmol, 1.00 eq.), Pd(dba)$_2$ (15 mg, 0.027 mmol, 0.10 eq.), P(*o*-Tol)$_3$ (16 mg, 0.540 mmol, 0.20 eq.), and Cs_2CO_3 (350 mg, 1.07 mmol, 4.00 eq.) in degassed THF/H_2O (10:1, 2.75 mL) was stirred in the dark at 50 °C for 13 h. The solvent was removed under reduced pressure and purification by flash chromatography on silica gel [petroleum ether/EtOAc (4:1 to 2:1)] afforded the product **23** as a colorless foam (158 mg, 0.245 mmol, 91%).

TLC [petroleum ether/EtOAc (2:1)]: R_f = 0.37 [vanillin: red-brown].

$[\alpha]_D^{20}$ = –32.7° (*c* = 0.11, MeOH).

^{1}H NMR (500 MHz, $CDCl_3$, indole moiety marked with *): δ [ppm] = 8.14 (s, 1 H, 1*-*H*), 8.00 – 7.96 (m, 2 H, *o*-H_{Ph}), 7.68 – 7.64 (m, 2 H, *o*-H_{Ts}), 7.57 – 7.52 (m, 1 H, *p*-H_{Ph}, 8-*H*), 7.51 – 7.49 (m, 1 H, 4*-*H*), 7.44 – 7.39 (m, 2 H, *m*-H_{Ph}), 7.29 (d, *J* = 8.5 Hz, 1 H, 7*-*H*), 7.22 – 7.19 (m, 2 H, *m*-H_{Ts}, 6*-*H*), 7.17 (dd, *J* = 3.0 Hz, *J* = 2.5 Hz, 1 H, 2*-*H*), 6.99 (s, 1 H, 4-*H*), 6.48 (ddd, *J* = 3.1 Hz, *J* = 2.1 Hz, *J* = 1.0 Hz, 1 H, 3*-*H*), 6.36 (d, *J* = 16.0 Hz, 1 H, C-5-C*H*=CH), 6.15 (d, *J* = 16.0 Hz, 1 H, C-5-CH=C*H*), 4.57 (d, *J* = 10.9 Hz, 1 H, BzO-CH_2), 4.54 (d, *J* = 10.9 Hz, 1 H,

BzO-CH_2), 4.02 – 3.84 (m, 2 H, 2-*H*), 3.31 – 3.22 (m, 1 H, 7-*H*), 2.89 – 2.70 (m, 2 H, 3-*H*), 2.51 (dd, *J* = 12.6 Hz, *J* = 7.2 Hz, 1 H, 6-H (*syn* to 7-*H*)), 2.50 (s, 1 H, 6-H (*syn* to 7-*H*)), 2.37 (s, 3 H, *p*-C_{Ts}-CH_3), 1.94 – 1.86 (m, 1 H, C-7-CH_2-CH-(CH_3)$_2$), 1.91 (dd, *J* = 12.6 Hz, *J* = 9.5 Hz, 1 H, 6-H (*anti* to 7-*H*)), 1.84 – 1.74 (m, 1 H, C-7-CH_2-C*H*-(CH_3)$_2$), 1.39 (ddd, *J* = 13.1 Hz, *J* = 10.8 Hz, *J* = 4.2 Hz, 1 H, C-7-CH_2-CH-(CH_3)$_2$), 1.01 (d, *J* = 6.5 Hz, 6 H, C-7-CH_2-CH-(CH_3)$_2$).

^{13}C NMR (126 MHz, $CDCl_3$, indole moiety marked with *): δ [ppm] = 166.8 (1 C, Ph-*C*(=O)O), 148.5 (1 C, *C*-7a), 144.1 (1 C, *p*-C_{Ts}), 141.9 (1 C, *C*-8a), 141.0 (1 C, *C*-4a), 135.5 (1 C, *C*-7a*), 134.2 (1 C, *ipso*-C_{Ts}), 133.1 (1 C, *p*-C_{Ph}), 131.0 (1 C, C-5-*C*H=CH), 131.0 (1 C, *C*-3a), 130.4 (1 C, *ipso*-C_{Ph}), 130.2 (1 C, C-5-CH=*C*H), 129.7 (2 C, *m*-C_{Ts}), 129.7 (2 C, *o*-C_{Ph}), 129.4 (1 C, *C*-5*), 128.6 (2 C, *m*-C_{Ph}), 128.2 (1 C, *C*-3a*), 127.5 (2 C, *o*-C_{Ts}), 124.8 (1 C, *C*-2*), 120.8 (1 C, *C*-4), 120.5 (1 C, *C*-6*), 119.1 (1 C, *C*-4*), 111.2 (1 C, *C*-7*), 111.2 (1 C, *C*-8), 102.9 (1 C, *C*-3*), 70.7 (1 C, BzO-*C*H_2), 52.6 (1 C, *C*-5), 50.7 (1 C, *C*-2), 44.7 (1 C, C-7-*C*H_2-CH-(CH_3)$_2$), 43.8 (1 C, *C*-6), 39.7 (1 C, *C*-7), 28.0 (1 C, *C*-3), 26.4 (1 C, C-7-CH_2-*C*H-(CH_3)$_2$), 24.3 (1 C, C-7-CH_2-CH-(*C*H_3)$_2$), 21.8 (1 C, C-7-CH_2-CH-(*C*H_3)$_2$), 21.7 (1 C, *p*-C_{Ts}-*C*H_3).

IR (diamond ATR): $\tilde{\nu}$ [cm^{-1}] = 3403 (w), 2955 (w), 1713 (m), 1601 (w), 1472 (m), 1348 (m), 1268 (m), 1160 (s), 1092 (m), 1065 (m), 1025 (m), 966 (m), 881 (w), 803 (m), 756 (m), 711 (s), 660 (m), 590 (s), 546 (m).

UV/Vis (MeOH): λ_{max} (lg ε) = 295 (4.11), 249 (4.65), 227 (4.58), 201 (4.72).

HRESIMS: calculated [$C_{40}H_{40}N_2O_4S+Na^+$]: 667.26010
found: 667.26027 (0.25 ppm)

2.14 ((5*R*,7*R*)-5-((*E*)-2-(1*H*-Indol-5-yl)vinyl)-7-isobutyl-1-tosyl-1,2,3,5,6,7-hexahydrocyclopenta[*f*]indol-5-yl)methanol (S2)

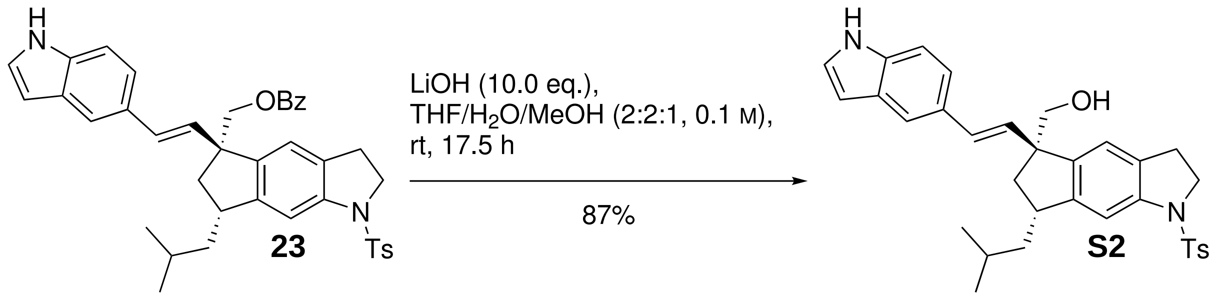

A solution of benzoate **23** (158 mg, 0.245 mmol, 1.00 eq.) and LiOH · H_2O (103 mg, 2.45 mmol, 10.0 eq.) in THF/H_2O/MeOH (2:2:1, 2.5 mL) was stirred at rt for 17.5 h. The solution was extracted with TBME (3x10 mL). The combined organic phases were washed with sat. NaCl, dried over $MgSO_4$, filtered, and concentrated. Purification of the residue by flash chromatography on silica gel [petroleum ether/EtOAc (2:1 to 1:1)] afforded the product **S2** as a colorless foam (115 mg, 0.213 mmol, 87%).

TLC [petroleum ether/EtOAc (1:1)]: R_f = 0.55 [vanillin: red-brown].

$[\alpha]_D^{20}$ = −1.9° (*c* = 0.21, THF).

^{1}H NMR (500 MHz, $CDCl_3$, indole moiety marked with *): δ [ppm] = 8.18 (s, 1 H, 1*-*H*), 7.69 – 7.66 (m, 2 H, *o*-H_{Ts}), 7.51 – 7.50 (m, 1 H, 4*-*H*), 7.50 (s, 1 H, 8-*H*), 7.29 (d, *J* = 8.5 Hz, 1 H, 7*-*H*), 7.25 – 7.22 (m, 2 H, *m*-H_{Ts}), 7.21 (dd, *J* = 8.6 Hz, *J* = 1.6 Hz, 1 H, 6*-*H*), 7.16 (dd, *J* = 3.1 Hz, *J* = 2.4 Hz, 1 H, 2*-*H*), 7.02 (s, 1 H, 4-*H*), 6.47 (ddd, *J* = 3.1 Hz, *J* = 2.1 Hz, *J* = 1.0 Hz, 1 H, 3*-*H*), 6.23 (d, *J* = 16.0 Hz, 1 H, C-5-C*H*=CH), 6.16 (d, *J* = 16.1 Hz, 1 H, C-5-CH=C*H*), 3.99 (ddd,

J = 10.6 Hz, J = 9.4 Hz, J = 6.7 Hz, 1 H, 2-*H*), 3.91 (ddd, J = 10.6 Hz, J = 9.7 Hz, J = 7.7 Hz, 1 H, 2-*H*), 3.85 (d, J = 10.7 Hz, 1 H, HO-CH_2), 3.78 (d, J = 11.0 Hz, 1 H, HO-CH_2), 3.25 – 3.16 (m, 1 H, 7-*H*), 2.91 – 2.77 (m, 2 H, 3-*H*), 2.39 (s, 3 H, *p*-C_{Ts}-CH_3), 2.36 (dd, J = 12.7 Hz, J = 7.2 Hz, 1 H, 6-H (*syn* to 7-*H*)), 1.89 – 1.74 (m, 3 H, 6-H (*anti* to 7-*H*), C-7-CH_2-CH-$(CH_3)_2$, C-7-CH_2-C*H*-$(CH_3)_2$), 1.35 (ddd, J = 12.7 Hz, J = 10.8 Hz, J = 3.7 Hz, 1 H, C-7-CH_2-CH-$(CH_3)_2$), 1.01 (d, J = 6.4 Hz, 3 H, C-7-CH_2-CH-(CH_3)$_2$), 1.00 (d, J = 6.4 Hz, 3 H, C-7-CH_2-CH-(CH_3)$_2$).
^{13}C NMR (126 MHz, $CDCl_3$, indole moiety marked with *): δ [ppm] = 149.1 (1 C, *C*-7a), 144.1 (1 C, *p*-C_{Ts}), 141.8 (1 C, *C*-8a), 140.9 (1 C, *C*-4a), 135.5 (1 C, *C*-7a*), 134.3 (1 C, *ipso*-C_{Ts}), 131.1 (1 C, *C*-3a), 130.9 (1 C, C-5-*C*H=CH), 130.8 (1 C, C-5-CH=*C*H), 129.7 (2 C, *m*-C_{Ts}), 129.2 (1 C, *C*-5*), 128.2 (1 C, *C*-3a*), 127.5 (2 C, *o*-C_{Ts}), 124.9 (1 C, *C*-2*), 120.6 (1 C, *C*-4), 120.5 (1 C, *C*-6*), 119.1 (1 C, *C*-4*), 111.2 (1 C, *C*-7*), 111.0 (1 C, *C*-8), 102.9 (1 C, *C*-3*), 68.9 (1 C, HO-*C*H_2), 54.8 (1 C, *C*-5), 50.7 (1 C, *C*-2), 44.6 (1 C, C-7-*C*H_2-CH-$(CH_3)_2$), 42.6 (1 C, *C*-6), 39.6 (1 C, *C*-7), 27.9 (1 C, *C*-3), 26.4 (1 C, C-7-CH_2-*C*H-$(CH_3)_2$), 24.3 (1 C, C-7-CH_2-CH-(*C*H_3)$_2$), 21.7 (1 C, C-7-CH_2-CH-(*C*H_3)$_2$), 21.7 (1 C, *p*-C_{Ts}-*C*H_3).
IR (diamond ATR): $\tilde{\nu}$ [cm^{-1}] = 3397 (w), 2952 (w), 2923 (w), 2863 (w), 1732 (w), 1598 (w), 1473 (m), 1437 (w), 1347 (m), 1246 (m), 1160 (s), 1090 (m), 1038 (m), 962 (w), 877 (w), 805 (m), 765 (w), 725 (w), 660 (s), 590 (s), 544 (s), 426 (w).
UV/Vis (THF): λ_{max} (lg ε) = 292 (3.99), 242 (4.20).
HRESIMS: calculated [$C_{33}H_{36}N_2O_3S$+Na^+]: 563.23380
found: 563.23401 (0.37 ppm)

2.15 ((5*R*,7*R*)-5-((*E*)-2-(1*H*-indol-5-yl)vinyl)-7-isobutyl-1,2,3,5,6,7-hexahydrocyclopenta[*f*]indol-5-yl)methanol (S3)

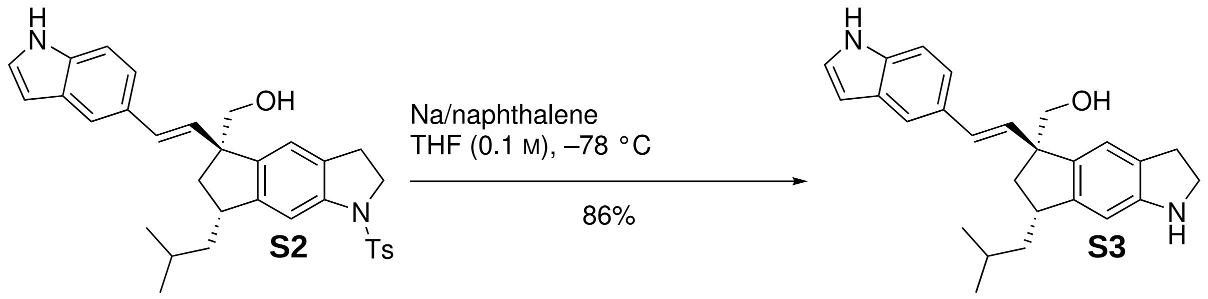

Sulfonamide **S2** (178 mg, 0.329 mmol, 1.00 eq.) was dissolved in dry THF (3.3 mL) and cooled to –78 °C. Separately, freshly cut sodium (227 mg, 9.88 mmol, 30.0 eq.) was added to a solution of naphthalene (633 mg, 4.94 mmol, 15.0 eq.) in dry THF (25 mL). This suspension was treated by sonication for 30 min, which gave a deep green solution (~0.2 M). The Na/naphthalene solution was added dropwise to the starting material at –78 °C. After full consumption of the starting material (monitored by TLC), the reaction was quenched with sat. NH_4Cl (20 mL) and extracted with TBME (3x20 mL). The combined organic phases were washed with sat. NaCl, dried over $MgSO_4$, filtered, and concentrated. Purification of the residue by flash chromatography on silica gel [petroleum ether/EtOAc (4:1 to 2:1)] afforded the product **S3** as a colorless foam (110 mg, 0.285 mmol, 86%).
TLC [petroleum ether/EtOAc (1:1)]: R_f = 0.35 [vanillin: red (pre heating: yellow)].
$[\alpha]_D^{20}$ = –120.2° (c = 0.23, MeOH).

^{1}H NMR (500 MHz, $CDCl_3$, indole moiety marked with *): δ [ppm] = 8.12 (s, 1 H, 1*-*H*), 7.55 (s, 1 H, 4*-*H*), 7.28 (d, *J* = 8.5 Hz, 1 H, 7*-*H*), 7.24 (dd, *J* = 8.5 Hz, *J* = 1.6 Hz, 1 H, 6*-*H*), 7.16 (dd, *J* = 3.1 Hz, *J* = 2.5 Hz, 1 H, 2*-*H*), 7.04 (s, 1 H, 4-*H*), 6.54 (s, 1 H, 8-*H*), 6.48 (ddd, *J* = 3.1 Hz, *J* = 2.1 Hz, *J* = 0.9 Hz, 1 H, 3*-*H*), 6.30 (d, *J* = 16.1 Hz, 1 H, C-5-C*H*=CH), 6.25 (d, *J* = 16.1 Hz, 1 H, C-5-CH=C*H*), 3.87 (d, *J* = 10.8 Hz, 1 H, HO-CH_2), 3.80 (d, *J* = 10.9 Hz, 1 H, HO-CH_2), 3.63 – 3.54 (m, 2 H, 2-*H*), 3.16 – 2.95 (m, 3 H, 7-*H*, 3-*H*), 2.35 (dd, *J* = 12.5 Hz, *J* = 7.3 Hz, 1 H, 6-H (*syn* to 7-H)), 1.87 (dd, *J* = 12.5 Hz, *J* = 9.4 Hz, 1 H, 6-H (*anti* to 7-H)), 1.82 – 1.67 (m, 2 H, C-7-CH_2-C*H*-$(CH_3)_2$, C-7-CH_2-CH-$(CH_3)_2$), 1.34 (ddd, *J* = 12.7 Hz, *J* = 10.7 Hz, *J* = 3.9 Hz, 1 H, C-7-CH_2-CH-$(CH_3)_2$), 0.96 (d, *J* = 6.4 Hz, 3 H, C-7-CH_2-CH-(CH_3)$_2$ (A)), 0.96 (d, *J* = 6.4 Hz, 3 H, C-7-CH_2-CH-(CH_3)$_2$ (B)).

^{13}C NMR (126 MHz, $CDCl_3$, indole moiety marked with *): δ [ppm] = 151.6 (1 C, *C*-8a), 148.7 (1 C, *C*-7a), 135.4 (1 C, *C*-7a*), 135.2 (1 C, *C*-4a), 131.9 (1 C, C-5-*C*H=CH), 130.2 (1 C, C-5-H=*C*H), 129.6 (1 C, *C*-5*), 128.4 (1 C, *C*-3a*), 128.2 (1 C, *C*-3a), 124.7 (1 C, *C*-2*), 120.6 (1 C, *C*-6*), 120.1 (1 C, *C*-4), 119.1 (1 C, *C*-4*), 111.1 (1 C, *C*-7*), 105.3 (1 C, *C*-8), 103.0 (1 C, *C*-3*), 69.0 (1 C, HO-*C*H_2), 54.8 (1 C, *C*-5), 48.0 (1 C, *C*-2), 44.8 (1 C, C-7-*C*H_2-CH-$(CH_3)_2$), 42.6 (1 C, *C*-6), 39.5 (1 C, *C*-7), 29.9 (1 C, *C*-3), 26.4 (1 C, C-7-CH_2-*C*H-$(CH_3)_2$), 24.3 (1 C, C-7-CH_2-CH-(*C*H_3)$_2$ (A)), 21.8 (1 C, C-7-CH_2-CH-(*C*H_3)$_2$ (B)).

IR (diamond ATR): $\tilde{\nu}$ [cm^{-1}] = 3399 (m), 3321 (m), 3281 (m), 3017 (w), 2951 (m), 2901 (m), 2863 (m), 1614 (m), 1467 (m), 1385 (w), 1364 (w), 1327 (m), 1278 (m), 1198 (w), 1166 (w), 1055 (w), 968 (m), 886 (w), 851 (w), 797 (m), 764 (m), 729 (s), 606 (m), 551 (w), 519 (w), 428 (w).

UV/Vis (MeOH): λ_{max} (lg ε) = 248 (4.65), 205 (4.57).

HRESIMS: calculated [$C_{26}H_{30}N_2O+H^+$]: 387.24309
found: 387.24340 (0.80 ppm)

2.16 ((5*R*,7*R*)-5-((*E*)-2-(1*H*-indol-5-yl)vinyl)-7-isobutyl-1,5,6,7-tetrahydrocyclopenta[*f*]indol-5-yl)methanol (3)

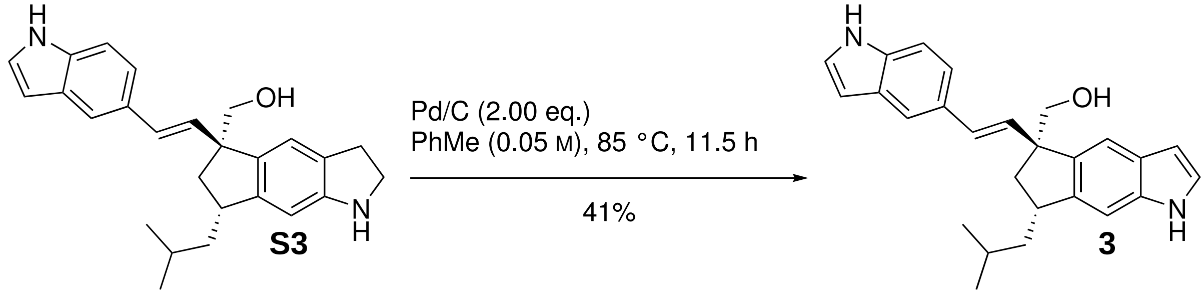

To a solution of indoline **S3** (51 mg, 0.132 mmol, 1.00 eq.) in dry, degassed PhMe (2.6 mL) was added palladium on charcoal (10% Pd on C, 281 mg, 0.264 mmol, 2.00 eq.). The suspension was stirred at 85 °C for 11.5 h. After cooling to rt, the suspension was filtered trough Celite 545, rinsed with $CHCl_3$ (50 mL) and concentrated. Purification of the residue by flash chromatography on silica gel [petroleum ether/EtOAc (4:1)] afforded the product **3** (21 mg, 0.055 mmol, 41%).

TLC [petroleum ether/EtOAc (3:1)]: R_f = 0.19 [vanillin: red].

$[\alpha]_D^{20}$ = −75.3° (*c* = 0.75, MeOH).

^{1}H NMR (500 MHz, $CDCl_3$, indole moiety marked with *): δ [ppm] = 8.09 (s, 1 H, 1-*H*), 8.07 (s, 1 H, 1*-*H*), 7.53 (s, 1 H, 4-*H*), 7.51 (s, 1 H, 4*-*H*), 7.25 (d, *J* = 8.5 Hz, 1 H, 7*-*H*), 7.23

(d, J = 0.9 Hz, 1 H, 8-H), 7.21 (dd, J = 8.6 Hz, J = 1.6 Hz, 1 H, 6*-H), 7.17 (dd, J = 2.8 Hz, J = 2.8 Hz, 1 H, 2-H), 7.12 (dd, J = 3.4 Hz, J = 2.5 Hz, 1 H, 2*-H), 6.54 (ddd, J = 3.1 Hz, J = 2.1 Hz, J = 0.9 Hz, 1 H, 3-H), 6.45 (ddd, J = 3.1 Hz, J = 2.1 Hz, J = 0.9 Hz, 1 H, 3*-H), 6.33 (d, J = 16.0 Hz, 1 H, C-5-CH=CH), 6.28 (d, J = 16.1 Hz, 1 H, C-5-CH=CH), 4.00 (d, J = 11.0 Hz, 1 H, HO-CH_2), 3.90 (d, J = 10.9 Hz, 1 H, HO-CH_2), 3.34 – 3.26 (m, 1 H, 7-H), 2.43 (dd, J = 12.3 Hz, J = 7.0 Hz, 1 H, 6-H (*syn* to 7-H)), 1.97 (dd, J = 12.3 Hz, J = 10.1 Hz, 1 H, 6-H (*anti* to 7-H)), 1.89 – 1.78 (m, 2 H, C-7-CH_2-CH-$(CH_3)_2$, C-7-CH_2-CH-$(CH_3)_2$), 1.43 (dd, J = 10.7 Hz, J = 9.1 Hz, 1 H, C-7-CH_2-CH-$(CH_3)_2$), 1.00 (d, J = 5.2 Hz, 6 H, C-7-CH_2-CH-(CH_3)$_2$).

^{13}C NMR (126 MHz, $CDCl_3$, indole moiety marked with *): δ [ppm] = 144.4 (1 C, *C*-7a), 137.4 (1 C, *C*-4a), 136.2 (1 C, *C*-8a), 135.4 (1 C, *C*-7a*), 131.9 (1 C, C-5-*C*H=CH), 130.6 (1 C, C-5-CH=*C*H), 129.5 (1 C, *C*-5*), 128.2 (1 C, *C*-3a*), 127.3 (1 C, *C*-3a), 124.7 (1 C, *C*-2*), 124.0 (1 C, *C*-2), 120.6 (1 C, *C*-6*), 119.1 (1 C, *C*-4*), 115.3 (1 C, *C*-4), 111.1 (1 C, *C*-7*), 106.1 (1 C, *C*-8), 103.0 (1 C, *C*-3*), 102.7 (1 C, *C*-3), 68.9 (1 C, HO-*C*H_2), 54.8 (1 C, *C*-5), 44.8 (1 C, C-7-*C*H_2-CH-$(CH_3)_2$), 43.0 (1 C, *C*-6), 39.1 (1 C, *C*-7), 26.4 (1 C, C-7-CH_2-*C*H-$(CH_3)_2$), 24.4 (1 C, C-7-CH_2-CH-(*C*H_3)$_2$), 21.9 (1 C, C-7-CH_2-CH-(*C*H_3)$_2$).

IR (diamond ATR): $\tilde{\nu}$ [cm^{-1}] = 3045 (w), 2920 (s), 2853 (m), 1460 (m), 1376 (w), 1342 (w), 1262 (w), 1197 (w), 1163 (w), 1094 (w), 1024 (w), 970 (w), 888 (w), 851 (w), 801 (w), 763 (w), 727 (w).

UV/Vis (MeOH): λ_{max} (lg ε) = 298 (3.82), 248 (4.37), 222 (4.29).

HRESIMS: calculated [$C_{26}H_{28}N_2O$+Na^+]: 407.20938
found: 407.20952 (0.34 ppm)

3.1 (*rac*)-Benzoate (*rac*)-13

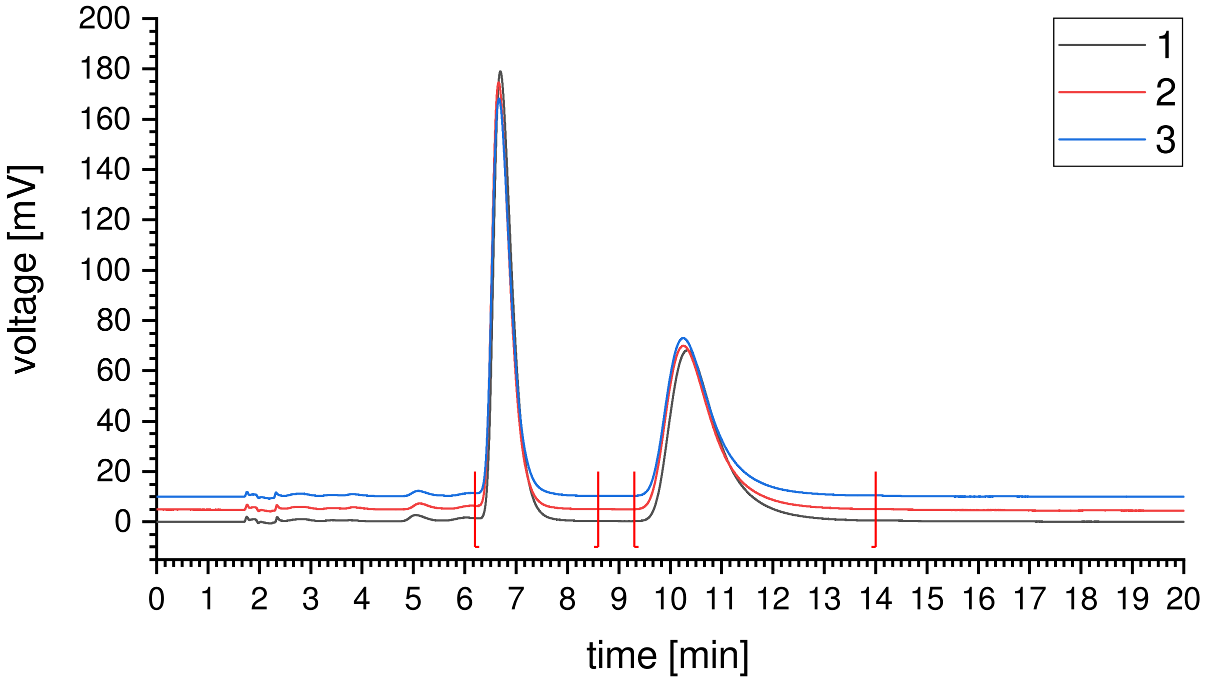

Figure S1. Chiral phase HPLC chromatogram of (*rac*)-benzoate (*rac*)-**13**, phenomenex Lux Amylose 2, 150x4.6 mm, 1 mL min^{-1} *n*-hexane/EtOH (75:25), 270 nm, 20 µL injection volume, 1 mg mL^{-1} sample concentration. The chromatograms are baseline corrected (straight line, run 1: (0|−0.10) – (20|−0.42), run 2: (0|−0.11) – (20|−0.51), run 3: (0|−0.46) – (20|−0.77)) and stacked (5 mV). The integral limits are displayed and the results are given in Table S1.

Table S1. Integration results for the chromatograms in Figure S1.

run	peak	retention time [min]	area [mV min]	area [%]	height [mV]	FWHM [min]	*ee* [%]
1	1	6.69	79.13	48.66	179.03	0.40	0.29
	2	10.33	78.66	48.38	68.22	0.99	
2	1	6.66	73.22	48.69	169.64	0.39	0.85
	2	10.25	71.99	47.87	64.98	0.96	
3	1	6.67	70.27	48.57	158.18	0.40	0.05
	2	10.25	70.20	48.52	63.04	0.95	

3.2 Benzoate 13

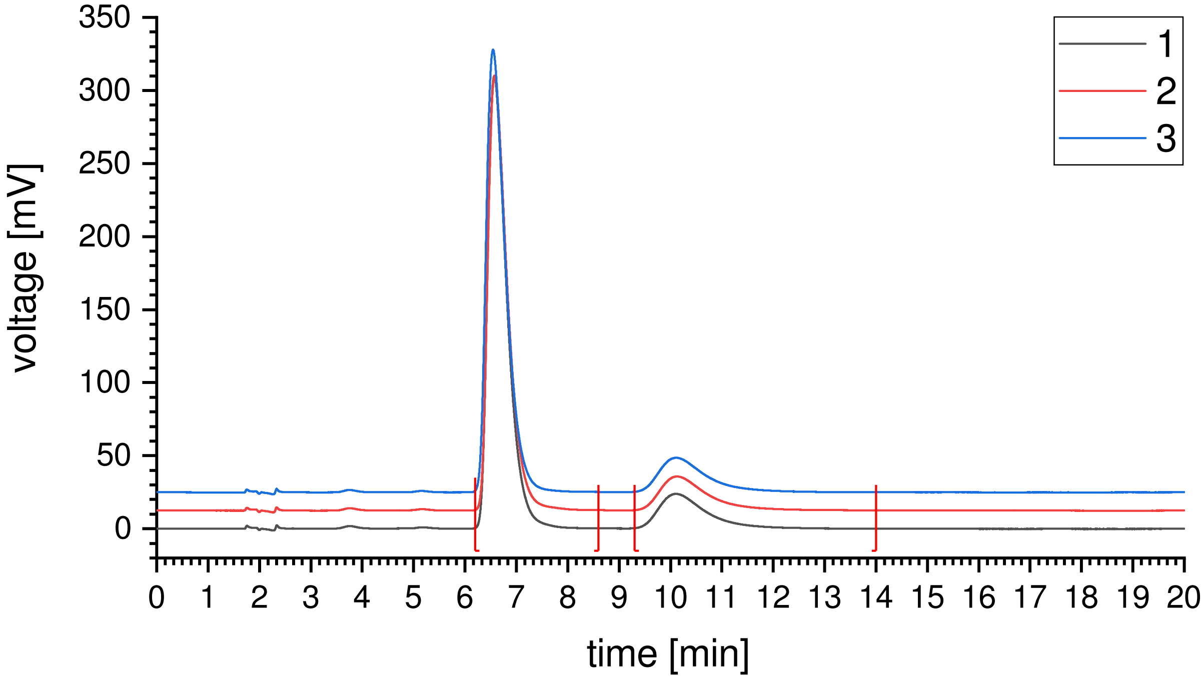

Figure S2. Chiral phase HPLC chromatogram of benzoate **13**, phenomenex Lux Amylose 2, 150x4.6 mm, 1 mL min^{-1} *n*-hexane/EtOH (75:25), 270 nm, 20 µL injection volume, 1 mg mL^{-1} sample concentration. The chromatograms are baseline corrected (straight line, run 1: (0|−4.11) – (20|−4.32), run 2: (0|−4.35) – (20|−4.65), run 3: (0|−4.57) – (20|−4.91)) and stacked (12.5 mV). The integral limits are displayed and the results are given in Table S2.

Table S2. Integration results for the chromatograms in Figure S2.

run	peak	retention time [min]	area [mV min]	area [%]	height [mV]	FWHM [min]	*ee* [%]
1	1	6.57	137.19	82.65	309.78	0.40	67.84
	2	10.11	26.29	15.84	23.89	0.95	
2	1	6.58	132.55	82.75	297.70	0.40	67.55
	2	10.13	25.67	16.03	23.29	0.94	
3	1	6.55	134.04	82.88	302.77	0.40	67.86
	2	10.11	25.66	15.87	23.59	0.94	

[H]

3.3 Dihydroraputindole D (3)

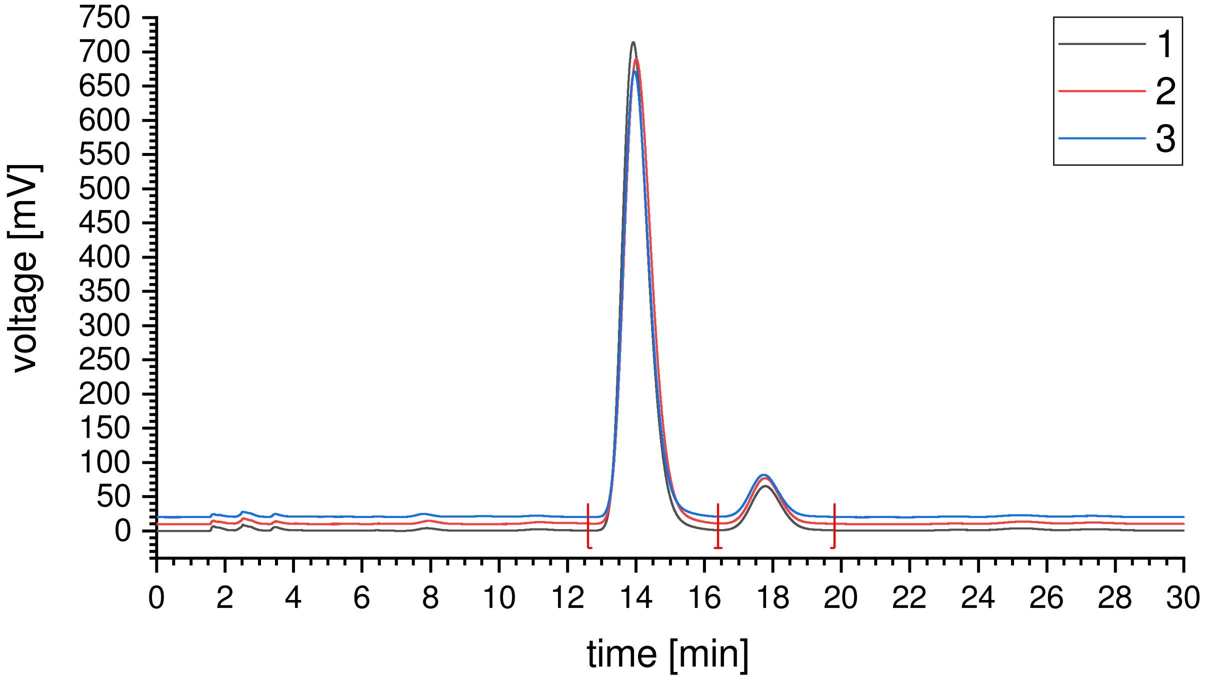

Figure S3. Chiral phase HPLC chromatogram of dihydroraputindole D **3**, phenomenex Lux Cellulose 1, 150x4.6 mm, 1 mL min^{-1} *n*-hexane/EtOH (85:15), 250 nm, 20 µL injection volume, 1 mg mL^{-1} sample concentration. The chromatograms are baseline corrected (straight line, run 1: (0|0.67) – (20|–0.02), run 2: (0|–0.77) – (20|–1.44), run 3: (0|1.89) – (20|–2.15)) and stacked (10 mV). The integral limits are displayed and the results are given in Table S3.

Table S3. Integration results for the chromatograms in Figure S3.

run	peak	retention time [min]	area [mV min]	area [%]	height [mV]	FWHM [min]	*ee* [%]
1	1	13.92	658.80	87.00	714.21	0.85	80.60
	2	17.78	70.76	9.34	65.33	0.99	
2	1	14.001	660.58	87.86	679.51	0.90	80.90
	2	17.77	69.75	9.28	67.05	0.96	
3	1	13.96	600.56	87.38	651.51	0.85	80.74
	2	17.74	64.00	9.31	61.82	0.95	

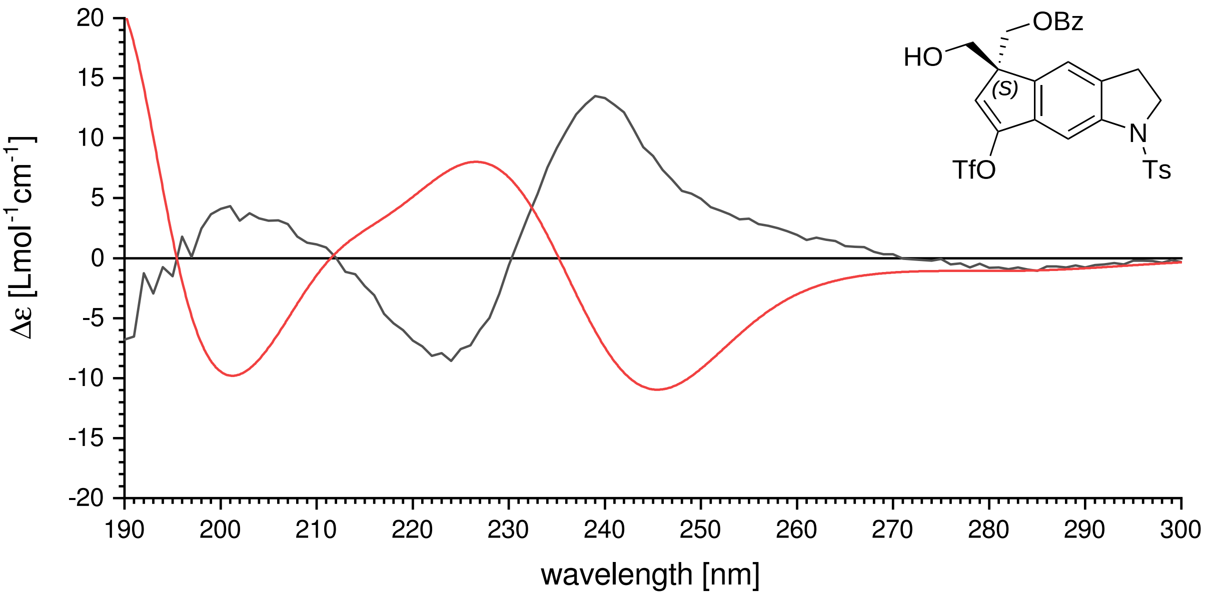

Figure S4. Experimental (black, c = 0.05 mg mL^{-1} in MeCN, path length: 2 mm) and calculated (red, (*R*)) ECD spectra of triflate **13**. The confrontational analysis employing MMFF with Monte-Carlo searching gave 313 conformers in an energy range of 5 kcal mol^{-1} with respect to the most stable one. The optimization of these conformers by DFT calculations at the B3LYP/6-31G(D) level gave 43 conformers in an energy range of 5 kcal mol^{-1} with respect to the most stable one. These conformers were again optimized by DFT calculations at the ωB97XD/TApr-cc-pVDZ level with acetonitrile as the solvent (PCM). A total of 38 conformers were not identical to each other. The 12 conformers which are at least 1% populated were submitted to TDDFT calculations at the ωB97XD/TApr-cc-pVDZ level with acetonitrile as the solvent (PCM).

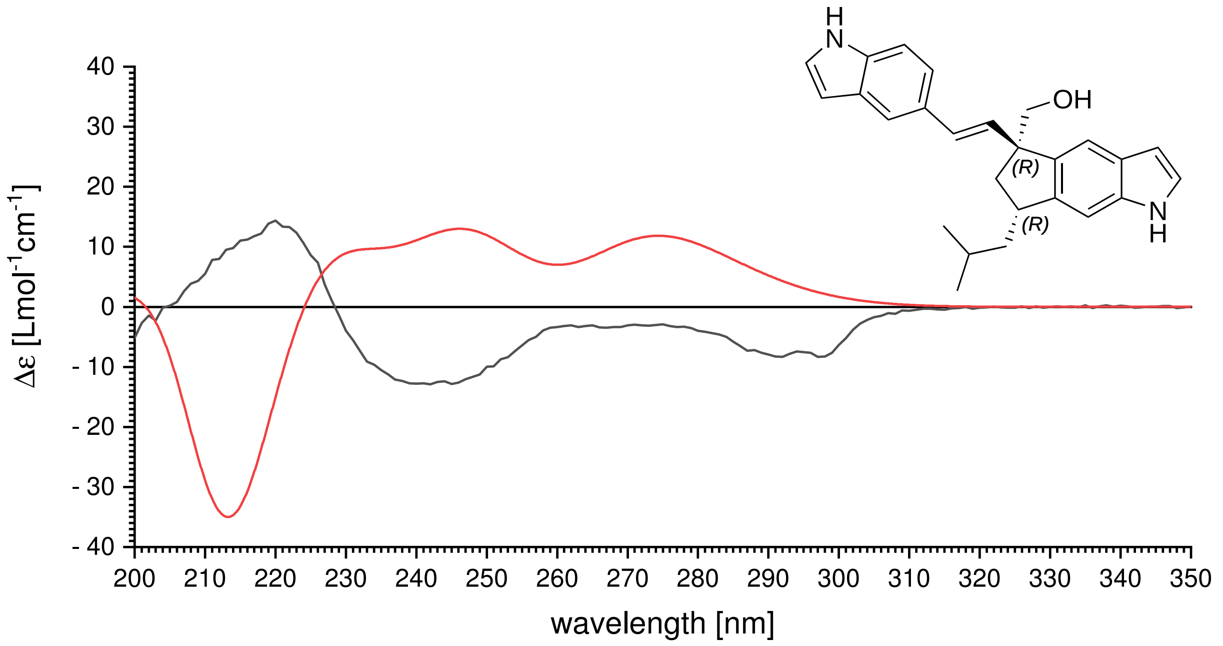

Figure S5. Experimental (black, c = 0.05 mg mL^{-1} in MeOH, path length: 2 mm) and calculated (red, (5*S*,7*S*)) ECD spectra of dihydroraputindole D (**3**). The confrontational analysis employing MMFF with Monte-Carlo searching gave 382 conformers in an energy range of 10 kcal mol^{-1} with respect to the most stable one. The optimization of these conformers by DFT calculations at the B3LYP/6-31G(D) level gave 106 conformers in an energy range of 5 kcal mol^{-1} with respect to the most stable one. These conformers were again optimized by DFT calculations at the ωB97XD/TApr-cc-pVDZ level with methanol as the solvent (PCM). A total of 106 conformers were not identical to each other. The 7 conformers which are at least 1% populated were submitted to TDDFT calculations at the ωB97XD/TApr-cc-pVDZ level with methanol as the solvent (PCM).

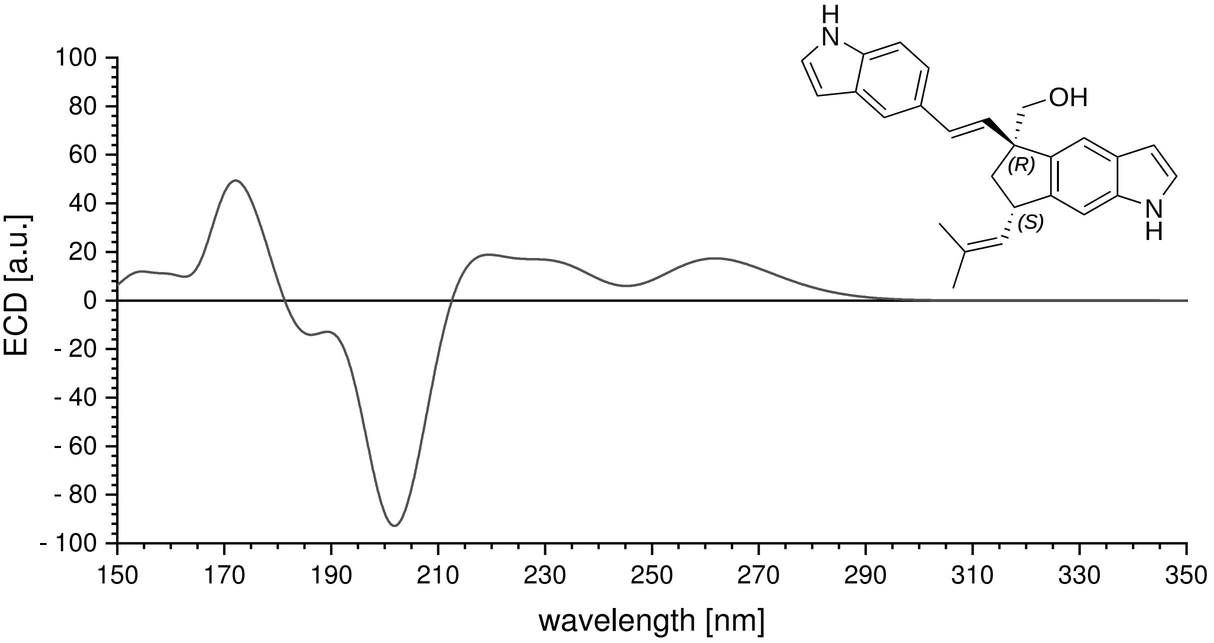

Figure S6. Calculated ((5*R*,7*S*)) ECD spectra of raputindole D **2**. The confrontational analysis employing MMFF with Monte-Carlo searching gave 92 conformers in an energy range of 10 kcal mol^{-1} with respect to the most stable one. The optimization of these conformers by DFT calculations at the B3LYP/6-31G(D) level gave 53 conformers in an energy range of 5 kcal mol^{-1} with respect to the most stable one. These conformers were again optimized by DFT calculations at the ωB97XD/TApr-cc-pVDZ level with methanol as the solvent (PCM). A total of 53 conformers were not identical to each other. The 15 conformers which are at least 1% populated were submitted to TDDFT calculations at the ωB97XD/TApr-cc-pVDZ level with methanol as the solvent (PCM).

Table S4. Comparison of experimental and calculated optical rotations of triflate **13**, dihydroraputindole D (**3**) and raputindole D (**2**).

	experimental		calculated	
	conditions	$[\alpha]_D^{20}$	$[\alpha]_D^{20}$	stereochemistry
triflate **13**	*c* = 2.00, MeCN	+49.5°	−170.2°	(*R*)
dihydroraputindole D (**3**)	*c* = 0.75, MeOH	−75.3°	+415.1°	(5*S*,7*S*)
raputindole D (**2**)[a]	*c* = 0.091, MeOH	+22.3°	+188.4°	(5*R*,7*S*)

[a]: Experimental value by Skaltsounis et. al.[4]

[4] K. Vougogiannopoulou, N. Fokialakis, N. Aligiannis, C. Cantrell, A.-L. Skaltsounis, *Org. Lett.* **2010**, *12*, 1908–1911.

5.1 6-Ethynyl-3,3,9,9-tetraisopropyl-2,10-dimethyl-4,8-dioxa-3,9-disilaundecan-6-ol (7)

^{1}H NMR (300 MHz, $CDCl_3$)

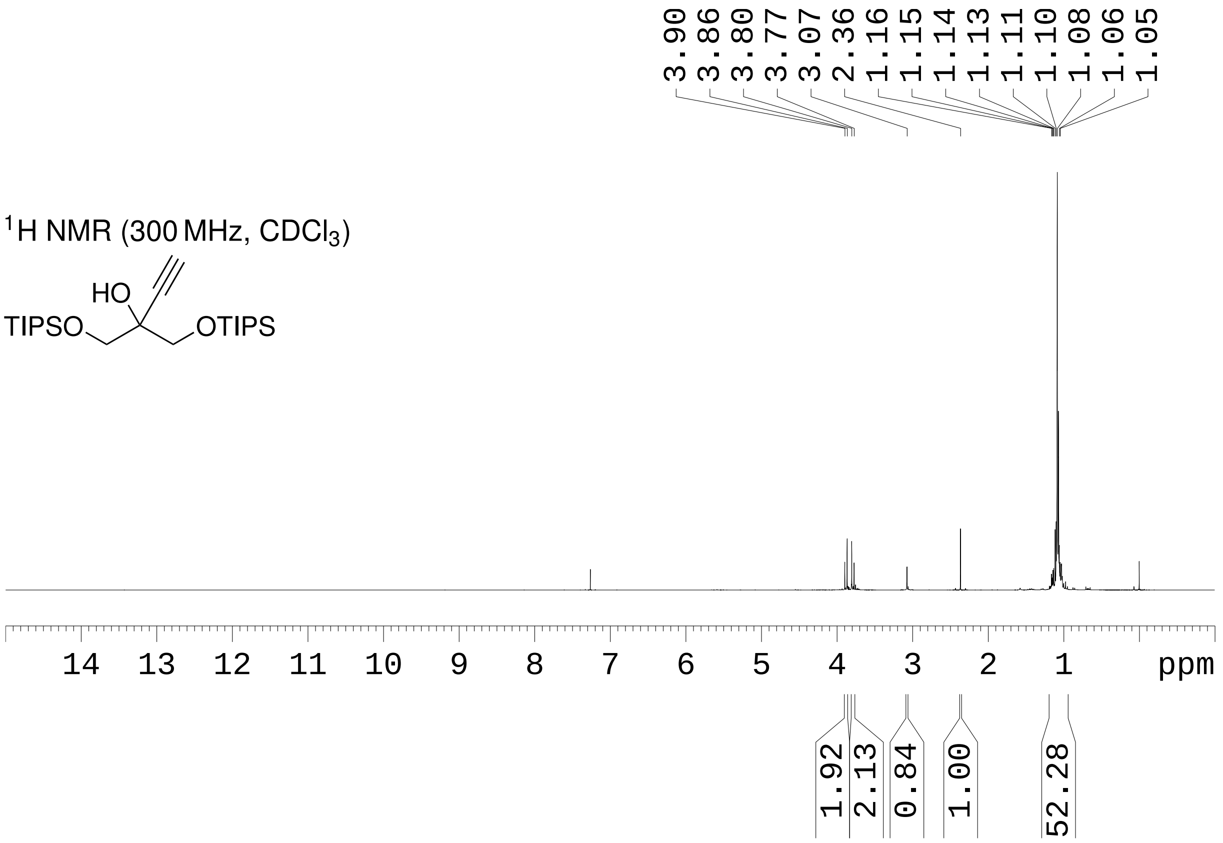

^{13}C NMR (76 MHz, $CDCl_3$)

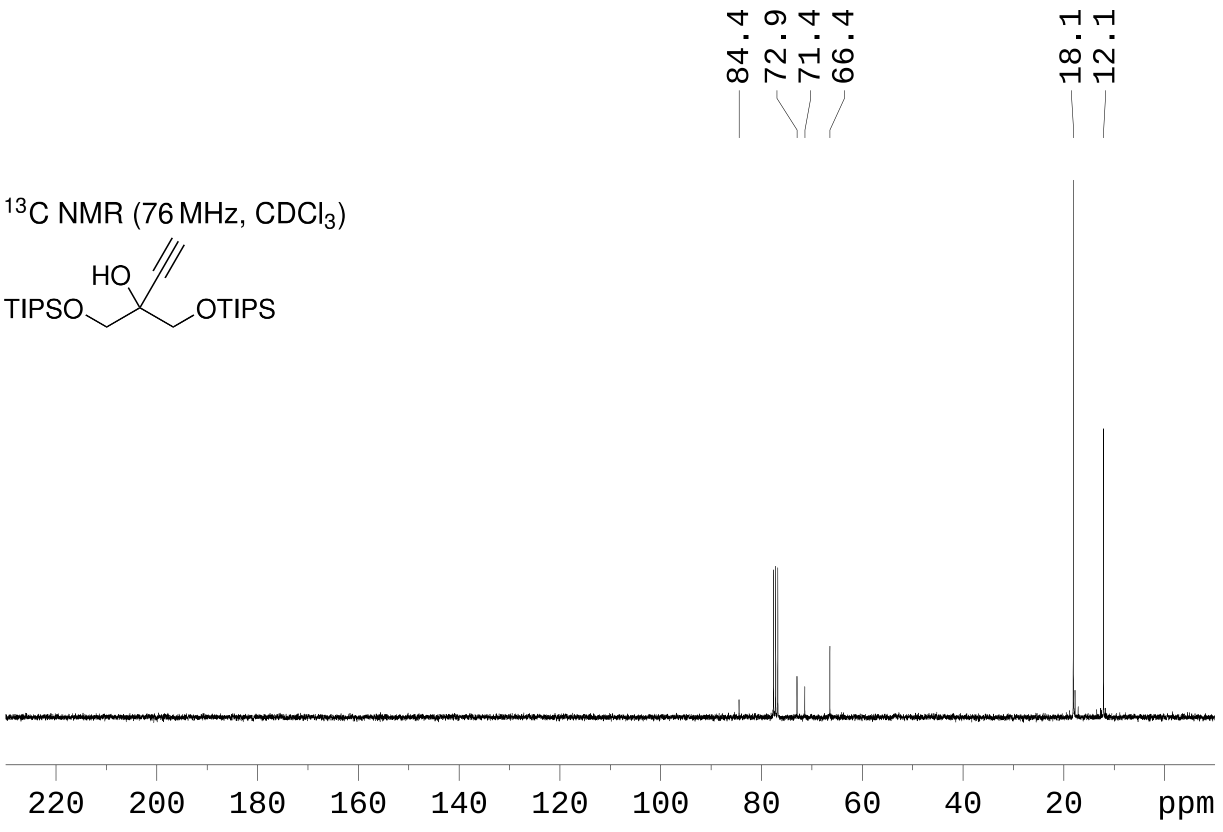

5.2 3,3,9,9-Tetraisopropyl-2,10-dimethyl-6-((1-(triisopropylsilyl)indolin-6-yl)-ethynyl)-4,8-dioxa-3,9-disilaundecan-6-yl acetate (9)

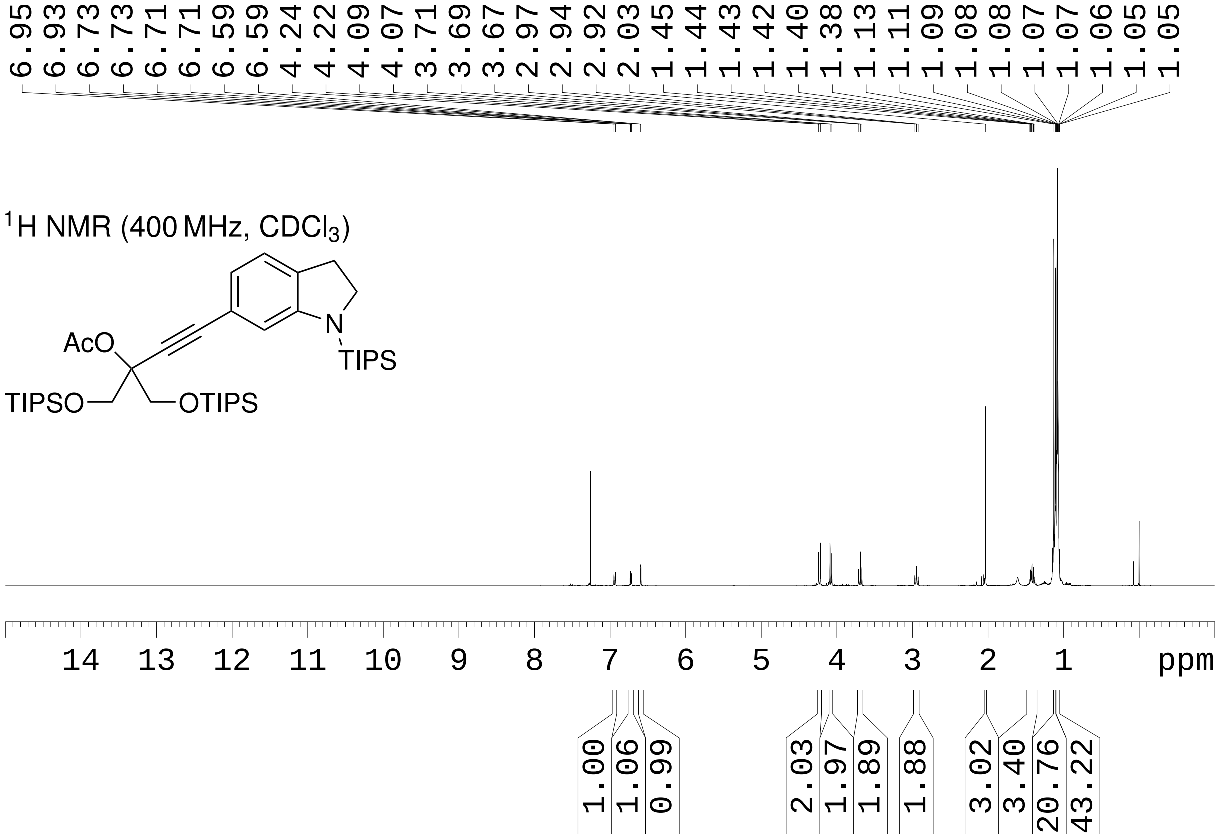

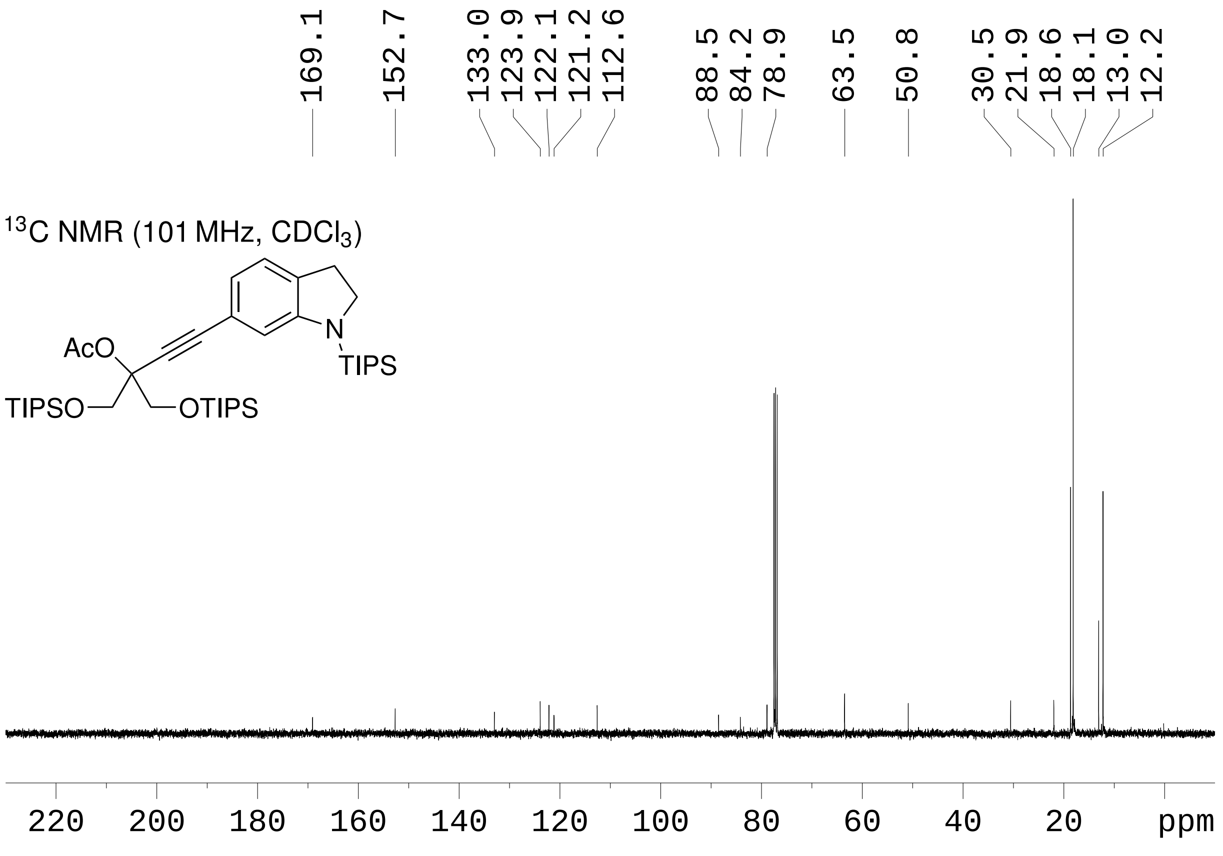

5.3 1-(Triisopropylsilyl)-5,5-bis(((triisopropylsilyl)oxy)methyl)-2,3,5,6-tetrahydrocyclopenta[*f*]indol-7(1*H*)-one (10)

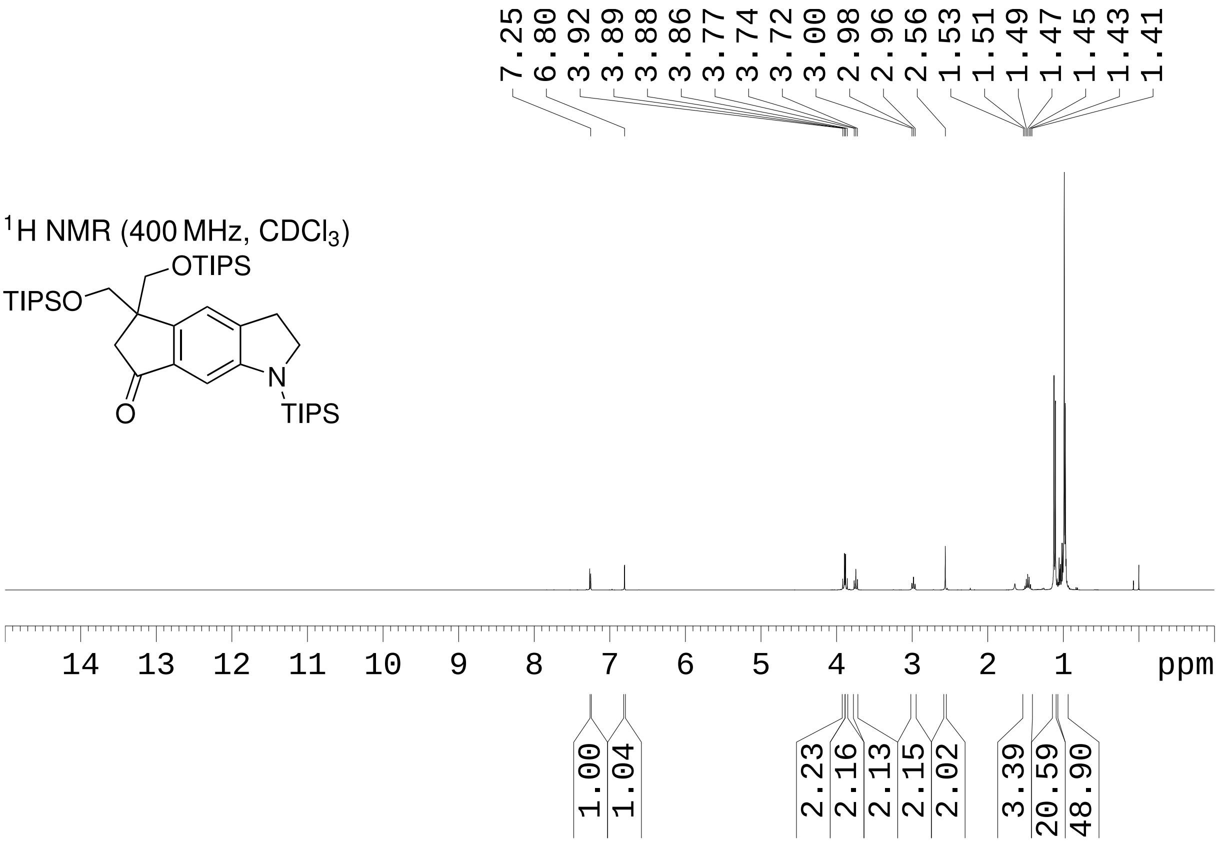

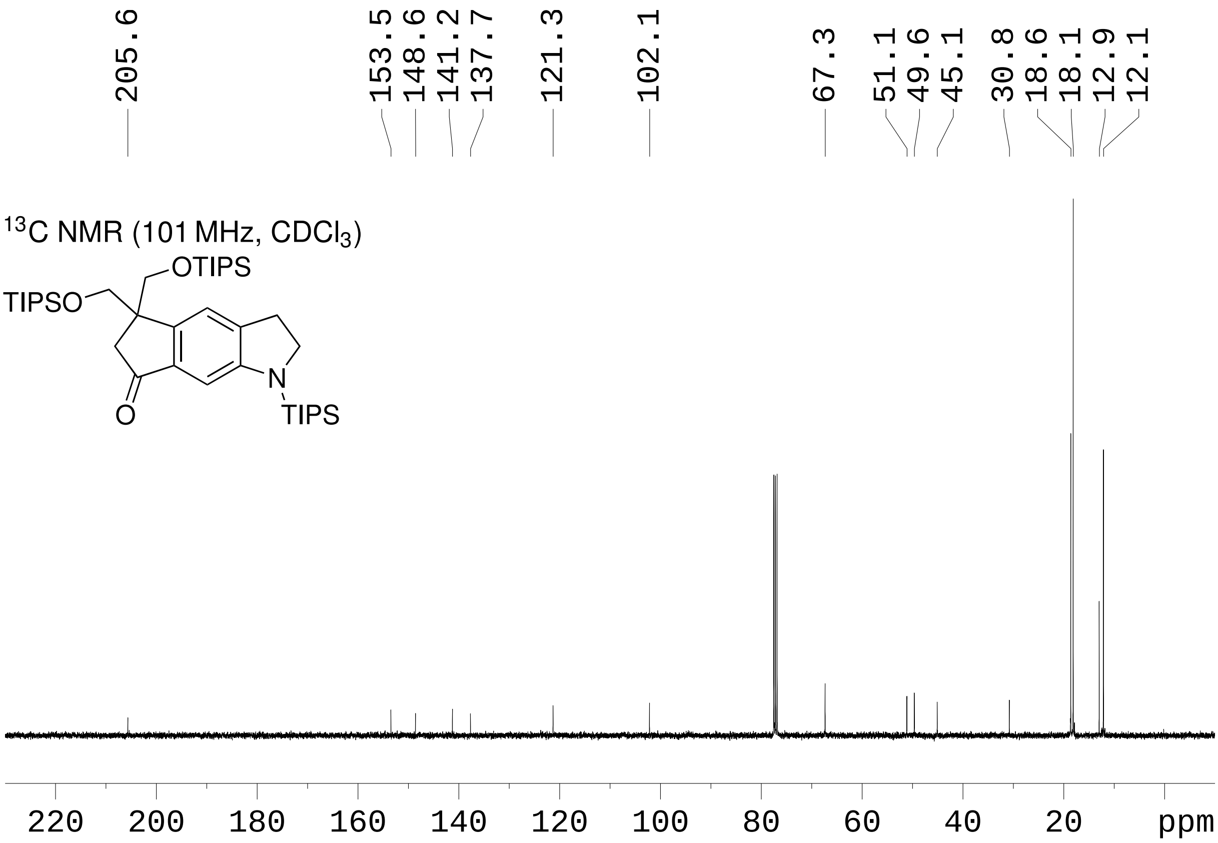

5.4 5,5-Bis(hydroxymethyl)-1-tosyl-1,2,3,5-tetrahydrocyclopenta[*f*]indol-7-yl trifluormethansulfonate (11)

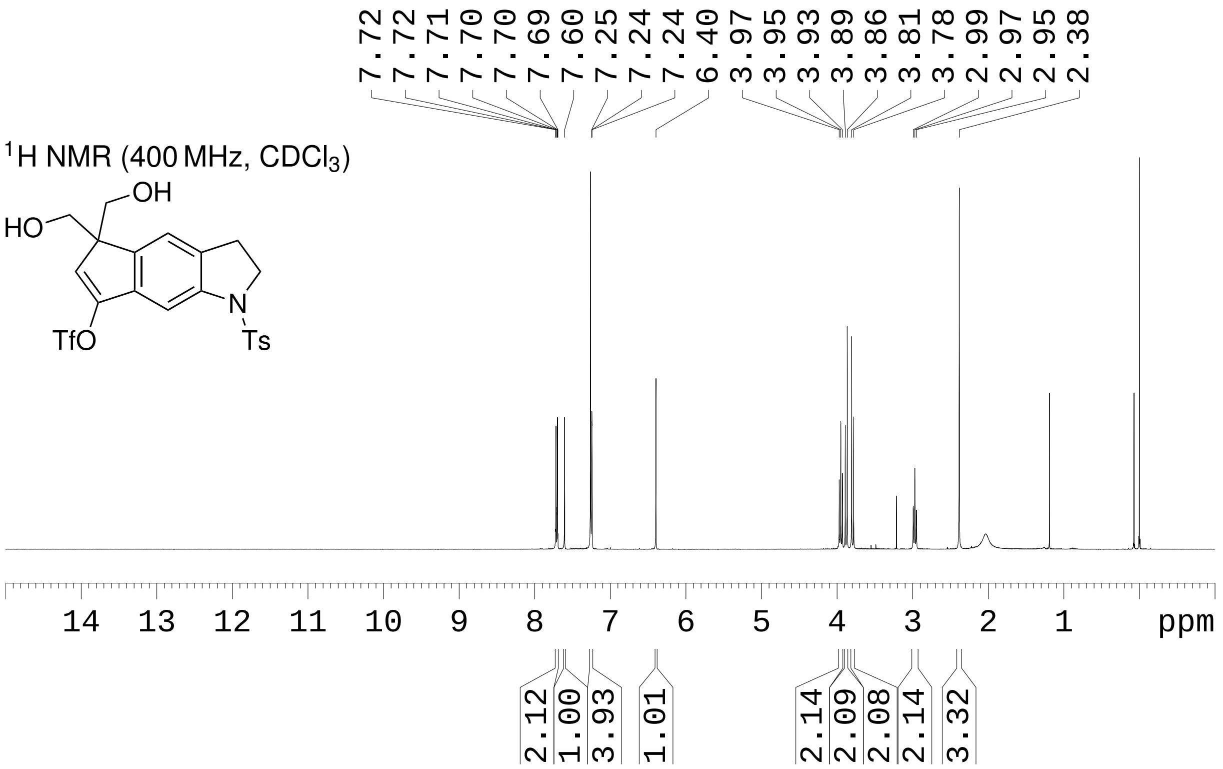

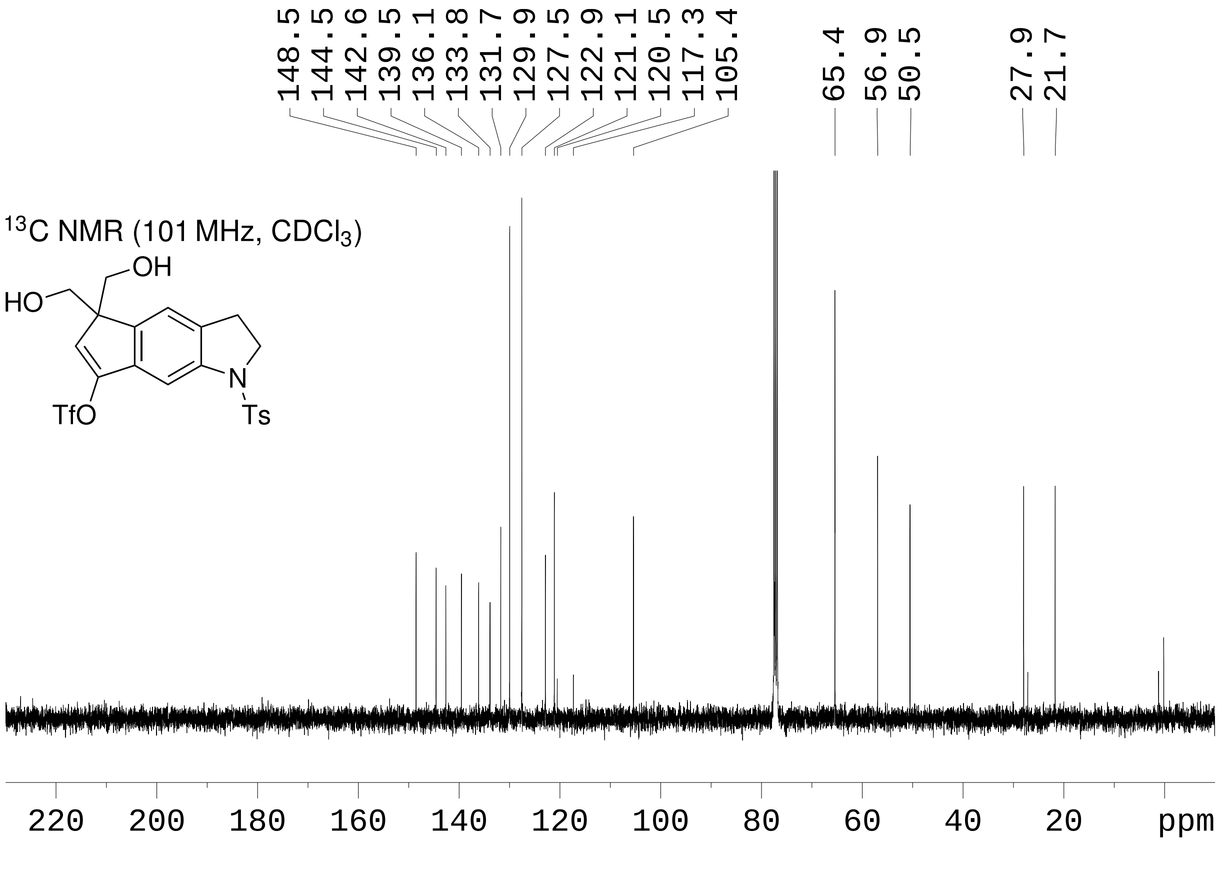

5 NMR spectra of new compounds

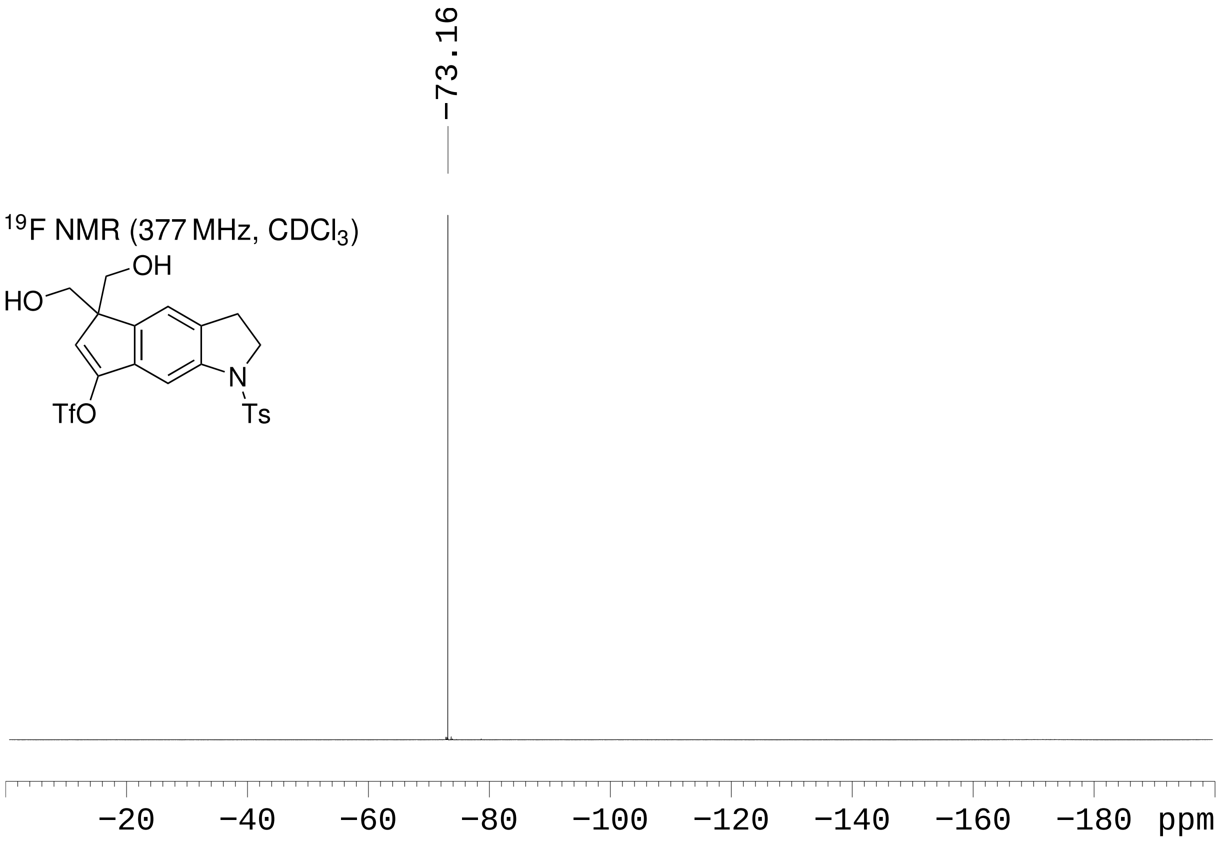

5.5 (7-(2-Methylprop-1-en-1-yl)-1-tosyl-1,2,3,5-tetrahydrocyclopenta[*f*]indole-5,5-diyl)dimethanol (14)

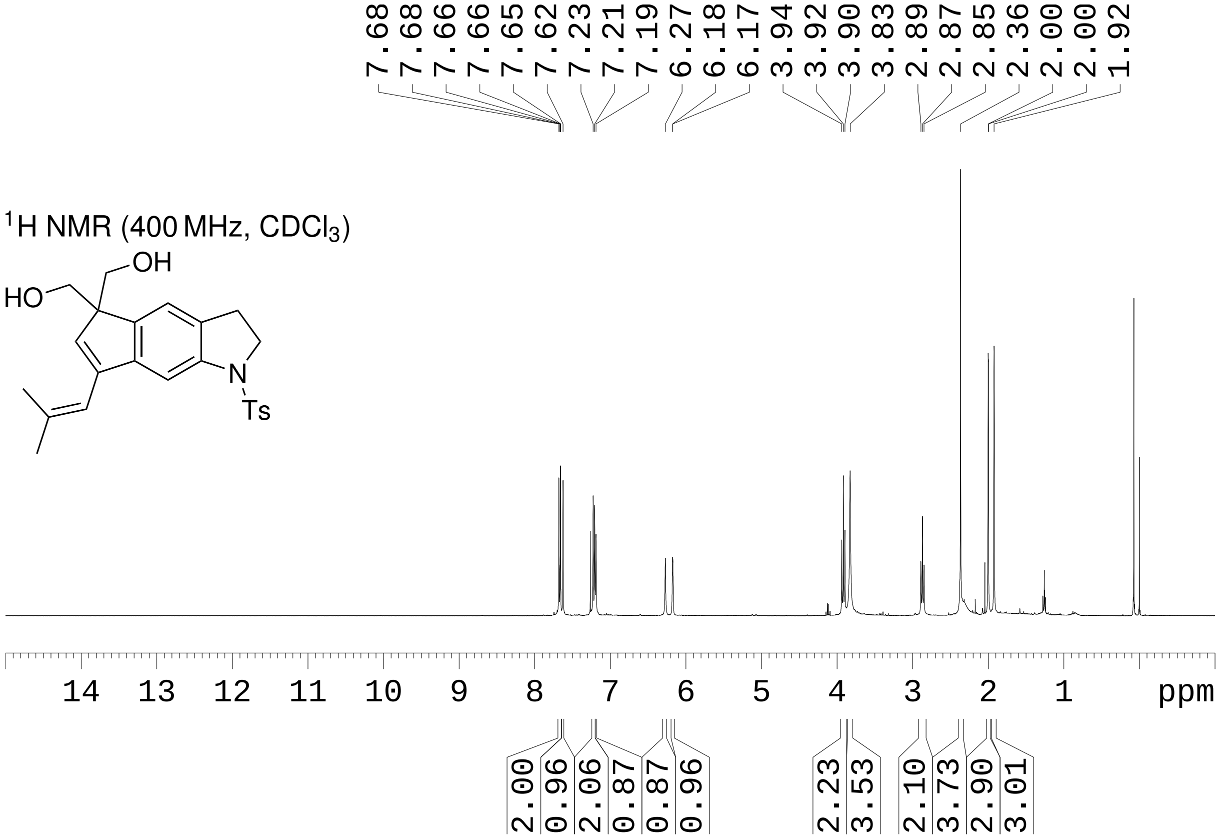

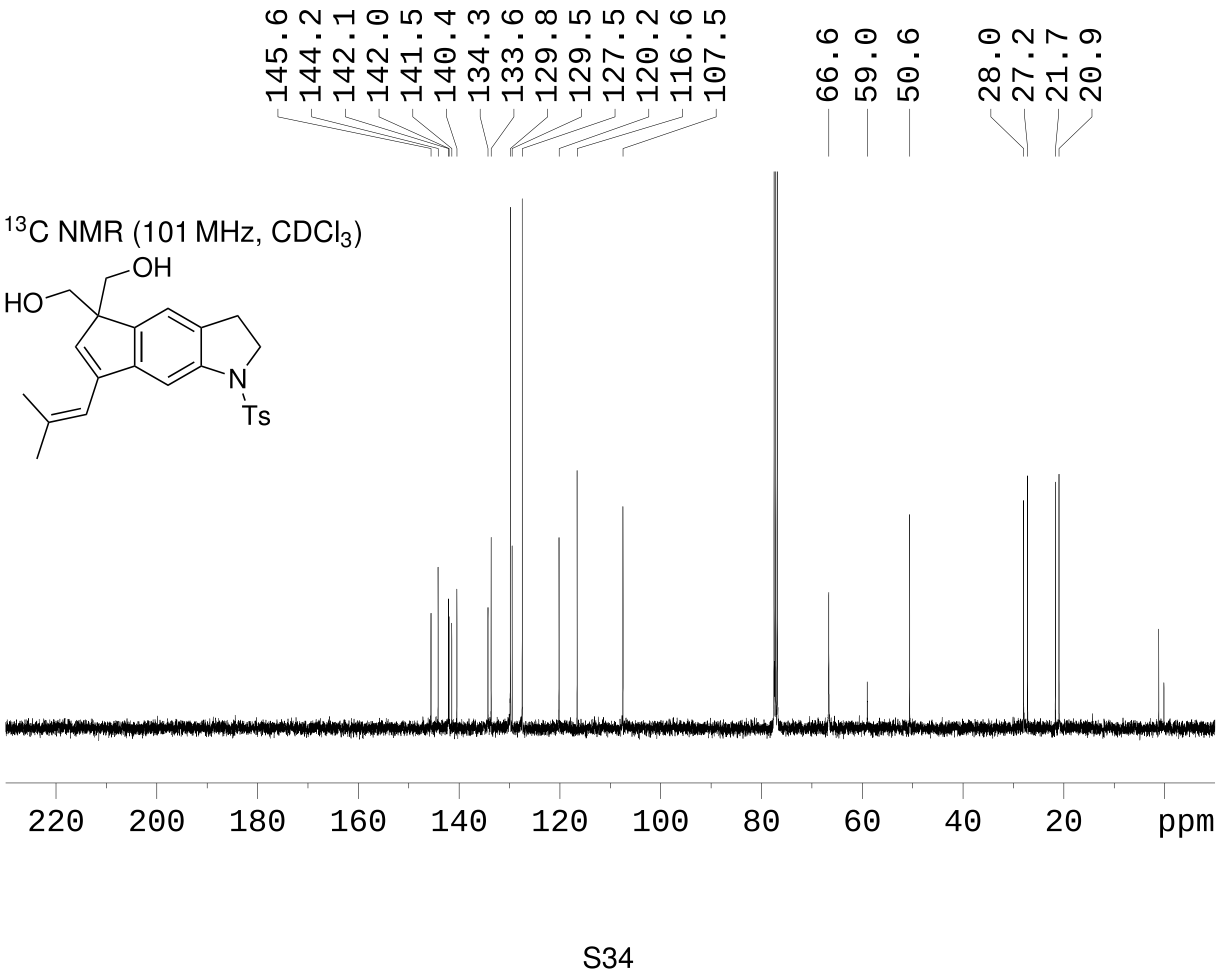

5.6 Dimer 20a

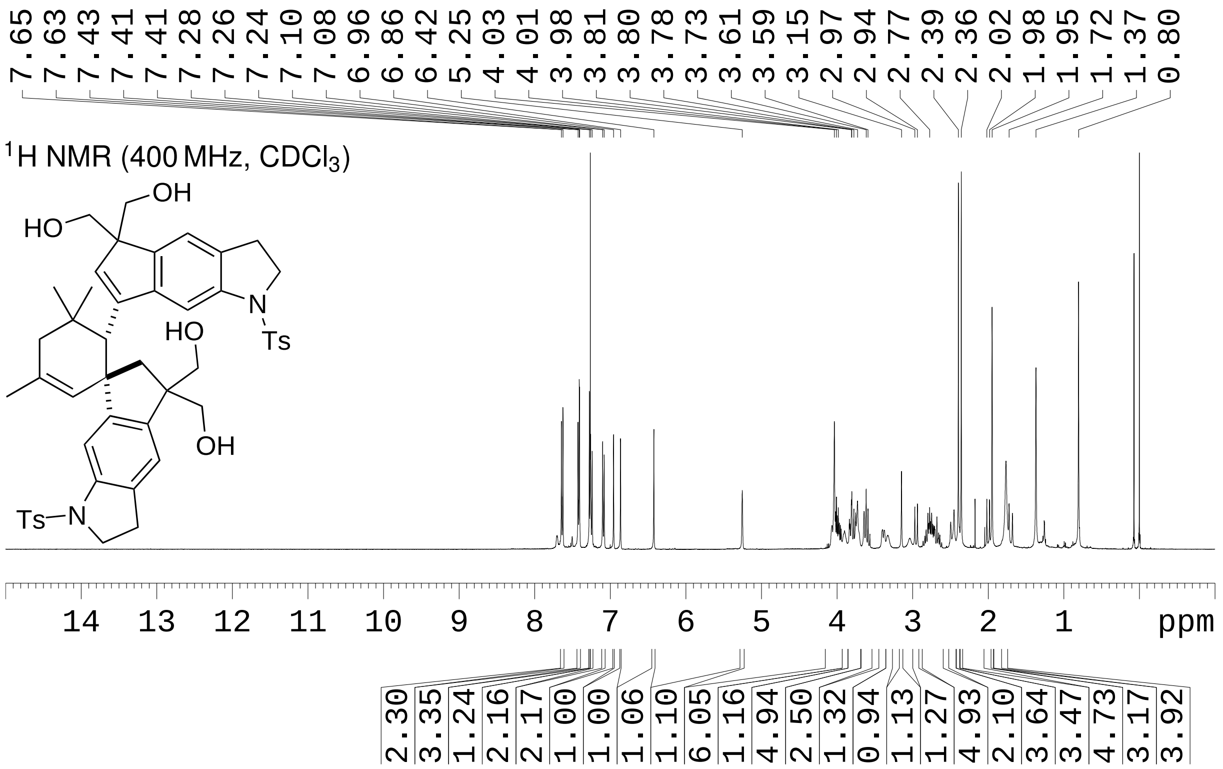
7.65 7.63 7.43 7.41 7.41 7.28 7.26 7.24 7.10 7.08 6.96 6.86 6.42 5.25 4.03 4.01 3.98 3.81 3.80 3.78 3.73 3.61 3.59 3.15 2.97 2.94 2.77 2.39 2.36 2.02 1.98 1.95 1.72 1.37 0.80
^{1}H NMR (400 MHz, CDCl$_3$)
HO
OH
HO
OH
N
Ts
Ts–N
14 13 12 11 10 9 8 7 6 5 4 3 2 1 ppm
2.30 3.35 1.24 2.16 2.17 1.00 1.00 1.06 1.10 6.05 1.16 4.94 2.50 1.32 0.94 1.13 1.27 4.93 2.10 3.64 3.47 4.73 3.17 3.92

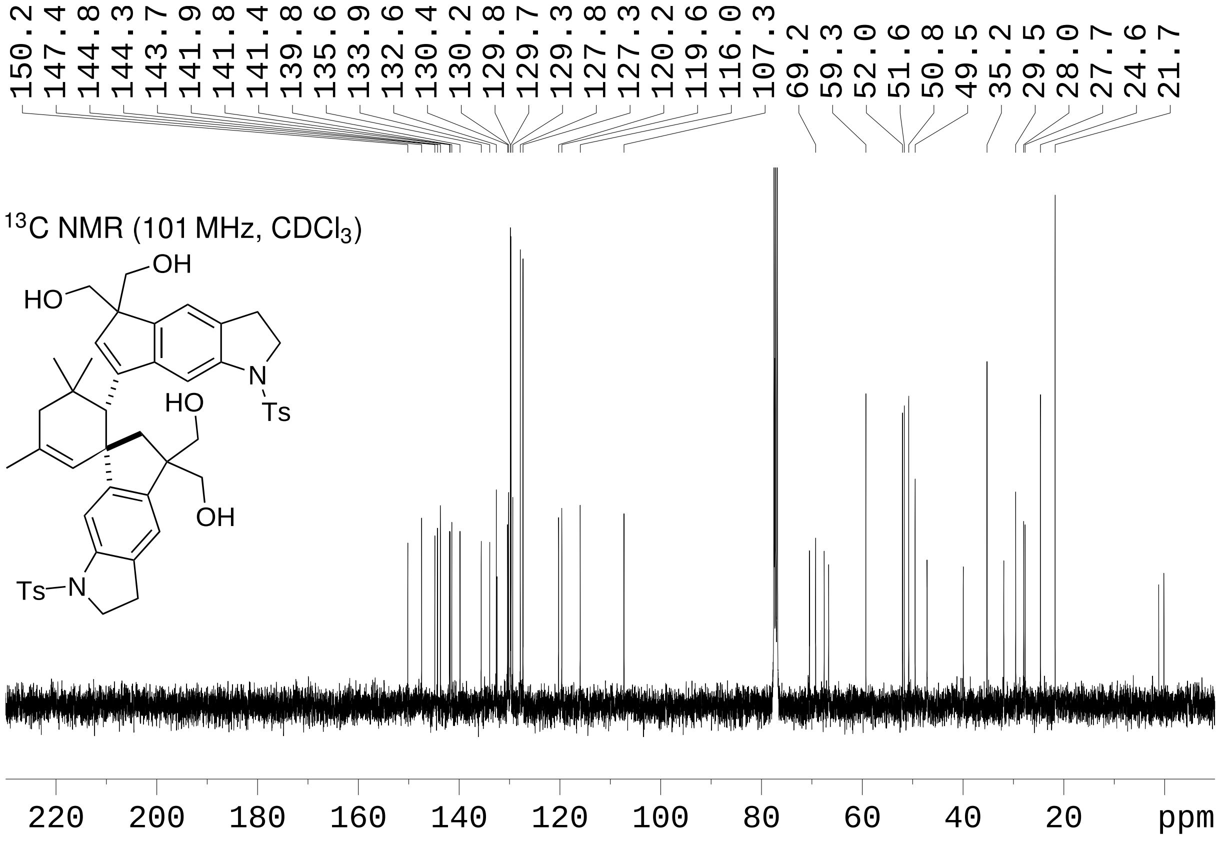
150.2 147.4 144.8 144.3 143.7 141.9 141.8 141.4 139.8 135.6 133.9 132.6 130.4 130.2 129.8 129.7 129.3 127.8 127.3 120.2 119.6 116.0 107.3 69.2 59.3 52.0 51.6 50.8 49.5 35.2 29.5 28.0 27.7 24.6 21.7
^{13}C NMR (101 MHz, CDCl$_3$)
HO
OH
HO
OH
N
Ts
Ts–N
220 200 180 160 140 120 100 80 60 40 20 ppm

5.7 Dimer 20b

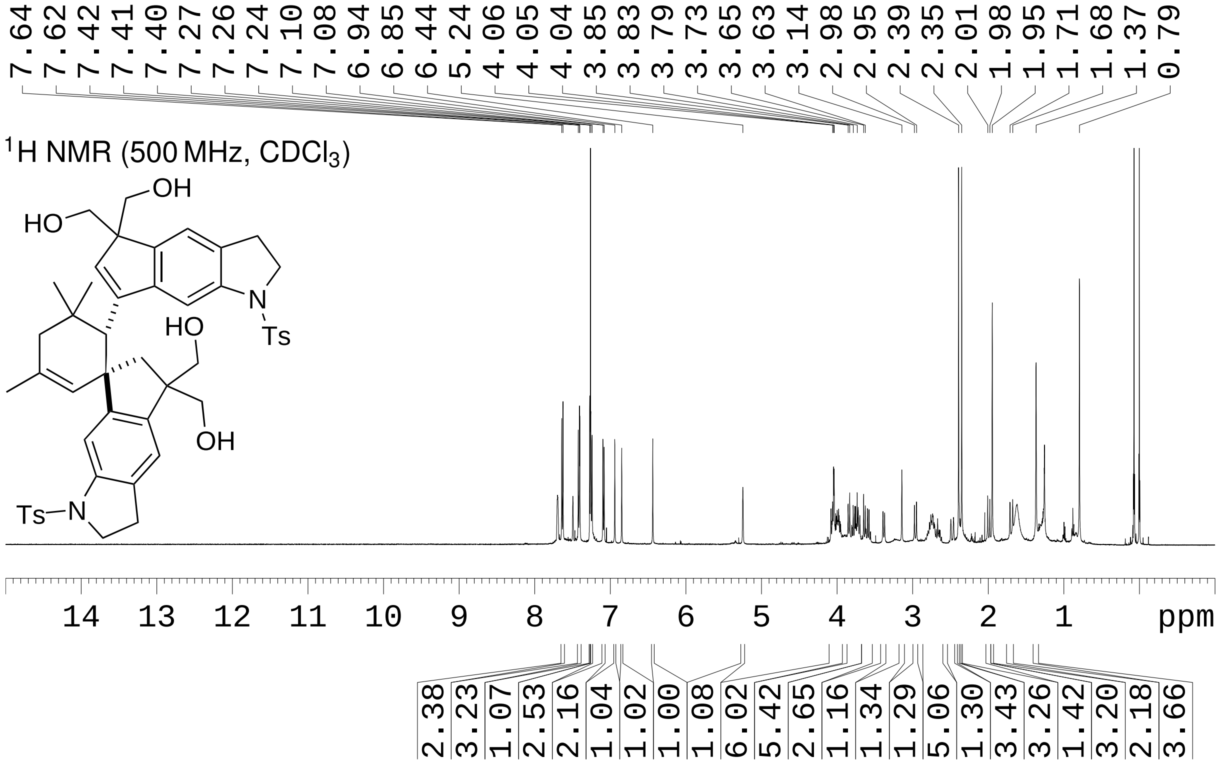
7.64 7.62 7.42 7.41 7.40 7.27 7.26 7.24 7.10 7.08 6.94 6.85 6.44 5.24 4.06 4.05 4.04 3.85 3.83 3.79 3.73 3.65 3.63 3.14 2.98 2.95 2.39 2.35 2.01 1.98 1.95 1.71 1.68 1.37 0.79
^{1}H NMR (500 MHz, CDCl$_3$)
OH
HO
N
Ts
HO
OH
Ts–N
14 13 12 11 10 9 8 7 6 5 4 3 2 1 ppm
2.38 3.23 1.07 2.53 2.16 1.04 1.02 1.00 1.08 6.02 5.42 2.65 1.16 1.34 1.29 5.06 1.30 3.43 3.26 1.42 3.20 2.18 3.66

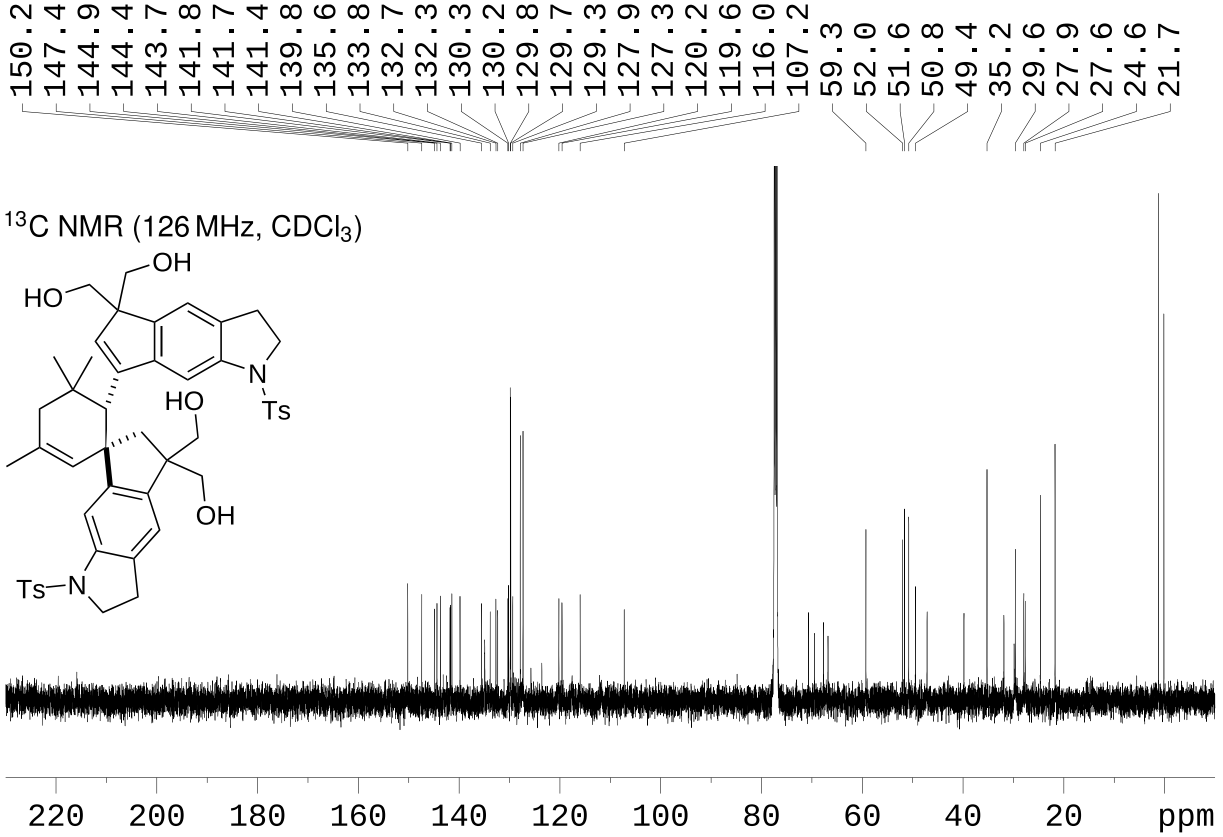
150.2 147.4 144.9 144.4 143.7 141.8 141.7 141.4 139.8 135.6 133.8 132.7 132.3 130.3 130.2 129.8 129.7 129.3 127.9 127.3 120.2 119.6 116.0 107.2 59.3 52.0 51.6 50.8 49.4 35.2 29.6 27.9 27.6 24.6 21.7
^{13}C NMR (126 MHz, CDCl$_3$)
OH
HO
N
Ts
HO
OH
Ts–N
220 200 180 160 140 120 100 80 60 40 20 ppm

5.8 (*S*)-(5-(Hydroxymethyl)-1-tosyl-7-(((trifluoromethyl)sulfonyl)oxy)-1,2,3,5-tetrahydrocyclopenta[*f*]indol-5-yl)methyl benzoate (13)

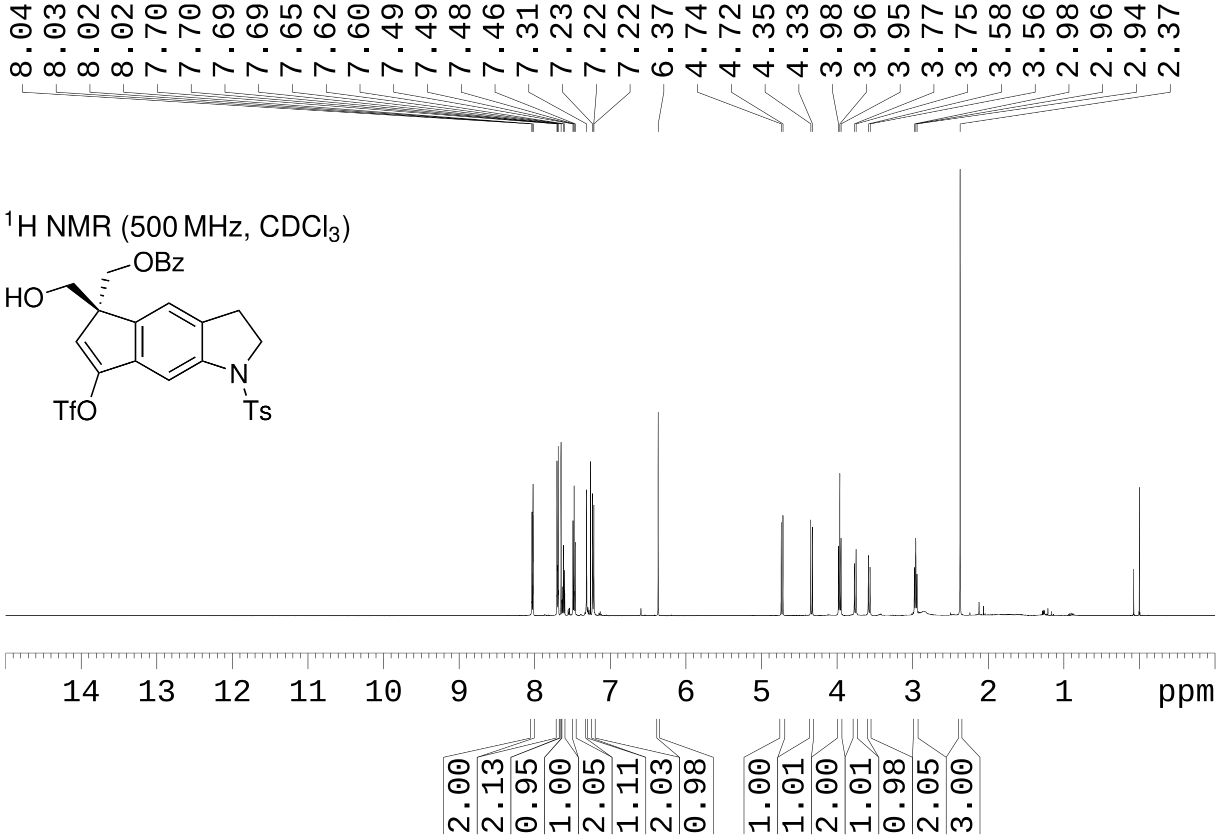

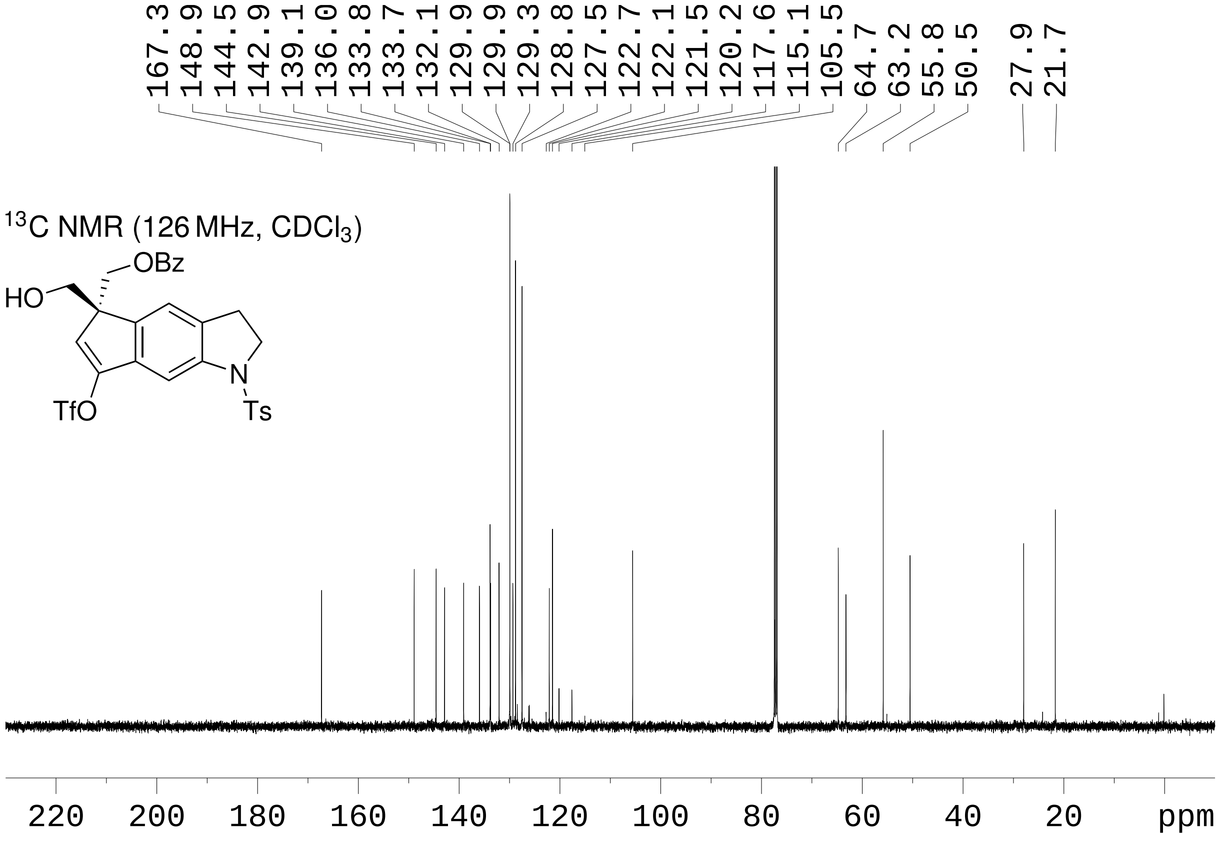

5 NMR spectra of new compounds

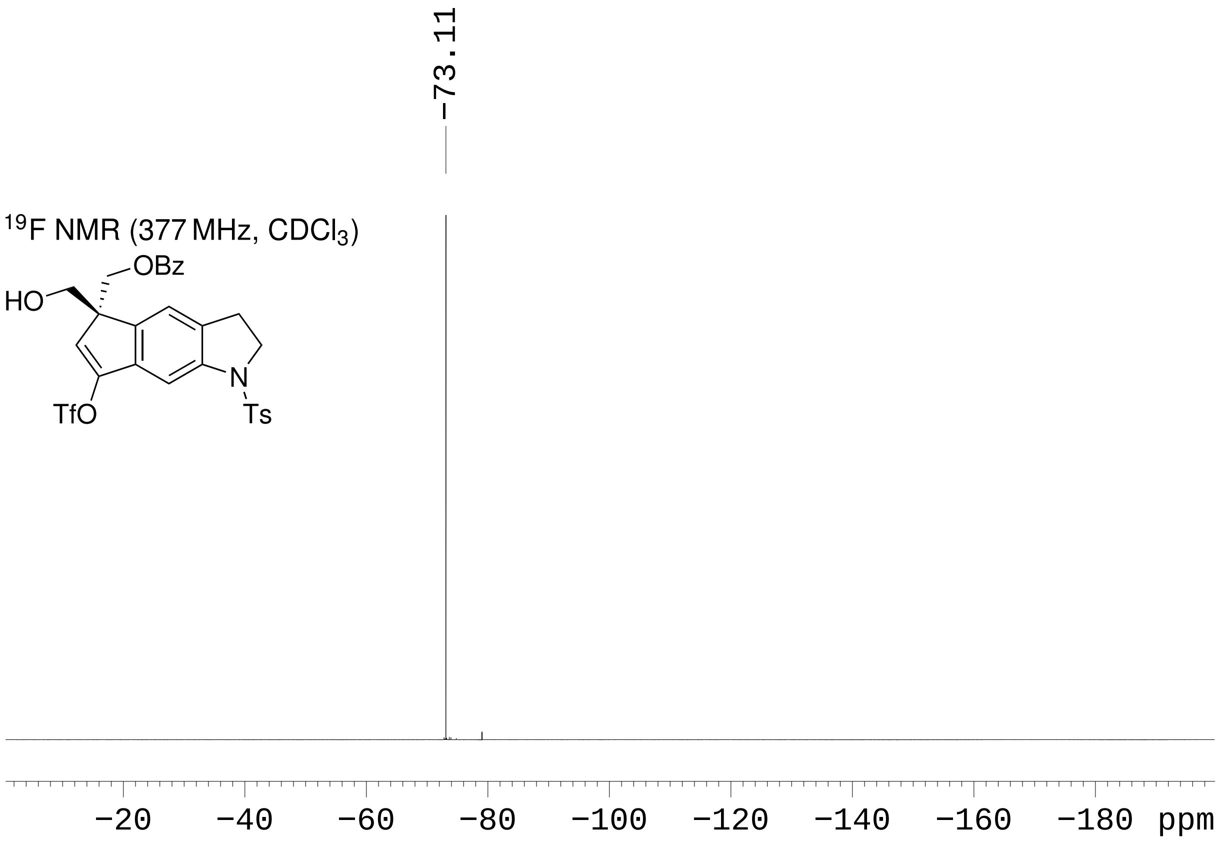

5.9 (*S*)-(5-(Hydroxymethyl)-7-(2-methylprop-1-en-1-yl)-1-tosyl-1,2,3,5-tetrahydro-cyclopenta[*f*]indol-5-yl)methyl benzoate (15)

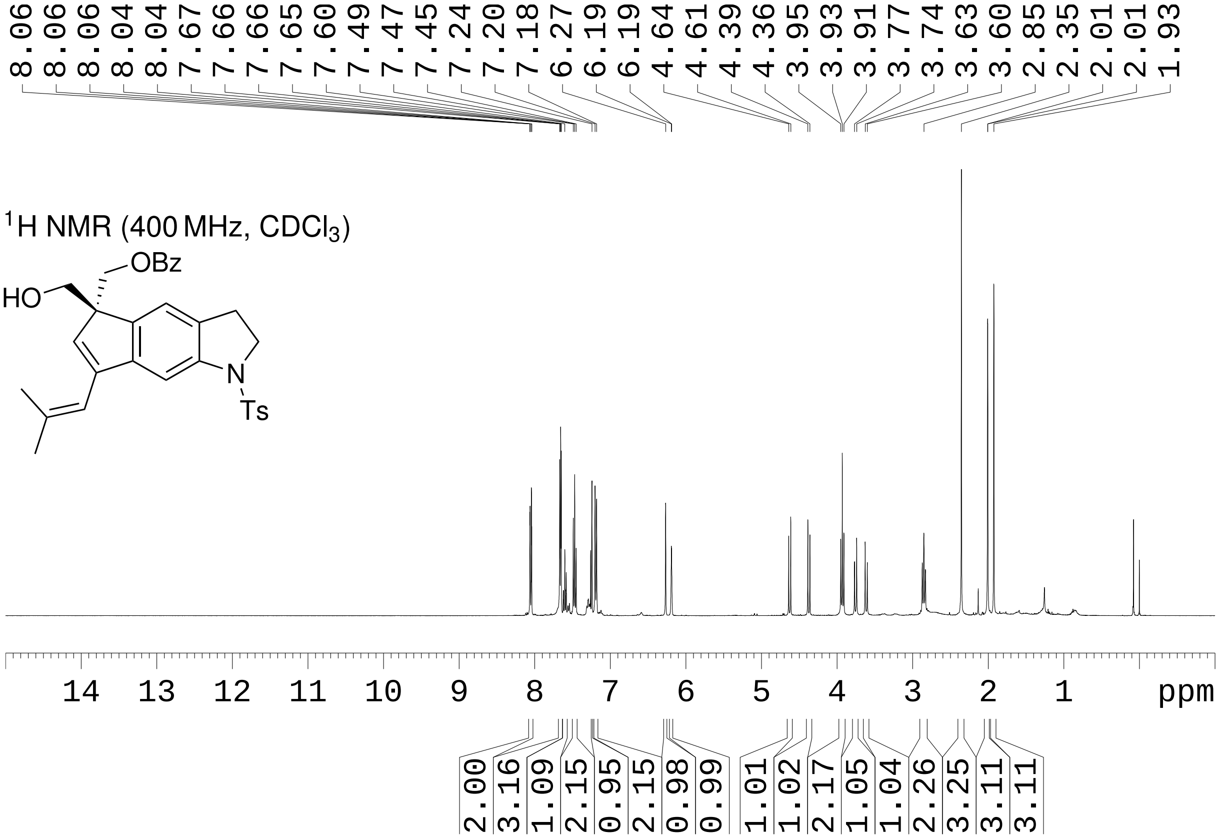

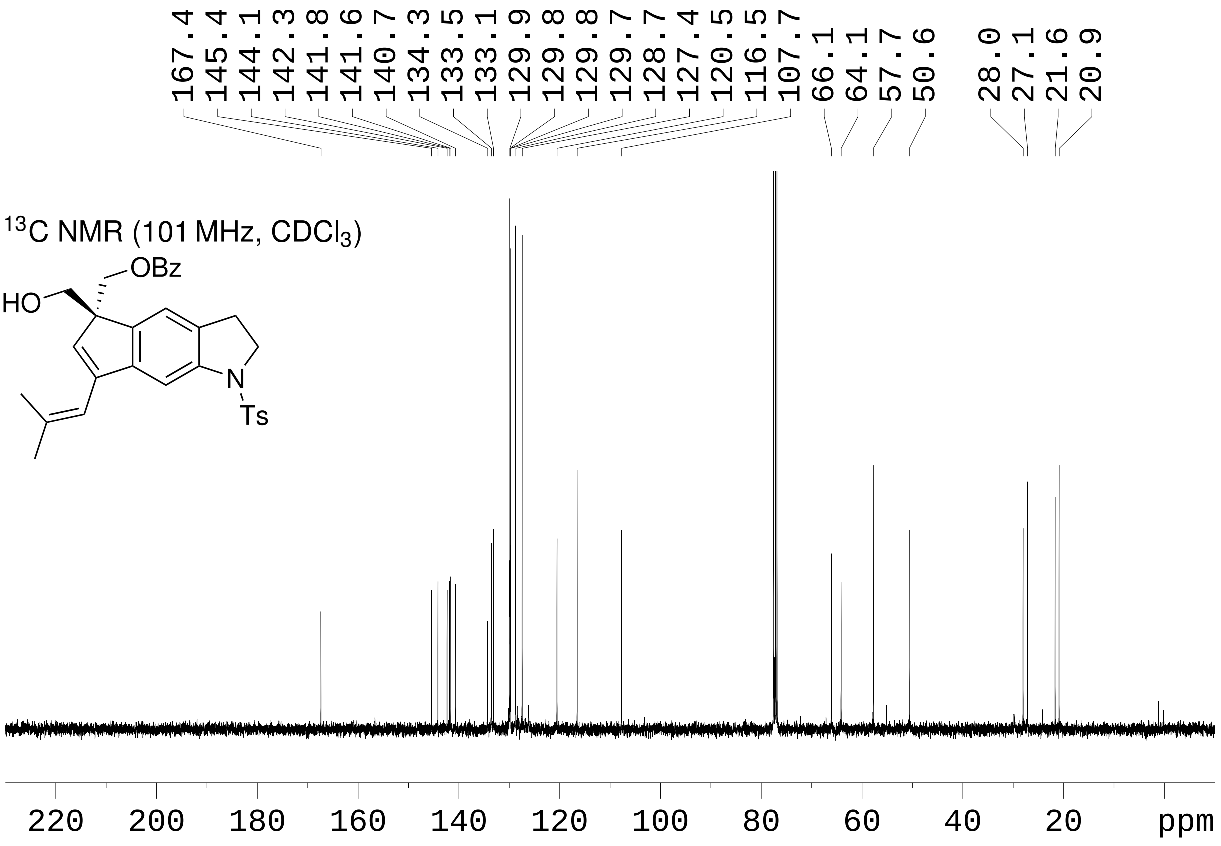

5.10 ((5*S*,7*R*)-5-(Hydroxymethyl)-7-isobutyl-1-tosyl-1,2,3,5,6,7-hexahydrocyclopenta[*f*]indol-5-yl)methyl benzoate (17)

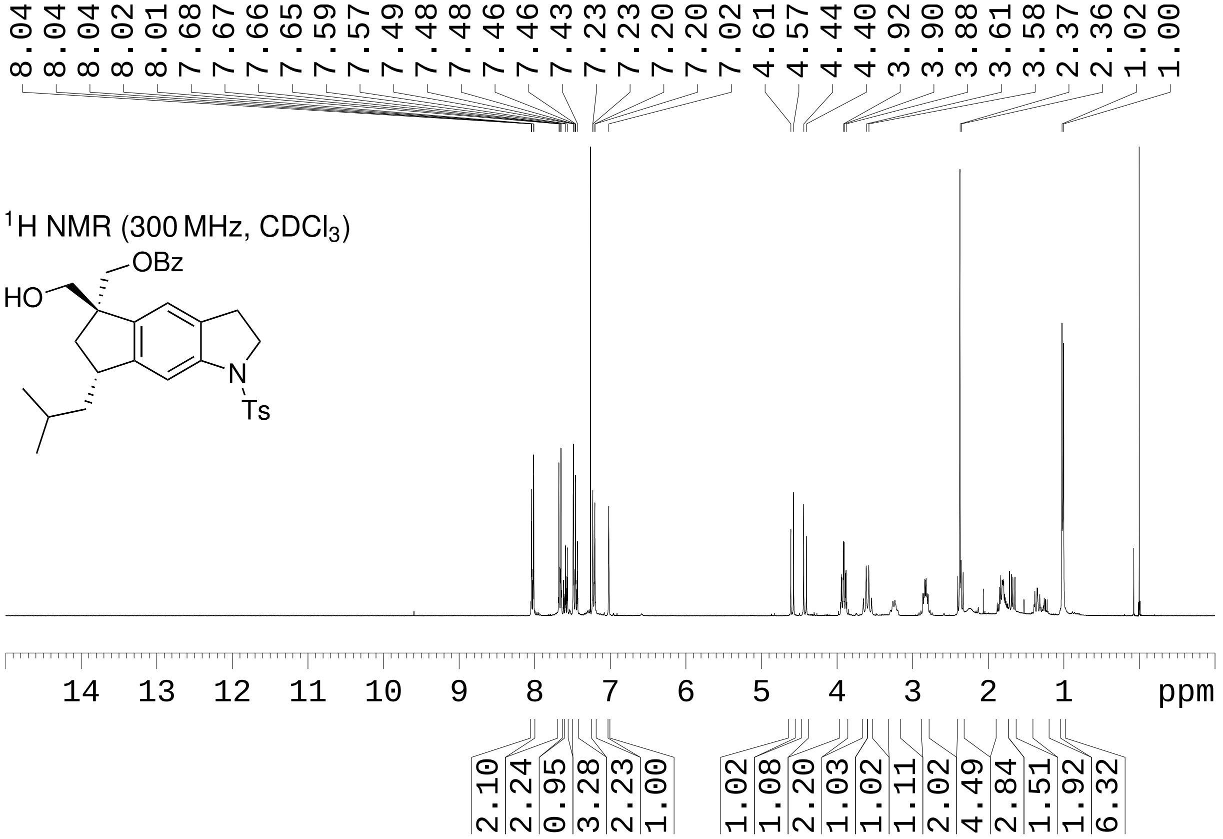

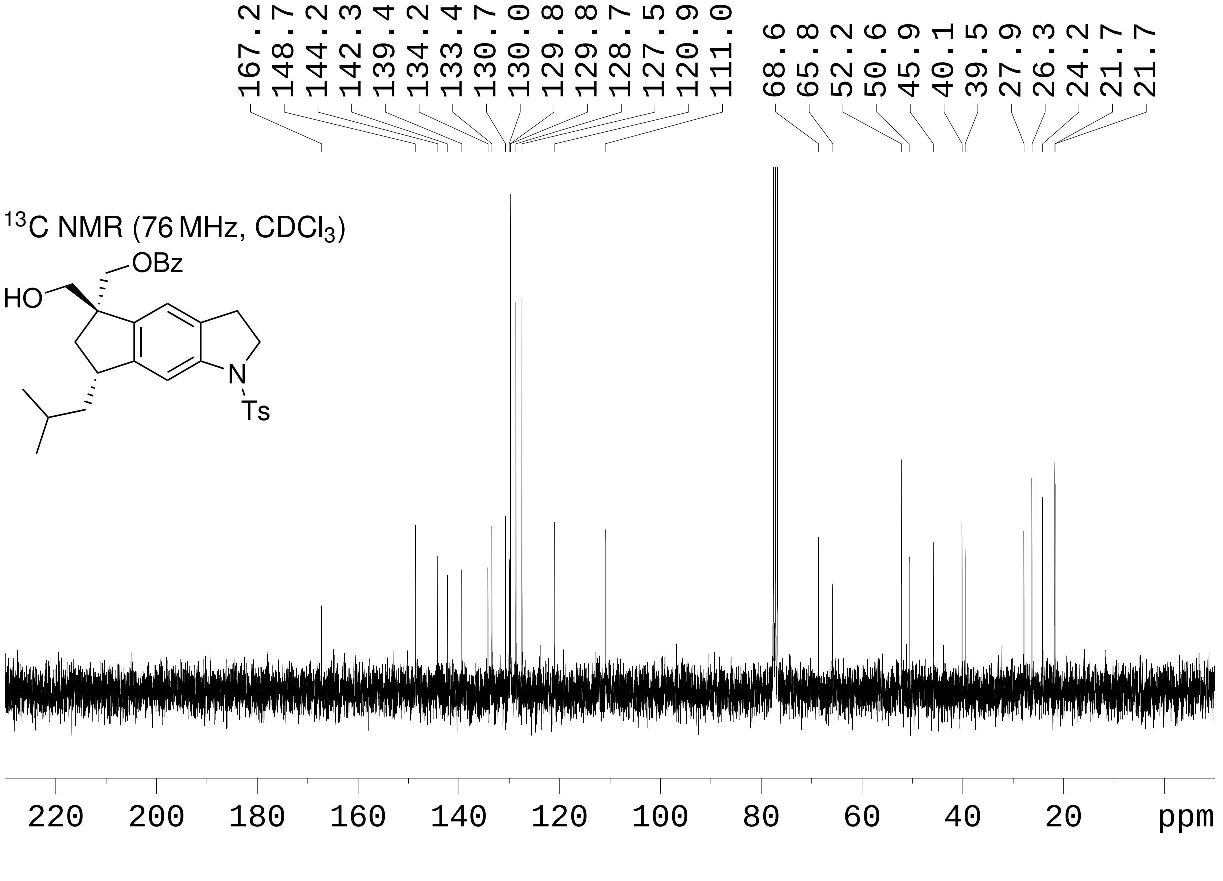

5.11 ((5*R*,7*R*)-5-Formyl-7-isobutyl-1-tosyl-1,2,3,5,6,7-hexahydrocyclopenta[*f*]indol-5-yl)methyl benzoate (S1)

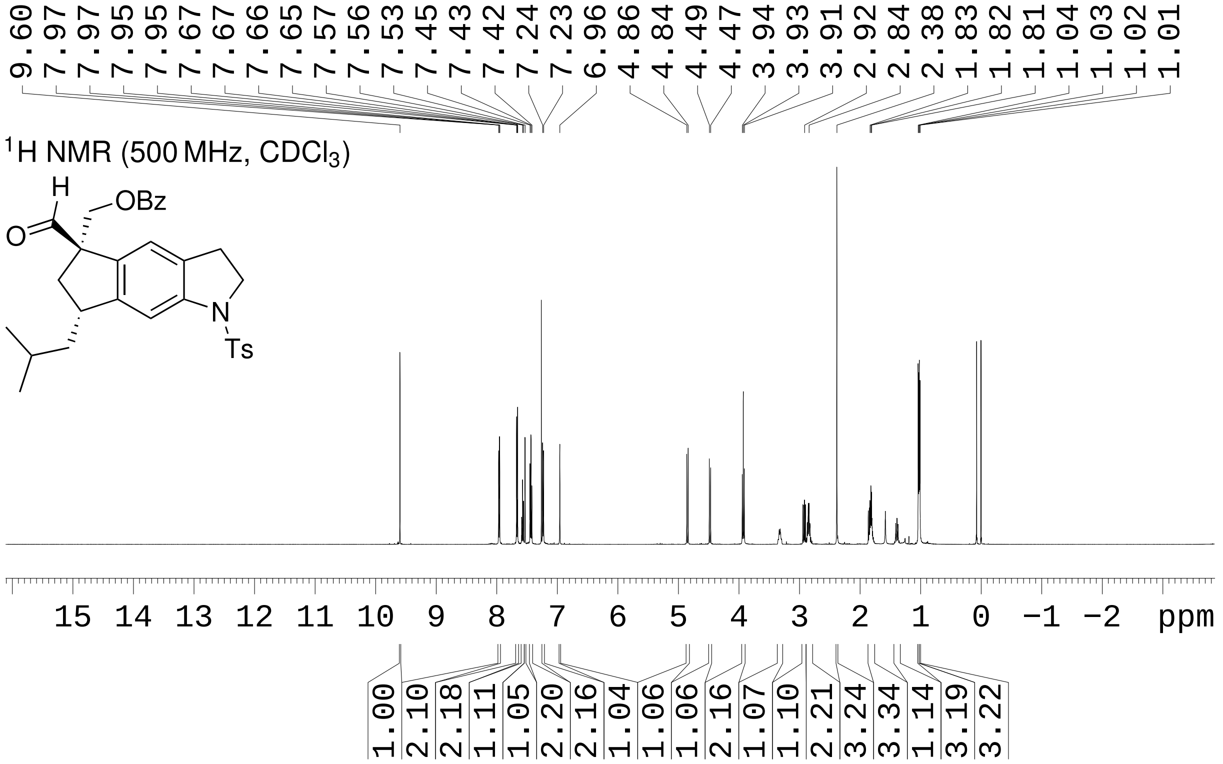

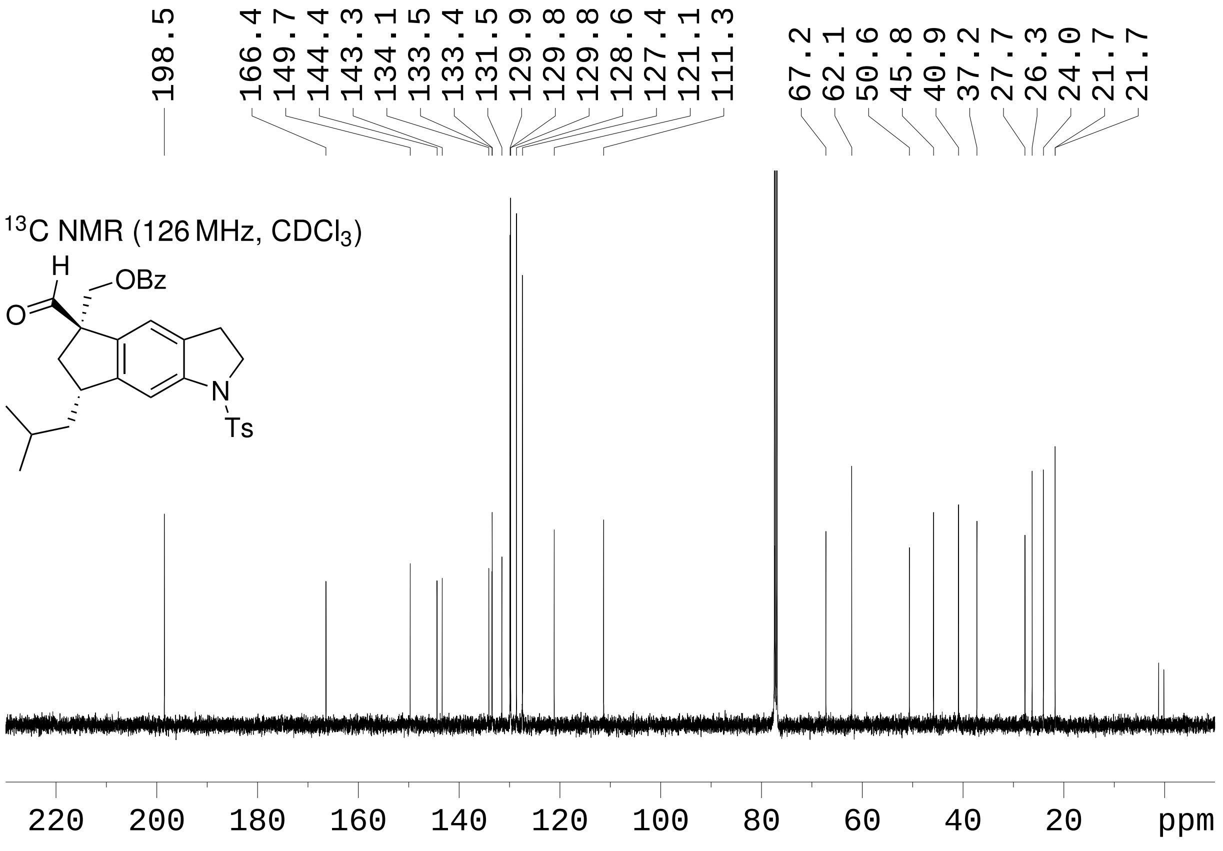

5.12 ((5*R*,7*R*)-5-((*E*)-2-Iodovinyl)-7-isobutyl-1-tosyl-1,2,3,5,6,7-hexahydrocyclopenta[*f*]indol-5-yl)methyl benzoate (21)

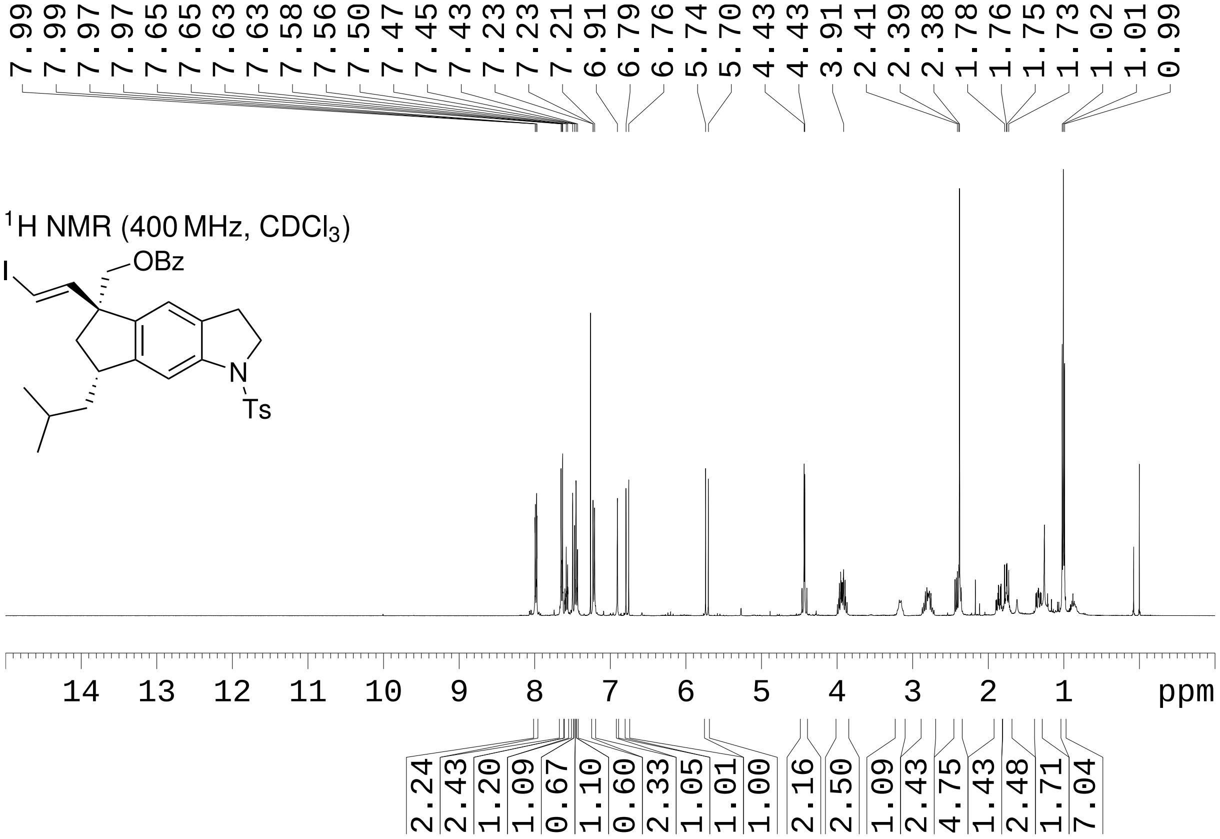

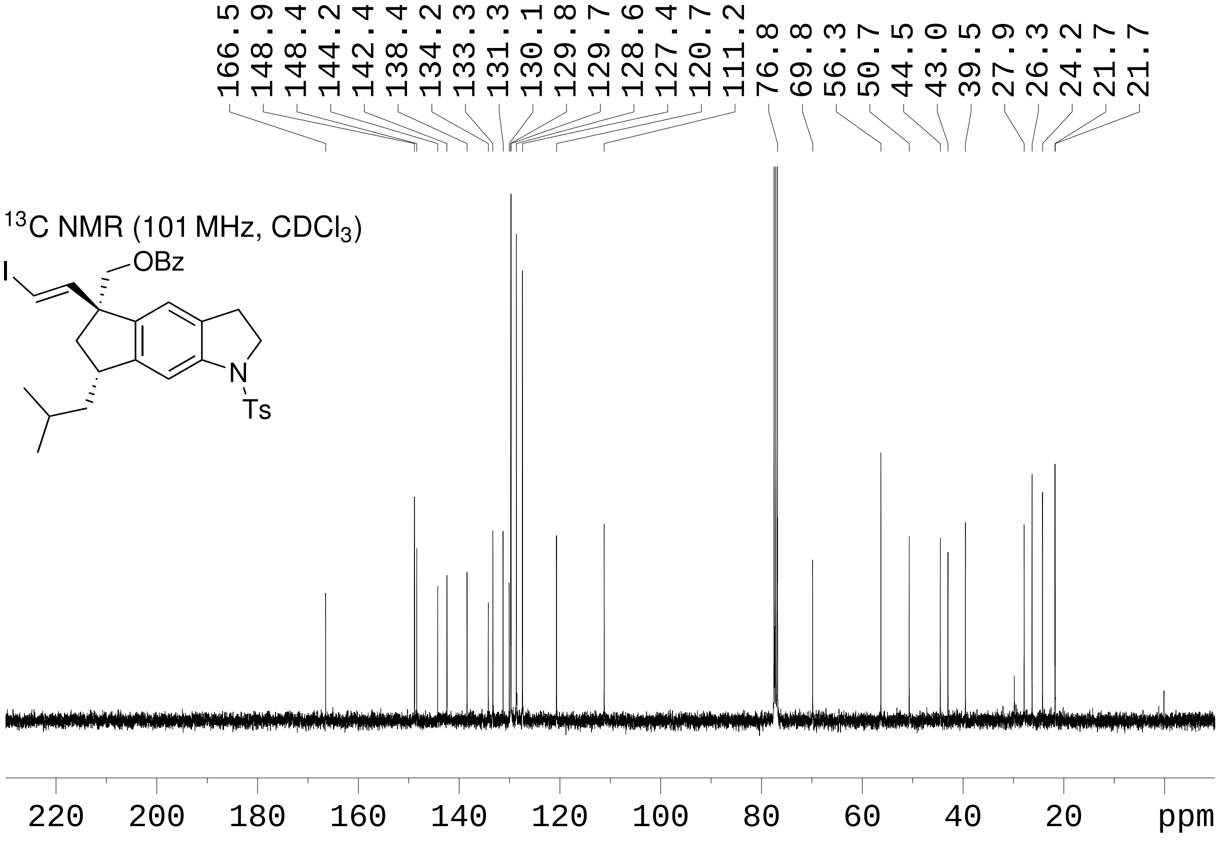

5.13 ((5*R*,7*R*)-5-((*E*)-2-(1*H*-Indol-5-yl)vinyl)-7-isobutyl-1-tosyl-1,2,3,5,6,7-hexahydrocyclopenta[*f*]indol-5-yl)methyl benzoate (23)

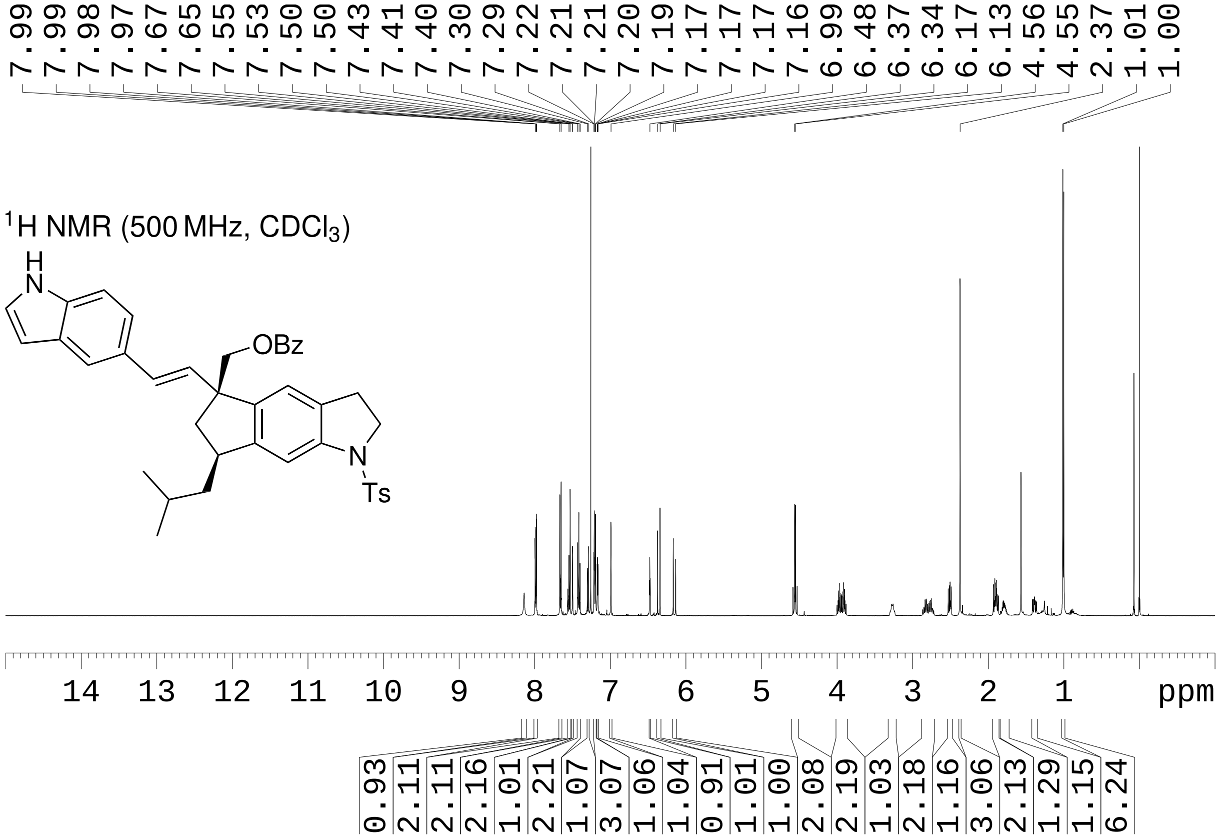

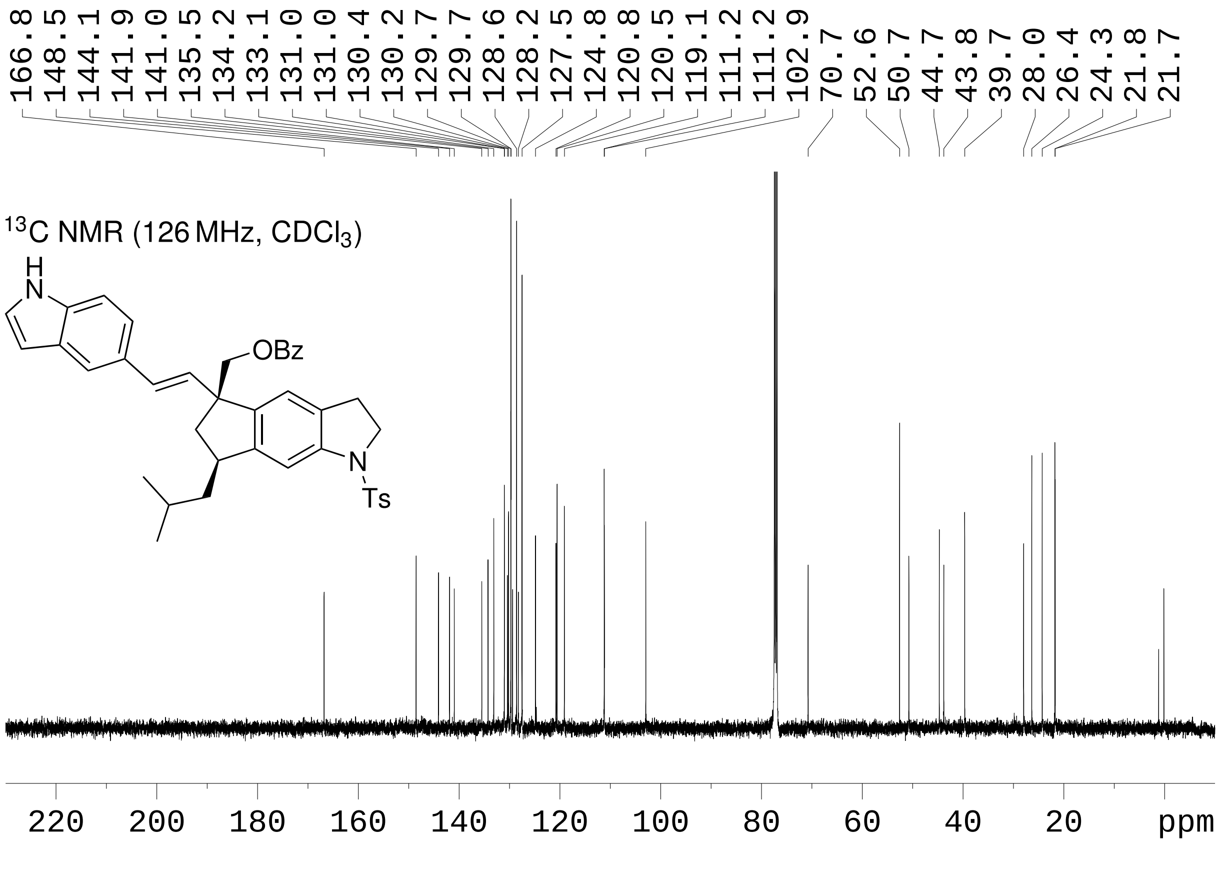

5.14 ((5*R*,7*R*)-5-((*E*)-2-(1*H*-Indol-5-yl)vinyl)-7-isobutyl-1-tosyl-1,2,3,5,6,7-hexahydrocyclopenta[*f*]indol-5-yl)methanol (S2)

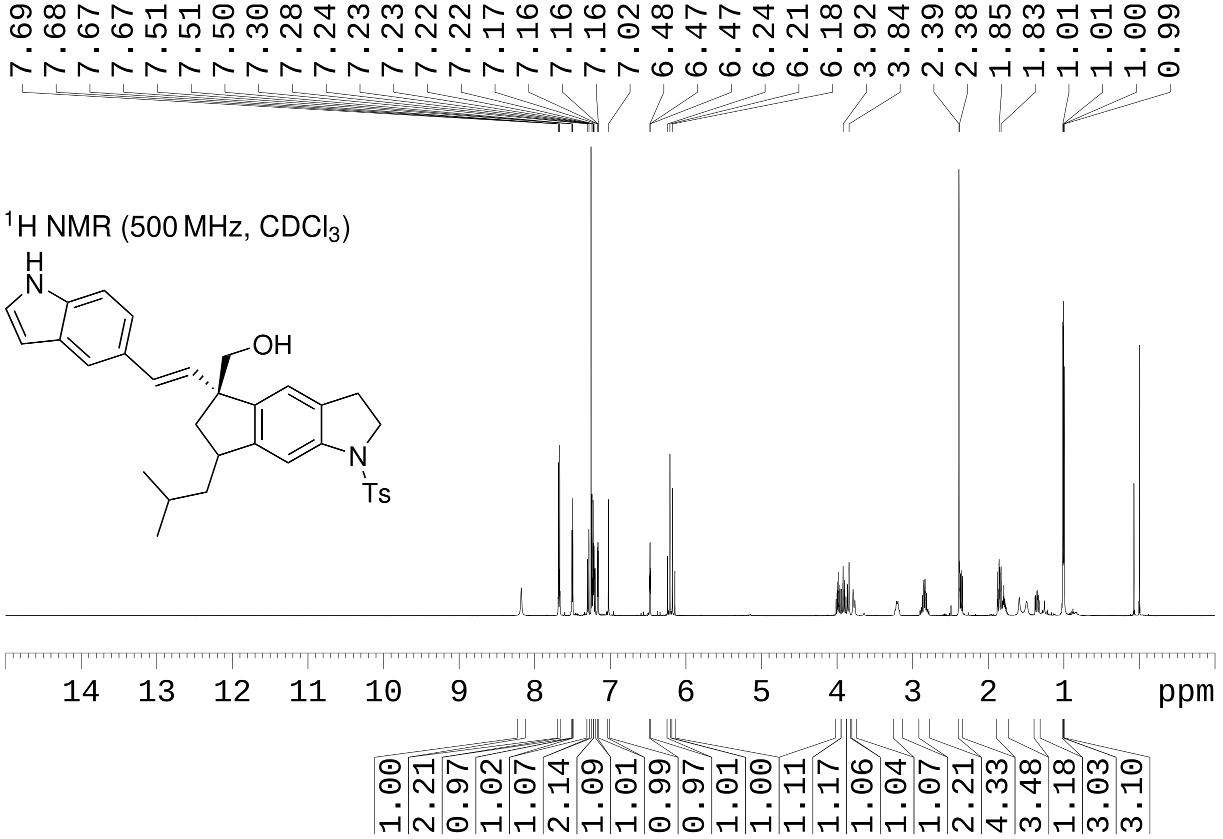

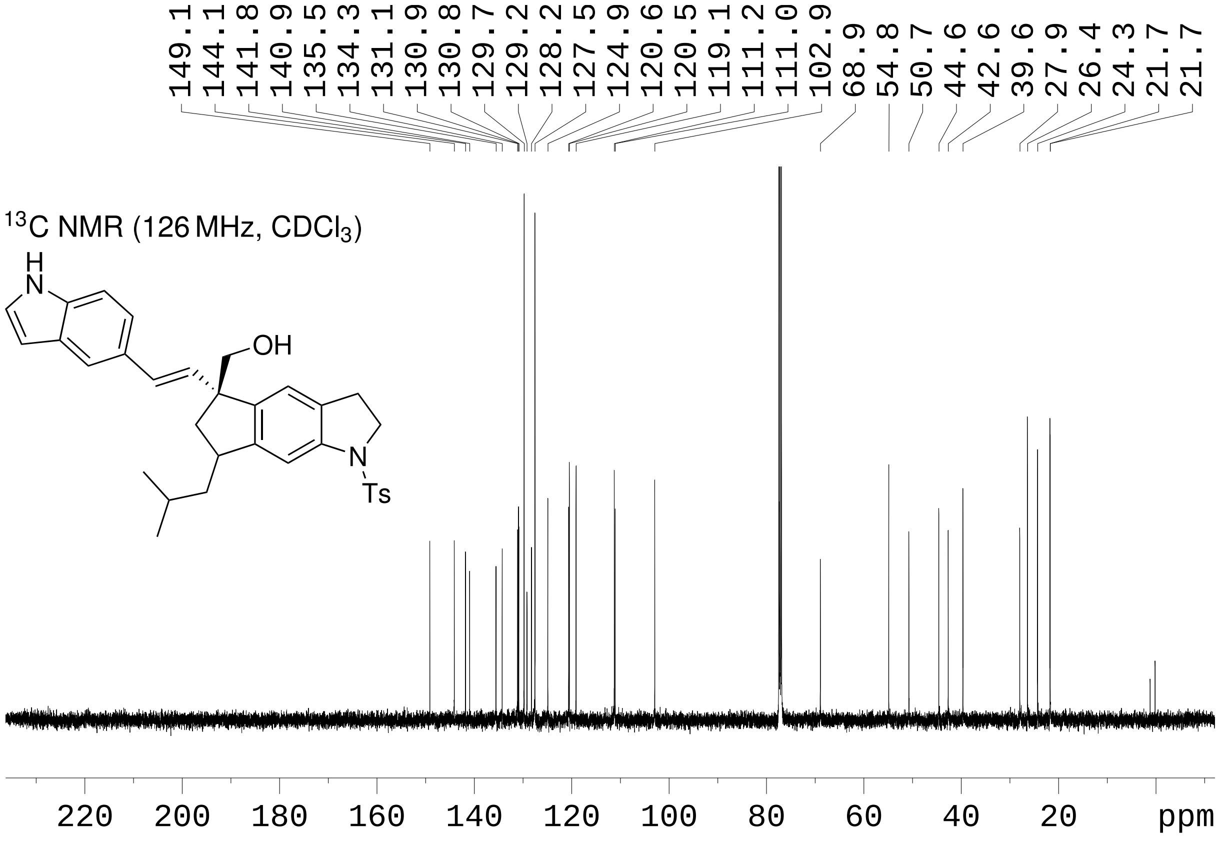

5.15 ((5*R*,7*R*)-5-((*E*)-2-(1*H*-indol-5-yl)vinyl)-7-isobutyl-1,2,3,5,6,7-hexahydro-cyclopenta[*f*]indol-5-yl)methanol (S3)

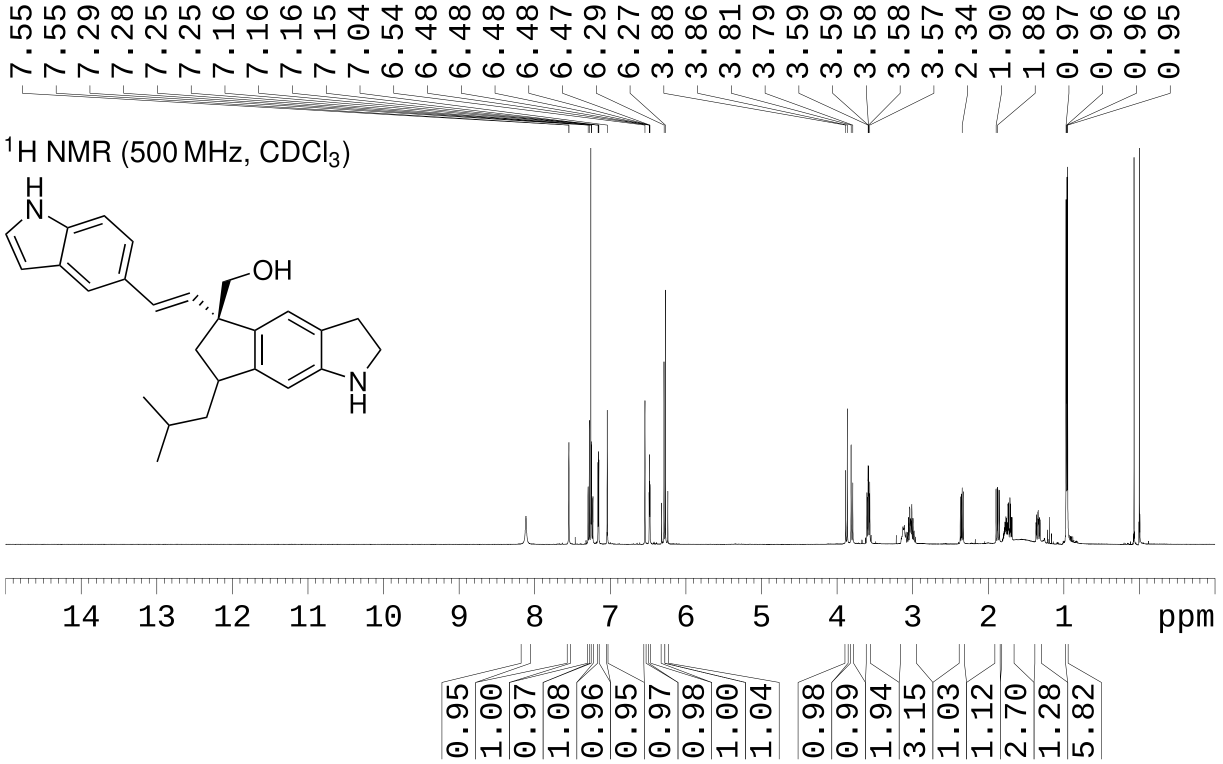

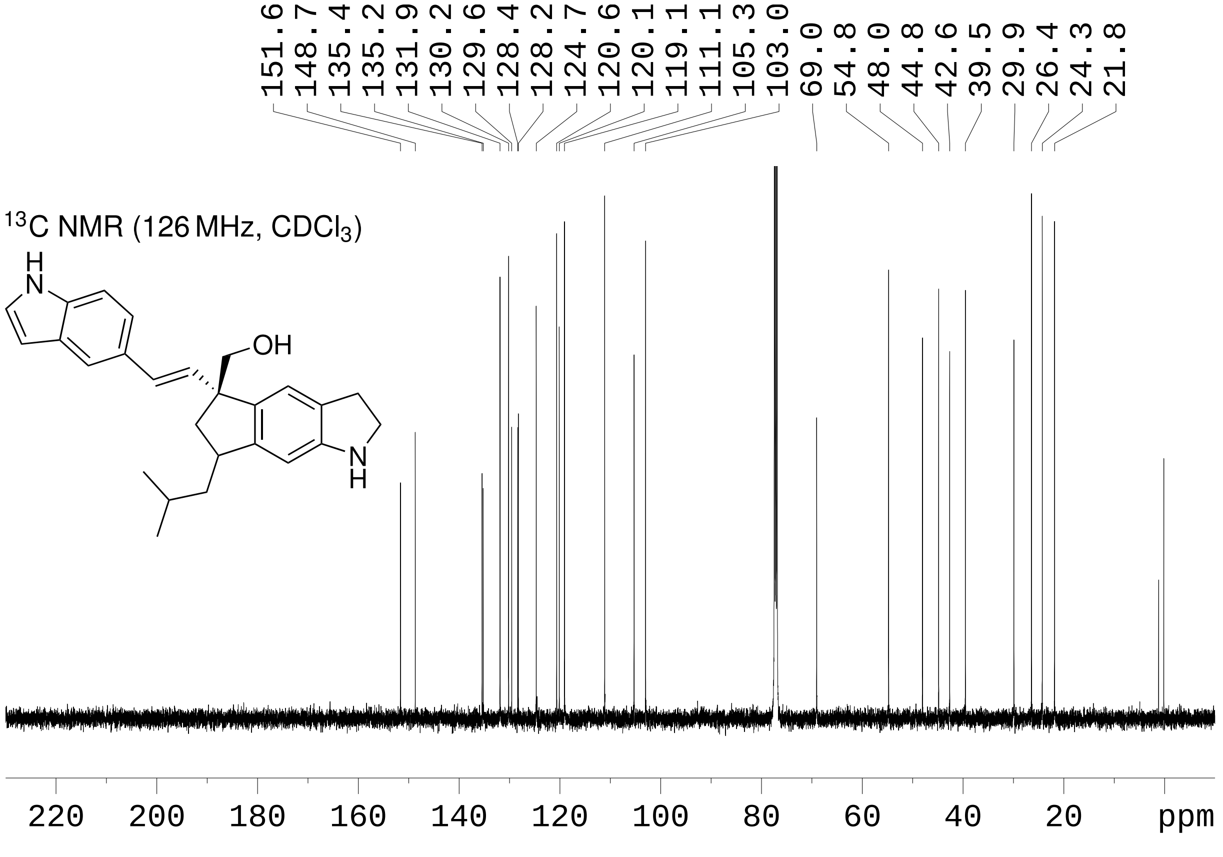

5.16 ((5*R*,7*R*)-5-((*E*)-2-(1*H*-indol-5-yl)vinyl)-7-isobutyl-1,5,6,7-tetrahydrocyclopenta[*f*]indol-5-yl)methanol (3)

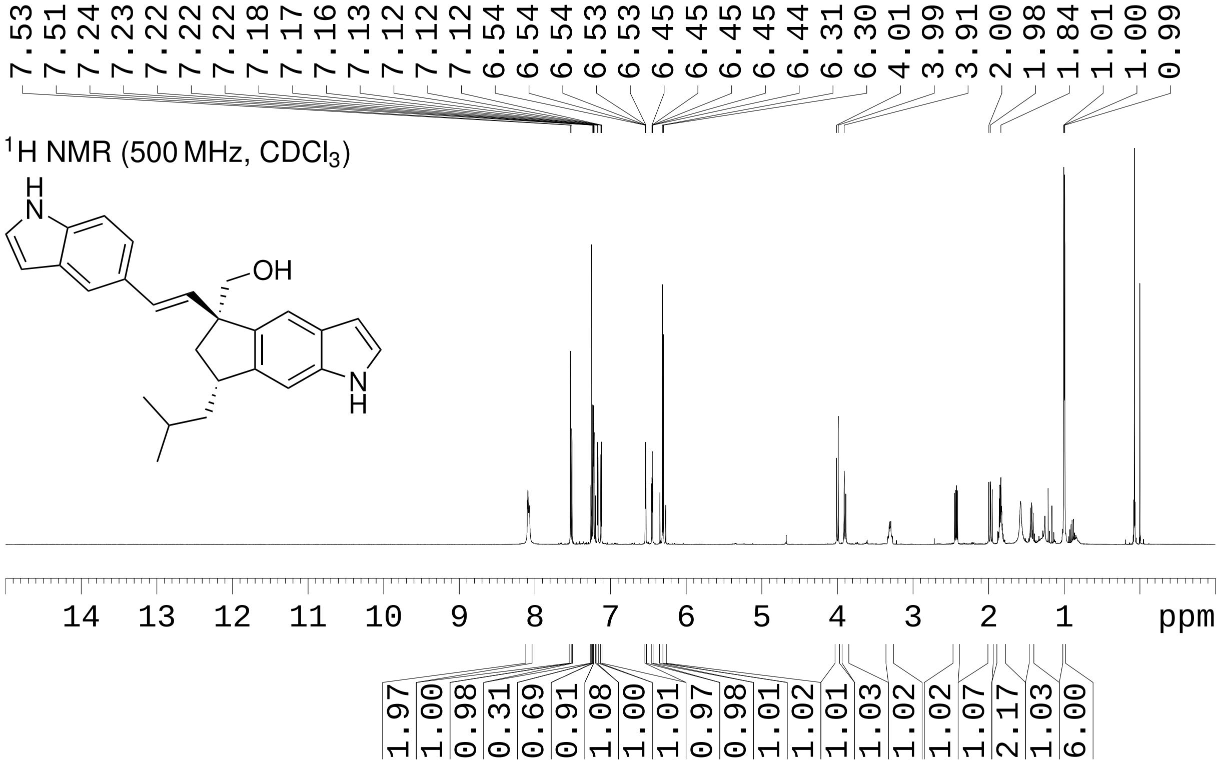

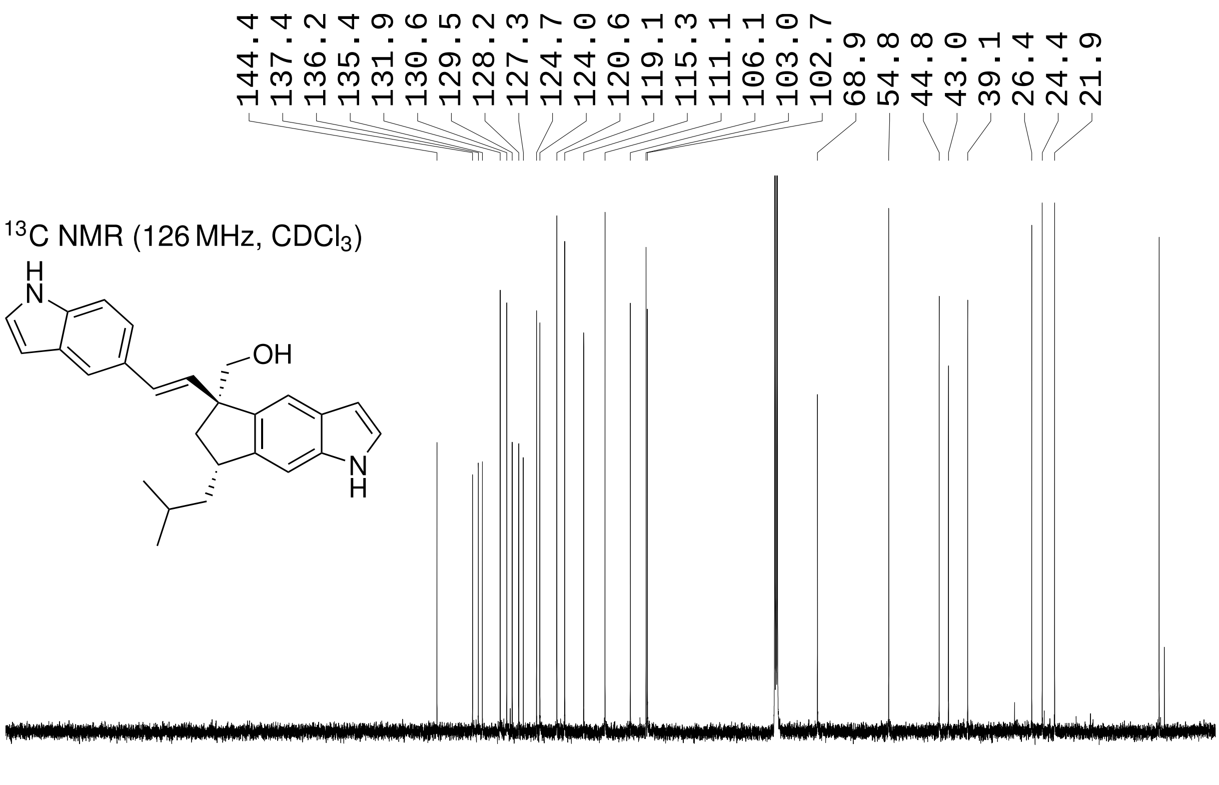

6.1 Dimer 20a: rel. energy 0.00 kcal mol^{-1}

1\1\GINC-HEISENBERG13\FOpt\RB3LYP\6-31G(d)\C48H54N2O8S2\MFRESIA\26-May
-2020\0\\# opt=tight int=(grid=ultrafine) b3lyp/6-31g(d) geom=connecti
vity\\Dimer_RR_opt_B3LYP_6-31G-d\\0,1\C,-7.7430402237,-0.2323645113,-2
.0240567641\C,-6.6335694203,0.5723284735,-1.6796544116\C,-5.5933424992
,0.7868128092,-2.5870081527\C,-5.6855804844,0.1375340162,-3.8149292154
\C,-6.7977778965,-0.6298658061,-4.1833823512\C,-7.848758627,-0.8171318
67,-3.2836680348\N,-4.7285613879,0.2060556562,-4.8903319088\C,-5.44979
5971,-0.2721997046,-6.1110855519\C,-6.6102203006,-1.1507744362,-5.5913
273776\C,-8.6797271291,-0.3169557903,-0.8388407511\C,-7.9672892746,0.5
500905423,0.1774032287\C,-6.8098114596,1.0693704418,-0.2969388298\C,-1
0.1009313425,0.2466639409,-1.1235293742\C,-8.8121096715,-1.7893588809,
-0.3679385487\O,-10.8935895176,-0.6136430382,-1.9335608369\O,-9.740830
1629,-1.8974695523,0.7011376446\C,-5.8103491412,2.0118742279,0.3592106
667\C,-5.0234679822,1.397021575,1.5979025317\C,-6.4650368504,3.4074901
384,0.701478075\C,-7.4177957751,3.2631367888,1.9071793331\C,-6.8437784
007,2.4525748204,3.0464122544\C,-5.787333618,1.6440290586,2.8842143107
\C,-7.2459745915,3.9419936578,-0.5167386977\C,-5.4039903558,4.47788120
28,1.0477736926\C,-7.5327213492,2.6005470464,4.379588856\C,-4.72570139
11,-0.1027305705,1.4516319507\C,-3.3733554518,-0.3627192936,1.19591691
7\C,-2.5265090299,0.893192629,1.2710619381\C,-3.5581396511,1.989054015
1,1.6781710735\C,-1.4591538752,0.7163784537,2.3683171416\C,-1.79880826
03,1.2320717261,-0.0527177069\O,-2.6768558453,1.4570791495,-1.14716503
35\O,-0.7528208283,1.9444048295,2.5247431971\C,-5.6358555195,-1.156005
1985,1.5943468645\C,-5.1632398951,-2.4537324507,1.4104358196\C,-3.8247
510679,-2.720068233,1.0930074346\C,-2.9181980706,-1.6716715478,0.99319
24855\N,-5.9203890189,-3.6714380379,1.4322918056\C,-5.0516325377,-4.76
60273027,0.9009395912\C,-3.6091634104,-4.2102725044,0.9500068593\H,-7.
6823069052,4.2656068678,2.2758668011\H,-8.3714293263,2.818294855,1.588
698542\H,-5.0412317821,2.2111642084,-0.3917021379\C,-2.0774652194,0.82
5164805,-5.2542103982\C,-1.6957684742,1.8806847654,-4.4217763988\C,-0.
8564543433,2.866787899,-4.9345796595\C,-0.3931192796,2.8197037006,-6.2
5804763\C,-0.7809976088,1.7393779999,-7.0610924322\C,-1.6182912086,0.7
393571783,-6.5687525442\C,0.4914805889,3.9151081927,-6.8038925126\S,-3
.1613957718,-0.4567609284,-4.6258048807\O,-2.9748222162,-1.645033876,-
5.4703211968\O,-2.9622273925,-0.5570653363,-3.1716373828\C,-6.12425131
85,-4.0907117886,4.2196551746\C,-5.7056665655,-5.3306710926,4.70863471
05\C,-4.9766777687,-5.3814498113,5.894962194\C,-4.6584983065,-4.213742
9731,6.6023959816\C,-5.0933714662,-2.9831893219,6.0885541411\C,-5.8257
223128,-2.9101103038,4.906241494\C,-3.8977875211,-4.2809178037,7.90492
08876\S,-7.0177088759,-4.0263953858,2.6670755729\O,-7.5207720209,-5.36
8632202,2.3754617453\O,-7.9251395658,-2.8689996825,2.7455171473\H,-4.7
440982167,1.4136514225,-2.3479356155\H,-8.7285368586,-1.3933037432,-3.
555448355\H,-4.7686716665,-0.8004356504,-6.7770504185\H,-5.8342034705,

0.6162372303,-6.6231944291\H,-7.503802314,-1.0434455813,-6.2152059004\
H,-6.3338677938,-2.2141488603,-5.5893637781\H,-8.3697650942,0.68625499
33,1.1741052869\H,-10.599636687,0.4382986978,-0.1654044073\H,-10.01637
57903,1.1935272734,-1.6650613355\H,-9.1717569701,-2.3886121153,-1.2151
350032\H,-7.826648656,-2.1768561658,-0.09110554\H,-11.1911058001,-1.33
35316765,-1.3539531592\H,-9.2732520333,-2.2721311659,1.4685396208\H,-5
.4117566779,1.0976272543,3.751304729\H,-7.6794178218,4.9227209682,-0.2
843498295\H,-6.5845821971,4.0685426542,-1.3826827704\H,-8.0604494433,3
.2750812591,-0.8099493494\H,-4.6528599774,4.5717578155,0.2548807306\H,
-5.8950394737,5.4536700764,1.1491279816\H,-4.8885286612,4.273828193,1.
9893764341\H,-8.5989716554,2.3446293885,4.3040453834\H,-7.085165382,1.
9576053163,5.1447104742\H,-7.487488868,3.6391033521,4.7362403103\H,-3.
4493463165,2.8592349367,1.0343719689\H,-3.3487780812,2.3291858819,2.69
75166547\H,-0.7687572608,-0.0977007274,2.0916185415\H,-1.9577361363,0.
4226098256,3.3052921687\H,-1.2282318244,2.1531927958,0.0930227079\H,-1
.0811867559,0.4326162114,-0.2927407104\H,-2.8686924895,0.6072261279,-1
.5837400002\H,-0.1094905788,1.8305458649,3.2399961931\H,-6.6722420107,
-0.9731332102,1.8363651508\H,-1.8717222002,-1.8723769798,0.7741486487\
H,-5.3667757467,-4.9631488916,-0.1281734555\H,-5.194055558,-5.68221462
71,1.4747286751\H,-3.04909079,-4.6127265183,1.805289036\H,-3.053750913
2,-4.4752163367,0.0447401636\H,-2.0236086574,1.9213257815,-3.386727942
2\H,-0.5494429999,3.6865654102,-4.2894930667\H,-0.4168412711,1.6731391
286,-8.0833842313\H,-1.8955801454,-0.1080701443,-7.1864141365\H,1.2015
477259,4.2698828148,-6.0491358596\H,1.0610627074,3.5752723826,-7.67461
38191\H,-0.1050664978,4.7813702631,-7.1192639206\H,-5.9702234823,-6.23
83223923,4.1768481983\H,-4.6564272934,-6.3458241394,6.2815842806\H,-4.
8613654097,-2.0672656953,6.6264958571\H,-6.1732189579,-1.9555712661,4.
5277486459\H,-3.2487584904,-5.1615079263,7.9457692701\H,-3.2768105448,
-3.3913689935,8.0531412111\H,-4.5862465648,-4.3428169524,8.7580613671\
\Version=ES64L-G09RevD.01\State=1-A\HF=-3369.1279014\RMSD=9.405e-09\RM
SF=3.120e-07\Dipole=3.0321013,0.1708941,1.5018811\Quadrupole=-22.45598
78,-6.9835061,29.4394939,-5.0499267,5.4211059,-18.0766661\PG=C01 [X(C4
8H54N2O8S2)]\\@

6.2 Dimer 20b: rel. energy 14.16 kcal mol^{-1}

1\1\GINC-HEISENBERG13\FOpt\RB3LYP\6-31G(d)\C48H54N2O8S2\MFRESIA\26-May
-2020\0\\# opt=tight int=(grid=ultrafine) b3lyp/6-31g(d) geom=connecti
vity\\Dimer_RS_opt_B3LYP_6-31G-d\\0,1\C,-4.6246014858,4.4918363101,0.9
282449079\C,-5.0730278296,3.1540650372,0.994121513\C,-5.4988772167,2.5
998275255,2.2030292246\C,-5.398773734,3.4031490639,3.3373253498\C,-4.9
604132678,4.7313688288,3.2841569678\C,-4.580238704,5.2957843143,2.0645
662963\N,-5.7103255124,3.0081999688,4.6810868907\C,-5.7265978745,4.263
6820287,5.4970716603\C,-4.9326455759,5.3110648207,4.6814043948\C,-4.19
20202939,4.7959578824,-0.4949366905\C,-4.4970202449,3.4845901697,-1.19
53508495\C,-4.9747455031,2.5447878894,-0.3486066486\C,-4.9692904981,5.
9577646593,-1.1379250559\C,-2.6735956618,5.0760126889,-0.5329763939\O,
-4.6744509759,7.1598075464,-0.4269541143\O,-2.275420685,5.1663047429,-
1.9016828656\C,-5.356425174,1.0995663454,-0.6370669563\C,-4.1849320588
,0.2542625808,-1.3393616091\C,-6.7432205597,1.068816393,-1.4021352371\
C,-6.5307737011,1.499692139,-2.8669006292\C,-5.3785561019,0.7890396032
,-3.5393064336\C,-4.377023921,0.2351610624,-2.841473209\C,-7.750891911
1,2.0244336823,-0.7258222795\C,-7.3964093933,-0.3304466334,-1.39184313
66\C,-5.4088500867,0.7481224355,-5.0465336785\C,-4.0356157801,-1.18352
42085,-0.7650962099\C,-2.9160181306,-1.2917112319,0.0772242722\C,-2.17
26295644,0.0253267962,0.1938519123\C,-2.7365125077,0.7934424038,-1.024
8482457\C,-2.5278884329,0.6495351709,1.5568259189\C,-0.6439484161,-0.1
087746739,0.1144841887\O,-0.280538668,-0.7941237826,-1.0814192737\O,-1
.9518432157,1.9412693383,1.6949510672\C,-4.8147286476,-2.3156733721,-1
.032143525\C,-4.4881934072,-3.5086022886,-0.3877540084\C,-3.3830435114
,-3.6119447121,0.4652250487\C,-2.5846156117,-2.4987921998,0.696117399\
N,-5.1114881633,-4.7918403014,-0.5212516431\C,-4.5879469363,-5.6695345
672,0.5739229431\C,-3.2162010017,-5.048296623,0.9051589584\H,-7.456245
5879,1.3119001367,-3.4315941276\H,-6.3734664827,2.587098749,-2.9224246
967\H,-5.5424167748,0.6201659767,0.3301005353\C,-5.7448332862,0.287113
6478,4.7583035125\C,-5.1222049129,-0.7183660578,4.0168606245\C,-5.8744
85747,-1.8073870187,3.5815050917\C,-7.2390344833,-1.9171099543,3.88763
53235\C,-7.8317955924,-0.9008441046,4.6527813866\C,-7.0981091507,0.199
3808038,5.0903232568\C,-8.0403150512,-3.1018150062,3.4047907817\S,-4.7
819495691,1.6945800113,5.3077408257\O,-4.8655238211,1.8071020744,6.768
4114611\O,-3.4622852017,1.6149975518,4.6540662721\C,-6.8544011547,-4.7
105598447,-2.6115953218\C,-5.9996299246,-5.4308260181,-3.4505078643\C,
-6.1320517796,-5.2955272845,-4.8284011897\C,-7.1119507518,-4.458689108
9,-5.3866800782\C,-7.9627089449,-3.7596890873,-4.5218446649\C,-7.84280
31998,-3.8790174915,-3.1370077364\C,-7.2256406863,-4.3038442484,-6.884
2235777\S,-6.7765696798,-4.9430284517,-0.8375005142\O,-7.5477844151,-3
.8559068424,-0.2136868036\O,-7.0896506411,-6.3380336041,-0.5111208169\
H,-5.8721974035,1.5844244386,2.2706018547\H,-4.2521469573,6.3283216874
,1.9995623823\H,-5.329886217,4.0704448409,6.4931742428\H,-6.7755300273

,4.5629609309,5.5950960224\H,-5.3966511013,6.3009177109,4.7488203907\H
,-3.900225712,5.4105731856,5.0447959663\H,-4.2481444968,3.3331834901,-
2.2386763117\H,-4.6647070251,6.0419827065,-2.1896237194\H,-6.045990824
5,5.7325078112,-1.1011025725\H,-2.4708141986,6.0166990031,-0.003840167
5\H,-2.1554600039,4.257405986,-0.0147721975\H,-5.0902846997,7.89397079
15,-0.9024024601\H,-1.3274785649,5.3643553079,-1.9181684216\H,-3.57924
82373,-0.268106698,-3.3888048111\H,-8.7069642565,1.9898977074,-1.26231
75292\H,-7.9452454332,1.7272934883,0.3118746935\H,-7.4080979075,3.0618
857482,-0.7211818369\H,-7.3182549065,-0.8270302146,-0.4195727545\H,-8.
4630026574,-0.2375239461,-1.6310682706\H,-6.9593841167,-0.9856044778,-
2.1464646227\H,-5.4580622989,1.7618458883,-5.469179708\H,-4.5234846384
,0.253583385,-5.4591254388\H,-6.299676705,0.2169147705,-5.4111011662\H
,-2.1009819596,0.5370552175,-1.8780217982\H,-2.6917429762,1.8707729268
,-0.8923940273\H,-2.1595388378,-0.0215465568,2.3510573509\H,-3.6179436
479,0.6966400582,1.6588488046\H,-0.2062127939,0.8979000377,0.149984569
9\H,-0.2795766041,-0.6547380612,1.0011150881\H,0.6757727776,-0.6935482
578,-1.1962242742\H,-2.2845162402,2.2949755554,2.5373456019\H,-5.64258
49992,-2.2744222211,-1.7225968405\H,-1.7007897945,-2.5764851922,1.3249
922451\H,-4.5389689169,-6.7019967514,0.2290051103\H,-5.2515587384,-5.6
280214061,1.4462282612\H,-2.9671920842,-5.163053999,1.9651348163\H,-2.
4195954773,-5.5366679851,0.3272789676\H,-4.070156724,-0.6341356705,3.7
717123924\H,-5.3949066154,-2.5748911385,2.9792145051\H,-8.8879740709,-
0.967943957,4.9009661194\H,-7.5724529775,0.9875830458,5.6654704847\H,-
7.8917760016,-3.2783210159,2.3333842019\H,-7.73809262,-4.0178102361,3.
929167128\H,-9.1107374449,-2.9594356796,3.5820029437\H,-5.2375109292,-
6.0765218948,-3.0275447144\H,-5.464652894,-5.849285638,-5.4842222268\H
,-8.7317690686,-3.1118657692,-4.9347962189\H,-8.5001088716,-3.33435356
57,-2.4681985425\H,-7.1064599842,-5.2652217804,-7.3957980141\H,-8.1940
401451,-3.8831265554,-7.1716937238\H,-6.4465961674,-3.6334387133,-7.27
01536745\\Version=ES64L-G09RevD.01\State=1-A\HF=-3369.1053364\RMSD=3.4
58e-09\RMSF=1.592e-07\Dipole=0.0873889,0.5194391,-2.1951388\Quadrupole
=1.1460158,4.414626,-5.5606418,-12.7558964,-2.8664832,4.1330015\PG=C01
[X(C48H54N2O8S2)]\\@

6.3 Triflate 13

6.3.1 Conformation 1 (internal code: M0022): rel. energy 1.52 kcal mol^{-1}

1\1\GINC-HEISENBERG03\SP\RwB97XD TD-FC\tApr-CC-pVDZ/Auto\C28H24F3N1O8S
2\MFRESIA\04-May-2020\0\\# td=(nstates=60) wB97XD/TApr-cc-pVDZ/auto sc
rf=(solvent=Acetonitrile) geom=connectivity\\Triflat_M0022_td_wB97XD_T
Apr-cc-pVDZ_Acetonitrile\\0,1\C,0,0.639099,-1.35467,-1.06984\C,0,-0.20
4241,-0.253727,-1.26799\C,0,-1.588468,-0.365302,-1.36113\C,0,-2.105558
,-1.6483,-1.201182\C,0,-1.278571,-2.768129,-1.027028\C,0,0.10497,-2.63
704,-0.967109\N,0,-3.468243,-2.057285,-1.219324\C,0,-3.531352,-3.45794
7,-0.722593\C,0,-2.127528,-4.011883,-0.99876\C,0,2.082661,-0.886582,-1
.000804\C,0,1.951975,0.610215,-1.191014\C,0,0.658606,0.923918,-1.33510
4\C,0,2.629459,-1.201417,0.401183\C,0,2.944332,-1.512742,-2.122926\O,0
,3.094699,-2.901727,-1.978626\O,0,3.906,-0.585158,0.627147\C,0,5.01179
7,-1.304956,0.421095\C,0,8.622292,0.782666,1.344495\C,0,7.395492,1.412
716,1.560185\C,0,6.208294,0.748029,1.265078\C,0,6.250878,-0.554177,0.7
52664\C,0,7.481517,-1.184415,0.537211\C,0,8.665451,-0.515603,0.832692\
O,0,5.007214,-2.451426,0.007219\O,0,0.151273,2.201095,-1.562818\S,0,-0
.242782,3.083586,-0.230651\O,0,-0.688279,2.197596,0.839725\O,0,0.76651
3,4.109742,-0.015723\C,0,-1.743268,3.890975,-1.009992\F,0,-2.195505,4.
797368,-0.161471\F,0,-1.400813,4.462075,-2.148304\F,0,-2.672828,2.9714
89,-1.223195\C,0,-4.287205,-0.699014,1.054576\C,0,-3.427759,0.349379,1
.385631\C,0,-3.080751,0.538814,2.718778\C,0,-3.588255,-0.292218,3.7260
87\C,0,-4.461335,-1.326988,3.364856\C,0,-4.817615,-1.53707,2.036503\C,
0,-3.232138,-0.05699,5.169248\S,0,-4.677479,-1.013929,-0.653208\O,0,-4
.541916,0.239831,-1.410827\O,0,-5.917588,-1.801165,-0.720039\H,0,-2.22
8899,0.494002,-1.544405\H,0,0.750446,-3.507464,-0.849157\H,0,-3.733201
,-3.467002,0.362463\H,0,-4.331336,-3.999824,-1.236571\H,0,-1.816798,-4
.738045,-0.237134\H,0,-2.089643,-4.514061,-1.978482\H,0,2.788192,1.306
411,-1.194583\H,0,1.965917,-0.773302,1.162064\H,0,2.709536,-2.285599,0
.553286\H,0,2.441499,-1.326751,-3.083674\H,0,3.922218,-0.99841,-2.1604
43\H,0,3.764739,-3.027208,-1.284689\H,0,9.551176,1.306767,1.576208\H,0
,7.363901,2.42661,1.961529\H,0,5.247012,1.233384,1.430377\H,0,7.495556
,-2.197915,0.135544\H,0,9.624984,-1.006431,0.664244\H,0,-3.03513,1.016
556,0.621001\H,0,-2.398533,1.351268,2.97551\H,0,-4.872271,-1.980819,4.
136624\H,0,-5.51028,-2.333357,1.76357\H,0,-3.239104,-0.995434,5.740609
\H,0,-2.241438,0.407821,5.266595\H,0,-3.964367,0.621355,5.635948\\Vers
ion=ES64L-G09RevD.01\State=1-A\HF=-2833.249171\RMSD=3.799e-09\PG=C01 [
X(C28H24F3N1O8S2)]\\@

6.3.2 Conformation 2 (internal code: M0129): rel. energy 0.34 kcal mol^{-1}

1\1\GINC-HEISENBERG12\SP\RwB97XD TD-FC\tApr-CC-pVDZ/Auto\C28H24F3N1O8S
2\MFRESIA\03-Jan-2020\0\\# td=(nstates=60) wB97XD/TApr-cc-pVDZ/auto sc
rf=(solvent=Acetonitrile) geom=connectivity\\Triflat_M0129_td_wB97XD_T
Apr-cc-pVDZ_Acetonitrile\\0,1\C,0,-0.287671,-0.827237,-0.935242\C,0,0.
352251,0.38775,-1.224314\C,0,1.675498,0.455779,-1.652446\C,0,2.337415,
-0.763946,-1.758442\C,0,1.714901,-1.988976,-1.486546\C,0,0.388235,-2.0
36045,-1.066805\N,0,3.710382,-0.95444,-2.120526\C,0,3.867043,-2.410999
,-2.388315\C,0,2.702589,-3.112324,-1.66751\C,0,-1.701251,-0.556032,-0.
455587\C,0,-1.80005,0.951388,-0.544956\C,0,-0.62595,1.443663,-0.961345
\C,0,-2.704123,-1.236879,-1.397234\C,0,-1.870197,-1.011242,1.017009\O,
0,-1.766933,-2.402363,1.171291\O,0,-4.054835,-0.855226,-1.094983\C,0,-
4.784889,-1.659017,-0.317536\C,0,-8.791956,-0.318591,0.306809\C,0,-7.9
37477,0.440272,-0.494967\C,0,-6.628082,0.019743,-0.713045\C,0,-6.17329
5,-1.166066,-0.123981\C,0,-7.030972,-1.924823,0.680364\C,0,-8.338841,-
1.501321,0.894126\O,0,-4.359796,-2.687007,0.181198\O,0,-0.274589,2.770
62,-1.1869\S,0,-0.38216,3.861257,0.03363\O,0,-0.055959,5.134656,-0.583
251\O,0,-1.575707,3.640015,0.836631\C,0,1.090298,3.319522,1.066304\F,0
,1.27894,4.211673,2.02025\F,0,0.833096,2.132197,1.599843\F,0,2.164796,
3.239754,0.299098\C,0,4.343918,-0.895696,0.580374\C,0,4.875051,-2.0989
88,1.041241\C,0,4.392141,-2.632793,2.232869\C,0,3.390235,-1.982684,2.9
65193\C,0,2.890352,-0.764345,2.484604\C,0,3.363711,-0.212542,1.300548\
C,0,2.877901,-2.559675,4.257142\S,0,4.826874,-0.274519,-1.017618\O,0,6
.142895,-0.823985,-1.381059\O,0,4.588206,1.176991,-1.047888\H,0,2.1763
77,1.39901,-1.861533\H,0,-0.095021,-2.981001,-0.819836\H,0,3.787233,-2
.542313,-3.4767\H,0,4.86321,-2.749429,-2.083051\H,0,2.300458,-3.941305
,-2.264202\H,0,3.002554,-3.519613,-0.688703\H,0,-2.687137,1.52125,-0.2
80486\H,0,-2.535448,-0.904569,-2.428339\H,0,-2.60831,-2.329102,-1.3452
72\H,0,-1.066546,-0.544897,1.60674\H,0,-2.829619,-0.625242,1.408617\H,
0,-2.614214,-2.770508,0.865768\H,0,-9.817687,0.014281,0.474866\H,0,-8.
294197,1.36394,-0.952779\H,0,-5.956339,0.607265,-1.338096\H,0,-6.65785
2,-2.844213,1.132039\H,0,-9.006936,-2.093827,1.520704\H,0,5.654657,-2.
611097,0.476233\H,0,4.802531,-3.57558,2.599803\H,0,2.115593,-0.237787,
3.045532\H,0,2.976417,0.734989,0.931345\H,0,1.796401,-2.395039,4.36340
3\H,0,3.077071,-3.637682,4.323797\H,0,3.370249,-2.073871,5.114709\\Ver
sion=ES64L-G09RevD.01\State=1-A\HF=-2833.2515626\RMSD=9.865e-09\PG=C01
[X(C28H24F3N1O8S2)]\\@

6.3.3 Conformation 3 (internal code: M0151): rel. energy 1.48 kcal mol^{-1}

1\1\GINC-HEISENBERG01\SP\RwB97XD TD-FC\tApr-CC-pVDZ/Auto\C28H24F3N1O8S
2\MFRESIA\04-May-2020\0\\# td=(nstates=60) wB97XD/TApr-cc-pVDZ/auto sc
rf=(solvent=Acetonitrile) geom=connectivity\\Triflat_M0151_td_wB97XD_T
Apr-cc-pVDZ_Acetonitrile\\0,1\C,0,-0.045855,-0.534181,-0.458524\C,0,0.
417033,0.6908,0.046905\C,0,1.689542,1.181866,-0.229463\C,0,2.487532,0.
370059,-1.029192\C,0,2.040885,-0.846796,-1.559196\C,0,0.761137,-1.3179
79,-1.27722\N,0,3.842044,0.6352,-1.413031\C,0,4.14563,-0.322809,-2.513
178\C,0,3.145046,-1.482769,-2.363948\C,0,-1.461666,-0.783311,0.028685\
C,0,-1.740865,0.439508,0.876154\C,0,-0.671575,1.245328,0.852617\C,0,-2
.400585,-0.823702,-1.187539\C,0,-1.544389,-2.070886,0.884071\O,0,-1.28
1224,-3.234221,0.142963\O,0,-3.780014,-0.850595,-0.791707\C,0,-4.40167
2,-2.030165,-0.718924\C,0,-8.532774,-1.750298,0.330423\C,0,-7.936534,-
2.995496,0.122971\C,0,-6.589696,-3.071286,-0.218434\C,0,-5.836186,-1.8
99703,-0.353192\C,0,-6.434255,-0.651296,-0.143718\C,0,-7.782308,-0.580
656,0.197681\O,0,-3.854305,-3.098692,-0.930277\O,0,-0.504572,2.454187,
1.518247\S,0,-1.437208,3.744163,1.10766\O,0,-2.764543,3.319382,0.68791
\O,0,-1.200242,4.720775,2.156496\C,0,-0.513897,4.296605,-0.428651\F,0,
-1.076715,5.403949,-0.877718\F,0,0.752411,4.523194,-0.12268\F,0,-0.594
166,3.343886,-1.34497\C,0,4.841908,-1.017767,0.571765\C,0,3.765912,-1.
33241,1.403664\C,0,3.608372,-2.647289,1.827317\C,0,4.513713,-3.64639,1
.444483\C,0,5.596204,-3.294171,0.627277\C,0,5.767472,-1.986084,0.18530
8\C,0,4.343535,-5.061055,1.928818\S,0,4.958057,0.620411,-0.117128\O,0,
6.285505,0.792977,-0.727573\O,0,4.453682,1.59305,0.864737\H,0,2.055433
,2.125321,0.169947\H,0,0.410468,-2.275853,-1.660853\H,0,3.981018,0.215
772,-3.457009\H,0,5.199294,-0.622581,-2.479785\H,0,2.804175,-1.85108,-
3.340321\H,0,3.576828,-2.335844,-1.815523\H,0,-2.677799,0.61987,1.3974
23\H,0,-2.288479,0.0976,-1.771544\H,0,-2.18023,-1.6911,-1.823298\H,0,-
0.786148,-1.999894,1.678312\H,0,-2.53314,-2.115748,1.376997\H,0,-2.090
22,-3.417592,-0.365169\H,0,-9.589581,-1.690243,0.596507\H,0,-8.523112,
-3.909191,0.228007\H,0,-6.10689,-4.034753,-0.383504\H,0,-5.843529,0.25
798,-0.249739\H,0,-8.250685,0.391123,0.359971\H,0,3.056917,-0.562616,1
.708226\H,0,2.762293,-2.903087,2.468278\H,0,6.316702,-4.057328,0.32684
6\H,0,6.60784,-1.715552,-0.454414\H,0,4.759877,-5.782526,1.212342\H,0,
4.868949,-5.202033,2.886912\H,0,3.284098,-5.300356,2.094273\\Version=E
S64L-G09RevD.01\State=1-A\HF=-2833.2493566\RMSD=9.561e-09\PG=C01 [X(C2
8H24F3N1O8S2)]\\@

6.3.4 Conformation 4 (internal code: M0153): rel. energy 1.48 kcal mol^{-1}

1\1\GINC-HEISENBERG02\SP\RwB97XD TD-FC\tApr-CC-pVDZ/Auto\C28H24F3N1O8S
2\MFRESIA\04-May-2020\0\\# td=(nstates=60) wB97XD/TApr-cc-pVDZ/auto sc
rf=(solvent=Acetonitrile) geom=connectivity\\Triflat_M0153_td_wB97XD_T
Apr-cc-pVDZ_Acetonitrile\\0,1\C,0,-0.045827,-0.534202,-0.458527\C,0,0.
417031,0.69079,0.046904\C,0,1.689533,1.181882,-0.229448\C,0,2.487557,0
.370087,-1.029159\C,0,2.040949,-0.846794,-1.559139\C,0,0.7612,-1.31799
4,-1.277194\N,0,3.842063,0.635252,-1.412986\C,0,4.145688,-0.322787,-2.
5131\C,0,3.145184,-1.482795,-2.363777\C,0,-1.46165,-0.783347,0.028651\
C,0,-1.74087,0.43946,0.876137\C,0,-0.671597,1.245302,0.852605\C,0,-2.4
00536,-0.823694,-1.187604\C,0,-1.544393,-2.070926,0.884018\O,0,-1.2811
98,-3.234258,0.142917\O,0,-3.779979,-0.850576,-0.791828\C,0,-4.401622,
-2.030152,-0.719031\C,0,-8.532669,-1.75039,0.330591\C,0,-7.782242,-0.5
80727,0.197801\C,0,-6.43421,-0.651332,-0.14369\C,0,-5.836122,-1.899722
,-0.353211\C,0,-6.589595,-3.071324,-0.218407\C,0,-7.936411,-2.995571,0
.123092\O,0,-3.854243,-3.098661,-0.930435\O,0,-0.504622,2.454165,1.518
236\S,0,-1.437309,3.744103,1.107656\O,0,-2.764657,3.319267,0.688005\O,
0,-1.200308,4.720771,2.156433\C,0,-0.514095,4.296512,-0.428722\F,0,-1.
076977,5.403821,-0.877802\F,0,0.752219,4.523159,-0.122806\F,0,-0.59436
7,3.343757,-1.344997\C,0,4.841965,-1.017654,0.571827\C,0,5.767582,-1.9
85932,0.185392\C,0,5.596318,-3.294043,0.627291\C,0,4.513785,-3.646326,
1.444414\C,0,3.608405,-2.647262,1.827247\C,0,3.765939,-1.332357,1.4036
64\C,0,4.343576,-5.061022,1.928652\S,0,4.958057,0.620528,-0.117062\O,0
,6.285505,0.79316,-0.727488\O,0,4.453611,1.593158,0.864773\H,0,2.05540
3,2.125348,0.169959\H,0,0.410557,-2.275878,-1.660826\H,0,3.981019,0.21
5743,-3.456951\H,0,5.199371,-0.622491,-2.479723\H,0,2.804413,-1.85132,
-3.340097\H,0,3.577022,-2.335705,-1.815131\H,0,-2.67781,0.619798,1.397
401\H,0,-2.288388,0.09762,-1.771581\H,0,-2.180181,-1.691077,-1.823384\
H,0,-0.786185,-1.999929,1.67829\H,0,-2.533163,-2.115804,1.376909\H,0,-
2.090151,-3.417594,-0.365296\H,0,-9.589459,-1.690363,0.596749\H,0,-8.2
50634,0.391038,0.360126\H,0,-5.843517,0.25796,-0.249744\H,0,-6.106776,
-4.034779,-0.383515\H,0,-8.522957,-3.909281,0.228166\H,0,6.607977,-1.7
15362,-0.454278\H,0,6.316852,-4.057168,0.326871\H,0,2.762298,-2.903104
,2.468153\H,0,3.056911,-0.562593,1.708222\H,0,3.284119,-5.300336,2.093
971\H,0,4.759996,-5.782455,1.212183\H,0,4.868868,-5.202048,2.886803\\V
ersion=ES64L-G09RevD.01\State=1-A\HF=-2833.2493565\RMSD=9.564e-09\PG=C
01 [X(C28H24F3N1O8S2)]\\@

6.3.5 Conformation 5 (internal code: M0171): rel. energy 0.05 kcal mol^{-1}

1\1\GINC-HEISENBERG12\SP\RwB97XD TD-FC\tApr-CC-pVDZ/Auto\C28H24F3N1O8S
2\MFRESIA\03-Jan-2020\0\\# td=(nstates=60) wB97XD/TApr-cc-pVDZ/auto sc
rf=(solvent=Acetonitrile) geom=connectivity\\Triflat_M0171_td_wB97XD_T
Apr-cc-pVDZ_Acetonitrile\\0,1\C,0,0.103366,-0.827182,-1.444776\C,0,-0.
52136,0.426417,-1.531521\C,0,-1.901692,0.57061,-1.641784\C,0,-2.63352,
-0.613317,-1.631135\C,0,-2.03092,-1.875105,-1.549485\C,0,-0.646839,-1.
998768,-1.460579\N,0,-4.059233,-0.728406,-1.693842\C,0,-4.348773,-2.16
4634,-1.960894\C,0,-3.09228,-2.944824,-1.537991\C,0,1.605396,-0.64109,
-1.333379\C,0,1.759471,0.863209,-1.401581\C,0,0.547052,1.425347,-1.488
654\C,0,2.051312,-1.151462,0.047883\C,0,2.360354,-1.335204,-2.491081\O
,0,2.252157,-2.734866,-2.446151\O,0,3.412431,-0.795962,0.330205\C,0,4.
373534,-1.687902,0.077466\C,0,8.290863,-0.347793,1.128389\C,0,7.196474
,0.482142,1.377905\C,0,5.911641,0.062081,1.044047\C,0,5.723144,-1.1959
,0.458717\C,0,6.82136,-2.026801,0.209839\C,0,8.103466,-1.602128,0.5446
61\O,0,4.167832,-2.784436,-0.413433\O,0,0.228226,2.778774,-1.546256\S,
0,0.650533,3.753284,-0.293771\O,0,1.990029,3.445539,0.185253\O,0,0.232
416,5.081406,-0.705255\C,0,-0.558062,3.13676,1.007147\F,0,-0.478149,3.
933752,2.056001\F,0,-1.785453,3.155315,0.51466\F,0,-0.235839,1.897517,
1.354947\C,0,-4.15965,-0.754823,1.079188\C,0,-3.002345,-0.186077,1.610
751\C,0,-2.372734,-0.814707,2.678969\C,0,-2.888226,-1.996211,3.228982\
C,0,-4.062033,-2.534171,2.683881\C,0,-4.704408,-1.922275,1.611782\C,0,
-2.213075,-2.654145,4.402017\S,0,-4.888603,-0.029933,-0.375235\O,0,-4.
555667,1.402851,-0.402894\O,0,-6.285708,-0.476369,-0.493871\H,0,-2.386
713,1.542318,-1.708627\H,0,-0.163769,-2.97436,-1.4098\H,0,-5.268232,-2
.469448,-1.4491\H,0,-4.521159,-2.258091,-3.042428\H,0,-3.191553,-3.374
211,-0.527874\H,0,-2.876058,-3.767821,-2.231397\H,0,2.712752,1.381643,
-1.33999\H,0,1.460244,-0.661433,0.830803\H,0,1.927603,-2.239649,0.1226
\H,0,1.919466,-0.992967,-3.439202\H,0,3.416173,-1.006947,-2.484636\H,0
,2.869707,-3.028811,-1.754138\H,0,9.296787,-0.014482,1.389796\H,0,7.34
5261,1.46149,1.834901\H,0,5.053506,0.705578,1.235317\H,0,6.656419,-3.0
02053,-0.248747\H,0,8.959638,-2.249226,0.349112\H,0,-2.602194,0.733925
,1.188856\H,0,-1.462788,-0.376198,3.093463\H,0,-4.482983,-3.448815,3.1
06039\H,0,-5.619198,-2.344499,1.194895\H,0,-2.398122,-3.736891,4.41546
8\H,0,-1.128047,-2.482212,4.38584\H,0,-2.600834,-2.238614,5.346016\\Ve
rsion=ES64L-G09RevD.01\State=1-A\HF=-2833.2519305\RMSD=9.949e-09\PG=C0
1 [X(C28H24F3N1O8S2)]\\@

6.3.6 Conformation 6 (internal code: M0194): rel. energy 0.04 kcal mol^{-1}

1\1\GINC-HEISENBERG11\SP\RwB97XD TD-FC\tApr-CC-pVDZ/Auto\C28H24F3N1O8S
2\MFRESIA\03-Jan-2020\0\\# td=(nstates=60) wB97XD/TApr-cc-pVDZ/auto sc
rf=(solvent=Acetonitrile) geom=connectivity\\Triflat_M0194_td_wB97XD_T
Apr-cc-pVDZ_Acetonitrile\\0,1\C,0,0.099741,-0.823674,-1.446171\C,0,-0.
524722,0.43023,-1.530359\C,0,-1.905135,0.574954,-1.638904\C,0,-2.63724
,-0.608791,-1.629439\C,0,-2.034903,-1.870904,-1.550801\C,0,-0.650761,-
1.995062,-1.463413\N,0,-4.063162,-0.723257,-1.689854\C,0,-4.353488,-2.
158862,-1.95942\C,0,-3.096579,-2.940317,-1.540004\C,0,1.601882,-0.6381
21,-1.335336\C,0,1.756325,0.866226,-1.401567\C,0,0.543985,1.42881,-1.4
86813\C,0,2.048299,-1.15027,0.045128\C,0,2.356233,-1.331062,-2.494128\
O,0,2.247859,-2.730764,-2.450631\O,0,3.409557,-0.79516,0.327297\C,0,4.
370544,-1.686838,0.073195\C,0,8.288251,-0.347841,1.124116\C,0,8.100659
,-1.601523,0.539049\C,0,6.818429,-2.025856,0.204259\C,0,5.720291,-1.19
5269,0.454517\C,0,5.908981,0.062052,1.041203\C,0,7.193936,0.481771,1.3
75028\O,0,4.164644,-2.782814,-0.418883\O,0,0.225413,2.782415,-1.542184
\S,0,0.64936,3.755124,-0.288905\O,0,1.98946,3.446641,0.187945\O,0,0.23
0786,5.083859,-0.697961\C,0,-0.557705,3.137143,1.012733\F,0,-0.476547,
3.933109,2.062294\F,0,-1.785673,3.156236,0.521692\F,0,-0.235147,1.8975
65,1.358922\C,0,-4.155304,-0.757963,1.082412\C,0,-4.7018,-1.92386,1.61
6639\C,0,-4.05869,-2.536816,2.687642\C,0,-2.880231,-2.003629,3.22756\C
,0,-2.366994,-0.81978,2.680595\C,0,-2.997457,-0.190027,1.613444\C,0,-2
.169837,-2.697787,4.358064\S,0,-4.889022,-0.028152,-0.367131\O,0,-4.55
5936,1.404638,-0.391875\O,0,-6.286527,-0.474035,-0.483118\H,0,-2.38996
7,1.546898,-1.703491\H,0,-0.167816,-2.970827,-1.414796\H,0,-5.272226,-
2.464463,-1.44679\H,0,-4.527529,-2.250037,-3.040881\H,0,-3.194583,-3.3
71757,-0.530644\H,0,-2.881629,-3.761927,-2.235444\H,0,2.709794,1.38429
1,-1.339871\H,0,1.457588,-0.661194,0.828912\H,0,1.924584,-2.238541,0.1
18521\H,0,1.91502,-0.987733,-3.441702\H,0,3.412106,-1.002988,-2.487766
\H,0,2.865865,-3.025557,-1.759385\H,0,9.294271,-0.014791,1.385487\H,0,
8.95678,-2.248361,0.342412\H,0,6.653331,-3.000603,-0.25534\H,0,5.05090
3,0.705308,1.233544\H,0,7.342875,1.460615,1.83305\H,0,-5.621366,-2.341
004,1.205159\H,0,-4.48486,-3.446272,3.115628\H,0,-1.460593,-0.377971,3
.099206\H,0,-2.600424,0.733089,1.19539\H,0,-1.628845,-1.981339,4.99128
6\H,0,-2.87164,-3.263336,4.986117\H,0,-1.43094,-3.412291,3.960682\\Ver
sion=ES64L-G09RevD.01\State=1-A\HF=-2833.2519335\RMSD=9.931e-09\PG=C01
[X(C28H24F3N1O8S2)]\\@

6.3.7 Conformation 7 (internal code: M0226): rel. energy 1.30 kcal mol^{-1}

1\1\GINC-HEISENBERG12\SP\RwB97XD TD-FC\tApr-CC-pVDZ/Auto\C28H24F3N1O8S
2\MFRESIA\03-Jan-2020\0\\# td=(nstates=60) wB97XD/TApr-cc-pVDZ/auto sc
rf=(solvent=Acetonitrile) geom=connectivity\\Triflat_M0226_td_wB97XD_T
Apr-cc-pVDZ_Acetonitrile\\0,1\C,0,1.084335,0.530884,-1.074857\C,0,-0.2
9626,0.710843,-1.223585\C,0,-1.171892,-0.324941,-1.538278\C,0,-0.59793
4,-1.588532,-1.651993\C,0,0.784612,-1.790869,-1.526768\C,0,1.643953,-0
.73389,-1.245371\N,0,-1.240266,-2.826266,-1.925891\C,0,-0.269085,-3.91
7238,-1.646242\C,0,1.099736,-3.239046,-1.795314\C,0,1.733592,1.857257,
-0.723195\C,0,0.551138,2.805729,-0.683494\C,0,-0.564481,2.123251,-0.96
5032\C,0,2.352115,1.826013,0.684493\C,0,2.752158,2.292289,-1.803026\O,
0,3.324124,3.544593,-1.5296\O,0,3.367836,0.818819,0.794528\C,0,4.64298
5,1.168597,0.60613\C,0,7.388393,-2.081165,0.990007\C,0,6.026973,-2.310
869,1.195542\C,0,5.11715,-1.263281,1.079341\C,0,5.574325,0.019915,0.75
4318\C,0,6.939445,0.248188,0.548939\C,0,7.844799,-0.801671,0.66743\O,0
,5.002689,2.301151,0.336037\O,0,-1.844564,2.655503,-1.067789\S,0,-2.88
7809,2.341246,0.169084\O,0,-4.083005,1.73341,-0.399977\O,0,-2.172484,1
.755702,1.296516\C,0,-3.265784,4.133504,0.567859\F,0,-4.129524,4.15664
1,1.567355\F,0,-2.143771,4.734575,0.922253\F,0,-3.782547,4.717965,-0.4
96008\C,0,-2.815999,-2.892981,0.350314\C,0,-3.056741,-1.636403,0.91073
8\C,0,-2.955516,-1.483817,2.287582\C,0,-2.629545,-2.567685,3.115932\C,
0,-2.419757,-3.820721,2.528372\C,0,-2.511587,-3.994322,1.149618\C,0,-2
.514667,-2.375306,4.604045\S,0,-2.835944,-3.088028,-1.418024\O,0,-3.64
5471,-2.00874,-2.00481\O,0,-3.109457,-4.495698,-1.742403\H,0,-2.237415
,-0.159508,-1.688079\H,0,2.716724,-0.903576,-1.145973\H,0,-0.397687,-4
.285125,-0.61389\H,0,-0.430243,-4.746725,-2.341477\H,0,1.841973,-3.659
525,-1.104828\H,0,1.48736,-3.356995,-2.81962\H,0,0.626784,3.867102,-0.
459151\H,0,2.772843,2.805245,0.946854\H,0,1.588995,1.544624,1.419681\H
,0,3.52079,1.505321,-1.917198\H,0,2.220737,2.365198,-2.763814\H,0,3.99
7363,3.387685,-0.845868\H,0,8.099166,-2.904344,1.081485\H,0,5.67251,-3
.31134,1.447509\H,0,4.053282,-1.435084,1.240851\H,0,7.276764,1.253569,
0.295805\H,0,8.908935,-0.62389,0.506602\H,0,-3.337219,-0.792065,0.2828
95\H,0,-3.13126,-0.499112,2.724672\H,0,-2.183036,-4.680152,3.158534\H,
0,-2.365496,-4.976973,0.700478\H,0,-2.320525,-3.324759,5.119919\H,0,-1
.694751,-1.680136,4.841296\H,0,-3.438221,-1.940011,5.014162\\Version=E
S64L-G09RevD.01\State=1-A\HF=-2833.249975\RMSD=3.856e-09\PG=C01 [X(C28
H24F3N1O8S2)]\\@

6.3.8 Conformation 8 (internal code: M0231): rel. energy 0.00 kcal mol^{-1}

1\1\GINC-HEISENBERG13\SP\RwB97XD TD-FC\tApr-CC-pVDZ/Auto\C28H24F3N1O8S
2\MFRESIA\03-Jan-2020\0\\# td=(nstates=60) wB97XD/TApr-cc-pVDZ/auto sc
rf=(solvent=Acetonitrile) geom=connectivity\\Triflat_M0231_td_wB97XD_T
Apr-cc-pVDZ_Acetonitrile\\0,1\C,0,-0.731104,0.650091,-0.728256\C,0,0.5
75402,0.861895,-1.194809\C,0,1.378342,-0.169709,-1.675014\C,0,0.811342
,-1.440438,-1.642475\C,0,-0.49179,-1.675151,-1.18612\C,0,-1.282513,-0.
627878,-0.722348\N,0,1.444864,-2.662877,-2.034785\C,0,0.364786,-3.6858
94,-2.106642\C,0,-0.798652,-3.148587,-1.255242\C,0,-1.318086,1.962189,
-0.246596\C,0,-0.205885,2.950095,-0.529769\C,0,0.842264,2.292994,-1.04
286\C,0,-2.563255,2.379946,-1.041529\C,0,-1.586894,1.89749,1.279406\O,
0,-2.072831,3.108717,1.793283\O,0,-3.601022,1.392689,-0.96214\C,0,-4.5
54361,1.528019,-0.036463\C,0,-7.462774,-1.600003,-0.124986\C,0,-6.4397
03,-1.592005,-1.074441\C,0,-5.485338,-0.578467,-1.062767\C,0,-5.556094
,0.431406,-0.095143\C,0,-6.582919,0.422151,0.855482\C,0,-7.53447,-0.59
3091,0.839568\O,0,-4.594409,2.44181,0.76847\O,0,2.079313,2.802059,-1.4
26966\S,0,3.012794,3.582516,-0.325336\O,0,4.165077,4.04068,-1.081028\O
,0,2.211359,4.451883,0.523543\C,0,3.54914,2.120202,0.726663\F,0,4.4675
16,2.5379,1.577252\F,0,2.501618,1.646413,1.388429\F,0,4.047979,1.17448
3,-0.053052\C,0,2.263123,-3.007252,0.592599\C,0,2.313443,-1.786487,1.2
65964\C,0,1.775222,-1.699556,2.543837\C,0,1.192427,-2.815516,3.16095\C
,0,1.181323,-4.034097,2.470386\C,0,1.71235,-4.140443,1.187548\C,0,0.58
0122,-2.693087,4.530137\S,0,2.812777,-3.0818,-1.099908\O,0,3.13171,-4.
475122,-1.449883\O,0,3.795879,-2.010195,-1.322415\H,0,2.394419,-0.0058
18,-2.028554\H,0,-2.294038,-0.811213,-0.358956\H,0,0.073475,-3.764913,
-3.163511\H,0,0.746584,-4.663255,-1.791778\H,0,-1.770025,-3.358411,-1.
721605\H,0,-0.811235,-3.585977,-0.244083\H,0,-0.262946,4.009134,-0.293
548\H,0,-2.943719,3.346367,-0.685299\H,0,-2.321469,2.452451,-2.108317\
H,0,-2.271809,1.055902,1.492116\H,0,-0.632392,1.674166,1.779617\H,0,-3
.015708,3.139321,1.558182\H,0,-8.209652,-2.395601,-0.136799\H,0,-6.386
29,-2.378367,-1.828521\H,0,-4.686487,-0.565534,-1.803444\H,0,-6.621785
,1.215232,1.602538\H,0,-8.333836,-0.601106,1.581666\H,0,2.770506,-0.92
0185,0.792036\H,0,1.81128,-0.745411,3.0732\H,0,0.751245,-4.918611,2.94
4024\H,0,1.704318,-5.09352,0.657761\H,0,0.442066,-3.676103,4.999823\H,
0,1.204282,-2.072869,5.189124\H,0,-0.407266,-2.209246,4.46268\\Version
=ES64L-G09RevD.01\State=1-A\HF=-2833.2519924\RMSD=4.390e-09\PG=C01 [X(
C28H24F3N1O8S2)]\\@

6.3.9 Conformation 9 (internal code: M0257): rel. energy 0.50 kcal mol^{-1}

1\1\GINC-HEISENBERG12\SP\RwB97XD TD-FC\tApr-CC-pVDZ/Auto\C28H24F3N1O8S
2\MFRESIA\03-Jan-2020\0\\# td=(nstates=60) wB97XD/TApr-cc-pVDZ/auto sc
rf=(solvent=Acetonitrile) geom=connectivity\\Triflat_M0257_td_wB97XD_T
Apr-cc-pVDZ_Acetonitrile\\0,1\C,0,0.476631,-1.514278,-0.875865\C,0,-0.
276523,-0.430963,-1.347899\C,0,-1.665136,-0.46644,-1.461786\C,0,-2.278
566,-1.652357,-1.058756\C,0,-1.537866,-2.757867,-0.613298\C,0,-0.15183
3,-2.702708,-0.515541\N,0,-3.662168,-1.98792,-1.077702\C,0,-3.843521,-
3.253298,-0.318137\C,0,-2.464035,-3.920981,-0.375452\C,0,1.948772,-1.1
46698,-0.857537\C,0,1.94433,0.259272,-1.41718\C,0,0.681459,0.629556,-1
.66623\C,0,2.424761,-1.13767,0.604734\C,0,2.794044,-2.088316,-1.746837
\O,0,2.834254,-3.40526,-1.259874\O,0,3.740973,-0.578405,0.733148\C,0,4
.787173,-1.406799,0.773942\C,0,8.538067,0.56118,1.324199\C,0,7.367419,
1.321187,1.293788\C,0,6.135096,0.698186,1.115746\C,0,6.076038,-0.69294
1,0.96829\C,0,7.250552,-1.453174,0.998662\C,0,8.479679,-0.825933,1.176
456\O,0,4.697293,-2.617666,0.664776\O,0,0.234647,1.831175,-2.206379\S,
0,0.543532,3.247679,-1.435156\O,0,1.803652,3.193746,-0.709489\O,0,0.21
6102,4.27802,-2.404259\C,0,-0.833931,3.181142,-0.165494\F,0,-0.784028,
4.268903,0.57916\F,0,-1.999321,3.113568,-0.792635\F,0,-0.672955,2.1059
06,0.594094\C,0,-4.634454,-0.227534,0.823912\C,0,-3.737139,0.813155,1.
065861\C,0,-3.519188,1.225056,2.37433\C,0,-4.192057,0.619495,3.445498\
C,0,-5.098128,-0.41191,3.170825\C,0,-5.328245,-0.840923,1.865902\C,0,-
3.959233,1.095112,4.854087\S,0,-4.855409,-0.812586,-0.841707\O,0,-4.55
9139,0.285721,-1.774745\O,0,-6.128569,-1.543291,-0.919649\H,0,-2.23518
8,0.374613,-1.850788\H,0,0.426119,-3.565176,-0.183579\H,0,-4.111854,-3
.031487,0.729386\H,0,-4.643164,-3.847546,-0.770996\H,0,-2.240895,-4.47
6888,0.544056\H,0,-2.40371,-4.62732,-1.218513\H,0,2.83822,0.86058,-1.5
61973\H,0,1.777245,-0.482844,1.200248\H,0,2.410077,-2.150589,1.027737\
H,0,2.336525,-2.114115,-2.747\H,0,3.808801,-1.664154,-1.861627\H,0,3.4
63994,-3.397887,-0.518471\H,0,9.502233,1.053219,1.463913\H,0,7.416006,
2.404742,1.410493\H,0,5.217327,1.285053,1.091063\H,0,7.184372,-2.53493
2,0.880111\H,0,9.395598,-1.418166,1.199445\H,0,-3.230447,1.300797,0.23
6411\H,0,-2.8141,2.035826,2.567946\H,0,-5.637676,-0.888848,3.991331\H,
0,-6.04742,-1.63301,1.656541\H,0,-4.343087,0.377158,5.590974\H,0,-2.88
8105,1.25418,5.044233\H,0,-4.467635,2.057731,5.02169\\Version=ES64L-G0
9RevD.01\State=1-A\HF=-2833.2507317\RMSD=3.944e-09\PG=C01 [X(C28H24F3N
1O8S2)]\\@

6.3.10 Conformation 10 (internal code: M0264): rel. energy 1.30 kcal mol^{-1}

1\1\GINC-HEISENBERG12\SP\RwB97XD TD-FC\tApr-CC-pVDZ/Auto\C28H24F3N1O8S
2\MFRESIA\03-Jan-2020\0\\# td=(nstates=60) wB97XD/TApr-cc-pVDZ/auto sc
rf=(solvent=Acetonitrile) geom=connectivity\\Triflat_M0264_td_wB97XD_T
Apr-cc-pVDZ_Acetonitrile\\0,1\C,0,1.084124,0.530837,-1.074978\C,0,-0.2
96463,0.710744,-1.223791\C,0,-1.172033,-0.325072,-1.538554\C,0,-0.5980
11,-1.588641,-1.652208\C,0,0.784543,-1.790902,-1.526975\C,0,1.643818,-
0.73389,-1.245525\N,0,-1.240298,-2.826416,-1.92612\C,0,-0.269018,-3.91
7333,-1.646539\C,0,1.099753,-3.239046,-1.795592\C,0,1.733288,1.857209,
-0.72315\C,0,0.550798,2.805627,-0.683456\C,0,-0.564751,2.123129,-0.965
206\C,0,2.351689,1.825848,0.684595\C,0,2.751927,2.292383,-1.802851\O,0
,3.323743,3.544728,-1.529329\O,0,3.367524,0.818762,0.794565\C,0,4.6426
35,1.168703,0.606244\C,0,7.388281,-2.080898,0.989604\C,0,6.02689,-2.31
0711,1.195218\C,0,5.116993,-1.263173,1.079185\C,0,5.574063,0.020085,0.
754268\C,0,6.939152,0.248475,0.548818\C,0,7.84458,-0.801339,0.667132\O
,0,5.002237,2.301338,0.336354\O,0,-1.84489,2.655237,-1.06805\S,0,-2.88
7963,2.341454,0.169057\O,0,-4.083452,1.734016,-0.399788\O,0,-2.172585,
1.755666,1.296321\C,0,-3.265397,4.133813,0.567822\F,0,-4.128768,4.1572
07,1.567639\F,0,-2.143177,4.734763,0.921772\F,0,-3.78244,4.718286,-0.4
95909\C,0,-2.815547,-2.893267,0.350528\C,0,-2.511154,-3.994623,1.14981
4\C,0,-2.419248,-3.821026,2.52857\C,0,-2.628894,-2.567979,3.116135\C,0
,-2.954794,-1.484077,2.2878\C,0,-3.056169,-1.636679,0.910974\C,0,-2.51
4027,-2.375572,4.604237\S,0,-2.835852,-3.08829,-1.417814\O,0,-3.645579
,-2.00903,-2.004358\O,0,-3.109361,-4.49597,-1.742152\H,0,-2.237557,-0.
159675,-1.68838\H,0,2.716593,-0.903538,-1.146075\H,0,-0.397584,-4.2853
16,-0.614219\H,0,-0.430119,-4.746779,-2.341838\H,0,1.842026,-3.65953,-
1.105149\H,0,1.487357,-3.35691,-2.819917\H,0,0.6264,3.866984,-0.45901\
H,0,2.772261,2.805101,0.947137\H,0,1.588548,1.544232,1.419675\H,0,3.52
0625,1.505475,-1.916995\H,0,2.220591,2.365294,-2.763686\H,0,3.996906,3
.387868,-0.845506\H,0,8.099113,-2.904044,1.080939\H,0,5.672509,-3.3112
32,1.44711\H,0,4.053142,-1.435062,1.240744\H,0,7.276384,1.253908,0.295
768\H,0,8.908695,-0.623474,0.506246\H,0,-2.365171,-4.977292,0.700673\H
,0,-2.182557,-4.680475,3.158726\H,0,-3.130451,-0.499353,2.724886\H,0,-
3.336666,-0.792339,0.283148\H,0,-3.437785,-1.94069,5.014345\H,0,-2.319
464,-3.324921,5.120141\H,0,-1.694468,-1.679974,4.841448\\Version=ES64L
-G09RevD.01\State=1-A\HF=-2833.249975\RMSD=3.854e-09\PG=C01 [X(C28H24F
3N1O8S2)]\\@

6.3.11 Conformation 11 (internal code: M0271): rel. energy 1.34 kcal mol^{-1}

1\1\GINC-HEISENBERG13\SP\RwB97XD TD-FC\tApr-CC-pVDZ/Auto\C28H24F3N1O8S
2\MFRESIA\03-Jan-2020\0\\# td=(nstates=60) wB97XD/TApr-cc-pVDZ/auto sc
rf=(solvent=Acetonitrile) geom=connectivity\\Triflat_M0271_td_wB97XD_T
Apr-cc-pVDZ_Acetonitrile\\0,1\C,0,1.080453,0.531407,-1.078472\C,0,-0.3
00553,0.709001,-1.226313\C,0,-1.174549,-0.328316,-1.540465\C,0,-0.5984
74,-1.590925,-1.654509\C,0,0.784571,-1.790704,-1.530872\C,0,1.642237,-
0.732261,-1.249881\N,0,-1.239194,-2.829824,-1.927114\C,0,-0.265265,-3.
919078,-1.650003\C,0,1.102102,-3.23818,-1.80026\C,0,1.72754,1.858643,-
0.726076\C,0,0.543525,2.805136,-0.685784\C,0,-0.571024,2.120863,-0.967
158\C,0,2.345633,1.827411,0.681844\C,0,2.745684,2.2961,-1.805258\O,0,3
.315498,3.549152,-1.53077\O,0,3.363149,0.821994,0.791598\C,0,4.637711,
1.174374,0.60418\C,0,7.38971,-2.069627,0.989241\C,0,6.028672,-2.302223
,1.194088\C,0,5.116684,-1.256573,1.077438\C,0,5.571317,0.027602,0.7527
2\C,0,6.936053,0.258783,0.548048\C,0,7.843567,-0.789152,0.666952\O,0,4
.995315,2.307747,0.334718\O,0,-1.852185,2.650766,-1.068909\S,0,-2.8928
62,2.337255,0.170249\O,0,-4.088682,1.72808,-0.395993\O,0,-2.174976,1.7
53332,1.296898\C,0,-3.271306,4.129598,0.568116\F,0,-4.133464,4.153073,
1.568966\F,0,-2.149111,4.731696,0.920234\F,0,-3.790093,4.712895,-0.495
41\C,0,-2.807247,-2.896553,0.35452\C,0,-3.048117,-1.639649,0.913328\C,
0,-2.937585,-1.483105,2.289318\C,0,-2.603825,-2.563752,3.118148\C,0,-2
.392572,-3.817661,2.532011\C,0,-2.493356,-3.995022,1.154697\C,0,-2.501
722,-2.374101,4.60752\S,0,-2.832787,-3.094306,-1.413415\O,0,-3.646493,
-2.017483,-1.99893\O,0,-3.104469,-4.502988,-1.73493\H,0,-2.240499,-0.1
64774,-1.689299\H,0,2.715381,-0.900103,-1.151336\H,0,-0.391613,-4.2884
26,-0.617936\H,0,-0.425742,-4.748158,-2.345895\H,0,1.845867,-3.657535,
-1.110739\H,0,1.488867,-3.354997,-2.825017\H,0,0.617458,3.866525,-0.46
0927\H,0,2.764472,2.80721,0.94513\H,0,1.582722,1.544058,1.416498\H,0,3
.515605,1.510433,-1.919745\H,0,2.214452,2.368826,-2.766163\H,0,3.98865
4,3.392885,-0.846792\H,0,8.102173,-2.891308,1.08106\H,0,5.676229,-3.30
3465,1.445844\H,0,4.053089,-1.430611,1.238402\H,0,7.27131,1.264925,0.2
95178\H,0,8.907414,-0.60915,0.506659\H,0,-3.333129,-0.797101,0.285157\
H,0,-3.10971,-0.496878,2.724297\H,0,-2.145337,-4.674102,3.162331\H,0,-
2.344381,-4.977775,0.706701\H,0,-2.023422,-3.235503,5.092185\H,0,-1.92
4292,-1.47011,4.849944\H,0,-3.50325,-2.249272,5.04865\\Version=ES64L-G
09RevD.01\State=1-A\HF=-2833.2499683\RMSD=3.883e-09\PG=C01 [X(C28H24F3
N1O8S2)]\\@

6.3.12 Conformation 12 (internal code: M0311): rel. energy 0.42 kcal mol^{-1}

1\1\GINC-HEISENBERG11\SP\RwB97XD TD-FC\tApr-CC-pVDZ/Auto\C28H24F3N1O8S
2\MFRESIA\03-Jan-2020\0\\# td=(nstates=60) wB97XD/TApr-cc-pVDZ/auto sc
rf=(solvent=Acetonitrile) geom=connectivity\\Triflat_M0311_td_wB97XD_T
Apr-cc-pVDZ_Acetonitrile\\0,1\C,0,0.464668,-1.508334,-0.895886\C,0,-0.
283203,-0.41808,-1.360342\C,0,-1.671925,-0.445507,-1.473683\C,0,-2.291
325,-1.63116,-1.078996\C,0,-1.556004,-2.744047,-0.643308\C,0,-0.169632
,-2.696458,-0.544924\N,0,-3.677047,-1.958115,-1.099001\C,0,-3.864459,-
3.231311,-0.353627\C,0,-2.487832,-3.904679,-0.41569\C,0,1.93829,-1.146
97,-0.872882\C,0,1.940835,0.262176,-1.424624\C,0,0.679793,0.640109,-1.
671242\C,0,2.410331,-1.145939,0.590873\C,0,2.782679,-2.087708,-1.76366
3\O,0,2.817911,-3.406489,-1.281093\O,0,3.727051,-0.58941,0.725245\C,0,
4.772189,-1.419194,0.763294\C,0,8.52446,0.543532,1.323503\C,0,7.354753
,1.304934,1.292031\C,0,6.122011,0.683572,1.111161\C,0,6.061595,-0.7072
94,0.961706\C,0,7.235168,-1.468917,0.993114\C,0,8.464709,-0.843339,1.1
73973\O,0,4.681099,-2.629431,0.648573\O,0,0.237963,1.847809,-2.201921\
S,0,0.552787,3.256361,-1.418725\O,0,1.817414,3.194284,-0.701526\O,0,0.
220467,4.296212,-2.375851\C,0,-0.816718,3.178209,-0.140923\F,0,-0.7622
53,4.259195,0.613187\F,0,-1.985806,3.116203,-0.761677\F,0,-0.651113,2.
096138,0.607917\C,0,-4.62498,-0.221485,0.834447\C,0,-5.324995,-0.84033
8,1.869268\C,0,-5.085036,-0.434489,3.179461\C,0,-4.160386,0.577138,3.4
6732\C,0,-3.48646,1.1931,2.403147\C,0,-3.714347,0.8042,1.088968\C,0,-3
.879623,0.988351,4.887206\S,0,-4.86019,-0.776884,-0.839306\O,0,-4.5595
63,0.333552,-1.756425\O,0,-6.139637,-1.495953,-0.92276\H,0,-2.237832,0
.401669,-1.855365\H,0,0.404095,-3.564256,-0.219531\H,0,-4.133664,-3.02
0178,0.695809\H,0,-4.666,-3.817062,-0.814181\H,0,-2.267697,-4.469244,0
.49929\H,0,-2.430068,-4.604322,-1.264477\H,0,2.837677,0.860165,-1.5647
35\H,0,1.762426,-0.492688,1.187707\H,0,2.392688,-2.160847,1.008978\H,0
,2.327458,-2.108636,-2.764987\H,0,3.798949,-1.666246,-1.874688\H,0,3.4
46891,-3.403605,-0.53904\H,0,9.48896,1.034317,1.465336\H,0,7.404413,2.
388304,1.410012\H,0,5.204999,1.271584,1.085572\H,0,7.167906,-2.550432,
0.872901\H,0,9.379901,-1.436658,1.197858\H,0,-6.058482,-1.616321,1.649
672\H,0,-5.633206,-0.911729,3.994058\H,0,-2.774983,1.995926,2.605985\H
,0,-3.207437,1.300863,0.265052\H,0,-3.741422,2.075865,4.966651\H,0,-4.
691831,0.687074,5.562096\H,0,-2.952388,0.512774,5.244681\\Version=ES64
L-G09RevD.01\State=1-A\HF=-2833.2507286\RMSD=3.963e-09\PG=C01 [X(C28H2
4F3N1O8S2)]\\@

6.4 Dihydroraputindole D (3)

6.4.1 Conformation 1 (internal code: M0005): rel. energy 2.00 kcal mol^{-1}

1\1\GINC-HEISENBERG03\SP\RwB97XD TD-FC\tApr-CC-pVDZ/Auto\C26H28N2O1\MF
RESIA\27-Mar-2020\0\\# td=(nstates=60) wB97XD/TApr-cc-pVDZ/auto scrf=(
solvent=methanol) geom=connectivity\\DihydroraputindolD_M0005_td_wB97X
D_TApr-cc-pVDZ_methanol\\0,1\C,0,1.408631,0.261281,0.735999\C,0,2.0619
33,0.052187,-0.502754\C,0,2.61075,1.101658,-1.216726\C,0,2.508713,2.37
8592,-0.642721\C,0,1.876279,2.607775,0.607384\C,0,1.310622,1.520467,1.
302751\N,0,2.964125,3.591824,-1.100933\C,0,2.644328,4.573082,-0.191075
\C,0,1.978805,4.018889,0.871141\C,0,0.852682,-1.05442,1.258756\C,0,1.7
05469,-2.085653,0.478372\C,0,2.018188,-1.419007,-0.874223\C,0,3.214308
,-1.991125,-1.649186\C,0,4.51666,-2.26838,-0.881038\C,0,5.094201,-1.04
0376,-0.174398\C,0,5.551452,-2.870097,-1.835237\C,0,1.022054,-1.249215
,2.775207\C,0,-0.617488,-1.249141,0.927405\O,0,0.229106,-0.369033,3.54
6547\C,0,-1.458457,-0.315245,0.462257\C,0,-2.892736,-0.473877,0.153943
\C,0,-3.580799,-1.704555,0.334286\C,0,-4.925676,-1.842102,0.04354\C,0,
-5.608881,-0.717502,-0.442843\C,0,-4.957186,0.526033,-0.635891\C,0,-3.
589579,0.631723,-0.33093\N,0,-6.921389,-0.557502,-0.809699\C,0,-7.1214
02,0.738136,-1.225912\C,0,-5.947657,1.44057,-1.13749\H,0,3.119883,0.95
0845,-2.170729\H,0,0.814761,1.65775,2.264424\H,0,3.45839,3.742989,-1.9
68327\H,0,2.922564,5.606395,-0.380568\H,0,1.603084,4.552881,1.739891\H
,0,2.640574,-2.254644,1.036835\H,0,1.202993,-3.058416,0.372093\H,0,1.1
3355,-1.552637,-1.524665\H,0,2.892446,-2.941216,-2.108774\H,0,3.438132
,-1.317531,-2.496509\H,0,4.300805,-3.030662,-0.111054\H,0,4.407745,-0.
636754,0.584214\H,0,5.296944,-0.231897,-0.896618\H,0,6.042667,-1.29476
,0.325277\H,0,6.475494,-3.140348,-1.300783\H,0,5.819573,-2.148009,-2.6
25307\H,0,5.166253,-3.777194,-2.32712\H,0,0.796603,-2.305897,3.023832\
H,0,2.069511,-1.057036,3.053962\H,0,-0.983971,-2.263577,1.128279\H,0,-
0.655883,-0.377529,3.154645\H,0,-1.056836,0.68623,0.27443\H,0,-3.03985
5,-2.573074,0.71291\H,0,-5.436852,-2.795179,0.188536\H,0,-3.069369,1.5
82419,-0.473161\H,0,-7.632194,-1.274544,-0.780683\H,0,-8.104903,1.0596
82,-1.557857\H,0,-5.805401,2.485544,-1.398827\\Version=ES64L-G09RevD.0
1\State=1-A\HF=-1192.0881667\RMSD=3.094e-09\PG=C01 [X(C26H28N2O1)]\\@

6.4.2 Conformation 2 (internal code: M0008): rel. energy 1.52 kcal mol^{-1}

1\1\GINC-HEISENBERG01\SP\RwB97XD TD-FC\tApr-CC-pVDZ/Auto\C26H28N2O1\MF
RESIA\26-Mar-2020\0\\# td=(nstates=60) wB97XD/TApr-cc-pVDZ/auto scrf=(
solvent=methanol) geom=connectivity\\DihydroraputindolD_M0008_td_wB97X
D_TApr-cc-pVDZ_methanol\\0,1\C,0,1.368831,0.351717,0.6597\C,0,1.997358
,0.004582,-0.55806\C,0,2.717493,0.933563,-1.28732\C,0,2.814772,2.22523
4,-0.747591\C,0,2.206047,2.591414,0.482197\C,0,1.463184,1.627155,1.191
374\N,0,3.466388,3.335087,-1.228313\C,0,3.292231,4.382148,-0.353294\C,
0,2.528378,3.974954,0.709344\C,0,0.610478,-0.837402,1.228426\C,0,1.234
475,-2.023193,0.444663\C,0,1.711639,-1.446045,-0.901601\C,0,2.831719,-
2.227161,-1.605836\C,0,4.026995,-2.702647,-0.76339\C,0,4.731953,-1.583
571,0.0063\C,0,5.0209,-3.440564,-1.663669\C,0,0.839887,-0.989231,2.743
85\C,0,-0.880498,-0.772132,0.970999\O,0,2.20685,-1.063194,3.093799\C,0
,-1.530528,0.181608,0.292618\C,0,-2.979433,0.232687,0.008587\C,0,-3.52
3911,1.460946,-0.450566\C,0,-4.871643,1.61684,-0.729865\C,0,-5.701906,
0.503874,-0.549324\C,0,-5.192714,-0.743749,-0.103787\C,0,-3.821827,-0.
867579,0.169788\N,0,-7.052358,0.352515,-0.745689\C,0,-7.412892,-0.9397
74,-0.441964\C,0,-6.309922,-1.649703,-0.045706\H,0,3.205728,0.679452,-
2.23021\H,0,0.969155,1.889213,2.130551\H,0,3.990715,3.379042,-2.090205
\H,0,3.735903,5.351344,-0.564822\H,0,2.228542,4.588456,1.554624\H,0,2.
095384,-2.387501,1.023483\H,0,0.530541,-2.85976,0.323661\H,0,0.851014,
-1.439313,-1.595866\H,0,2.380421,-3.118811,-2.073606\H,0,3.210057,-1.6
16426,-2.445867\H,0,3.651014,-3.43241,-0.024238\H,0,4.068731,-1.110401
,0.744862\H,0,5.083726,-0.794317,-0.678728\H,0,5.607374,-1.981394,0.54
4342\H,0,5.853144,-3.860432,-1.077478\H,0,5.450813,-2.755048,-2.413842
\H,0,4.536053,-4.268106,-2.205315\H,0,0.334481,-0.159181,3.273903\H,0,
0.379727,-1.927341,3.091787\H,0,-1.441248,-1.619448,1.385564\H,0,2.623
998,-0.256576,2.761478\H,0,-0.951814,1.029175,-0.089867\H,0,-2.852791,
2.312969,-0.582586\H,0,-5.269268,2.570833,-1.079768\H,0,-3.423074,-1.8
30819,0.494758\H,0,-7.684446,1.072959,-1.06391\H,0,-8.449079,-1.253809
,-0.534843\H,0,-6.294214,-2.693825,0.254524\\Version=ES64L-G09RevD.01\
State=1-A\HF=-1192.0883207\RMSD=2.856e-09\PG=C01 [X(C26H28N2O1)]\\@

6.4.3 Conformation 3 (internal code: M0012): rel. energy 1.16 kcal mol^{-1}

1\1\GINC-HEISENBERG03\SP\RwB97XD TD-FC\tApr-CC-pVDZ/Auto\C26H28N2O1\MF
RESIA\26-Mar-2020\0\\# td=(nstates=60) wB97XD/TApr-cc-pVDZ/auto scrf=(
solvent=methanol) geom=connectivity\\DihydroraputindolD_M0012_td_wB97X
D_TApr-cc-pVDZ_methanol\\0,1\C,0,1.328934,0.261331,0.710128\C,0,1.9479
09,0.120477,-0.553025\C,0,2.478207,1.209484,-1.221109\C,0,2.395047,2.4
51833,-0.573682\C,0,1.79211,2.613705,0.702041\C,0,1.244179,1.487944,1.
347675\N,0,2.840906,3.687877,-0.975168\C,0,2.543596,4.61804,-0.006364\
C,0,1.901778,4.00785,1.039573\C,0,0.796252,-1.077442,1.196882\C,0,1.56
5052,-2.078268,0.293249\C,0,1.880098,-1.324453,-1.012727\C,0,3.0751,-1
.853813,-1.820405\C,0,4.369422,-2.194981,-1.063661\C,0,4.924448,-1.040
464,-0.226661\C,0,5.423305,-2.689975,-2.057239\C,0,1.116508,-1.312664,
2.685154\C,0,-0.696853,-1.234733,0.995743\O,0,2.492864,-1.194937,2.982
363\C,0,-1.521371,-0.340615,0.435952\C,0,-2.972886,-0.482373,0.208204\
C,0,-3.682562,-1.673212,0.523108\C,0,-5.043766,-1.794703,0.310779\C,0,
-5.722484,-0.694732,-0.234299\C,0,-5.049615,0.507804,-0.564343\C,0,-3.
665692,0.597826,-0.336253\N,0,-7.047906,-0.526989,-0.549372\C,0,-7.235
148,0.734225,-1.064912\C,0,-6.040515,1.406004,-1.094079\H,0,2.956662,1
.113723,-2.197814\H,0,0.758625,1.590086,2.321636\H,0,3.316586,3.886085
,-1.843576\H,0,2.819139,5.659964,-0.145643\H,0,1.546824,4.493895,1.944
307\H,0,2.499637,-2.341498,0.809233\H,0,0.999647,-3.006412,0.123376\H,
0,0.997145,-1.401091,-1.673963\H,0,2.749472,-2.766231,-2.348562\H,0,3.
311017,-1.12391,-2.616275\H,0,4.149308,-3.031564,-0.376671\H,0,4.22876
1,-0.736994,0.568989\H,0,5.117315,-0.154936,-0.854863\H,0,5.874556,-1.
333546,0.24822\H,0,6.338811,-3.013674,-1.537993\H,0,5.702511,-1.887764
,-2.76154\H,0,5.050427,-3.540626,-2.649409\H,0,0.512187,-0.620038,3.30
1949\H,0,0.825723,-2.337443,2.964702\H,0,-1.090255,-2.198391,1.341891\
H,0,2.762289,-0.307456,2.708858\H,0,-1.097218,0.612773,0.10343\H,0,-3.
144567,-2.524762,0.942395\H,0,-5.570397,-2.717868,0.55813\H,0,-3.12953
4,1.517516,-0.584271\H,0,-7.774227,-1.218018,-0.426578\H,0,-8.225597,1
.056236,-1.375115\H,0,-5.885061,2.420645,-1.450334\\Version=ES64L-G09R
evD.01\State=1-A\HF=-1192.088963\RMSD=3.206e-09\PG=C01 [X(C26H28N2O1)]
\\@

6.4.4 Conformation 4 (internal code: M0069): rel. energy 0.33 kcal mol^{-1}

1\1\GINC-HEISENBERG01\SP\RwB97XD TD-FC\tApr-CC-pVDZ/Auto\C26H28N2O1\MF
RESIA\26-Mar-2020\0\\# td=(nstates=60) wB97XD/TApr-cc-pVDZ/auto scrf=(
solvent=methanol) geom=connectivity\\DihydroraputindolD_M0069_td_wB97X
D_TApr-cc-pVDZ_methanol\\0,1\C,0,-1.66827,-0.697415,0.752556\C,0,-2.32
4432,-0.006744,-0.295757\C,0,-3.116954,-0.670025,-1.215434\C,0,-3.2364
16,-2.060598,-1.058764\C,0,-2.590519,-2.771815,-0.013768\C,0,-1.788612
,-2.067417,0.906977\N,0,-3.947994,-2.974222,-1.79879\C,0,-3.775392,-4.
229516,-1.263301\C,0,-2.953156,-4.155813,-0.169094\C,0,-0.86213,0.2820
67,1.59134\C,0,-1.546068,1.626796,1.238983\C,0,-2.021936,1.476423,-0.2
17078\C,0,-3.18376,2.389858,-0.604218\C,0,-2.93038,3.890824,-0.406781\
C,0,-4.183031,4.687515,-0.775395\C,0,-1.720448,4.386165,-1.201485\C,0,
-0.929576,0.01649,3.104517\C,0,0.602956,0.338664,1.193684\O,0,-0.27727
9,-1.175826,3.493992\C,0,1.239029,-0.511566,0.376649\C,0,2.663944,-0.4
74479,-0.009646\C,0,3.19932,-1.607456,-0.676702\C,0,4.526035,-1.678908
,-1.067881\C,0,5.342367,-0.576607,-0.786727\C,0,4.840184,0.578775,-0.1
32312\C,0,3.491188,0.618236,0.25108\N,0,6.671042,-0.356373,-1.051214\C
,0,7.025029,0.890729,-0.590517\C,0,5.938943,1.502249,-0.021543\H,0,-3.
634222,-0.145457,-2.021354\H,0,-1.28422,-2.57825,1.728091\H,0,-4.50893
6,-2.761261,-2.610926\H,0,-4.261866,-5.089168,-1.716436\H,0,-2.641082,
-4.989643,0.454077\H,0,-2.427761,1.762374,1.890865\H,0,-0.881466,2.489
413,1.39374\H,0,-1.167144,1.680624,-0.887354\H,0,-3.444777,2.216051,-1
.664103\H,0,-4.070472,2.096138,-0.014173\H,0,-2.727068,4.063451,0.6657
12\H,0,-5.05546,4.354958,-0.191189\H,0,-4.425971,4.559277,-1.844019\H,
0,-4.040826,5.763645,-0.590601\H,0,-1.592948,5.473513,-1.083148\H,0,-1
.848511,4.177773,-2.277689\H,0,-0.785296,3.906164,-0.874978\H,0,-0.505
802,0.891771,3.636774\H,0,-1.98142,-0.084314,3.412561\H,0,1.159794,1.1
58159,1.665902\H,0,0.57976,-1.183134,3.044312\H,0,0.660057,-1.333035,-
0.058824\H,0,2.538727,-2.452725,-0.883519\H,0,4.917845,-2.560353,-1.57
7903\H,0,3.097245,1.511146,0.740779\H,0,7.293797,-1.004126,-1.512207\H
,0,8.044876,1.247021,-0.708537\H,0,5.922554,2.492164,0.425942\\Version
=ES64L-G09RevD.01\State=1-A\HF=-1192.0898821\RMSD=2.504e-09\PG=C01 [X(
C26H28N2O1)]\\@

6.4.5 Conformation 5 (internal code: M0078): rel. energy 0.00 kcal mol^{-1}

1\1\GINC-HEISENBERG03\SP\RwB97XD TD-FC\tApr-CC-pVDZ/Auto\C26H28N2O1\MF
RESIA\26-Mar-2020\0\\# td=(nstates=60) wB97XD/TApr-cc-pVDZ/auto scrf=(
solvent=methanol) geom=connectivity\\DihydroraputindolD_M0078_td_wB97X
D_TApr-cc-pVDZ_methanol\\0,1\C,0,1.559778,0.747835,0.805923\C,0,2.2633
84,0.270285,-0.327098\C,0,2.832614,1.135651,-1.244024\C,0,2.676282,2.5
09265,-0.996814\C,0,1.979165,3.00926,0.134152\C,0,1.409271,2.101722,1.
04997\N,0,3.127378,3.591929,-1.713175\C,0,2.741861,4.750939,-1.080376\
C,0,2.037978,4.444975,0.055305\C,0,1.033479,-0.425059,1.618532\C,0,1.9
50528,-1.574503,1.131527\C,0,2.276777,-1.245359,-0.336193\C,0,3.568508
,-1.873141,-0.857156\C,0,3.64092,-3.402768,-0.751955\C,0,4.998974,-3.8
99881,-1.250167\C,0,2.50198,-4.091884,-1.506088\C,0,1.15438,-0.239412,
3.14054\C,0,-0.414731,-0.762031,1.305103\O,0,0.302759,0.766382,3.65207
\C,0,-1.272404,-0.006012,0.606199\C,0,-2.686703,-0.299309,0.304657\C,0
,-3.335293,-1.479905,0.758712\C,0,-4.661605,-1.744559,0.470533\C,0,-5.
365891,-0.802916,-0.294394\C,0,-4.753706,0.385273,-0.765088\C,0,-3.404
115,0.623553,-0.454806\N,0,-6.667933,-0.796521,-0.727012\C,0,-6.899545
,0.349347,-1.451706\C,0,-5.756967,1.104978,-1.503094\H,0,3.384321,0.77
7027,-2.115352\H,0,0.873547,2.448568,1.934451\H,0,3.65536,3.547066,-2.
572726\H,0,3.005612,5.717668,-1.50098\H,0,1.609627,5.158413,0.754233\H
,0,2.886861,-1.559345,1.717796\H,0,1.490239,-2.564555,1.264439\H,0,1.4
34387,-1.584835,-0.966104\H,0,3.709487,-1.588579,-1.915871\H,0,4.418,-
1.435649,-0.302528\H,0,3.555718,-3.674707,0.315847\H,0,5.82585,-3.4277
82,-0.696629\H,0,5.132398,-3.662869,-2.319486\H,0,5.092047,-4.990927,-
1.134373\H,0,2.604748,-5.187058,-1.455079\H,0,2.508162,-3.802667,-2.57
1157\H,0,1.515212,-3.832419,-1.093116\H,0,0.957329,-1.213166,3.632522\
H,0,2.183162,0.059845,3.392665\H,0,-0.749164,-1.71497,1.733729\H,0,-0.
567309,0.62219,3.25323\H,0,-0.901906,0.938682,0.194107\H,0,-2.778119,-
2.205254,1.353477\H,0,-5.143421,-2.65633,0.827189\H,0,-2.913971,1.5341
35,-0.808685\H,0,-7.351963,-1.516384,-0.542754\H,0,-7.880417,0.535976,
-1.880422\H,0,-5.643496,2.060942,-2.006825\\Version=ES64L-G09RevD.01\S
tate=1-A\HF=-1192.0904659\RMSD=2.961e-09\PG=C01 [X(C26H28N2O1)]\\@

6.4.6 Conformation 6 (internal code: M0154): rel. energy 1.97 kcal mol^{-1}

1\1\GINC-HEISENBERG02\SP\RwB97XD TD-FC\tApr-CC-pVDZ/Auto\C26H28N2O1\MF
RESIA\26-Mar-2020\0\\# td=(nstates=60) wB97XD/TApr-cc-pVDZ/auto scrf=(
solvent=methanol) geom=connectivity\\DihydroraputindolD_M0154_td_wB97X
D_TApr-cc-pVDZ_methanol\\0,1\C,0,1.50103,0.736792,0.81144\C,0,2.216861
,0.333116,-0.342148\C,0,2.758816,1.260074,-1.215254\C,0,2.564953,2.614
779,-0.900762\C,0,1.856255,3.039622,0.253296\C,0,1.312717,2.072216,1.1
22096\N,0,2.985094,3.743252,-1.563319\C,0,2.56911,4.858755,-0.874307\C
,0,1.875526,4.478433,0.24516\C,0,1.003616,-0.48549,1.564374\C,0,1.9460
66,-1.588678,1.022584\C,0,2.272227,-1.182773,-0.427409\C,0,3.572922,-1
.729629,-1.019373\C,0,3.640505,-3.251409,-1.237983\C,0,3.973115,-4.030
365,0.038066\C,0,4.667402,-3.580656,-2.3252\C,0,1.124094,-0.37371,3.09
3896\C,0,-0.437714,-0.838871,1.237313\O,0,0.249581,0.583563,3.657113\C
,0,-1.314824,-0.068614,0.579266\C,0,-2.723718,-0.37796,0.268488\C,0,-3
.352319,-1.581153,0.69013\C,0,-4.673346,-1.861208,0.392385\C,0,-5.3927
28,-0.912247,-0.349092\C,0,-4.80099,0.298923,-0.786183\C,0,-3.456405,0
.552643,-0.466566\N,0,-6.694003,-0.917177,-0.784021\C,0,-6.944762,0.24
358,-1.477986\C,0,-5.81573,1.020497,-1.506502\H,0,3.31867,0.964353,-2.
104668\H,0,0.767325,2.358842,2.022059\H,0,3.51391,3.755447,-2.42341\H,
0,2.805714,5.851746,-1.247165\H,0,1.428753,5.144748,0.978218\H,0,2.878
039,-1.586407,1.614862\H,0,1.497703,-2.588843,1.104643\H,0,1.439876,-1
.516654,-1.076209\H,0,3.716516,-1.244754,-1.999598\H,0,4.425324,-1.407
611,-0.39237\H,0,2.648988,-3.583789,-1.59749\H,0,3.228959,-3.881693,0.
831562\H,0,4.954015,-3.717216,0.434655\H,0,4.026465,-5.110946,-0.16872
9\H,0,4.733017,-4.666396,-2.497409\H,0,5.670434,-3.227306,-2.030449\H,
0,4.409783,-3.099559,-3.281714\H,0,0.952634,-1.375653,3.536408\H,0,2.1
45971,-0.062207,3.358958\H,0,-0.749463,-1.819219,1.618727\H,0,-0.61820
9,0.437551,3.253969\H,0,-0.966974,0.902494,0.211279\H,0,-2.783966,-2.3
11189,1.268355\H,0,-5.139994,-2.789858,0.724874\H,0,-2.981977,1.481091
,-0.794505\H,0,-7.365327,-1.653942,-0.62115\H,0,-7.928064,0.424318,-1.
903647\H,0,-5.718465,1.991619,-1.983964\\Version=ES64L-G09RevD.01\Stat
e=1-A\HF=-1192.0874314\RMSD=3.084e-09\PG=C01 [X(C26H28N2O1)]\\@

6.4.7 Conformation 7 (internal code: M0200): rel. energy 2.01 kcal mol^{-1}

```
1\1\GINC-HEISENBERG01\SP\RwB97XD TD-FC\tApr-CC-pVDZ/Auto\C26H28N2O1\MF
RESIA\26-Mar-2020\0\\# td=(nstates=60) wB97XD/TApr-cc-pVDZ/auto scrf=(
solvent=methanol) geom=connectivity\\DihydroraputindolD_M0200_td_wB97X
D_TApr-cc-pVDZ_methanol\\0,1\C,0,1.653573,0.653171,0.7569\C,0,2.298121
,0.239774,-0.434773\C,0,2.854661,1.156947,-1.308881\C,0,2.749941,2.512
091,-0.955543\C,0,2.113427,2.946271,0.236379\C,0,1.552246,1.988712,1.1
04972\N,0,3.206031,3.631876,-1.60896\C,0,2.88175,4.751098,-0.877906\C,
0,2.21325,4.381675,0.260302\C,0,1.120531,-0.558569,1.502666\C,0,1.9777
45,-1.697733,0.897514\C,0,2.265609,-1.274119,-0.555152\C,0,3.517124,-1
.870839,-1.204005\C,0,3.560694,-3.398396,-1.406364\C,0,2.297904,-3.934
345,-2.084318\C,0,3.875734,-4.186117,-0.130839\C,0,1.306481,-0.490714,
3.027706\C,0,-0.349011,-0.826904,1.224488\O,0,0.508861,0.498947,3.6467
6\C,0,-1.20223,-0.004688,0.598791\C,0,-2.634282,-0.236224,0.329037\C,0
,-3.315261,-1.402087,0.773661\C,0,-4.656941,-1.611517,0.512693\C,0,-5.
343564,-0.628146,-0.214824\C,0,-4.699649,0.547903,-0.673404\C,0,-3.335
353,0.730224,-0.39039\N,0,-6.654263,-0.565038,-0.615578\C,0,-6.860777,
0.604799,-1.308899\C,0,-5.692974,1.319977,-1.370784\H,0,3.359677,0.853
128,-2.227887\H,0,1.060301,2.282203,2.033124\H,0,3.700774,3.636031,-2.
489155\H,0,3.158929,5.738533,-1.237361\H,0,1.834503,5.053043,1.02634\H
,0,2.931514,-1.763976,1.449862\H,0,1.478316,-2.673896,0.974371\H,0,1.3
88758,-1.539299,-1.173737\H,0,3.626775,-1.406507,-2.19911\H,0,4.402283
,-1.558184,-0.621594\H,0,4.403297,-3.575651,-2.098284\H,0,2.063335,-3.
371695,-3.002187\H,0,1.423894,-3.867025,-1.415536\H,0,2.421185,-4.9935
81,-2.358832\H,0,4.069056,-5.243752,-0.371054\H,0,3.04285,-4.161591,0.
587642\H,0,4.769323,-3.787227,0.374766\H,0,1.096834,-1.492213,3.454534
\H,0,2.35355,-0.242032,3.258614\H,0,-0.702145,-1.792337,1.60797\H,0,-0
.380401,0.411368,3.274811\H,0,-0.812291,0.948486,0.225949\H,0,-2.77176
9,-2.159274,1.340773\H,0,-5.164133,-2.512023,0.862769\H,0,-2.820619,1.
630598,-0.735385\H,0,-7.359142,-1.26475,-0.432277\H,0,-7.843889,0.8360
59,-1.709895\H,0,-5.556515,2.282647,-1.855715\\Version=ES64L-G09RevD.0
1\State=1-A\HF=-1192.0875861\RMSD=3.048e-09\PG=C01 [X(C26H28N2O1)]\\@
```

6.5 Raputindole D (2)

6.5.1 Conformation 1 (internal code: M0001): rel. energy 0.33 kcal mol^{-1}


```
1\1\GINC-HEISENBERG03\SP\RwB97XD TD-FC\tApr-CC-pVDZ/Auto\C26H26N2O1\MF
RESIA\17-May-2020\0\\# td=(nstates=60) wB97XD/TApr-cc-pVDZ/auto scrf=(
solvent=methanol) geom=connectivity\\RaputindolD_M0001_td_wB97XD_TApr-
cc-pVDZ_methanol\\0,1\C,0,-1.60669,-0.704905,0.85911\C,0,-2.317724,0.1
49741,-0.016518\C,0,-3.172431,-0.341169,-0.985708\C,0,-3.297301,-1.737
75,-1.062352\C,0,-2.594675,-2.616158,-0.195212\C,0,-1.73131,-2.081244,
0.782559\N,0,-4.061812,-2.513044,-1.900849\C,0,-3.868297,-3.840936,-1.
598149\C,0,-2.979766,-3.953606,-0.560636\C,0,-0.745484,0.119982,1.8030
11\C,0,-1.430553,1.508187,1.71605\C,0,-1.991121,1.603194,0.281462\C,0,
-3.160948,2.533144,0.144774\C,0,-3.318507,3.548919,-0.717067\C,0,-4.58
2064,4.370758,-0.716951\C,0,-2.303721,3.973869,-1.745373\C,0,-0.741629
,-0.392554,3.253164\C,0,0.698772,0.234115,1.347216\O,0,-0.090136,-1.63
7767,3.405616\C,0,1.277176,-0.449515,0.350491\C,0,2.68347,-0.361131,-0
.092151\C,0,3.132128,-1.294467,-1.063376\C,0,4.434186,-1.305451,-1.535
731\C,0,5.316096,-0.347539,-1.021037\C,0,4.902378,0.607005,-0.055038\C
,0,3.576402,0.590442,0.401959\N,0,6.641087,-0.113483,-1.291672\C,0,7.0
77967,0.949142,-0.534767\C,0,6.050799,1.424654,0.237009\H,0,-3.730575,
0.325218,-1.646907\H,0,-1.18391,-2.726538,1.47055\H,0,-4.671322,-2.165
363,-2.626928\H,0,-4.390009,-4.611634,-2.159329\H,0,-2.637295,-4.88073
,-0.108909\H,0,-2.270312,1.537247,2.432258\H,0,-0.750075,2.335273,1.96
4793\H,0,-1.175622,1.913014,-0.392034\H,0,-3.986102,2.322114,0.837738\
H,0,-4.357322,5.433646,-0.525451\H,0,-5.298499,4.026048,0.042015\H,0,-
5.073996,4.327629,-1.703318\H,0,-1.379493,3.383338,-1.72212\H,0,-2.037
26,5.034108,-1.601501\H,0,-2.734112,3.893895,-2.757632\H,0,-1.77819,-0
.535975,3.594104\H,0,-0.278669,0.378998,3.900692\H,0,1.293573,0.941767
,1.938647\H,0,0.746504,-1.575943,2.923021\H,0,0.658735,-1.15917,-0.209
346\H,0,2.422085,-2.031134,-1.44672\H,0,4.758881,-2.03311,-2.281054\H,
0,3.254113,1.330113,1.137387\H,0,7.209655,-0.638492,-1.940487\H,0,8.10
813,1.286621,-0.611061\H,0,6.104336,2.256504,0.933869\\Version=ES64L-G
09RevD.01\State=1-A\HF=-1190.8584798\RMSD=9.795e-09\PG=C01 [X(C26H26N2
O1)]\\@
```

6.5.2 Conformation 2 (internal code: M0002): rel. energy 0.00 kcal mol^{-1}


```
1\1\GINC-HEISENBERG01\SP\RwB97XD TD-FC\tApr-CC-pVDZ/Auto\C26H26N2O1\MF
RESIA\17-May-2020\0\\# td=(nstates=60) wB97XD/TApr-cc-pVDZ/auto scrf=(
solvent=methanol) geom=connectivity\\RaputindolD_M0002_td_wB97XD_TApr-
cc-pVDZ_methanol\\0,1\C,0,1.575795,0.704416,0.914774\C,0,2.27509,-0.01
6769,-0.082182\C,0,2.941155,0.617513,-1.113934\C,0,2.887308,2.020662,-
1.125683\C,0,2.194674,2.767094,-0.135526\C,0,1.524804,2.087496,0.90235
9\N,0,3.446888,2.919861,-2.001239\C,0,3.13492,4.199185,-1.603693\C,0,2
.37108,4.156374,-0.466466\C,0,0.934642,-0.262082,1.898744\C,0,1.772341
,-1.544859,1.662384\C,0,2.165397,-1.510735,0.170442\C,0,3.415111,-2.27
9089,-0.146009\C,0,3.594489,-3.23,-1.075108\C,0,4.937423,-3.891531,-1.
250629\C,0,2.528254,-3.732529,-2.012163\C,0,1.026398,0.185442,3.367214
\C,0,-0.525374,-0.54408,1.587508\O,0,0.248866,1.330855,3.653459\C,0,-1
.28807,0.10312,0.695833\C,0,-2.709198,-0.149296,0.388793\C,0,-3.510669
,-1.028443,1.166722\C,0,-4.839148,-1.268311,0.866821\C,0,-5.388507,-0.
610676,-0.243858\C,0,-4.622956,0.27844,-1.038724\C,0,-3.276415,0.5022,
-0.705226\N,0,-6.654263,-0.651952,-0.772015\C,0,-6.715512,0.176549,-1.
86837\C,0,-5.496107,0.768627,-2.071481\H,0,3.490365,0.054277,-1.871668
\H,0,0.990557,2.629432,1.68359\H,0,4.001966,2.682058,-2.810411\H,0,3.4
89602,5.051967,-2.176327\H,0,1.97741,5.014233,0.072015\H,0,2.68921,-1.
491672,2.275338\H,0,1.235651,-2.463118,1.941614\H,0,1.318684,-1.894944
,-0.421238\H,0,4.282646,-1.997317,0.465541\H,0,4.855384,-4.981824,-1.1
04059\H,0,5.684155,-3.502755,-0.543847\H,0,5.316917,-3.740698,-2.27532
4\H,0,1.557987,-3.23848,-1.878595\H,0,2.381802,-4.817171,-1.877158\H,0
,2.843027,-3.587004,-3.058917\H,0,2.069325,0.443269,3.606695\H,0,0.733
089,-0.664587,4.015549\H,0,-0.955202,-1.358258,2.184197\H,0,-0.620055,
1.184812,3.253025\H,0,-0.822222,0.89835,0.104157\H,0,-3.075559,-1.5251
76,2.035199\H,0,-5.441079,-1.941992,1.478757\H,0,-2.669308,1.18339,-1.
30691\H,0,-7.422795,-1.201594,-0.415039\H,0,-7.643729,0.279007,-2.4239
45\H,0,-5.248679,1.471278,-2.862376\\Version=ES64L-G09RevD.01\State=1-
A\HF=-1190.859047\RMSD=3.222e-09\PG=C01 [X(C26H26N2O1)]\\@
```

6.5.3 Conformation 3 (internal code: M0006): rel. energy 1.67 $\mathrm{kcal\,mol^{-1}}$

1\1\GINC-HEISENBERG03\SP\RwB97XD TD-FC\tApr-CC-pVDZ/Auto\C26H26N2O1\MF
RESIA\17-May-2020\0\\# td=(nstates=60) wB97XD/TApr-cc-pVDZ/auto scrf=(
solvent=methanol) geom=connectivity\\RaputindolD_M0006_td_wB97XD_TApr-
cc-pVDZ_methanol\\0,1\C,0,-1.896954,-0.877803,0.713686\C,0,-2.386952,0
.185762,-0.081351\C,0,-3.345604,-0.014238,-1.056497\C,0,-3.807681,-1.3
29873,-1.222386\C,0,-3.328459,-2.413868,-0.439544\C,0,-2.348598,-2.174
674,0.545972\N,0,-4.745631,-1.834243,-2.090643\C,0,-4.875356,-3.188127
,-1.886962\C,0,-4.029309,-3.588396,-0.885484\C,0,-0.830767,-0.360097,1
.674029\C,0,-1.141441,1.152249,1.693083\C,0,-1.700323,1.48737,0.294596
\C,0,-2.607494,2.682892,0.275464\C,0,-2.542471,3.763114,-0.517264\C,0,
-3.558933,4.869419,-0.396438\C,0,-1.496548,3.99264,-1.576072\C,0,-0.93
7729,-0.982714,3.074645\C,0,0.531153,-0.716357,1.103205\O,0,-0.037941,
-0.39008,3.987998\C,0,1.539133,0.119802,0.818577\C,0,2.860063,-0.23918
1,0.264706\C,0,3.198664,-1.574111,-0.087535\C,0,4.438789,-1.904606,-0.
601877\C,0,5.373562,-0.872723,-0.77188\C,0,5.072391,0.470393,-0.434863
\C,0,3.801863,0.772508,0.084769\N,0,6.658733,-0.900757,-1.251794\C,0,7
.178854,0.37225,-1.229383\C,0,6.244994,1.246934,-0.737455\H,0,-3.72773
6,0.811709,-1.660244\H,0,-1.966469,-2.997862,1.154173\H,0,-5.258993,-1
.297326,-2.774581\H,0,-5.572334,-3.767741,-2.486218\H,0,-3.91624,-4.60
152,-0.509038\H,0,-1.933287,1.327217,2.440652\H,0,-0.289853,1.781933,1
.980282\H,0,-0.855831,1.637751,-0.397423\H,0,-3.429531,2.63482,1.00177
7\H,0,-3.066437,5.826586,-0.155268\H,0,-4.307254,4.658904,0.380543\H,0
,-4.084779,5.02107,-1.354166\H,0,-0.755614,3.186605,-1.643684\H,0,-0.9
60107,4.936768,-1.383881\H,0,-1.974162,4.10101,-2.564223\H,0,-0.778243
,-2.07687,3.009938\H,0,-1.950201,-0.818216,3.474214\H,0,0.658008,-1.79
1366,0.928319\H,0,0.835623,-0.416655,3.572853\H,0,1.402447,1.191117,0.
996663\H,0,2.462547,-2.368515,0.043836\H,0,4.679451,-2.935444,-0.86697
2\H,0,3.551449,1.803222,0.349023\H,0,7.148863,-1.723682,-1.572191\H,0,
8.193491,0.553777,-1.573305\H,0,6.377924,2.316988,-0.604468\\Version=E
S64L-G09RevD.01\State=1-A\HF=-1190.8564554\RMSD=3.117e-09\PG=C01 [X(C2
6H26N2O1)]\\@

6.5.4 Conformation 4 (internal code: M0007): rel. energy 0.51 kcal mol^{-1}


```
1\1\GINC-HEISENBERG03\SP\RwB97XD TD-FC\tApr-CC-pVDZ/Auto\C26H26N2O1\MF
RESIA\17-May-2020\0\\# td=(nstates=60) wB97XD/TApr-cc-pVDZ/auto scrf=(
solvent=methanol) geom=connectivity\\RaputindolD_M0007_td_wB97XD_TApr-
cc-pVDZ_methanol\\0,1\C,0,-1.556286,-0.69346,0.847979\C,0,-2.215153,0.
14215,-0.083575\C,0,-3.053426,-0.367393,-1.057099\C,0,-3.218828,-1.761
609,-1.075959\C,0,-2.56757,-2.622931,-0.15264\C,0,-1.717735,-2.068292,
0.826149\N,0,-3.981979,-2.549844,-1.902612\C,0,-3.836512,-3.868373,-1.
539527\C,0,-2.980949,-3.962793,-0.472846\C,0,-0.700447,0.139947,1.7906
83\C,0,-1.323336,1.549581,1.608158\C,0,-1.842915,1.595343,0.156677\C,0
,-2.972536,2.560659,-0.055706\C,0,-3.099201,3.491001,-1.013901\C,0,-4.
31941,4.37364,-1.074722\C,0,-2.085338,3.754794,-2.0957\C,0,-0.806657,-
0.339367,3.250574\C,0,0.763231,0.166411,1.402448\O,0,-2.131051,-0.3615
64,3.737286\C,0,1.323092,-0.460822,0.36051\C,0,2.745333,-0.416173,-0.0
37032\C,0,3.200665,-1.365764,-0.98939\C,0,4.517707,-1.416123,-1.415569
\C,0,5.408279,-0.480818,-0.874601\C,0,4.988406,0.490898,0.070877\C,0,3
.646555,0.514634,0.480598\N,0,6.747647,-0.283827,-1.102565\C,0,7.18726
8,0.772266,-0.338541\C,0,6.147822,1.280343,0.395393\H,0,-3.568208,0.28
4414,-1.766258\H,0,-1.201784,-2.714159,1.541073\H,0,-4.560181,-2.21607
1,-2.660275\H,0,-4.364895,-4.647181,-2.08295\H,0,-2.679576,-4.879444,0
.026778\H,0,-2.176229,1.633495,2.300818\H,0,-0.613113,2.358233,1.83432
1\H,0,-1.000638,1.841497,-0.510376\H,0,-3.792791,2.460599,0.667251\H,0
,-4.037277,5.436488,-0.986648\H,0,-5.037674,4.13892,-0.276567\H,0,-4.8
31093,4.264679,-2.04584\H,0,-1.210638,3.094448,-2.047404\H,0,-1.73181,
4.798178,-2.043557\H,0,-2.549984,3.631027,-3.088168\H,0,-0.247545,0.35
0127,3.902306\H,0,-0.32724,-1.33347,3.339677\H,0,1.386712,0.782336,2.0
62671\H,0,-2.64218,-0.914967,3.130989\H,0,0.68362,-1.088072,-0.270022\
H,0,2.483858,-2.084384,-1.394067\H,0,4.847183,-2.15572,-2.146967\H,0,3
.316291,1.27165,1.194801\H,0,7.322812,-0.827498,-1.72975\H,0,8.228299,
1.081132,-0.381814\H,0,6.20044,2.11544,1.088497\\Version=ES64L-G09RevD
.01\State=1-A\HF=-1190.8582672\RMSD=2.843e-09\PG=C01 [X(C26H26N2O1)]\\
@
```

6.5.5 Conformation 5 (internal code: M0008): rel. energy 0.15 kcal mol^{-1}

1\1\GINC-HEISENBERG02\SP\RwB97XD TD-FC\tApr-CC-pVDZ/Auto\C26H26N2O1\MF
RESIA\17-May-2020\0\\# td=(nstates=60) wB97XD/TApr-cc-pVDZ/auto scrf=(
solvent=methanol) geom=connectivity\\RaputindolD_M0008_td_wB97XD_TApr-
cc-pVDZ_methanol\\0,1\C,0,1.538109,0.686045,0.898533\C,0,2.160444,-0.0
0101,-0.16956\C,0,2.782935,0.670367,-1.205133\C,0,2.769252,2.07314,-1.
141744\C,0,2.153985,2.786295,-0.078179\C,0,1.523935,2.069021,0.959015\
N,0,3.303948,3.001286,-2.001817\C,0,3.050568,4.265926,-1.524507\C,0,2.
35029,4.185203,-0.34902\C,0,0.925904,-0.305247,1.877636\C,0,1.693764,-
1.605115,1.518523\C,0,2.012607,-1.501744,0.01232\C,0,3.219069,-2.29016
,-0.40623\C,0,3.313074,-3.21795,-1.370615\C,0,4.622639,-3.910724,-1.64
8198\C,0,2.175182,-3.665291,-2.249675\C,0,1.160504,0.103864,3.343379\C
,0,-0.560784,-0.507264,1.666089\O,0,2.523918,0.280168,3.663055\C,0,-1.
313116,0.082445,0.728452\C,0,-2.754095,-0.117249,0.480131\C,0,-3.56312
6,-0.947337,1.303185\C,0,-4.911482,-1.133433,1.058142\C,0,-5.474615,-0
.471285,-0.042885\C,0,-4.701613,0.366726,-0.884186\C,0,-3.334375,0.535
244,-0.606569\N,0,-6.760592,-0.466141,-0.52241\C,0,-6.827229,0.343057,
-1.632569\C,0,-5.591295,0.875348,-1.893798\H,0,3.269045,0.135686,-2.02
4135\H,0,1.036776,2.598236,1.781969\H,0,3.80675,2.790508,-2.851814\H,0
,3.397046,5.137564,-2.073202\H,0,2.012023,5.023791,0.25356\H,0,2.63726
9,-1.609472,2.087635\H,0,1.131428,-2.515223,1.773851\H,0,1.126606,-1.8
25119,-0.557849\H,0,4.12991,-2.051467,0.158736\H,0,4.520725,-5.001327,
-1.516926\H,0,5.424793,-3.555842,-0.985855\H,0,4.936263,-3.748603,-2.6
93213\H,0,1.229642,-3.149807,-2.040881\H,0,2.007013,-4.748948,-2.13298
9\H,0,2.425607,-3.499987,-3.310798\H,0,0.786246,-0.689402,4.009219\H,0
,0.574295,1.017668,3.561306\H,0,-1.012508,-1.230907,2.355536\H,0,2.876
832,0.925647,3.035439\H,0,-0.825991,0.790717,0.049818\H,0,-3.116209,-1
.453554,2.160126\H,0,-5.517275,-1.772967,1.702246\H,0,-2.721625,1.1773
24,-1.244647\H,0,-7.538478,-0.972358,-0.124115\H,0,-7.770645,0.476423,
-2.155057\H,0,-5.343578,1.550823,-2.708024\\Version=ES64L-G09RevD.01\S
tate=1-A\HF=-1190.8588421\RMSD=3.231e-09\PG=C01 [X(C26H26N2O1)]\\@

6.5.6 Conformation 6 (internal code: M0010): rel. energy 1.75 kcal mol^{-1}

1\1\GINC-HEISENBERG01\SP\RwB97XD TD-FC\tApr-CC-pVDZ/Auto\C26H26N2O1\MF
RESIA\17-May-2020\0\\# td=(nstates=60) wB97XD/TApr-cc-pVDZ/auto scrf=(
solvent=methanol) geom=connectivity\\RaputindolD_M0010_td_wB97XD_TApr-
cc-pVDZ_methanol\\0,1\C,0,-1.556124,-0.773993,0.603233\C,0,-2.470446,0
.020385,-0.124904\C,0,-3.435419,-0.541983,-0.940443\C,0,-3.461237,-1.9
43642,-1.008741\C,0,-2.551021,-2.763222,-0.287642\C,0,-1.575922,-2.156
515,0.528786\N,0,-4.293692,-2.778614,-1.713555\C,0,-3.944875,-4.085971
,-1.467406\C,0,-2.884711,-4.127102,-0.599818\C,0,-0.593626,0.107482,1.
402012\C,0,-1.348534,1.464903,1.399558\C,0,-2.202694,1.496346,0.113038
\C,0,-3.449047,2.324353,0.237045\C,0,-3.860266,3.333659,-0.544984\C,0,
-5.157756,4.04649,-0.261863\C,0,-3.11261,3.857339,-1.742531\C,0,-0.391
303,-0.421959,2.827988\C,0,0.701387,0.224044,0.629013\O,0,-1.605157,-0
.560305,3.536985\C,0,1.934591,-0.071613,1.061153\C,0,3.18677,0.040412,
0.284709\C,0,3.189965,0.293278,-1.114321\C,0,4.363272,0.406305,-1.8375
6\C,0,5.574913,0.261254,-1.146458\C,0,5.612937,0.000375,0.245913\C,0,4
.401751,-0.111079,0.950744\N,0,6.871832,0.320355,-1.592371\C,0,7.72457
6,0.107625,-0.534849\C,0,7.001071,-0.093313,0.612225\H,0,-4.144762,0.0
75119,-1.496074\H,0,-0.860198,-2.767176,1.085126\H,0,-5.043283,-2.4817
34,-2.321641\H,0,-4.491299,-4.896999,-1.941327\H,0,-2.397509,-5.023587
,-0.226241\H,0,-2.019894,1.472326,2.272709\H,0,-0.666981,2.323741,1.47
8629\H,0,-1.585,1.868796,-0.720264\H,0,-4.094931,2.036921,1.077286\H,0
,-4.981731,5.121334,-0.086959\H,0,-5.671162,3.632197,0.617201\H,0,-5.8
38782,3.97748,-1.126905\H,0,-2.163798,3.339593,-1.929853\H,0,-2.897061
,4.931351,-1.615769\H,0,-3.734507,3.76833,-2.648872\H,0,0.224657,0.284
405,3.405039\H,0,0.155024,-1.383311,2.793282\H,0,0.569526,0.596587,-0.
393471\H,0,-2.179963,-1.122152,2.999053\H,0,2.075268,-0.426328,2.08699
7\H,0,2.241412,0.390908,-1.644364\H,0,4.344376,0.595263,-2.912205\H,0,
4.411918,-0.312118,2.02517\H,0,7.159081,0.492253,-2.545226\H,0,8.80010
5,0.11769,-0.689805\H,0,7.40992,-0.28378,1.600709\\Version=ES64L-G09Re
vD.01\State=1-A\HF=-1190.8560854\RMSD=3.165e-09\PG=C01 [X(C26H26N2O1)]
\\@

6.5.7 Conformation 7 (internal code: M0014): rel. energy 1.29 kcal mol^{-1}

1\1\GINC-HEISENBERG03\SP\RwB97XD TD-FC\tApr-CC-pVDZ/Auto\C26H26N2O1\MF
RESIA\17-May-2020\0\\# td=(nstates=60) wB97XD/TApr-cc-pVDZ/auto scrf=(
solvent=methanol) geom=connectivity\\RaputindolD_M0014_td_wB97XD_TApr-
cc-pVDZ_methanol\\0,1\C,0,-1.613318,-0.865585,0.74593\C,0,-2.235605,0.
055252,-0.129186\C,0,-3.082018,-0.359298,-1.139603\C,0,-3.29266,-1.742
887,-1.255174\C,0,-2.680246,-2.687229,-0.388211\C,0,-1.819636,-2.22965
9,0.631054\N,0,-4.074811,-2.445632,-2.139102\C,0,-3.979127,-3.789799,-
1.865705\C,0,-3.137848,-3.986728,-0.801472\C,0,-0.717846,-0.122513,1.7
40228\C,0,-1.289544,1.307214,1.65204\C,0,-1.820096,1.47558,0.212996\C,
0,-2.925479,2.482935,0.084447\C,0,-3.032444,3.486852,-0.79888\C,0,-4.2
30257,4.401536,-0.785201\C,0,-2.017908,3.806451,-1.864942\C,0,-0.83498
9,-0.698667,3.163686\C,0,0.71618,-0.269586,1.273895\O,0,-2.161512,-0.7
34278,3.643765\C,0,1.533527,0.705913,0.857153\C,0,2.919213,0.556712,0.
366257\C,0,3.657431,1.740717,0.103064\C,0,4.964129,1.715008,-0.355719\
C,0,5.552064,0.461185,-0.560641\C,0,4.84479,-0.744731,-0.314522\C,0,3.
5219,-0.682812,0.149159\N,0,6.806203,0.121775,-1.004036\C,0,6.916217,-
1.24878,-1.047511\C,0,5.742567,-1.823469,-0.63574\H,0,-3.56933,0.35561
9,-1.806103\H,0,-1.335851,-2.93976,1.306925\H,0,-4.63171,-2.040963,-2.
877976\H,0,-4.529564,-4.510022,-2.464946\H,0,-2.874554,-4.945087,-0.36
2316\H,0,-2.139493,1.358313,2.350768\H,0,-0.572604,2.086502,1.944007\H
,0,-0.979708,1.749256,-0.445371\H,0,-3.743557,2.348979,0.804412\H,0,-3
.920374,5.448388,-0.627096\H,0,-4.946942,4.129734,0.002489\H,0,-4.7532
93,4.373013,-1.755922\H,0,-1.161159,3.12138,-1.874617\H,0,-1.635538,4.
832709,-1.735052\H,0,-2.491183,3.772275,-2.860277\H,0,-0.265853,-0.060
761,3.857266\H,0,-0.372629,-1.704607,3.191243\H,0,1.073274,-1.306723,1
.271887\H,0,-2.679845,-1.232682,2.996878\H,0,1.169927,1.738315,0.86765
3\H,0,3.174008,2.706201,0.2702\H,0,5.51174,2.6382,-0.551575\H,0,2.9741
7,-1.610135,0.328341\H,0,7.534394,0.772481,-1.26151\H,0,7.842696,-1.71
2172,-1.375825\H,0,5.541341,-2.888898,-0.566044\\Version=ES64L-G09RevD
.01\State=1-A\HF=-1190.8568433\RMSD=2.808e-09\PG=C01 [X(C26H26N2O1)]\\
@

6.5.8 Conformation 8 (internal code: M0015): rel. energy 1.11 kcal mol^{-1}


```
1\1\GINC-HEISENBERG02\SP\RwB97XD TD-FC\tApr-CC-pVDZ/Auto\C26H26N2O1\MF
RESIA\17-May-2020\0\\# td=(nstates=60) wB97XD/TApr-cc-pVDZ/auto scrf=(
solvent=methanol) geom=connectivity\\RaputindolD_M0015_td_wB97XD_TApr-
cc-pVDZ_methanol\\0,1\C,0,-1.793745,-0.857416,0.667932\C,0,-2.262168,0
.216336,-0.124783\C,0,-3.17243,0.023679,-1.146686\C,0,-3.607867,-1.294
102,-1.36068\C,0,-3.152257,-2.38871,-0.577923\C,0,-2.22217,-2.156756,0
.456367\N,0,-4.498323,-1.79164,-2.28092\C,0,-4.620188,-3.150727,-2.110
547\C,0,-3.816355,-3.561783,-1.079108\C,0,-0.778972,-0.350057,1.694631
\C,0,-1.100442,1.157865,1.720427\C,0,-1.613966,1.51924,0.31093\C,0,-2.
543225,2.697938,0.289147\C,0,-2.496816,3.777078,-0.50633\C,0,-3.531981
,4.866197,-0.388761\C,0,-1.454498,4.020644,-1.565539\C,0,-0.980614,-1.
002251,3.075388\C,0,0.606153,-0.704674,1.19369\O,0,-2.288702,-0.846077
,3.58162\C,0,1.587209,0.142016,0.856089\C,0,2.926429,-0.215014,0.34618
8\C,0,3.280692,-1.551302,0.014787\C,0,4.539657,-1.882757,-0.451418\C,0
,5.478132,-0.850265,-0.593871\C,0,5.160873,0.494878,-0.28068\C,0,3.870
983,0.797965,0.188672\N,0,6.780747,-0.878842,-1.025088\C,0,7.29635,0.3
95725,-0.994227\C,0,6.342154,1.272068,-0.54601\H,0,-3.539646,0.857138,
-1.749458\H,0,-1.856456,-2.986066,1.067555\H,0,-4.985582,-1.247163,-2.
977924\H,0,-5.281695,-3.72613,-2.752539\H,0,-3.707732,-4.581043,-0.718
408\H,0,-1.919626,1.300972,2.442553\H,0,-0.257808,1.782644,2.046833\H,
0,-0.749283,1.703662,-0.347157\H,0,-3.365275,2.635816,1.014353\H,0,-3.
05557,5.832145,-0.150238\H,0,-4.276551,4.645035,0.388843\H,0,-4.06034,
5.006474,-1.346848\H,0,-0.701247,3.225892,-1.63093\H,0,-0.933227,4.973
956,-1.376873\H,0,-1.933863,4.11779,-2.554017\H,0,-0.303317,-0.52591,3
.800823\H,0,-0.699759,-2.072078,3.018981\H,0,0.771138,-1.784192,1.0950
64\H,0,-2.891607,-1.197921,2.912143\H,0,1.418806,1.218272,0.963014\H,0
,2.539813,-2.3453,0.119754\H,0,4.791079,-2.914621,-0.702508\H,0,3.6085
51,1.830346,0.434318\H,0,7.28525,-1.703287,-1.317776\H,0,8.323351,0.57
7103,-1.299401\H,0,6.467615,2.343561,-0.41717\\Version=ES64L-G09RevD.0
1\State=1-A\HF=-1190.8574008\RMSD=3.169e-09\PG=C01 [X(C26H26N2O1)]\\@
```

6.5.9 Conformation 9 (internal code: M0022): rel. energy 1.38 kcal mol^{-1}

1\1\GINC-HEISENBERG02\SP\RwB97XD TD-FC\tApr-CC-pVDZ/Auto\C26H26N2O1\MF
RESIA\17-May-2020\0\\# td=(nstates=60) wB97XD/TApr-cc-pVDZ/auto scrf=(
solvent=methanol) geom=connectivity\\RaputindolD_M0022_td_wB97XD_TApr-
cc-pVDZ_methanol\\0,1\C,0,-1.614952,-0.686274,0.954544\C,0,-2.302161,0
.059594,-0.030818\C,0,-3.152473,-0.538446,-0.942011\C,0,-3.299809,-1.9
30864,-0.840766\C,0,-2.616589,-2.703515,0.136514\C,0,-1.754615,-2.0597
01,1.047165\N,0,-4.06521,-2.795422,-1.585587\C,0,-3.890393,-4.077476,-
1.119374\C,0,-3.013963,-4.071463,-0.065753\C,0,-0.742222,0.227934,1.79
6682\C,0,-1.379068,1.618387,1.50633\C,0,-1.935394,1.529714,0.069667\C,
0,-3.075312,2.468112,-0.199115\C,0,-3.200688,3.35777,-1.195383\C,0,-4.
431358,4.220664,-1.306664\C,0,-2.175074,3.595563,-2.272079\C,0,-0.8208
35,-0.117041,3.294787\C,0,0.70942,0.235369,1.360964\O,0,-0.101237,0.78
6314,4.11145\C,0,1.215082,-0.362888,0.275577\C,0,2.622553,-0.345442,-0
.174393\C,0,2.933666,-0.99312,-1.39895\C,0,4.219202,-1.03275,-1.913804
\C,0,5.229769,-0.405211,-1.175725\C,0,4.959289,0.249005,0.054733\C,0,3
.645366,0.27224,0.546255\N,0,6.57515,-0.282467,-1.421832\C,0,7.161741,
0.420898,-0.395401\C,0,6.214678,0.768258,0.531755\H,0,-3.685711,0.0456
04,-1.695164\H,0,-1.212904,-2.639441,1.798018\H,0,-4.661444,-2.534128,
-2.357579\H,0,-4.414409,-4.906549,-1.587394\H,0,-2.687524,-4.940013,0.
499856\H,0,-2.220906,1.780868,2.203408\H,0,-0.671336,2.450736,1.630623
\H,0,-1.110388,1.719914,-0.635635\H,0,-3.903954,2.386863,0.516628\H,0,
-4.163977,5.289703,-1.254127\H,0,-5.158643,4.006381,-0.510939\H,0,-4.9
26982,4.069571,-2.280471\H,0,-1.292125,2.950044,-2.188325\H,0,-1.83598
2,4.644842,-2.251625\H,0,-2.624292,3.431769,-3.265797\H,0,-0.371773,-1
.106037,3.473309\H,0,-1.885098,-0.1729,3.59222\H,0,1.35792,0.814399,2.
027122\H,0,-0.535911,1.645602,4.05064\H,0,0.534584,-0.932736,-0.366382
\H,0,2.125379,-1.476234,-1.953266\H,0,4.434109,-1.535261,-2.858195\H,0
,3.436061,0.772745,1.493721\H,0,7.06124,-0.651872,-2.226266\H,0,8.2300
51,0.619626,-0.410624\H,0,6.389171,1.325307,1.448156\\Version=ES64L-G0
9RevD.01\State=1-A\HF=-1190.8563611\RMSD=2.826e-09\PG=C01 [X(C26H26N2O
1)]\\@

6.5.10 Conformation 10 (internal code: M0023): rel. energy 1.44 kcal mol^{-1}

1\1\GINC-HEISENBERG02\SP\RwB97XD TD-FC\tApr-CC-pVDZ/Auto\C26H26N2O1\MF
RESIA\17-May-2020\0\\# td=(nstates=60) wB97XD/TApr-cc-pVDZ/auto scrf=(
solvent=methanol) geom=connectivity\\RaputindolD_M0023_td_wB97XD_TApr-
cc-pVDZ_methanol\\0,1\C,0,1.636616,0.660334,0.993102\C,0,2.22939,0.065
73,-0.145174\C,0,2.875819,0.815978,-1.109575\C,0,2.915867,2.203804,-0.
901399\C,0,2.324627,2.825406,0.231034\C,0,1.668467,2.029081,1.191432\N
,0,3.484048,3.195799,-1.663602\C,0,3.27364,4.413362,-1.059502\C,0,2.56
956,4.237419,0.103219\C,0,0.984642,-0.400837,1.863053\C,0,1.70266,-1.6
88641,1.365061\C,0,2.015659,-1.437036,-0.125118\C,0,3.177537,-2.232684
,-0.643226\C,0,3.196039,-3.109426,-1.658445\C,0,4.467585,-3.825228,-2.
036115\C,0,2.003486,-3.479752,-2.50029\C,0,1.242464,-0.161572,3.360906
\C,0,-0.506721,-0.532607,1.623892\O,0,0.732879,-1.192826,4.184665\C,0,
-1.205705,0.063723,0.650052\C,0,-2.649065,-0.078298,0.374347\C,0,-3.49
7147,-0.902763,1.163113\C,0,-4.848174,-1.033493,0.898269\C,0,-5.374266
,-0.319847,-0.188809\C,0,-4.56157,0.511794,-0.998261\C,0,-3.192035,0.6
22403,-0.701294\N,0,-6.653703,-0.254695,-0.682007\C,0,-6.677952,0.5863
17,-1.76993\C,0,-5.420334,1.079853,-2.002932\H,0,3.3371,0.350675,-1.98
3252\H,0,1.199173,2.491031,2.063045\H,0,3.976605,3.056549,-2.53412\H,0
,3.651333,5.323559,-1.517518\H,0,2.260501,5.020876,0.789851\H,0,2.6563
93,-1.804109,1.911186\H,0,1.110808,-2.60242,1.519587\H,0,1.109322,-1.6
57261,-0.711786\H,0,4.120705,-2.0527,-0.110717\H,0,4.34052,-4.917138,-
1.943343\H,0,5.315443,-3.520572,-1.406402\H,0,4.727386,-3.628367,-3.08
9881\H,0,1.086381,-2.944602,-2.224788\H,0,1.804673,-4.561566,-2.420624
\H,0,2.21054,-3.277613,-3.564342\H,0,0.733375,0.759044,3.684692\H,0,2.
327993,-0.017301,3.518313\H,0,-0.999829,-1.207974,2.330772\H,0,1.23318
5,-1.995406,3.993456\H,0,-0.672465,0.73433,-0.032624\H,0,-3.076125,-1.
452897,2.00583\H,0,-5.482804,-1.671169,1.515977\H,0,-2.549441,1.258938
,-1.31521\H,0,-7.454074,-0.742699,-0.306108\H,0,-7.609631,0.767793,-2.
298954\H,0,-5.139057,1.767776,-2.795559\\Version=ES64L-G09RevD.01\Stat
e=1-A\HF=-1190.8570328\RMSD=3.204e-09\PG=C01 [X(C26H26N2O1)]\\@

6.5.11 Conformation 11 (internal code: M0037): rel. energy 1.71 kcal mol^{-1}

1\1\GINC-HEISENBERG01\SP\RwB97XD TD-FC\tApr-CC-pVDZ/Auto\C26H26N2O1\MF
RESIA\17-May-2020\0\\# td=(nstates=60) wB97XD/TApr-cc-pVDZ/auto scrf=(
solvent=methanol) geom=connectivity\\RaputindolD_M0037_td_wB97XD_TApr-
cc-pVDZ_methanol\\0,1\C,0,-1.627477,-0.745902,0.901856\C,0,-2.229001,0
.070668,-0.083814\C,0,-2.888573,-0.463802,-1.175003\C,0,-2.929027,-1.8
64676,-1.257736\C,0,-2.331225,-2.706066,-0.281383\C,0,-1.663731,-2.126
606,0.81665\N,0,-3.506493,-2.678556,-2.202879\C,0,-3.295341,-3.994689,
-1.86313\C,0,-2.582211,-4.06179,-0.694657\C,0,-0.964565,0.118336,1.963
987\C,0,-1.68971,1.476951,1.750798\C,0,-2.019972,1.538781,0.245173\C,0
,-3.194771,2.410975,-0.088807\C,0,-3.265878,3.396416,-0.996258\C,0,-4.
550206,4.15571,-1.210151\C,0,-2.127167,3.841222,-1.875518\C,0,-1.19391
3,-0.373577,3.394376\C,0,0.521863,0.310148,1.727626\O,0,-0.444231,-1.5
54457,3.611417\C,0,1.213509,-0.107489,0.660768\C,0,2.646776,0.112561,0
.383306\C,0,3.471228,0.914702,1.21885\C,0,4.814415,1.112235,0.954486\C
,0,5.356761,0.491813,-0.180607\C,0,4.566869,-0.311446,-1.039683\C,0,3.
204731,-0.490761,-0.742734\N,0,6.633236,0.507088,-0.685561\C,0,6.67758
1,-0.256619,-1.828472\C,0,5.43635,-0.778345,-2.086552\H,0,-3.3611,0.17
3363,-1.925715\H,0,-1.195132,-2.739342,1.587658\H,0,-4.006109,-2.36294
2,-3.021691\H,0,-3.677436,-4.790785,-2.496566\H,0,-2.269645,-4.9706,-0
.187388\H,0,-2.633244,1.473546,2.32447\H,0,-1.092048,2.334321,2.09274\
H,0,-1.122452,1.882376,-0.293956\H,0,-4.105523,2.177334,0.478413\H,0,-
4.399466,5.234353,-1.035199\H,0,-5.351494,3.805231,-0.544409\H,0,-4.89
489,4.052434,-2.252949\H,0,-1.193674,3.293806,-1.695862\H,0,-1.92844,4
.915705,-1.727414\H,0,-2.395403,3.716136,-2.93787\H,0,-2.275681,-0.549
714,3.541457\H,0,-0.884382,0.423569,4.099051\H,0,1.024728,0.881821,2.5
16895\H,0,-0.648504,-1.872328,4.49811\H,0,0.681688,-0.685906,-0.102399
\H,0,3.038474,1.397909,2.096088\H,0,5.430471,1.733553,1.606713\H,0,2.5
79789,-1.107699,-1.393724\H,0,7.418979,0.997211,-0.282634\H,0,7.610628
,-0.369173,-2.373976\H,0,5.172626,-1.420746,-2.922313\\Version=ES64L-G
09RevD.01\State=1-A\HF=-1190.8557519\RMSD=3.136e-09\PG=C01 [X(C26H26N2
O1)]\\@

6.5.12 Conformation 12 (internal code: M0039): rel. energy 1.59 kcal mol^{-1}

1\1\GINC-HEISENBERG01\SP\RwB97XD TD-FC\tApr-CC-pVDZ/Auto\C26H26N2O1\MF
RESIA\17-May-2020\0\\# td=(nstates=60) wB97XD/TApr-cc-pVDZ/auto scrf=(
solvent=methanol) geom=connectivity\\RaputindolD_M0039_td_wB97XD_TApr-
cc-pVDZ_methanol\\0,1\C,0,1.475159,0.2976,0.710327\C,0,2.11313,-0.1696
9,-0.463226\C,0,2.881442,0.661176,-1.256803\C,0,3.004205,1.994387,-0.8
35638\C,0,2.382821,2.484674,0.343669\C,0,1.600249,1.612345,1.126326\N,
0,3.699781,3.038166,-1.397367\C,0,3.541078,4.163222,-0.621461\C,0,2.74
4255,3.873373,0.455216\C,0,0.679117,-0.827236,1.354887\C,0,1.344967,-2
.081472,0.733072\C,0,1.809074,-1.642465,-0.675052\C,0,2.889737,-2.4970
93,-1.297133\C,0,4.175987,-2.599013,-0.92652\C,0,5.116952,-3.520614,-1
.659166\C,0,4.80972,-1.847358,0.214665\C,0,0.778644,-0.865575,2.889141
\C,0,-0.793564,-0.798513,0.981856\O,0,0.142813,0.228741,3.519197\C,0,-
1.433867,0.190116,0.343077\C,0,-2.863629,0.236879,-0.024131\C,0,-3.360
036,1.437727,-0.596019\C,0,-4.684547,1.588675,-0.972079\C,0,-5.540668,
0.499156,-0.771328\C,0,-5.080045,-0.719528,-0.206925\C,0,-3.732427,-0.
838816,0.163073\N,0,-6.877864,0.349086,-1.042024\C,0,-7.277225,-0.9141
28,-0.670881\C,0,-6.213245,-1.606032,-0.155209\H,0,3.388472,0.290751,-
2.150116\H,0,1.113525,1.952497,2.041036\H,0,4.243691,2.990007,-2.24670
1\H,0,4.018904,5.096596,-0.906898\H,0,2.446749,4.568173,1.23603\H,0,2.
225067,-2.361689,1.334896\H,0,0.670822,-2.949858,0.706951\H,0,0.930864
,-1.694985,-1.342702\H,0,2.566899,-3.116319,-2.140818\H,0,5.972796,-2.
958094,-2.069076\H,0,4.620223,-4.046677,-2.486768\H,0,5.538051,-4.2734
65,-0.971358\H,0,4.116283,-1.179746,0.73825\H,0,5.652586,-1.238175,-0.
153506\H,0,5.23101,-2.558165,0.945652\H,0,1.837501,-0.830359,3.187995\
H,0,0.359261,-1.826824,3.248364\H,0,-1.348423,-1.689541,1.30225\H,0,-0
.723186,0.326814,3.098233\H,0,-0.851482,1.069674,0.048309\H,0,-2.66832
1,2.270647,-0.742137\H,0,-5.045503,2.520573,-1.409984\H,0,-3.374252,-1
.777894,0.589541\H,0,-7.477622,1.052302,-1.44902\H,0,-8.310273,-1.2220
27,-0.80823\H,0,-6.233259,-2.625705,0.219535\\Version=ES64L-G09RevD.01
\State=1-A\HF=-1190.8562255\RMSD=9.896e-09\PG=C01 [X(C26H26N2O1)]\\@

6.5.13 Conformation 13 (internal code: M0042): rel. energy 1.52 kcal mol^{-1}

1\1\GINC-HEISENBERG01\SP\RwB97XD TD-FC\tApr-CC-pVDZ/Auto\C26H26N2O1\MF
RESIA\17-May-2020\0\\# td=(nstates=60) wB97XD/TApr-cc-pVDZ/auto scrf=(
solvent=methanol) geom=connectivity\\RaputindolD_M0042_td_wB97XD_TApr-
cc-pVDZ_methanol\\0,1\C,0,1.441497,0.193392,0.749706\C,0,2.091435,-0.0
32138,-0.486883\C,0,2.688903,0.993802,-1.194927\C,0,2.62519,2.273257,-
0.621455\C,0,1.989032,2.522783,0.623674\C,0,1.383492,1.455421,1.316317
\N,0,3.128359,3.470064,-1.072605\C,0,2.834653,4.460632,-0.164105\C,0,2
.139763,3.929065,0.890701\C,0,0.855087,-1.107248,1.27719\C,0,1.67647,-
2.163322,0.494873\C,0,2.009401,-1.501444,-0.862277\C,0,3.184245,-2.100
81,-1.601259\C,0,4.484422,-2.035665,-1.273158\C,0,5.528106,-2.708215,-
2.127541\C,0,5.038217,-1.317791,-0.070115\C,0,1.028852,-1.302615,2.793
154\C,0,-0.62062,-1.266971,0.952557\O,0,0.259289,-0.403767,3.566294\C,
0,-1.436147,-0.319252,0.470174\C,0,-2.872788,-0.446769,0.158752\C,0,-3
.593029,-1.655326,0.361727\C,0,-4.938939,-1.765731,0.064051\C,0,-5.590
043,-0.635628,-0.452655\C,0,-4.905916,0.586667,-0.667948\C,0,-3.538169
,0.66504,-0.355137\N,0,-6.895524,-0.450736,-0.832677\C,0,-7.060005,0.8
3992,-1.278677\C,0,-5.869486,1.514431,-1.19698\H,0,3.207424,0.811731,-
2.13848\H,0,0.892088,1.608289,2.277871\H,0,3.634134,3.604991,-1.936102
\H,0,3.150457,5.483882,-0.349062\H,0,1.77528,4.474385,1.757124\H,0,2.6
1349,-2.368899,1.037594\H,0,1.143271,-3.118324,0.381985\H,0,1.124143,-
1.617591,-1.512155\H,0,2.928489,-2.667776,-2.502773\H,0,6.264738,-1.97
2641,-2.492884\H,0,5.086387,-3.216105,-2.996477\H,0,6.093477,-3.45129,
-1.540074\H,0,4.269314,-0.85007,0.555133\H,0,5.739403,-0.528445,-0.390
455\H,0,5.618819,-2.018276,0.553494\H,0,2.081874,-1.13247,3.064895\H,0
,0.782654,-2.353925,3.044941\H,0,-1.013777,-2.26827,1.167596\H,0,-0.62
7878,-0.393718,3.179172\H,0,-1.008317,0.668364,0.267551\H,0,-3.077618,
-2.528374,0.764562\H,0,-5.474101,-2.702676,0.226109\H,0,-2.994054,1.59
946,-0.514309\H,0,-7.623959,-1.149293,-0.793358\H,0,-8.032473,1.179057
,-1.625485\H,0,-5.699137,2.549919,-1.478355\\Version=ES64L-G09RevD.01\
State=1-A\HF=-1190.8569589\RMSD=3.224e-09\PG=C01 [X(C26H26N2O1)]\\@

6.5.14 Conformation 14 (internal code: M0045): rel. energy 1.19 kcal mol^{-1}


```
1\1\GINC-HEISENBERG03\SP\RwB97XD TD-FC\tApr-CC-pVDZ/Auto\C26H26N2O1\MF
RESIA\17-May-2020\0\\# td=(nstates=60) wB97XD/TApr-cc-pVDZ/auto scrf=(
solvent=methanol) geom=connectivity\\RaputindolD_M0045_td_wB97XD_TApr-
cc-pVDZ_methanol\\0,1\C,0,1.380797,0.278532,0.657845\C,0,2.041002,-0.0
66411,-0.542231\C,0,2.814207,0.846677,-1.235448\C,0,2.917196,2.132473,
-0.683426\C,0,2.267556,2.502678,0.524915\C,0,1.483263,1.548478,1.20220
9\N,0,3.611633,3.231373,-1.128109\C,0,3.424752,4.275262,-0.252004\C,0,
2.61046,3.877203,0.775942\C,0,0.604614,-0.90861,1.207054\C,0,1.222932,
-2.097486,0.420392\C,0,1.746077,-1.509605,-0.911612\C,0,2.867797,-2.28
594,-1.564897\C,0,4.131801,-2.412564,-1.131111\C,0,5.136319,-3.223144,
-1.908824\C,0,4.67994,-1.780867,0.122278\C,0,0.824507,-1.0743,2.72313\
C,0,-0.883985,-0.826484,0.942694\O,0,2.189186,-1.155232,3.079727\C,0,-
1.517027,0.13396,0.257642\C,0,-2.963538,0.213908,-0.03137\C,0,-3.45985
2,1.416422,-0.6004\C,0,-4.800136,1.599601,-0.898283\C,0,-5.673674,0.54
1267,-0.620837\C,0,-5.214455,-0.678895,-0.059511\C,0,-3.850044,-0.8315
17,0.229515\N,0,-7.0289,0.426072,-0.807455\C,0,-7.44082,-0.817063,-0.3
86155\C,0,-6.367268,-1.53018,0.078846\H,0,3.337736,0.573033,-2.153677\
H,0,0.96915,1.812049,2.13007\H,0,4.170316,3.270453,-1.968349\H,0,3.897
621,5.236062,-0.436861\H,0,2.290904,4.49093,1.613809\H,0,2.066381,-2.4
99468,0.999876\H,0,0.501259,-2.912457,0.264667\H,0,0.90509,-1.49118,-1
.626517\H,0,2.604623,-2.804834,-2.49264\H,0,6.004322,-2.602117,-2.1888
55\H,0,4.7042,-3.647137,-2.82629\H,0,5.529197,-4.051004,-1.29471\H,0,3
.915573,-1.300577,0.74326\H,0,5.435571,-1.019534,-0.136754\H,0,5.19475
8,-2.540732,0.733378\H,0,0.35945,-2.013815,3.060354\H,0,0.318396,-0.24
6913,3.256676\H,0,-1.455655,-1.665783,1.357955\H,0,2.606652,-0.338444,
2.773813\H,0,-0.920975,0.966216,-0.131453\H,0,-2.755011,2.225264,-0.80
7088\H,0,-5.160092,2.532659,-1.334611\H,0,-3.492716,-1.773818,0.649801
\H,0,-7.631316,1.138202,-1.194465\H,0,-8.488732,-1.096566,-0.454625\H,
0,-6.393854,-2.541106,0.476229\\Version=ES64L-G09RevD.01\State=1-A\HF=
-1190.85705\RMSD=2.990e-09\PG=C01 [X(C26H26N2O1)]\\@
```

6.5.15 Conformation 15 (internal code: M0048): rel. energy 0.90 kcal mol^{-1}


```
1\1\GINC-HEISENBERG02\SP\RwB97XD TD-FC\tApr-CC-pVDZ/Auto\C26H26N2O1\MF
RESIA\17-May-2020\0\\# td=(nstates=60) wB97XD/TApr-cc-pVDZ/auto scrf=(
solvent=methanol) geom=connectivity\\RaputindolD_M0048_td_wB97XD_TApr-
cc-pVDZ_methanol\\0,1\C,0,1.359609,0.194411,0.730171\C,0,1.981883,0.03
949,-0.528819\C,0,2.566778,1.105155,-1.187542\C,0,2.517738,2.348886,-0
.539799\C,0,1.902162,2.529943,0.727975\C,0,1.31071,1.42309,1.368231\N,
0,3.013372,3.568891,-0.931639\C,0,2.734017,4.507701,0.034189\C,0,2.056
249,3.91899,1.069576\C,0,0.796712,-1.132232,1.217486\C,0,1.538736,-2.1
52838,0.311503\C,0,1.870932,-1.400336,-0.997939\C,0,3.036748,-1.954072
,-1.785567\C,0,4.336835,-1.92238,-1.45262\C,0,5.379888,-2.518463,-2.36
2443\C,0,4.889153,-1.306424,-0.193418\C,0,1.117875,-1.376641,2.704186\
C,0,-0.699836,-1.255464,1.019483\O,0,2.496302,-1.276802,2.997417\C,0,-
1.499272,-0.351863,0.438671\C,0,-2.952924,-0.459967,0.206985\C,0,-3.69
497,-1.625753,0.540565\C,0,-5.057833,-1.715946,0.322938\C,0,-5.704692,
-0.609402,-0.24706\C,0,-4.998929,0.568664,-0.596347\C,0,-3.614358,0.62
7263,-0.362073\N,0,-7.023751,-0.413183,-0.57229\C,0,-7.175358,0.841946
,-1.113481\C,0,-5.96347,1.48175,-1.149048\H,0,3.061367,0.97884,-2.1527
66\H,0,0.824136,1.540336,2.339956\H,0,3.506324,3.75173,-1.793789\H,0,3
.046556,5.539784,-0.099997\H,0,1.705113,4.415637,1.969983\H,0,2.472183
,-2.448174,0.811806\H,0,0.946986,-3.062824,0.135527\H,0,0.982276,-1.45
3984,-1.651112\H,0,2.777099,-2.441257,-2.731316\H,0,6.123369,-1.756922
,-2.652767\H,0,4.938945,-2.938001,-3.27775\H,0,5.936907,-3.319007,-1.8
46617\H,0,4.116658,-0.974806,0.509257\H,0,5.518175,-0.434462,-0.442521
\H,0,5.541947,-2.028268,0.324987\H,0,0.816317,-2.399509,2.9794\H,0,0.5
22605,-0.680603,3.3258\H,0,-1.118539,-2.201533,1.384037\H,0,2.774222,-
0.38844,2.735512\H,0,-1.048908,0.58295,0.088089\H,0,-3.181742,-2.48279
1,0.979192\H,0,-5.609155,-2.620686,0.584285\H,0,-3.053513,1.528138,-0.
624407\H,0,-7.767955,-1.083117,-0.440505\H,0,-8.15525,1.183862,-1.4358
42\H,0,-5.780189,2.484524,-1.525093\\Version=ES64L-G09RevD.01\State=1-
A\HF=-1190.8576797\RMSD=3.341e-09\PG=C01 [X(C26H26N2O1)]\\@
```
